# Cobalt-Catalyzed Hydrosilylation of Carbon Dioxide to the Formic Acid, Formaldehyde, and Methanol Level—How to Control the Catalytic Network?

**DOI:** 10.1021/jacsau.1c00350

**Published:** 2021-10-04

**Authors:** Hanna H. Cramer, Shengfa Ye, Frank Neese, Christophe Werlé, Walter Leitner

**Affiliations:** † Max Planck Institute for Chemical Energy Conversion, Stiftstr. 34−36, 45470 Mülheim an der Ruhr, Germany; ‡ Institut für Technische und Makromolekulare Chemie (ITMC), RWTH Aachen University, Worringer Weg 2, 52074 Aachen, Germany; § State Key Laboratory of Catalysis, Dalian Institute of Chemical Physics, Chinese Academy of Sciences, Dalian 116023, China; ∥ Max-Planck-Institut für Kohlenforschung, Kaiser-Wilhelm-Platz 1, D-45470 Mülheim an der Ruhr, Germany; ⊥ Ruhr University Bochum, Universitätsstr. 150, 44801 Bochum, Germany

**Keywords:** molecular control, carbon dioxide, cobalt, homogeneous, catalysis, reduction reactions, hydrosilylation

## Abstract

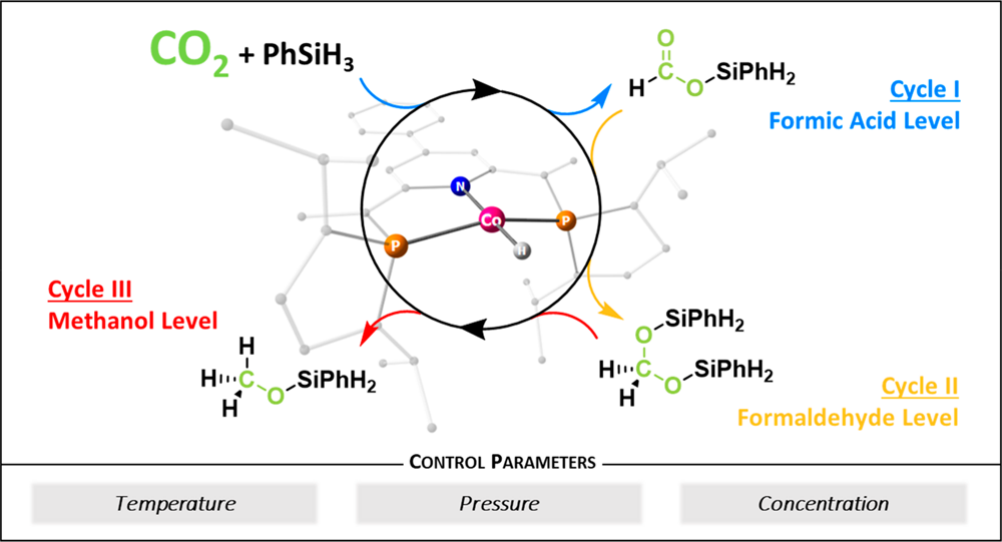

The selective hydrosilylation of carbon dioxide (CO_2_) to either the formic acid, formaldehyde, or methanol level using a molecular cobalt(II) triazine complex can be controlled based on reaction parameters such as temperature, CO_2_ pressure, and concentration. Here, we rationalize the catalytic mechanism that enables the selective arrival at each product platform. Key reactive intermediates were prepared and spectroscopically characterized, while the catalytic mechanism and the energy profile were analyzed with density functional theory (DFT) methods and microkinetic modeling. It transpired that the stepwise reduction of CO_2_ involves three consecutive catalytic cycles, including the same cobalt(I) triazine hydride complex as the active species. The increasing kinetic barriers associated with each reduction step and the competing hydride transfer steps in the three cycles corroborate the strong influence of the catalyst environment on the product selectivity. The fundamental mechanistic insights provide a consistent description of the catalytic system and rationalize, in particular, the experimentally verified opportunity to steer the reaction toward the formaldehyde product as the chemically most challenging reduction level.

## Introduction

The catalytic reduction of carbon dioxide (CO_2_) to value-added products is essential for utilizing renewable energy sources and starting materials in sustainable energy systems and chemical industries. Transition metal complexes can facilitate the transformation of CO_2_ into valuable chemicals on multiple oxidation levels. Many catalytic systems are reported to selectively catalyze the two-electron reduction of CO_2_ yielding derivatives of formic acid (HCOOH),^1−12^ while significantly fewer enable further reduction to the formaldehyde^13−20^ (H_2_CO) or methanol^21−29^ (H_3_COH) product platforms. With photosynthesis, nature has developed an ingenious but highly complex mechanism to exploit the intermediate reduction level of formaldehyde in the form of carbohydrates (C_*n*_H_2*n*_O_*n*_). In contrast, the thermodynamically favored over-reduction of formaldehyde and its derivatives to the methanol level often prevails with chemical catalysts, making this important product platform particularly difficult to exploit *via* direct reduction routes. Controlling selectivity in catalytic CO_2_ reduction by unraveling the precise interaction of elementary steps and reaction parameters thus remains a significant chemical challenge.

**Figure 1:**
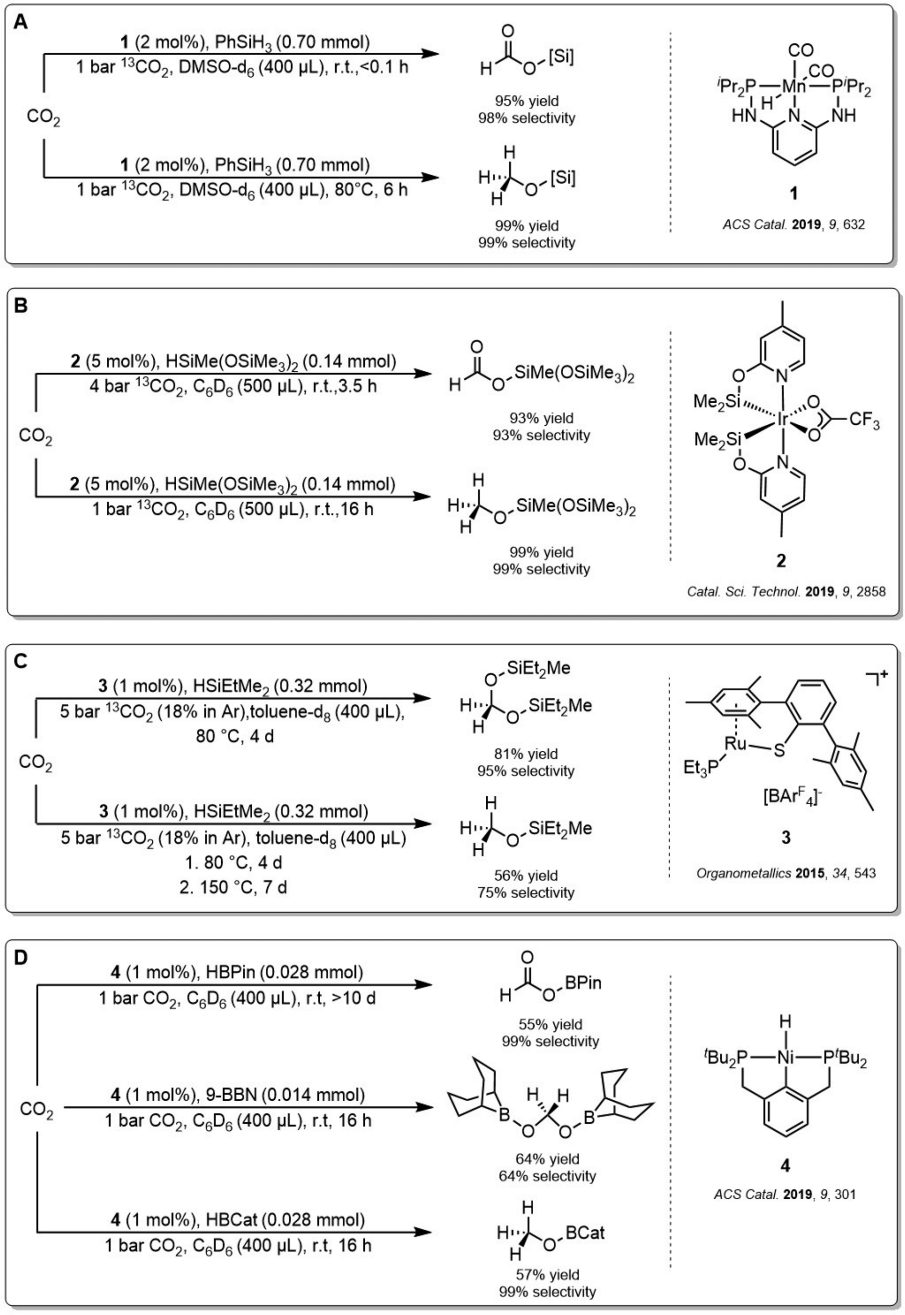
Selected examples of transition metal catalysts used for CO_2_ reduction to products on multiple oxidation levels.

While large-scale applications for CO_2_ reduction require the use of “green” hydrogen, catalytic reductions using activated hydrides such as boranes or silanes are very useful as molecular probes to expand our knowledge in this field.^30−38^ Their structures inherently enable heterolytic activation pathways to deliver hydrides for the two-electron reduction together with an oxophilic counterpart.^39^ Examples that facilitate the transformation of CO_2_ with such reducing agents to control the selectivity to two different reduction levels are shown in Figure 1A–C. The manganese pincer complex **1** reported by Gonsalvi was found capable of reducing CO_2_ to either the formate level at 25 °C or the methanol level at 80 °C.^40^ Selective formation of either formate or methanol derivatives was also reported for the hydrosilylation of CO_2_ by the iridium complex **2**, whereby the CO_2_ pressure determined the product preference.^41^ The tethered ruthenium–sulfur complex **3** was found to facilitate hydrosilylation of CO_2_ to the formaldehyde or the methanol level, as reported by the group of Oestreich.^42^ Here, the reaction temperature was the main parameter determining the selectivity. Selective arrival at three different reduction levels was reported for the nickel hydride complex **4** used in the hydroboration of CO_2_ but required adjustment of the reductant (Figure 1D).^43^

**Figure 2:**
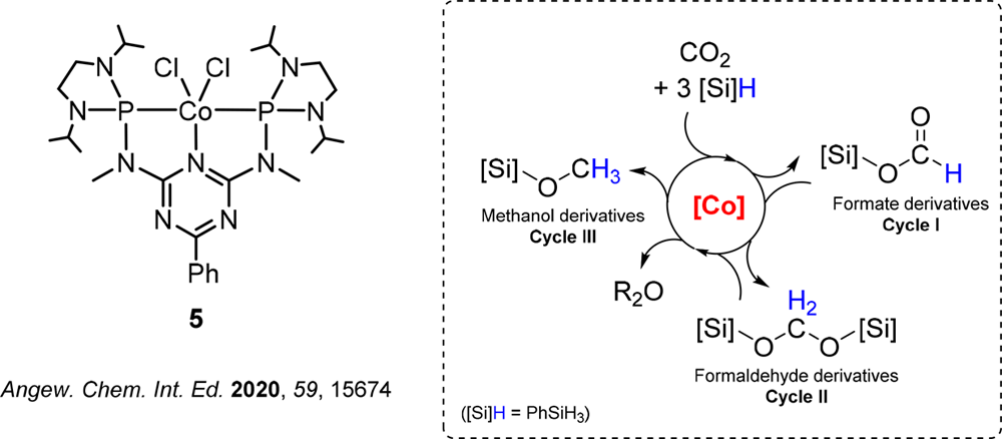
Mechanistic considerations of the reduction of CO_2_ with **5** through three consecutive catalytic cycles.

Our group recently reported that the cobalt triazine pincer complex **5** is a suitable catalyst for the selective hydrosilylation of CO_2_ to either formic acid, formaldehyde, or methanol derivatives using phenylsilane (PhSiH_3_) as a reducing agent (Figure 2). The catalyst was active at mild reaction conditions (r.t. to 80 °C, 1–40 bar), low catalyst loadings (0.2 mol %), and various solvents in the presence of potassium *tert*-butanolate (KO^*t*^Bu). The formation of silylated products could be steered precisely by adjusting the temperature, solvent, and pressure as control parameters.^44^ In general, higher temperatures, lower CO_2_ pressures, and higher concentrations favored the reduction to the formaldehyde and ultimately toward the methanol level. Higher CO_2_ pressures stopped the reaction at the formate level, however. Formate and methanol derivatives were obtained with selectivities of 98 and 99%, respectively. Notably, the most challenging formaldehyde level could also be reached with high selectivity of 71%, demonstrating the possibility of targeting the three different product platforms in high yields without changing the nature of the reducing agent.

Based on mechanistic investigations of related hydrosilylation^40^ and hydroboration^45^ catalysts for CO_2_ reduction to arrive ultimately at methanol derivatives, it can be suggested that the stepwise six-electron reduction achieved with catalyst **5** proceeds *via* a cascade of three individual catalytic cycles. CO_2_ is reduced first to silyl formate in this consecutive process, which then acts as the substrate to be reduced to the formaldehyde as silylated acetal, which is ultimately reduced to the methanol level, as seen in Figure 2. We, therefore, aimed to rationalize the selectivity control through temperature, CO_2_ pressure, and concentration in a combined experimental and theoretical effort by determining the detailed influence of these parameters on the relative kinetic barriers of each of the three consecutive cycles. The nature of the active catalyst species formed from complex **5** and the substrate activation processes were elucidated by synthesis, spectroscopy, and reactivity studies.

Based on the obtained insights, we subsequently investigated the catalytic mechanism with density functional theory (DFT) methods analyzing the three catalytic cycles individually and combining them to the energy profile for the overall reaction sequence. Finally, we simulated the product distribution under different sets of reaction parameters using microkinetic modeling of the reaction sequence compared to the experimental observations. The data provide a coherent description of the energy landscape, explaining in detail how fine adjustments of the control parameters lead to a high selectivity for the three different CO_2_ reduction levels and suggesting general guidelines on how to optimize for the formaldehyde product platform. This fundamental knowledge provides tools for designing and developing new adaptive catalysts that allow selective access to the different C_1_-reduction products.

## Results and Discussion

### Identification of the Active Species

**Figure 3:**
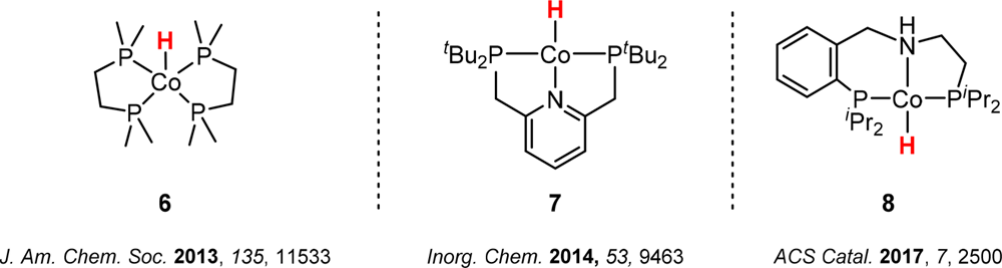
Selected examples of Co(I) hydride complexes active in CO_2_ reduction catalysis.

We first addressed the question of possible activation pathways of the precatalyst **5**. In many examples of catalytic CO_2_ reduction with molecular cobalt complexes, Co(I) hydride complexes are considered a significant part of the catalytic cycle as the active species.^46,7,47−50^ For example, complex **6** was reported as an active catalyst for the hydrogenation of CO_2_ to formate in the presence of Verkade’s base, achieving high turnover frequencies of 74 000 h^–1^ at room temperature and 10 bar of CO_2_/10 bar of H_2_ (Figure 3, left).^7^ Computational and thermodynamic studies indicated an *outer-sphere* activation of CO_2_ by the cobalt hydride complex with hydride transfer as a rate-limiting step of the reaction.^51,52^ Complex **7** (Figure 3, middle) was used as a catalyst for the hydrosilylation of CO_2_, leading to a mixture of silyl formates, bis(silyl)acetals, and methoxysilanes.^47^ More recently, cobalt-catalyzed N-formylation of amines in the presence of CO_2_ and H_2_ was reported.^50^ The authors suggested *in situ* formation of the hydride complex **8** (Figure 3, right) as the active species.

Since complex **5** used as a precursor complex contains Co in the oxidation state +II, the formation of a cobalt(I) complex requires its reduction prior to catalysis. This seems well in line with the catalytic results, where the activity of **5** was strongly enhanced in the presence of potassium *tert*-butanolate (KO^*t*^Bu).^44^ Alkali *tert*-butanolates were previously reported to activate silanes to form transient MH/alkoxysilane complexes as strong reducing agents.^53^ Following these lines, the *in situ* formation of cobalt(I) hydride complex **9** under catalytic conditions was considered. Therefore, we set out to synthesize **9** to investigate its performance as a kinetically competent species in CO_2_ hydrosilylation.

**Scheme 1:**
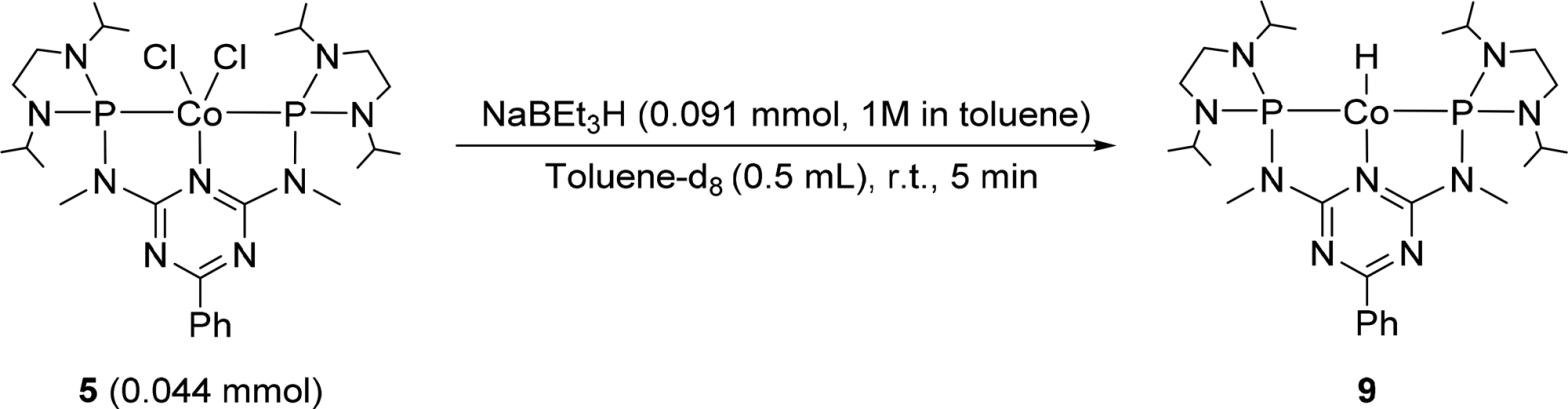
Generation of Cobalt(I) Hydride Complex **9**

The Co(I) hydride complex **9** was prepared by following a modified literature procedure reported for the synthesis of the structurally related complex **7**.^54^ The addition of 2.1 equiv of sodium triethylborohydride to complex **5** at room temperature in C_6_D_6_ led to the formation of a dark green solution containing **9** (Scheme 1).

The structure of complex **9** was confirmed by NMR spectroscopy. The ^1^H NMR spectrum of **9** corroborates a diamagnetic *C*_2*v*_-symmetric compound, and the resonances corresponding to the pincer ligand are found in the expected ranges. A high field signal at −13.31 ppm appears as a triplet with a ^2^*J*_P,H_ coupling constant of 56 Hz and can be assigned to the hydride ligand (Figure S1). The presence of a terminal cobalt hydride bond was further confirmed by infrared spectroscopy showing a medium intensity band at 1746 cm^–1^, which agrees with previously reported values for related complexes.^55,54^ The identity of **9** was further supported by HR-MS analysis showing the mass corresponding to the protonated molecular complex [C_27_H_49_N_9_P_2_Co]^+^.

**Scheme 2:**
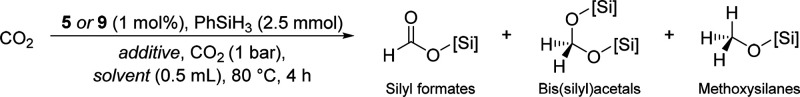
Hydrosilylation of CO_2_ Catalyzed by Complexes **5** and **9**

**Figure 4:**
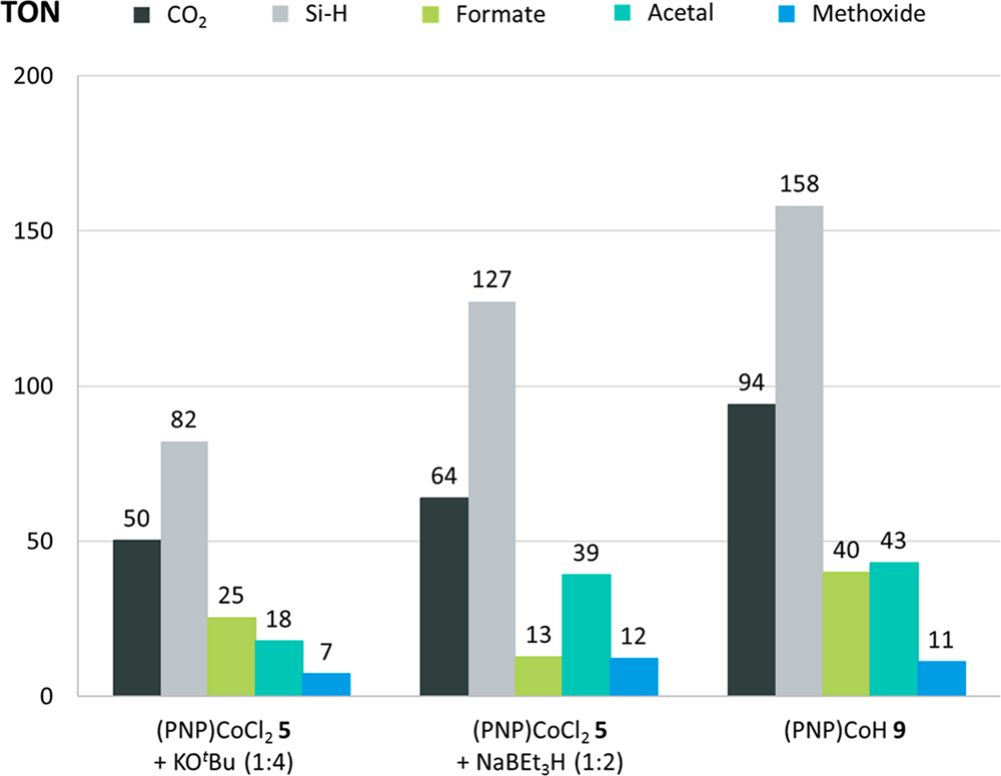
Catalytic CO_2_ hydrosilylation with PhSiH_3_ (2.5 mmol), CO_2_ (1 bar, continuous gas stream), 80 °C, 4 h. Column 1: **5** (1 mol %), KO^t^Bu (4 mol %), C_6_D_6_ (0.5 mL); column 2: **5** (1 mol %), NaBEt_3_H (1 M in toluene, 2.1 mol %), toluene-*d*_8_; column 3: **9** (1 mol %), C_6_D_6_ (0.5 mL).

The catalytic competence of **9** was examined in the hydrosilylation of CO_2_ with phenylsilane at 1 bar of CO_2_ applied as a continuous gas stream and 1 mol % catalyst loading at 80 °C in comparison with **5** in the presence of KO^*t*^Bu or NaBEt_3_H as an additive (Scheme 2). The quantities of silyl formates, bis(silyl)acetals, and methoxysilanes were determined by ^13^C{^1^H} NMR spectroscopy,^47^ and the observed turnover numbers (TONs) are shown in Figure 4. Within 4 h, a conversion of 35% of the Si–H units was observed, corresponding to a turnover of 158 Si–H units per catalyst (column 3). A total of 94 moles of CO_2_ per mole of cobalt catalyst was converted, leading to silyl formates and bis(silyl)acetals as main products in selectivities of 43% (TON = 40) and 46% (TON = 43), respectively, together with minor amounts of silyl methoxides (11%, TON = 11). Thus, compound **9** reaches even higher turnover numbers for the reduction process than complex **5** in the presence of 4 mol % KO^*t*^Bu under the same reaction conditions (column 1). The isolated complex **9** also shows higher activity than an *in situ* preparation from **5** and NaBEt_3_H (column 2). These results indicate that **9** acts as the active species in the catalytic hydrosilylation of CO_2_ and that the presence of KO^*t*^Bu is required mainly for its *in situ* formation from **5** but does not contribute to the actual hydride transfer during catalytic turnover.

### Activation of Phenylsilane and CO_2_ by Co–H Complex **9**

**Scheme 3:**
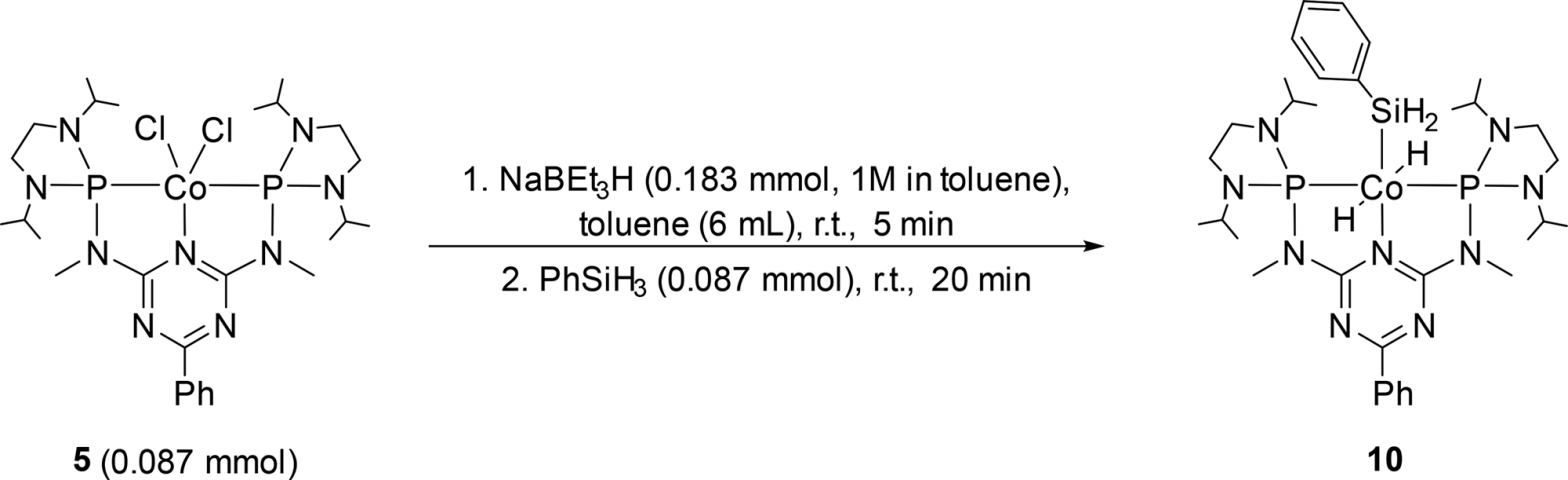
Preparation of the Cobalt(III) Dihydride Complex **10**

First, we evaluated the possibility that the activation of phenylsilane by **9** occurs before the reaction with CO_2_, since the structurally related complex **7** was reported to activate hydrosilanes by oxidative addition to form Co(III) dihydride complexes.^47^ In full analogy, adding phenylsilane to the *in situ* prepared complex **9** at room temperature led to the formation of the cobalt(III) complex **10** (Scheme 3, see the SI for details).

In the ^1^H NMR spectrum of **10**, the triplet resonance at −9.39 ppm exhibits a ^2^*J*_P,H_ coupling constant of 47 Hz. The chemical shift is similar to related cobalt(III) complexes and can be assigned to the two hydride ligands at the metal center in a mutual *trans*-position.^47^ A second triplet at 4.40 ppm with a smaller ^3^*J*_P,H_ coupling constant of 11 Hz corresponds to the hydrosilyl ligand. The ^31^P{^1^H} NMR spectrum features a resonance at 174.0 ppm corresponding to the triazine ligand. The symmetric and asymmetric Co–H stretching frequencies appear at 2026 and 1758 cm^–1^ in the IR spectrum, respectively. This reactivity indicates that cobalt(III) is accessible *via* oxidative addition of Si–H bonds with the triazine-based ligand platform.

The reactivity of **10** toward CO_2_ was found to be rather sluggish, however. When **10** was exposed to 1 bar of ^13^CO_2_, NMR spectroscopic analysis revealed that conversion of CO_2_ into a formate group required a reaction time of 2 days (see the SI for details). Considering that conversion of CO_2_ into formate derivatives was previously observed to be completed after 4 h at room temperature with 0.2 mol % catalyst loading,^44^ the low reactivity of **10** toward CO_2_ indicates that an *outer-sphere* CO_2_ activation pathway at the Co(III) species is unlikely to account for the catalytic process. Therefore, **10** is considered as an off-cycle species that can regenerate the active species **9** rather than being a catalytically competent intermediate.

**Scheme 4:**
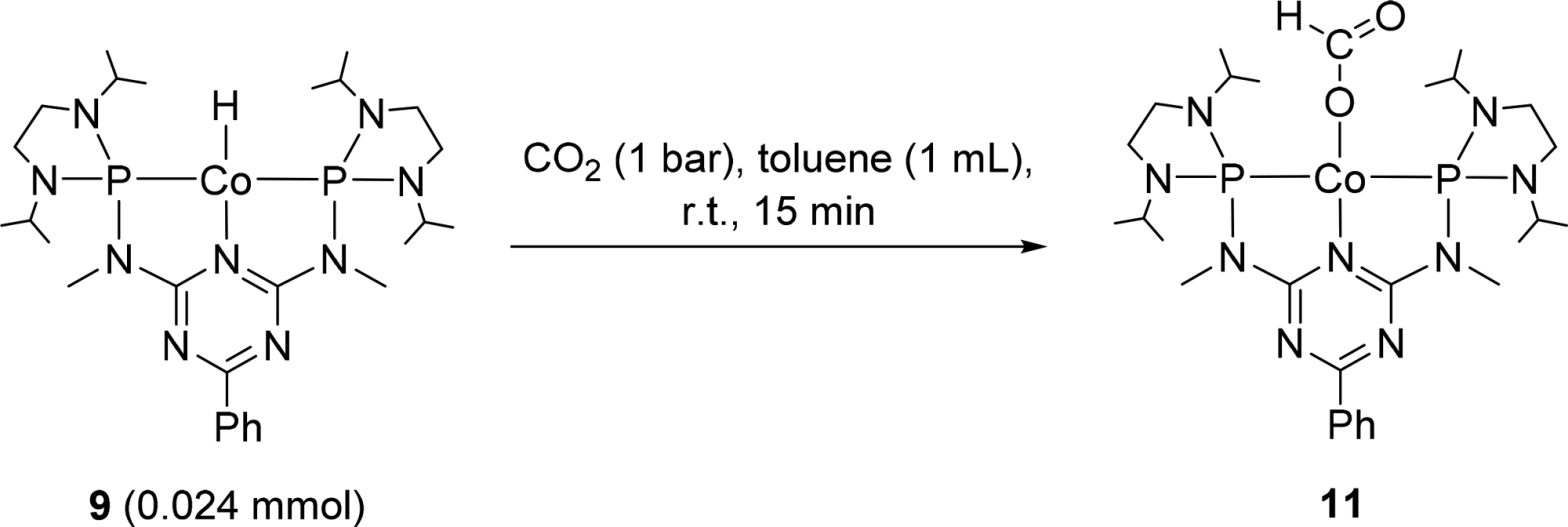
Preparation of the Formate Complex **11**

Next, we investigated the activation of CO_2_ by complex **9** as the initial step of the catalytic cycle. The insertion of CO_2_ into metal hydride bonds to furnish formate complexes is well-known and considered as an important step in CO_2_ reduction catalysis.^46−49,52,50,56,40,41^ Complex **9** reacts readily under 1 bar of CO_2_, leading to the formate complex **11** (Scheme 4, see the SI for details).

The NMR spectra of **11** reveal a diamagnetic *C*_2*v*_-symmetric product, and the resonances of the ligand backbone appear in the expected ranges. The ^1^H and ^13^C resonances of the formate ligand were located at 7.83 and 173.9 ppm, respectively, and were assigned by 2D NMR experiments and ^13^C-labeling. The ^13^C isotopologue prepared from **5** (see the SI for details) shows a ^1^*J*_C,H_ coupling of 202 Hz in the ^1^H NMR spectrum, while the ^13^C resonance at 173.9 ppm associated with the formate carbon atom is strongly increased in intensity (Figures S13 and S14). The symmetric and asymmetric C–O vibrations are visible at 1380 and 1593 cm^–1^ in the IR spectrum (Figure S15).

**Scheme 5:**
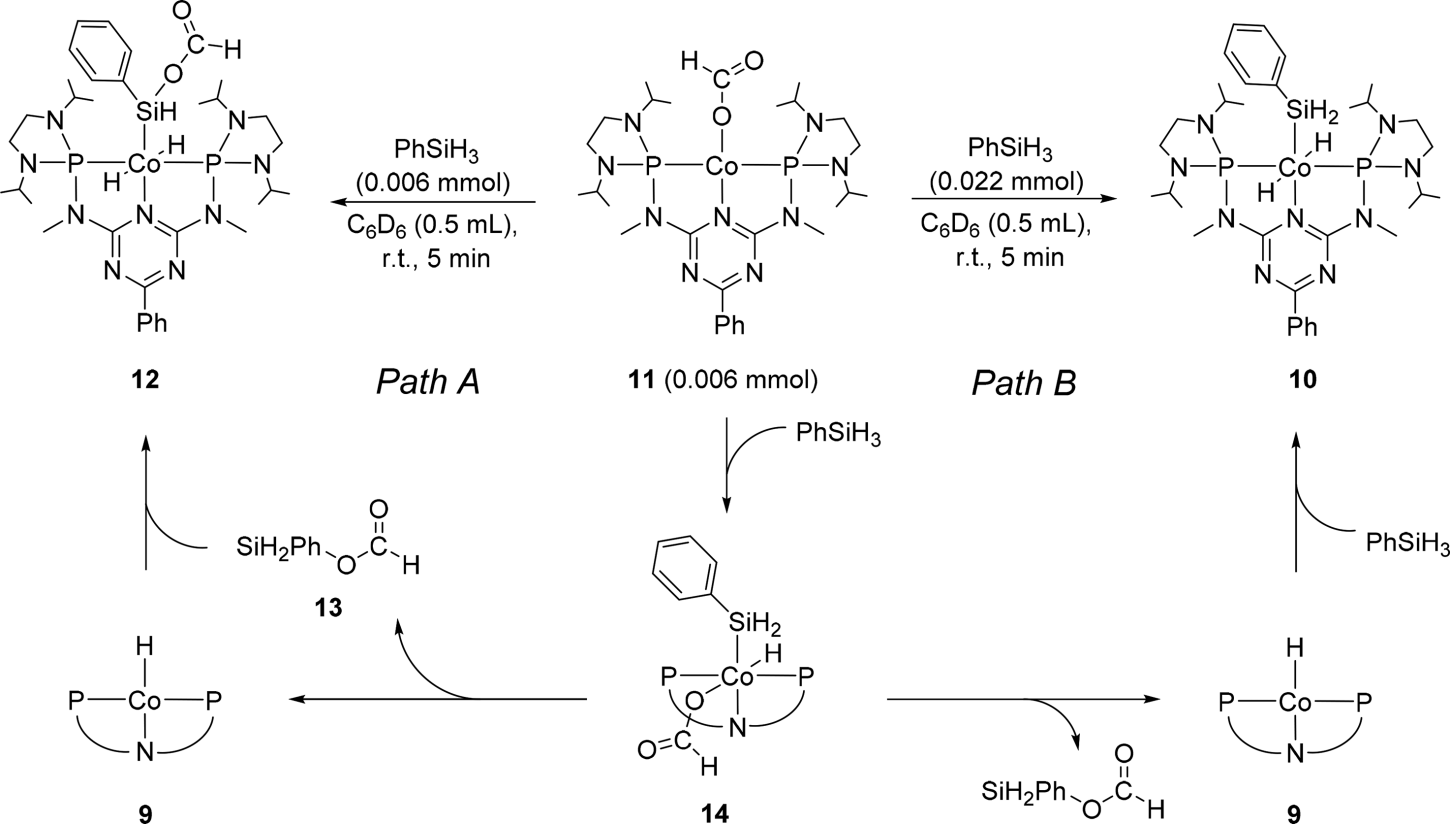
Reaction of Complex **11** with Different Amounts of PhSiH_3_ (top) and Possible Reaction Pathways (bottom)

Subsequently, we reacted the ^13^C isotopologue of **11** with PhSiH_3_ to explore the possibility of silylation of the formate group (Scheme 5, path A). When **11** was reacted with a stoichiometric amount of phenylsilane, rapid conversion to a new product was observed, which was identified as complex **12** by NMR spectroscopic analysis. The resonances corresponding to the two hydride ligands appear as a triplet with ^2^*J*_P,H_ = 47 Hz at −8.80 ppm. The Si–H unit corresponds to resonance at 5.79 ppm, while the formate proton resonance is visible at 8.48 ppm. The resonance of the formate carbon atom is visible at 162.1 ppm in the ^13^C{^1^H} NMR spectrum, while the ^1^H–^13^C HMBC NMR spectrum reveals a ^3^*J*_C,H_ coupling with the Si–H unit. Thus, the data indicate the presence of a silyl formate group, which is supported by comparison with chemical shifts of silyl formates reported in previous works that appear in a similar range.^57,40^ A possible pathway for the formation of **12** would start with oxidative addition of phenylsilane, followed by reductive elimination of silyl formate **13** and regeneration of **9**. Subsequently, activation of the Si–H bond of the product in a second oxidative addition step leads to **12**.

In contrast, upon addition of a slight excess of phenylsilane to **11** at room temperature, the immediate formation of complex **10** as the main product can be observed (Scheme 5, path B), while complex **12** was not visible. The isotope-labeled formate carbon atom can be assigned to a ^13^C resonance at 162.2 ppm. The ^1^H–^13^C HMBC NMR spectrum exhibits ^3^*J*_C,H_ coupling with a ^1^H resonance at 5.82 ppm that corresponds to a hydrosilyl group, suggesting silylation of the formate group in addition to the formation of **10**. Complex **10** could form in a similar pathway to **12**, while the last step involves oxidative addition of phenylsilane instead of silyl formate. Thus, the reactivity of **11** toward phenylsilane indicates the transformation of the formate group into a silyl formate unit and the intermediate generation of **9**, supporting the key role of **9** and **11** in the catalytic cycle.

**Figure 5:**
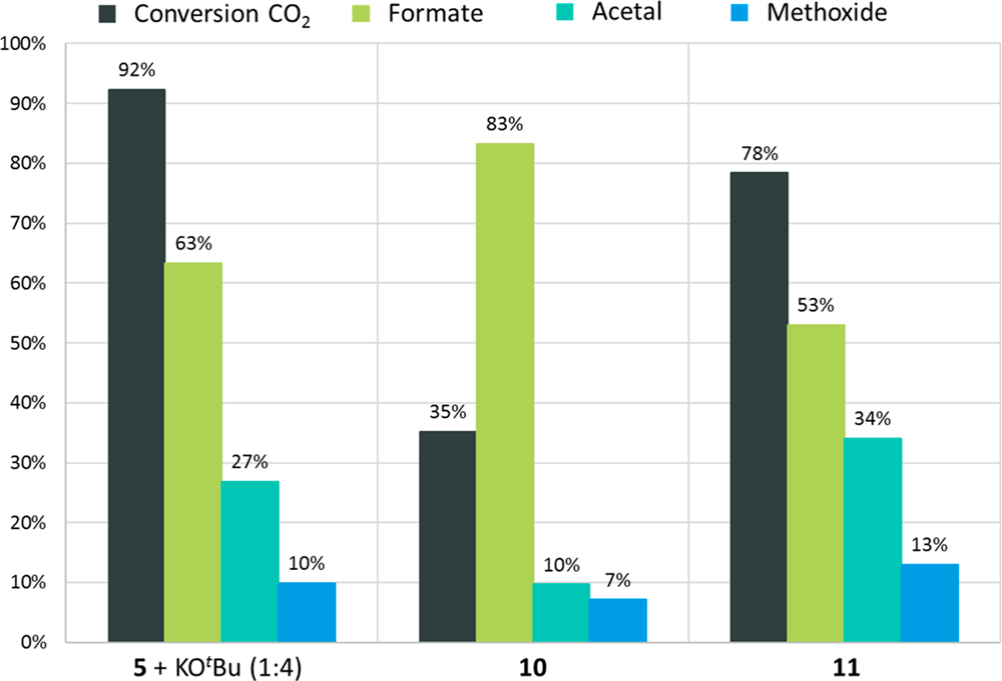
Catalytic CO_2_ hydrosilylation with PhSiH_3_ (2.5 mmol), ^13^CO_2_ (1 bar), C_6_D_6_, 80 °C, 4 h. Column 1: **5** (0.2 mol %), KO^*t*^Bu (0.4 mol %), C_6_D_6_ (0.5 mL);^44^ column 2: **10** (0.2 mol %); column 3: **11** (0.2 mol %).

We tested complexes **10** and **11** as catalysts for CO_2_ hydrosilylation in a closed Schlenk tube containing 1 bar of ^13^CO_2_ (see the SI for details). The products were quantified by ^13^C{^1^H} NMR spectroscopy, and the CO_2_ conversion was determined from the amount of silylated products formed.^44^ After 4 h at 80 °C, 35% of ^13^CO_2_ conversion and 83% selective formation of silyl formate were observed in the case of **10**, whereas 78% ^13^CO_2_ conversion and 53% selective formation of silyl formate could be detected for **11** (Figure 5). The catalytic performance of **10** is thus low compared to **11**, while the latter exhibits an activity comparable to complex **5** in the presence of KO^*t*^Bu.^44^ These results are consistent with the slow reactivity of **10** with CO_2_ (vide supra) and the rapid formation of **12** from **11** (Scheme 5).

Complex **12** is only observed in the presence of stoichiometric amounts of PhSiH_3_ (Scheme 5), which suggests that it is not formed under catalytic conditions under a large excess of PhSiH_3_ with respect to the catalyst. The significantly lower activity of **10** compared to **9** and **11** indicates that **10** is not contributing to a major pathway, since otherwise lower conversion would be obtained. Furthermore, NMR spectroscopic analysis of the reaction mixture under catalytic conditions did not reveal any signals associated with the presence of either **10** or **12**. Low relative energies calculated for **10** and **12** as compared to **9** and **11**, respectively, further suggest that they are not catalytically competent due to their high stability (see the SI for details). Therefore, complexes **9** and **11** were chosen as the key reactive intermediates for the description of the catalytic cycle, while **10** and **12** were considered as off-cycle species that are not directly involved in the product formation.

The high conversions observed when using **5** and KO^*t*^Bu indicate the formation of **9** in the reaction mixture. The strongly reducing conditions in the presence of *tert*-butanolates and PhSiH_3_ might favor a reduction of Co(II) to Co(I).^53^ NMR spectroscopic analysis of the reaction mixture containing **5**, PhSiH_3_ (2 equiv), and KO^*t*^Bu (2 equiv) indicated the presence of **10** (see the SI for details), which suggests a reaction pathway that involves the initial reduction of **5** to **9**, followed by oxidative addition of PhSiH_3_ resulting in **10**. Such an alternative reaction pathway generating **10** would rationalize the high catalytic activity of **5** in the presence of KO^*t*^Bu.

### Computational Mechanistic Studies

Based on the experimentally verified intermediates **9** and **11** and their reactivity toward PhSiH_3_ and CO_2_, a possible catalytic cycle was investigated with density functional methods on the B3LYP/D3BJ level of theory for optimization and frequency calculations with the def2-TZVP and the def2-SVP basis sets. Under conditions with an excess of phenylsilane compared to CO_2_, we considered the Si–H activation of phenylsilane to be the dominant pathway compared to Si–H activation of **13** and other hydrosilanes generated during the reaction (see the SI for details). Therefore, we chose to study the formation of the monosubstituted product **13** in the computational analysis as a model to describe the complex reaction network. The calculations were performed using a conductor-like polarizable continuum model (CPCM) with benzene as a typical low-polarity solvent, in which all three reduction levels can be obtained experimentally.^44^ It was also used in the experimental analysis of the mechanism for the synthesis of **9**, **11**, and **14**.

### Cycle 1: Catalytic Formation of Silyl Formate

**Scheme 6:**
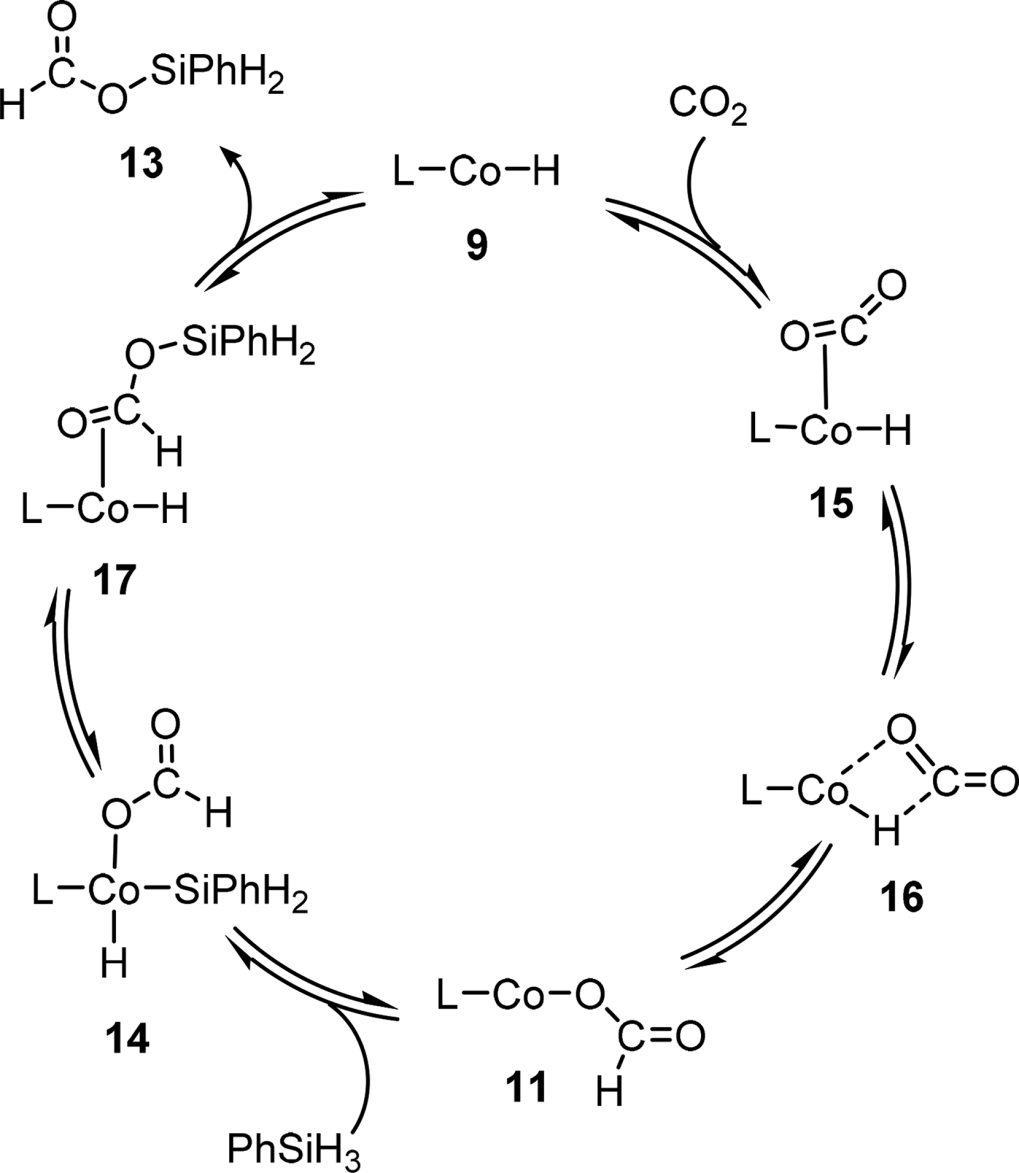
Catalytic Cycle of the CO_2_ Hydrosilylation to Silyl Formate **13** Based on the Experimentally Identified Intermediates **9** and **11** Used in the Computational Analysis

Two possible pathways are in principle conceivable for the generation of formate species from CO_2_ in homogeneous catalysis.^49,52,56^ In the *inner-sphere* process, activation of CO_2_ occurs *via* coordination to the metal and subsequent migratory insertion. Alternatively, the *outer-sphere* pathway involves nucleophilic attack of the metal hydride at the weakly Lewis acidic carbon atom of noncoordinated CO_2_. In the case of complex **9**, all attempts to locate a local minimum corresponding to an *outer-sphere* activation of CO_2_ resulted in dissociation of the two reactive components during the geometry optimization, and an *inner-sphere* process was thus considered more favorable. Scheme 6 depicts the corresponding catalytic cycle used as a basis for structural and energetical analysis.

**Figure 6:**
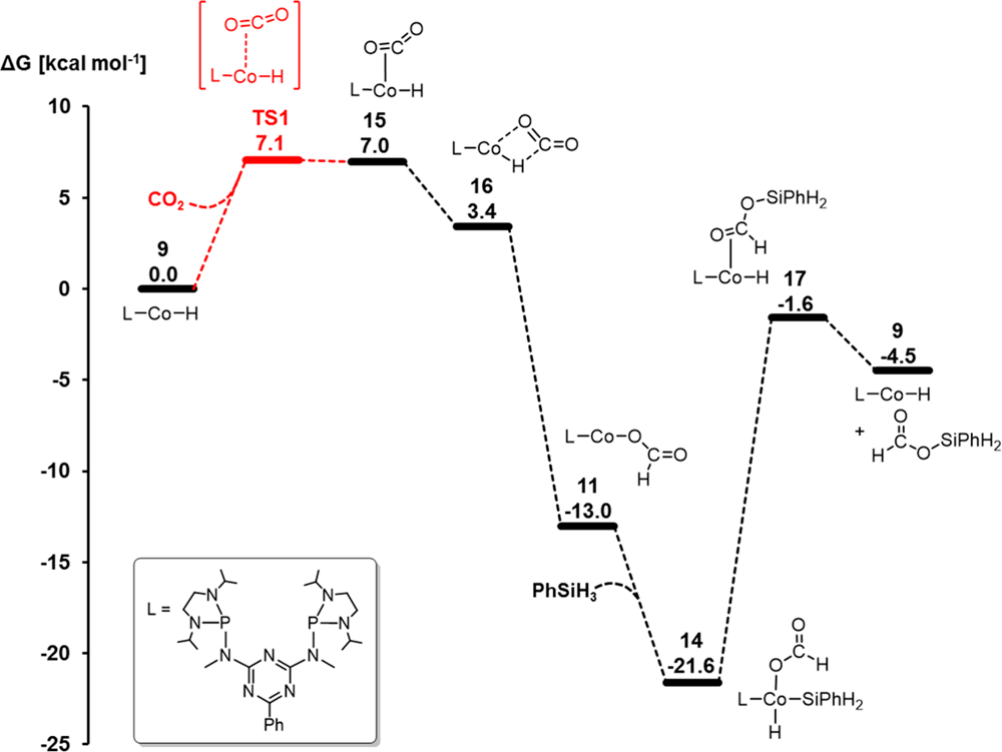
Relative Gibbs free energies [kcal mol^–1^] (B3LYP-D3BJ/def2-TZVP (selected atoms), def-SVP) for the hydrosilylation of CO_2_ catalyzed by **9**.

The first step in the formation of **11** was found to proceed *via* coordination of CO_2_ in a *side-on* η^2^-mode at complex **15** as a local minimum on the potential energy surface (PES, Figure 6). The alternative η^1^-O and η^1^-C coordination modes were considered but either led to dissociation during the geometry optimizations or were not connected to the transition states relevant for the hydride transfer, respectively. The coordination is slightly endothermic and proceeds *via* the transition state **TS1**, in which the imaginary frequency involves bending of the CO_2_.

**Figure 7:**
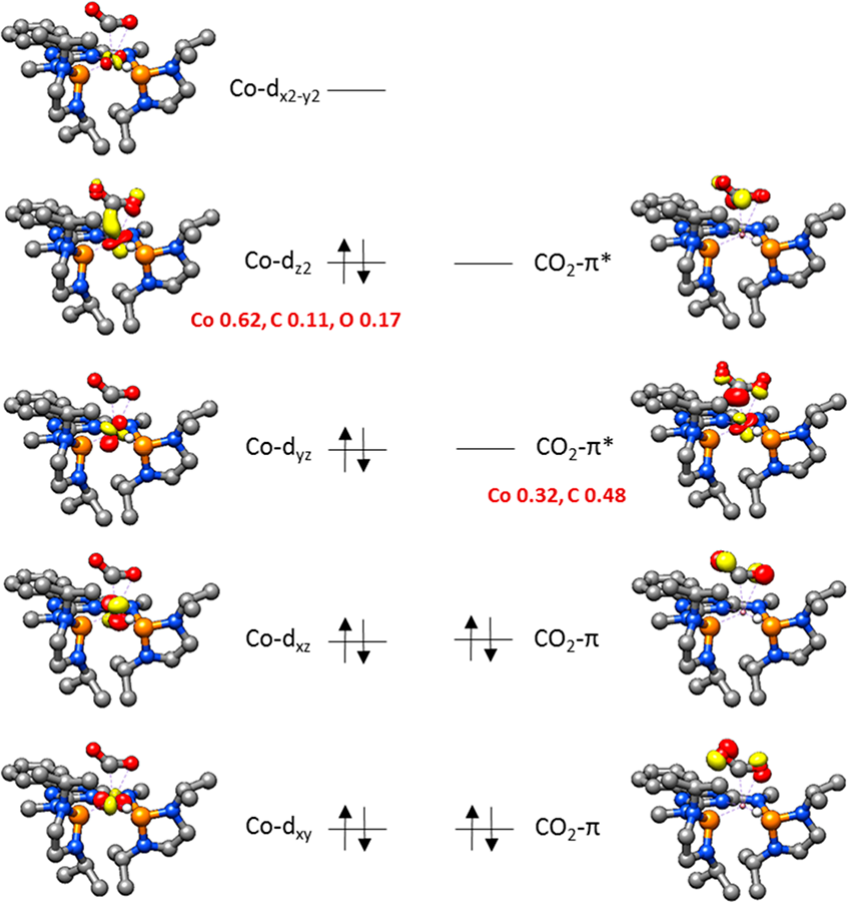
Electronic structure of **15**.

Complex **15** has a slightly distorted square pyramidal coordination geometry, whereby CO_2_ is aligned along the P–Co–P axis and exhibits significant bending (137.6°). The coordinated C–O bond is slightly elongated at 124 pm as compared to the noncoordinated one at 121 pm. The Co–C and Co–O1 distances are 200 and 223 pm, while the second Co–O2 distance is much longer in agreement with the lack of an additional interaction (297 pm). Analysis of the frontier orbitals confirms a mixing of the electron-donating cobalt d_*z*^2^_ orbital and the electron-accepting *in-plane* π*-orbital of the CO_2_ (Figure 7). The donation of electron density from the cobalt d_*z*^2^_ orbital into the antibonding *in-plane* π*-orbital (LUMO) is also manifested in the negative sum of the Mulliken gross atomic charges of CO_2_ (−0.48). Analysis of the orbital contributions reveals that both the C and the O atom participate in the interaction. However, only a partial electron transfer to the CO_2_ occurs, and cobalt can be considered to remain largely in its oxidation state of +I.

A decrease of the OCO angle is often associated with CO_2_ reduction processes, since it lowers the LUMO’s energy and also leads to more pronounced localization at the carbon atom. Both of these factors lead to enhanced electrophilicity of the carbon atom and thus facilitate nucleophilic attack.^58^ The enhanced carbon weight of the *in-plane* π*-orbital at the carbon atom also increases the spatial overlap with the d_*z*^2^_ orbital and thus further lowers the LUMO’s energy. Consequently, the nucleophilic attack of the hydride to the carbon atom corresponding to the migratory insertion occurs as a slightly exothermic spontaneous process (Figure 6). The initially formed structure **16** relaxes to the experimentally identified formate complex **11**.

The subsequent silane activation and product release could occur *via* a σ-metathesis pathway, as discussed in previous reports.^59,60,35,41^ However, thorough scans of the potential energy surfaces indicated far higher energy of intermediates and transition states than the oxidative addition/reductive elimination pathway. Additionally, the reactivity of **9** and **11** with hydrosilanes yielding **10** and **12** (Scheme 5) demonstrates the possible oxidative addition to the cobalt(I) complexes. The oxidative addition at **11** to form **14** is exergonic and occurs without a noticeable barrier. The primary product **17** arising from the reductive elimination contains the η^2^-C=O-bound silyl formate ester, whose release of product **13** regenerating the Co–H complex **9** is again slightly exergonic.

The driving force for the catalytic turnover of the low-lying intermediate **14** is provided by the exergonic overall catalytic process to convert CO_2_ and PhSiH_3_ to **13** with a Gibbs free energy of −4.5 kcal mol^–1^. This is very different from CO_2_ hydrogenation, where the formic acid formation is uphill under standard conditions.^3,61−63^ The strong Si–O bond provides the additional driving force. The possibility of reductive elimination of formic acid from an alternative isomer of **14** was found to be less favorable than the generation of silyl formate **13** (see the SI for details).

The rate of catalytic turnover can be estimated by the energy span δ*E* that is calculated from the most stable intermediate (turnover determining intermediate, TDI) and the highest transition state on the potential energy surface (turnover determining transition state, TDTS). If the TDI follows after the TDTS, the Gibbs free energy of the reaction is added to their energetic difference.^64^
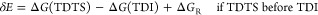
1
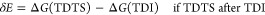
2The energy span from the first cycle can be calculated from **14** as the TDI and **TS1** as the TDTS. Since the TDI follows after the TDTS, the Gibbs free energy of Δ*G*_R_ = −4.5 kcal mol^–1^ is taken into account, and an energy span of 24.2 kcal mol^–1^ can be calculated using eq 1, which is in line with the experimental conditions for catalytic turnover.

### Cycle 2: Catalytic Formation of Bis(silyl)acetal

**Scheme 7:**
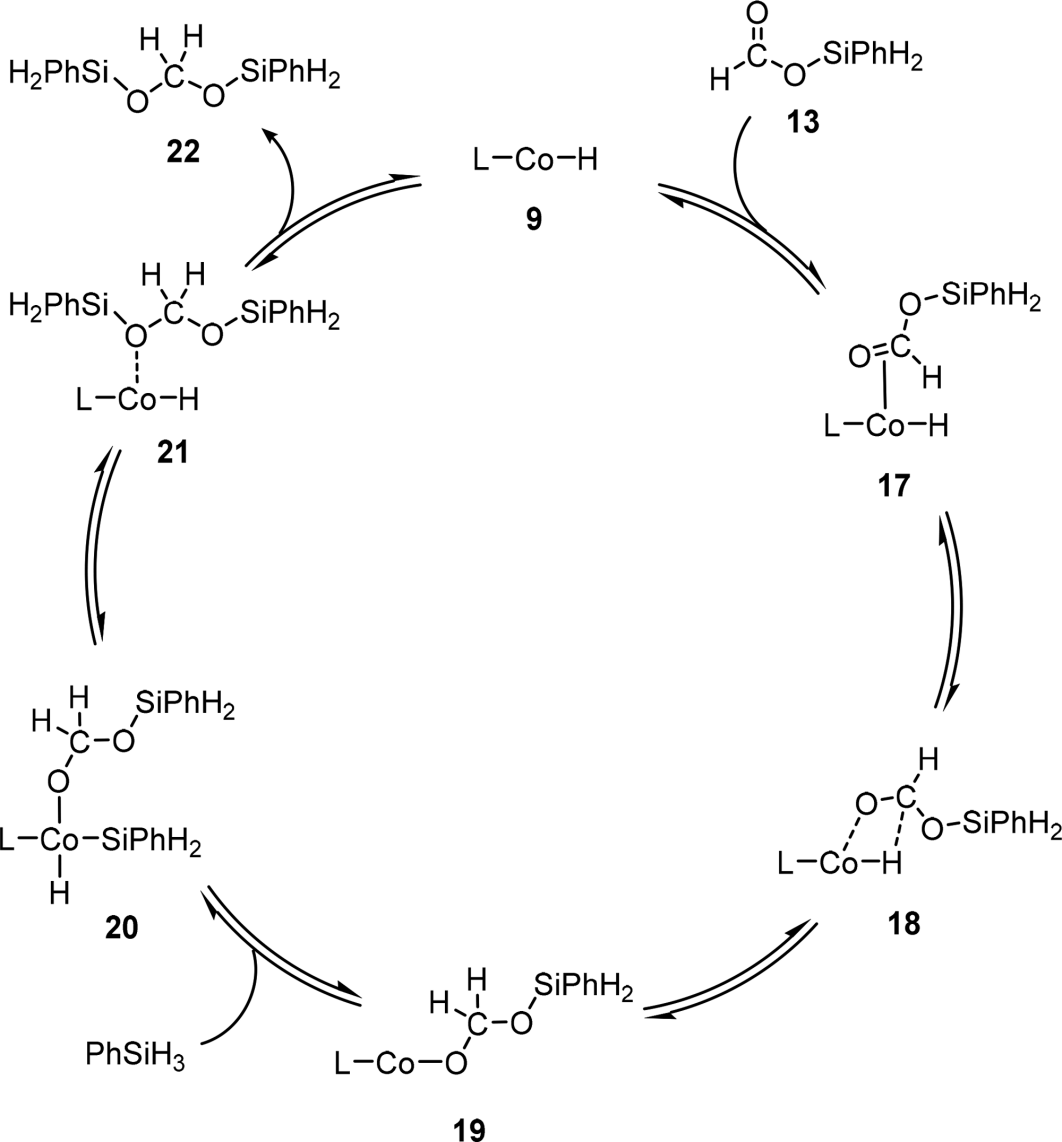
Catalytic Cycle of the Hydrosilylation of Silyl Formate **13** to Bis(silyl)acetal **22**

**Figure 8:**
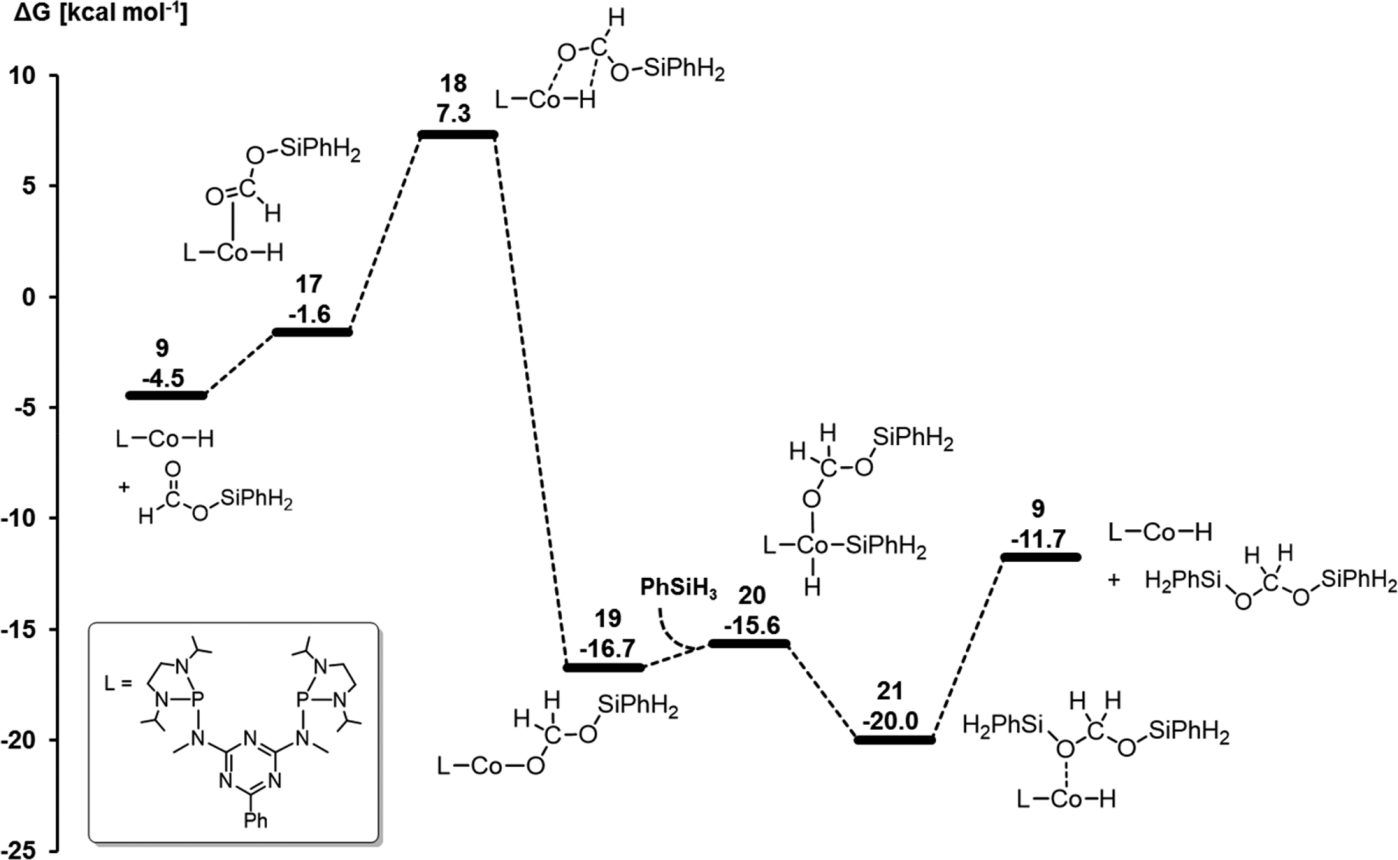
Relative Gibbs free energies [kcal mol^–1^] (B3LYP-D3BJ/def2-TZVP (selected atoms), def-SVP) for the hydrosilylation of silyl formate catalyzed by **9**.

To further reduce the silyl formate to the formaldehyde level, we considered insertion into the Co–H bond of complex **9** as a starting point for the catalytic cycle (Scheme 7, Figure 8). Initially, this leads back to **17** where the silyl formate is coordinated *via* the C–O bond and forms a π-complex. The forward reaction under the formation of the η^2^-complex **18** leads to the second two-electron reduction of the carbon atom in competition with dissociation of silyl formate **13** from **17** to regenerate **9**. Finalizing the insertion step to form the C–H and Co–O bonds leads to **19** in a strongly exothermic process. Oxidative addition of phenylsilane results in the formation of **20**, followed by extrusion to reform the Co–H unit in **21** that constitutes the lowest energy structure at −20.0 kcal mol^–1^ relative to the starting point of the overall sequence. Finally, product release generates **9** and bis(silyl)formate **22**. The four-electron reduction of CO_2_ to bis(silyl)formate **22** has a Gibbs free energy of −11.7 kcal mol^–1^ and provides the driving force for the catalytic turnover. However, the second reduction step is hindered by a higher kinetic barrier than the first two-electron reduction step (Figure 6), as 7.3 kcal mol^–1^ destabilizes **18** compared to **9**. Thus, the high barrier that involves **18** hampers the bis(silyl)acetal formation at lower temperatures. Although **18** was characterized as a local minimum by frequency calculations, it is a high-lying structure on the potential energy surface. Surface scans indicate a rather smooth energy surface, which hampered localization of the transition state connecting **17** and **18**. Structure **18** thus provides a reasonable point of reference for the estimation of the energy span. This way, with the overall lowest intermediate **14** as TDI, an energy span of 28.9 kcal mol^–1^ can be estimated using eq 2.

### Cycle 3: Catalytic Formation of Methoxysilane

**Scheme 8:**
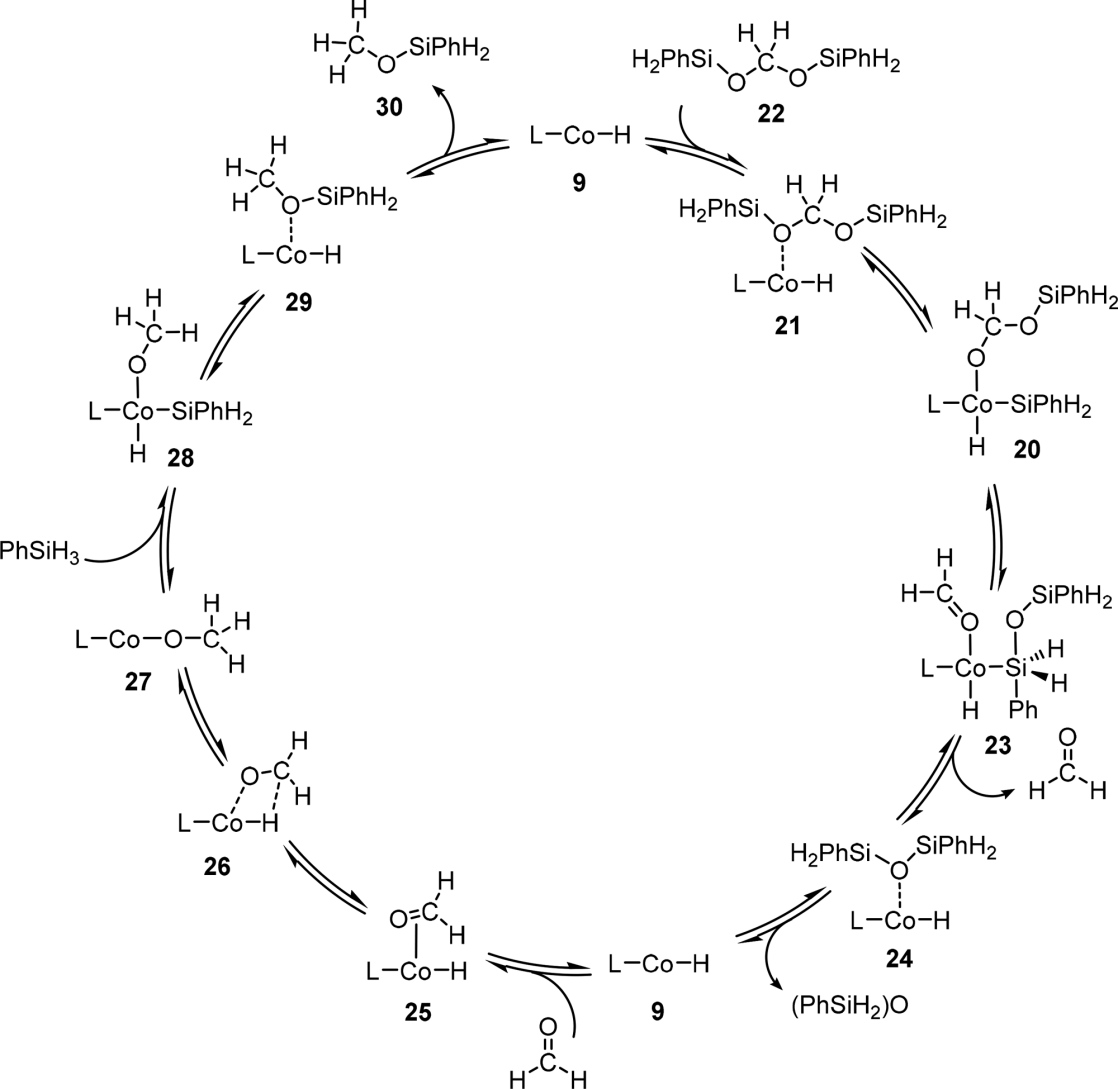
Catalytic Cycle of the Hydrosilylation of Bis(silyl)acetal **22** to the Methoxysilane **30**

As in the previous cycles, the reduction of bis(silyl)acetal **22** can be resumed with the Co–H complex **9** as the active species (Scheme 8). Coordination of the acetal **22** leads back to **20**. Ligand rearrangement within **20** leads to **23**, which bears 1,3-diphenyldisiloxane and formaldehyde in its coordination sphere. This results in a change of the cobalt oxidation state from +III to +I. Geometry scans indicated a concerted formation of the Si–O bond and cleavage of the C–O bond, while no geometry corresponding to an energy maximum was visible, indicating a smooth PES and a transition state close to **23**. With a pentacoordinated silicon atom, **23** is the highest lying structure of the catalytic cycle and has a relative Gibbs free energy of 8.3 kcal mol^–1^. Similar to **18**, the high-lying intermediate **23** was used as a proxy for the transition state that could not be located due to the smooth energy surface. The high energy rapidly relaxes by dissociation of formaldehyde to **24** as a strongly exothermic step, followed by dissociation of the siloxane resulting in the regeneration of **9**. The generation of formaldehyde as an organic intermediate was also reported for the hydrosilylation of CO_2_ with the manganese complex **1**(40) and proposed based on computational studies for the hydroboration of CO_2_ to a methanol derivative catalyzed by a nickel pincer complex.^60^

**Figure 9:**
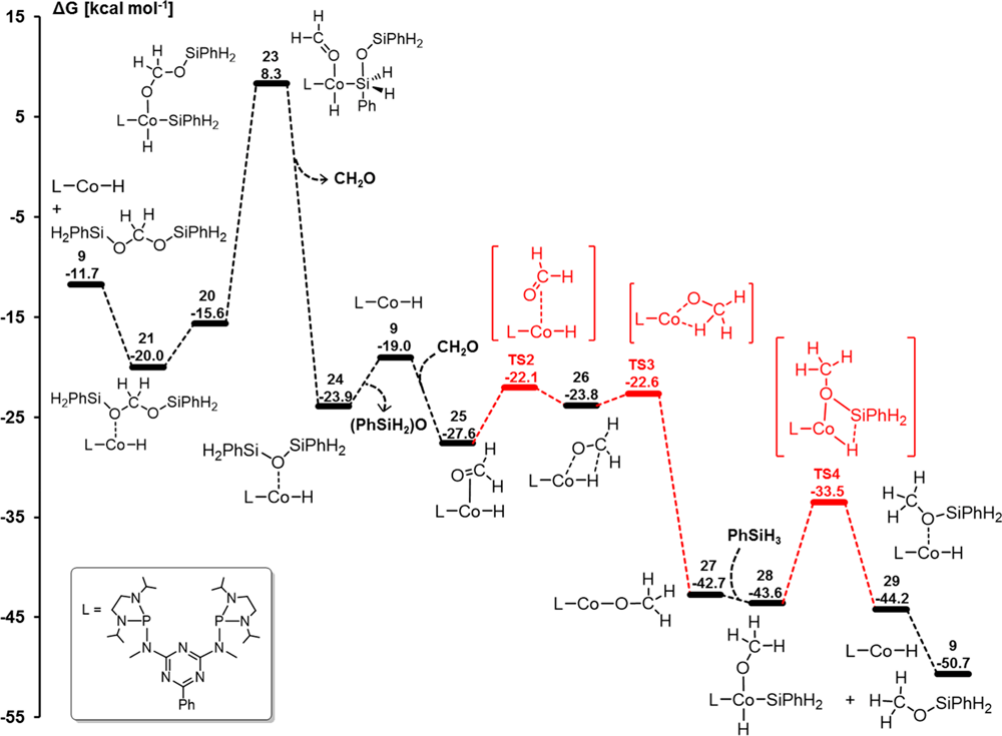
Relative Gibbs free energies [kcal mol^–1^] (B3LYP-D3BJ/def2-TZVP (selected atoms), def-SVP) for the hydrosilylation of formaldehyde catalyzed by **9**.

The hydrosilylation of formaldehyde to methoxysilane **30** involves a series of insertion, oxidative addition, and reductive elimination similar to the two previous reduction steps (Figure 9). Notably, the coordination of formaldehyde to **9** resulting in **25** occurs as an exergonic process and thus is more facile than the coordination of CO_2_ or silyl formate **13** to **9**. The hydride transfer affecting the third two-electron reduction to **26** and the rearrangement to the alkoxy complex **27** proceeds by the transition states **TS2** and **TS3** with moderate activation barriers, while the reaction to **27** has a strong thermodynamic driving force of −31 kcal mol^–1^ relative to the starting point of this cycle. The reductive elimination of the methoxysilane from **28** to **29** involves the transition state **TS4**. The third reduction level provides the strongest thermodynamic driving force of −50.7 kcal mol^–1^. However, due to the high energy of **23**, the third reduction level is associated with a high energy span of ca. 29.9 kcal mol^–1^ (eq 2).

### Selectivity Control in the Reaction Cascade of Cycles 1–3

**Figure 10:**
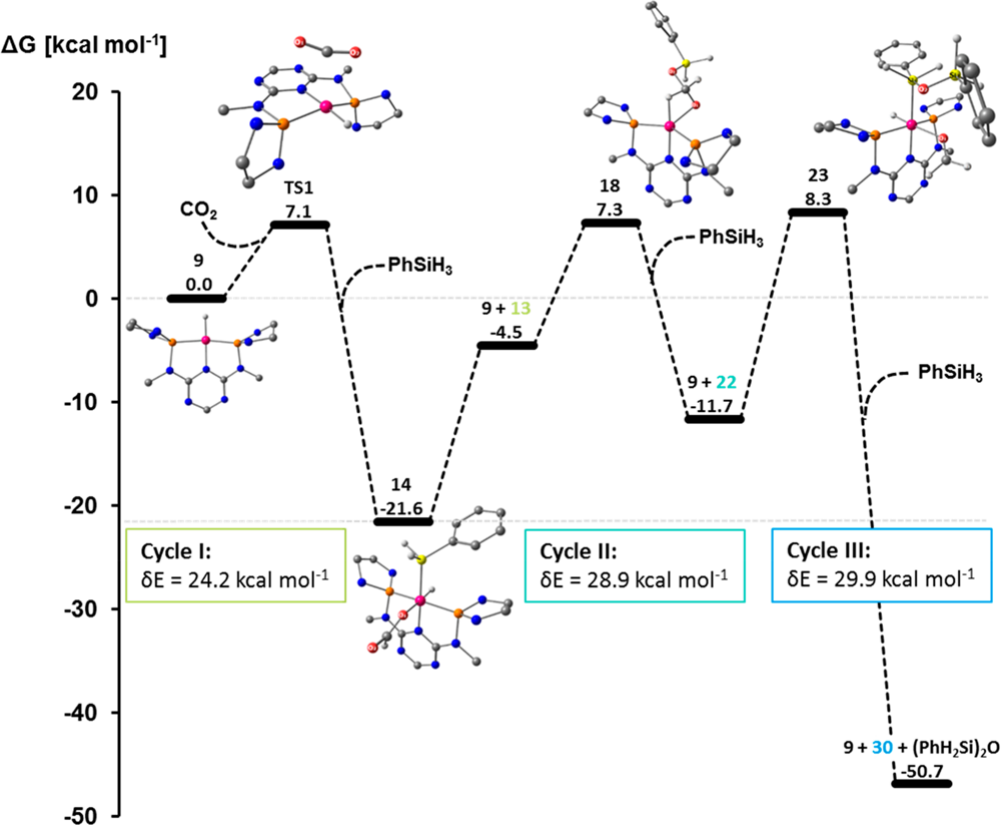
Relative Gibbs free energies [kcal mol^–1^] and energy spans for the key intermediates in the catalytic hydrosilylation of CO_2_ to silyl formate **13**, bis(silyl)acetal **22**, and methoxysilane **30** based on the Co–H complex **9** as the active species.

The energy landscape for the consecutive catalytic CO_2_ hydrosilylation to the formate, formaldehyde, and methanol level is composed of a sequence of the three catalytic cycles discussed above, as shown in Figure 10. The stepwise 2e^–^ reductions result from three mechanistically distinct hydride transfer steps that are all mediated by the same Co–H intermediate **9**. The resulting energy profile summarized in Figure 10 enables us to rationalize the selectivity control in a qualitative and semiquantitative way.

The relative values of the energy spans δ*E*(formate) ≪ δ*E*(formaldehyde) < δ*E*(methoxide) reflect that silyl formate can be obtained very selectively at mild temperatures. While higher temperatures allow for further reduction to bis(silyl)acetal, over-reduction to methoxide is more difficult to avoid due to the second and third cycle’s very similar energy spans. The high kinetic hindrance associated with the rearrangement of bis(silyl)acetal **22** to formaldehyde and 1,3-diphenylsiloxane makes the isolation of the silylated formaldehyde derivatives possible, while the subsequent reduction of formaldehyde to methoxide **30** is a very fast process.

The possibility to control the selectivity by the CO_2_ pressure results from the competing insertion processes of either CO_2_ (**9** to **11**) or silyl formate (**9** to **19**) into the Co–H bond. Higher CO_2_ pressure makes the CO_2_ insertion the dominant process and hinders the silyl formate insertion. Thus, it prevents the forward reaction after the first reduction, making the formate species the strongly preferred product. In contrast, reducing the solvent amount increases the concentration of silyl formate, while the chemical potential of CO_2_ is dependent on the partial pressure and thus remains constant. This agrees with the observation that the reduction beyond the formate level is favored under neat conditions without solvent. Similar considerations about the influence of the CO_2_ pressure and the reactant concentration on the selectivity in the hydrosilylation^41^ and hydroboration^43^ of CO_2_ have been discussed previously.

**Figure 11:**
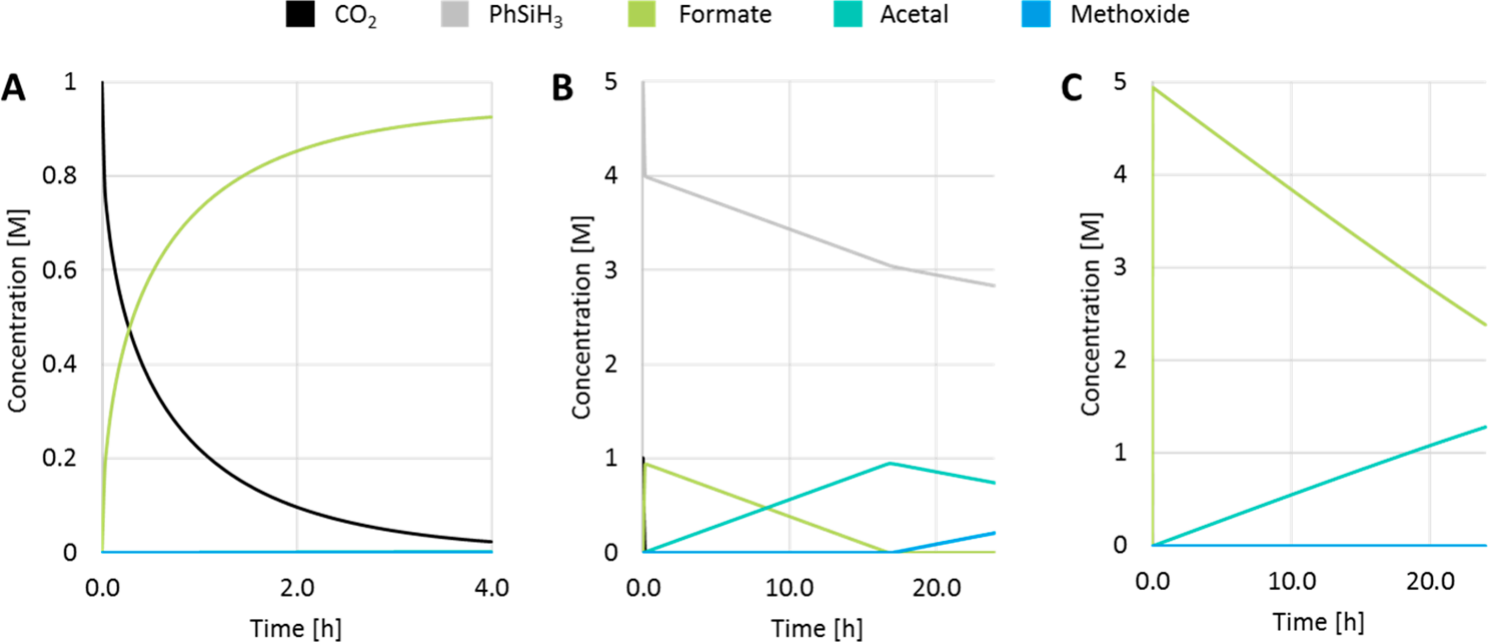
Simulated time profiles of the hydrosilylation of CO_2_ with 1 mol % catalyst **9**. (A) *T* = 80 °C, 1 bar, 4 h; (B) *T* = 120 °C, 1 bar, 24 h; (C) *T* = 120 °C, 20 bar, 24 h.

**Figure 12:**
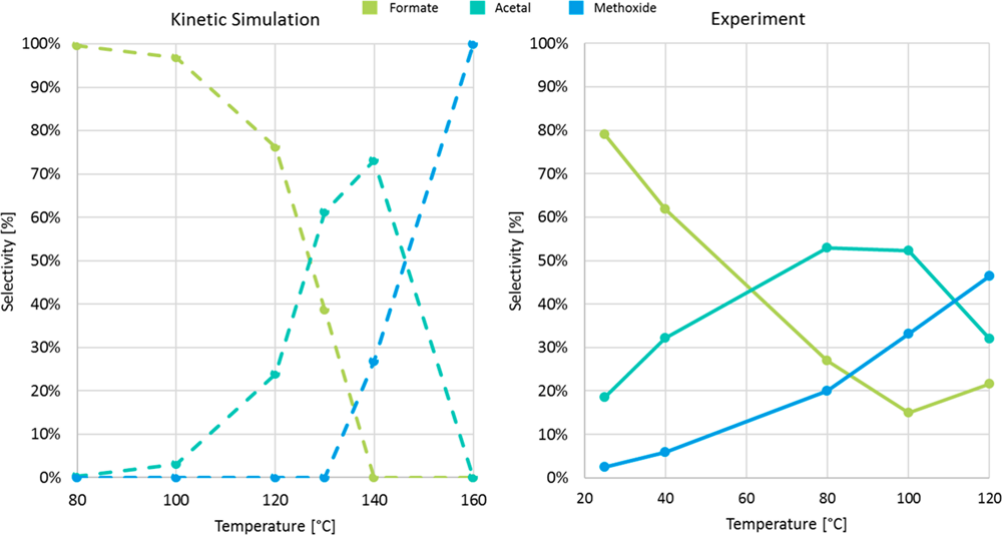
Product distribution of the hydrosilylation of CO_2_ catalyzed by **9**. Simulation (left): **9** (1 mol %), benzene solution, 1 bar, 4 h; Experiment^44^ (right): **5** (0.2 mol %), KO^*t*^Bu (0.8 mol %), neat conditions, 1 bar, 4 h.

To validate the qualitative conclusions, the calculated Gibbs free energy profiles for the three reduction steps were used to perform kinetic simulations of the product distributions (Figures 11 and 12). Bearing in mind the strong sensitivity of the rate constants on small variations of the Gibbs free energies, the relative distributions are considered rather than absolute values. Simulation of a reaction at 80 °C after 4 h results in nearly quantitative conversion of CO_2_ (98%) as observed in the experiment (97%). The absolute values of the simulated product distribution silyl formate ≫ bis(silyl)acetal > methoxysilane corroborate better with the experimental data obtained at lower temperatures indicating that the calculated energy spans are slightly overestimated (Figure 11, panel A). The simulation reflects the further consumption of PhSiH_3_ correctly at higher temperatures and extended reaction times whereby the bis(silyl)acetal and methoxysilane yields are increased consecutively (Figure 11, panel B). Simulating an increase of the CO_2_ pressure to 20 bar at the same temperature results in a higher yield of the silyl formate and shows a lower rate of further reduction (Figure 11, panel C). Therefore, the pronounced influence of variations in temperature and pressure on the product distribution is reproduced correctly in the simulations based on the relative energy profiles of the three consecutive catalytic cycles.

The temperature regimes favoring the selectivity for the individual products as obtained from the kinetic simulations are depicted in Figure 12 (left). The reaction conditions (solvent, catalyst concentration, pressure, reaction time) were chosen close to those used in the experiments (Figure 12, right).^44^ The absolute temperatures associated with high selectivity for each product are higher than in the experiment. For example, while maximum selectivity for the formaldehyde level (53 and 52%) was observed at 80 and 100 °C,^44^ the simulation shows a maximum selectivity of 73% at 140 °C. Still, the temperature windows and the selectivities obtained in the experiments are reproduced very well. It can be seen that high selectivity for the silyl formate can be obtained readily at milder temperatures, and nearly full conversion to methoxide can be achieved at optimized conditions. The intermediate formaldehyde level cannot be reached with the quantitative conversion of the formate and full suppression of the over reduction to methoxide simultaneously, however. The maximum selectivities of the three product levels obtained from the simulations compare remarkably well with the highest selectivities obtained after careful optimization experimentally.

## Conclusion

In conclusion, the present combined experimental and computational study rationalizes the selective reduction of carbon dioxide to silylated formic acid, formaldehyde, and methanol derivatives catalyzed by cobalt triazine complex **5**. The experimentally observed control factors are reflected directly in the energy profiles of the individual cycles and their connection to the full energy surface of the reaction network. The increasing energy spans δ*E*(silyl formate) ≪ δ*E*(bis(silyl)acetal) < δ*E*(methoxysilane) rationalize that higher temperature is required for reduction to formaldehyde and methanol derivatives. High CO_2_ pressures lock in the reaction on the formic acid level as the rate is defined by the competing hydride transfer to either CO_2_ or silyl formate mediated by the active species **9**. However, high concentrations of catalyst and reductants favor the forward reactions leading to formaldehyde or methanol derivatives.

The turnover determining transition state for the reduction beyond the formaldehyde level is not associated with the C=O reduction but with the cleavage of the corresponding silyl acetal (**23**). Most importantly, the high kinetic barrier for the acetal cleavage is identified as the crucial enabler to achieve synthetically relevant selectivities for the formaldehyde level, because the barrier of hydride attack at free H_2_C=O is small, leading to the thermodynamically favored over-reduction. Thus, utilization of the competing hydride transfer to either CO_2_ or silyl formate enables selectivity control in the two- or four-electron reduction of CO_2_, while differentiating the energy spans for the formaldehyde and the methanol level *via* the acetal cleavage rather than the hydride transfer seems to open promising strategies for developing effective catalytic systems that target the four- compared to the six-electron reduction of CO_2_.

In general, the present study highlights the potential of mapping out the complex reaction networks of catalytic CO_2_ reduction by experimental and computational techniques. Identifying the relative influence of the reaction parameters on the catalytic mechanism may contribute to parameter screening and experiment planning in the design of catalytic systems targeting challenging products.^65^ Obviously, general applicable mechanistic principles have to be available for such approaches, and the present study is hoped to provide a valuable contribution in this more general context.

## Supporting Information Available

General considerations, experimental methods and synthetic details, copies of NMR spectra, computational data (PDF)
