# Supplementary material for: Cobalt-Catalyzed Hydrosilylation of Carbon Dioxide to the Formic Acid, Formaldehyde, and Methanol Level—How to Control the Catalytic Network?
Source: JACS Au. 2021 Oct 4;1(11):2058–69. doi: 10.1021/jacsau.1c00350 (PMC8620560; doi:10.1021/jacsau.1c00350)
Supplement: Supplementary file 1 — au1c00350_si_001.pdf [file au1c00350_si_001.pdf]

Supporting Information

Cobalt-Catalyzed Hydrosilylation of Carbon Dioxide to the Formic Acid, Formaldehyde, and Methanol Level— How to Control the Catalytic Network?

Hanna H. Cramer,[1,2] Shengfa Ye,[3,4] Frank Neese,[4] Christophe Werlé,[1,5,*] and Walter Leitner[1,2,]

[1] Max Planck Institute for Chemical Energy Conversion, Stiftstr. 34 – 36, 45470 Mülheim an der Ruhr, Germany.
[2] Institut für Technische und Makromolekulare Chemie (ITMC), RWTH Aachen University, Worringer Weg 2, 52074 Aachen, Germany.
[3] State Key Laboratory of Catalysis, Dalian Institute of Chemical Physics, Chinese Academy of Sciences, Dalian 116023, China.
[4] Max-Planck-Institut für Kohlenforschung, Kaiser-Wilhelm-Platz 1, D-45470 Mülheim an der Ruhr, Germany.
[5] Ruhr University Bochum, Universitätsstr. 150, 44801 Bochum, Germany.
* Emails: christophe.werle@cec.mpg.de; walter.leitner@cec.mpg.de

Table of Contents

1. General Considerations S3
2. Experimental Procedures S4
   2.1 Preparations S4
      2.1.1 [Co(^{NMe}PNP)H] **9** S4
      2.1.2 [Co(^{NMe}PNP)($SiPhH_2$)H_2] **10** S7
      2.1.3 [Co(^{NMe}PNP)OCHO] **11** S11
      2.1.4 [Co(^{NMe}PNP)(SiHPh(OCHO))H_2] **12** S16
   2.2 Catalytic Hydrosilylation of Carbon Dioxide S19
      2.2.1 Catalytic activity of **9** S19
      2.2.2 Catalytic activity of **10** and **11** S22
   2.3 Control Experiments S24
      2.3.1 Reaction of **10** with CO_2 .... S24
      2.3.2 Reaction of **11** with xs. $PhSiH_3$ S27
      2.3.3 Preparation of **10** from **5**, KO^{t}Bu and $PhSiH_3$ S29
      2.3.4 Reaction of **5** and KO^{t}Bu S30
      2.3.5 NMR analysis of the reaction mixture containing **9**, $PhSiH_3$, and CO_2 under catalytic conditions S31
3. Computational Details S32
4. Orbital Localization Output S38
5. Off-cycle intermediates and alternative isomers S41
6. Multiple Si-H activations S42
7. Reductive elimination of formic acid S43
8. Kinetic Simulations S44
9. XYZ Coordinates of Optimized Geometries S45
10. Literature S123

1. General Considerations

All air and moisture-sensitive experiments were conducted under dry argon atmosphere using standard Schlenk techniques or an MBraun inert-gas glovebox containing an atmosphere of purified argon. Solvents for air- and moisture-sensitive experiments were purified using a two-column solvent purification system (MBraun-SPS-7) and transferred to the glovebox without exposure to air. Deuterated solvents were degassed and stored over activated molecular sieves prior to use. All chemicals were purchased from Sigma-Aldrich, TCI and ABCR chemicals and used without further purification. [Co(^{NMe}PNP)Cl_2] **5** was synthesized according to literature procedures.[1] NMR spectra were recorded on Bruker AV-400 and Bruker AV-500 spectrometers at the indicated temperatures with the chemical shifts (δ) given in ppm relative to TMS and the coupling constants (*J*) in Hz. The solvent signals were used as references and the chemical shifts converted to the TMS scale (C_6D_6: δ^1H = 7.16 ppm, δ^{13}C = 128.1 ppm; toluene-d_8: δ^1H = 2.09, 6.98, 7.00, 7.09, δ^{13}C = 125.5, 128.3, 129.2, 137.9). For quantification, the NMR samples were measured with a 500 MHz Bruker AVANCE III HD NMR spectrometer equipped with a Prodigy Cryo-Probe. ^{13}C spectra (125.74 MHz) were recorded using a 90° pulse with 1H *inverse gated* decoupling (pulse program zgig). 256 scans were accumulated using a recycling delay of 60 seconds to allow for complete relaxation of the ^{13}C nuclei. Spectra were thoroughly phased and integrated to provide quantitative results as checked by the internal reference. CHN elemental microanalyses were measured at "Mikroanalytisches Labor Kolbe" (c/o Fraunhofer Institut UMSICHT). HR-MS spectra were recorded on a Bruker ESQ3000 spectrometer. Infrared spectra were recorded on a Thermo Scientific Nicolet™ iS5 Spectrometer with an ID7 ATR accessory.

2. Experimental Procedures

2.1 Preparations

2.1.1 [Co(^{NMe}PNP)H] 9

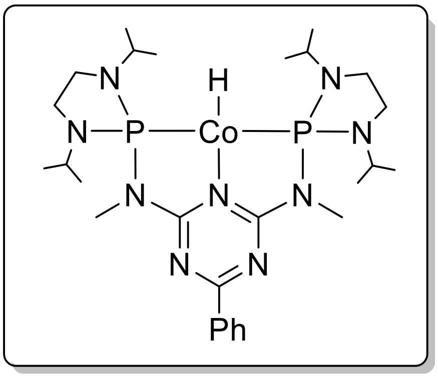

A solution of NaBEt$_3$H (91 µL, 0.0913 mmol, 1M in toluene) was added to a solution of [Co(^{NMe}PNP)Cl$_2$] **5** (30 mg, 0.0435 mmol) in 3 mL toluene. After stirring for 20 min at room temperature, the mixture was filtrated through a syringe filter, and the volatiles were removed in vacuo. The product mixture was obtained as a dark green solid and was characterized without further purification to prevent decomposition.

^{1}H NMR (500 MHz, Toluene-d$_8$, 298 K): δ –13.31 (t, $^3J_{P,H}$ = 56 Hz, 1H, Co–***H***), 0.98 (d, $^3J_{H,H}$ = 6.5 Hz, 12 H, ^{i}Pr–C***H***$_3$), 1.21 (d, $^3J_{HH}$ = 6.5 Hz, 12 H, ^{i}Pr–C***H***$_3$), 2.89–2.94 (m, 4 H, C***H***$_2$), 3.00 (s, 6 H, NC***H***$_3$), 3.02–3.07 (m, 4 H, C***H***$_2$), 4.56 (sept, $^3J_{H,H}$ = 6.5 Hz, 4 H, ^{i}Pr–C***H***), 7.19–7.30 (m, 3 H, Ph-*p*–***H***, Ph-*m*–***H***), 8.72 (d, $^3J_{H,H}$ = 7.4 Hz, 2 H, Ph-*o*–***H***).

^{13}C{^{1}H} NMR (126 MHz, Toluene-d$_8$, 298 K): δ 19.7 (CH$_3$, ^{i}Pr–***C***H$_3$), 20.9 (CH$_3$, ^{i}Pr–***C***H$_3$), 28.9 (CH$_3$, N***C***H$_3$), 39.9 (***C***H$_2$), 44.2 (m, CH, ^{i}Pr–***C***H), 128.2 (CH, Ph-*m*–***C***H), 128.9 (CH, Ph-*o*–***C***H), 131.0 (CH, Ph-*p*–***C***H), 138.2 (Ph-*i*–***C***), 165.9 (m, Triazine-*o*–***C***), 166.2 (Triazine-*p*–***C***).

^{31}P{^{1}H} NMR (202 MHz, Toluene-d$_8$, 298 K): δ 178.1.

HRMS (ESI$^+$): calcd. for [C$_{27}$H$_{49}$N$_9$P$_2$Co]$^+$: 620.29127; found: 620.29102

IR (Diamond ATR cell, cm^{-1}): 2960, 2926, 2853, 1746, 1588, 1538, 1520, 1463, 1979, 1359, 1260, 1223, 1173, 1086, 1047, 1027, 967, 910, 860, 801, 775, 764, 743, 699, 609, 585, 520, 507, 491, 485, 478, 466, 453, 441.

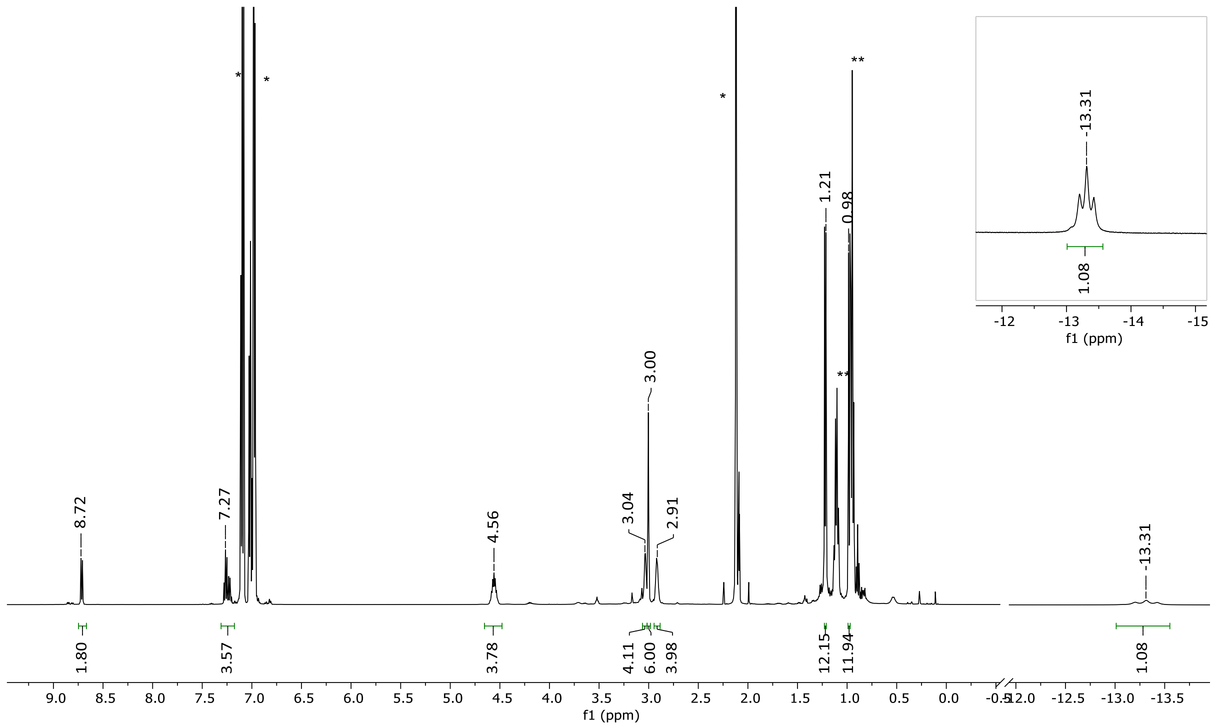

Figure S1: ¹H NMR spectrum (500 MHz, toluene-d$_8$, 298 K) of **9** (* = Toluene, ** = BEt_3).

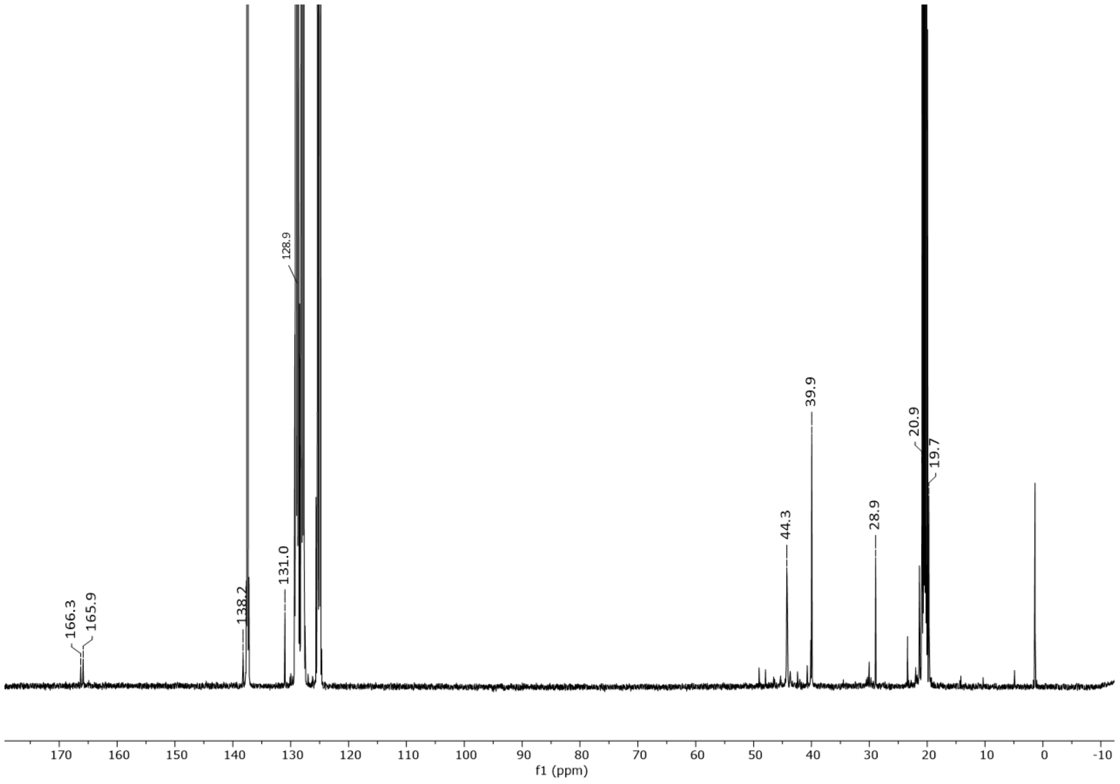

Figure S2: $^{13}C\{^1H\}$ NMR spectrum (126 MHz, toluene-d$_8$, 298 K) of **9**.

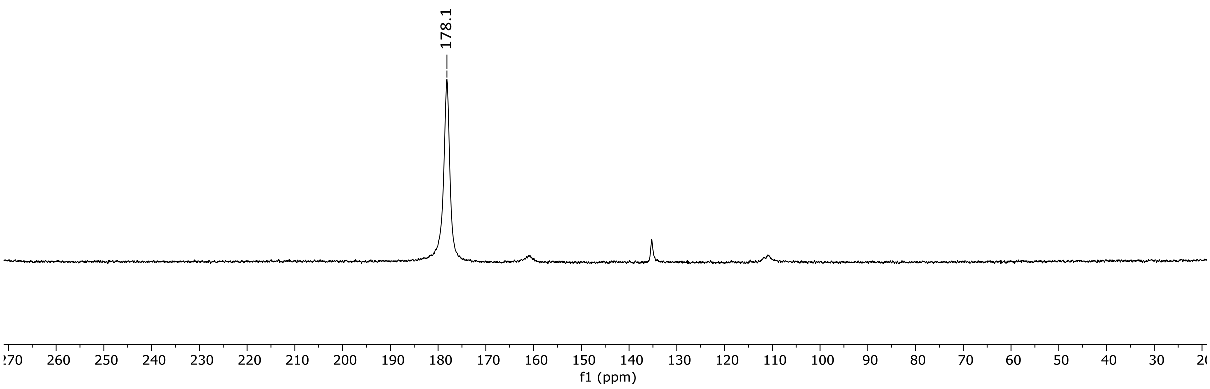

Figure S3: $^{31}P\{^{1}H\}$ NMR spectrum (202 MHz, toluene-d$_8$, 298 K) of **9**.

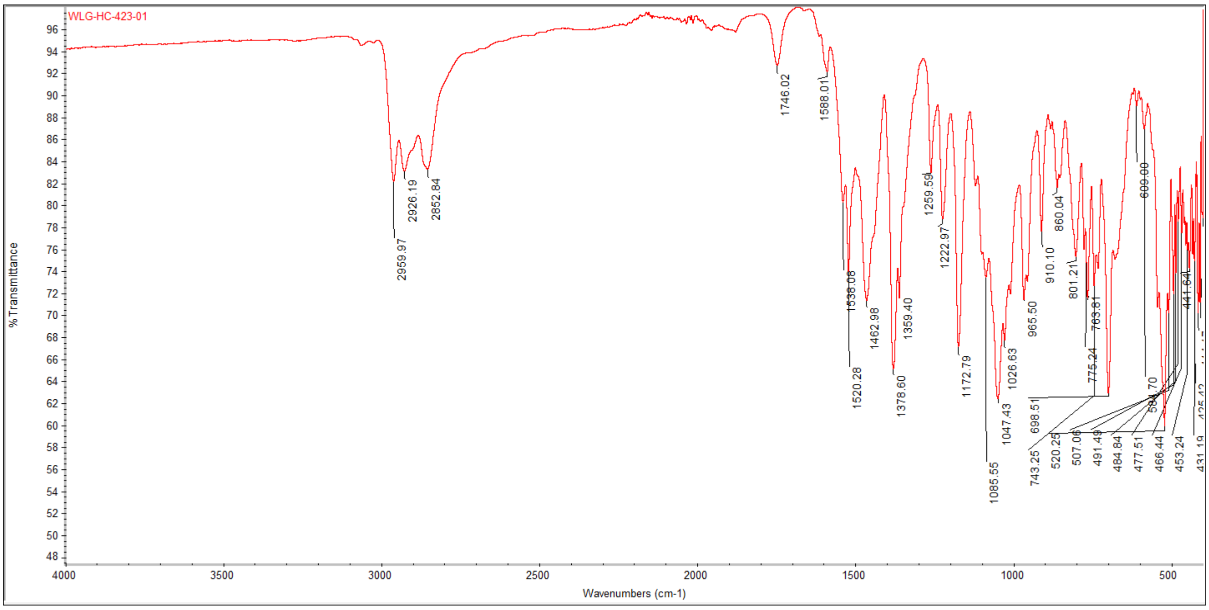

Figure S4: IR spectrum (Diamond ATR cell) of **9**.

2.1.2 [Co(^{NMe}PNP)(SiPhH$_2$)H$_2$] 10

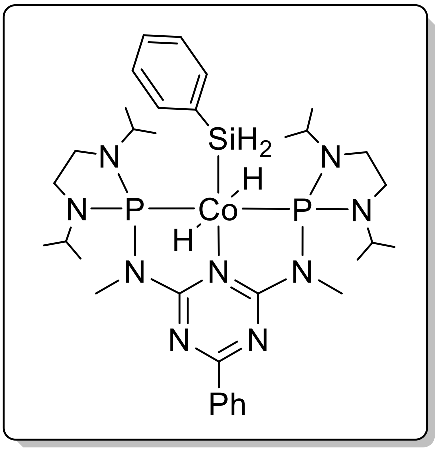

A solution of NaBEt$_3$H (183 µL, 0.183 mmol, 1M in toluene) was added to a solution of [Co(^{NMe}PNP)Cl$_2$] **5** (60 mg, 0.0870 mmol) in toluene (6 mL), and the mixture was stirred for 5 min. Subsequently, phenylsilane (10.7 µL, 0.0870 mmol) was added, and the mixture was stirred at room temperature for 20 min. After filtration, the volatiles were removed in vacuo. The product mixture was obtained as brown solid and was characterized without further purification to prevent decomposition.

^{1}H NMR (400 MHz, C$_6$D$_6$, 298 K): δ –9.28 (t, $^2J_{P,H}$ = 47.6 Hz, 2 H, Co–***H***), 0.89 (d, $^3J_{H,H}$ = 6.7 Hz, 12 H, ^{i}Pr–C***H***$_3$), 1.17 (d, $^3J_{H,H}$ = 6.7 Hz, 12 H, ^{i}Pr–C***H***$_3$), 2.93–2.96 (m, 14 H, NC***H***$_3$, C***H***$_2$), 4.54–4.59 (m, 6 H, ^{i}Pr–C***H***, Si***H***$_2$), 7.20–7.24 (m, 2 H, Ar–C***H***), 7.28–7.32 (m, 4 H, Ar–C***H***), 8.03–8.05 (m, 2 H, Si–Ph–***H***), 8.80–8.81 (m, 2 H, Ar–C***H***).

^{1}H NMR (400 MHz, toluene-d$_8$, 298 K): δ –9.39 (t, $^2J_{P,H}$ = 47.7 Hz, 2 H, Co–***H***), 0.88 (d, $^3J_{H,H}$ = 6.7 Hz, 12 H, ^{i}Pr–C***H***$_3$), 1.12 (d, $^3J_{H,H}$ = 6.7 Hz, 12 H, ^{i}Pr–C***H***$_3$), 2.90–3.03 (m, 14 H, NC***H***$_3$, C***H***$_2$), 4.40 (t, $^3J_{P,H}$ = 10.4 Hz, 2 H, Si***H***$_2$), 4.51 (sept, $^3J_{H,H}$ = 6.6 Hz, 4 H, ^{i}Pr–C***H***,), 7.14–7.31 (m, 6 H, Ar–C***H***), 7.92 (d, $^3J_{H,H}$ = 6.8 Hz, 2 H, Ar–C***H***), 8.72 (d, $^3J_{H,H}$ = 7.2 Hz, 2 H, Ar–C***H***).

^{13}C{^{1}H} NMR (400 MHz, C$_6$D$_6$, 298 K): δ 20.8 (^{i}Pr–***C***H$_3$), 21.2 (m, ^{i}Pr–***C***H$_3$), 30.2 (m, N–***C***H$_3$), 40.5 (***C***H$_2$), 44.1 (m, ^{i}Pr–***C***H), 127.0 (CH, Ar–***C***H), 128.3 (CH, Ar–***C***H), 128.4 (CH, Ar–***C***H), 129.1 (CH, Ar–***C***H), 131.5 (CH, Ar–***C***H), 136.0 (CH, Ar–***C***H), 137.9 (Triazine–Ph-*i*–***C***), 147.3 (SiPh-*i*–***C***), 165.9 (m, Triazine-*o*–***C***), 167.0 (Triazine-*p*–***C***).

^{31}P{^{1}H} NMR (400 MHz, C$_6$D$_6$, 298 K): δ 174.0.

IR (Diamond ATR cell, cm^{-1}): 2961, 2927, 2864, 2025, 1758, 1587, 1544, 1520, 1469, 1425, 1380, 1361, 1260, 1222, 1173, 1093, 1054, 1027, 968, 933, 912, 839, 801, 776, 764, 742, 729, 698, 543, 520, 491, 463, 454, 443, 436.

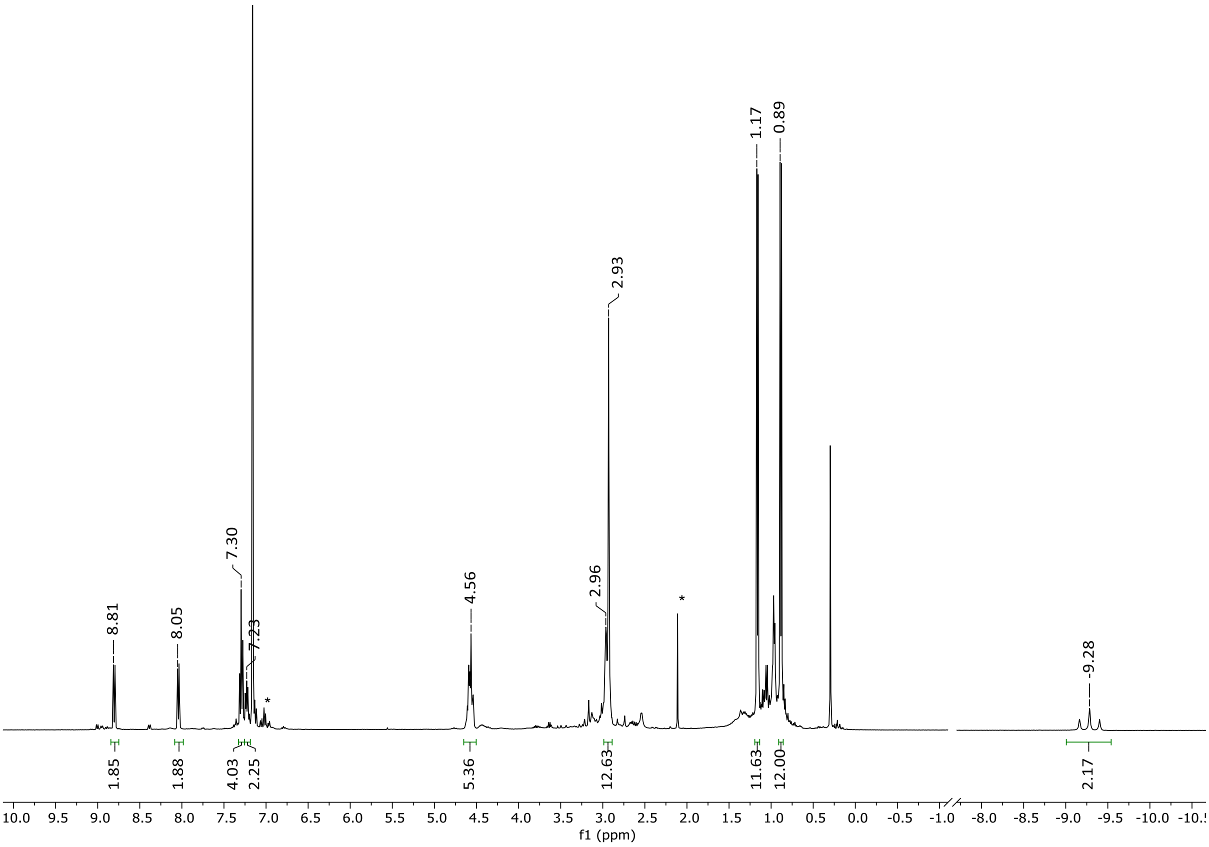

Figure S5: ^{1}H NMR spectrum (400 MHz, C_6D_6, 298 K) of **10** (* = Toluene).

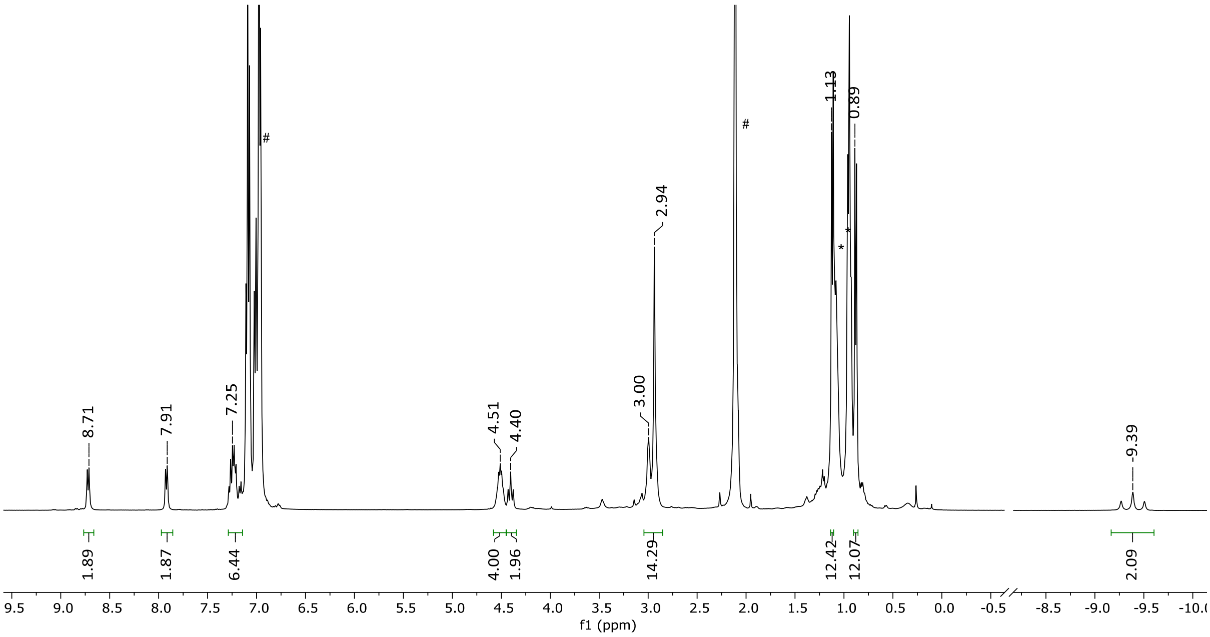

Figure S6: ¹H NMR spectrum (400 MHz, toluene-d₈, 298 K) of **10.** * = BEt_3, # - Toluene.

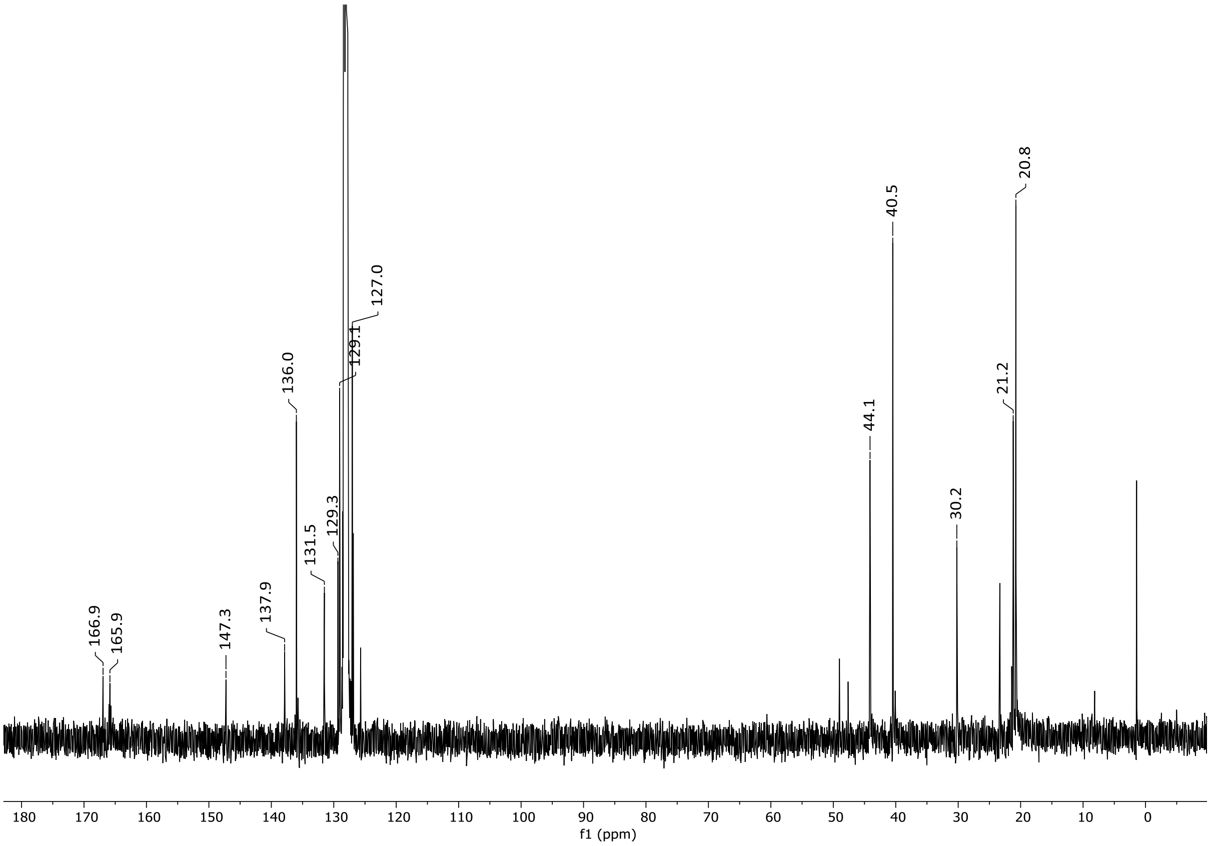

Figure S7: ¹³C{¹H} NMR spectrum (400 MHz, C_6D_6, 298 K) of **10**.

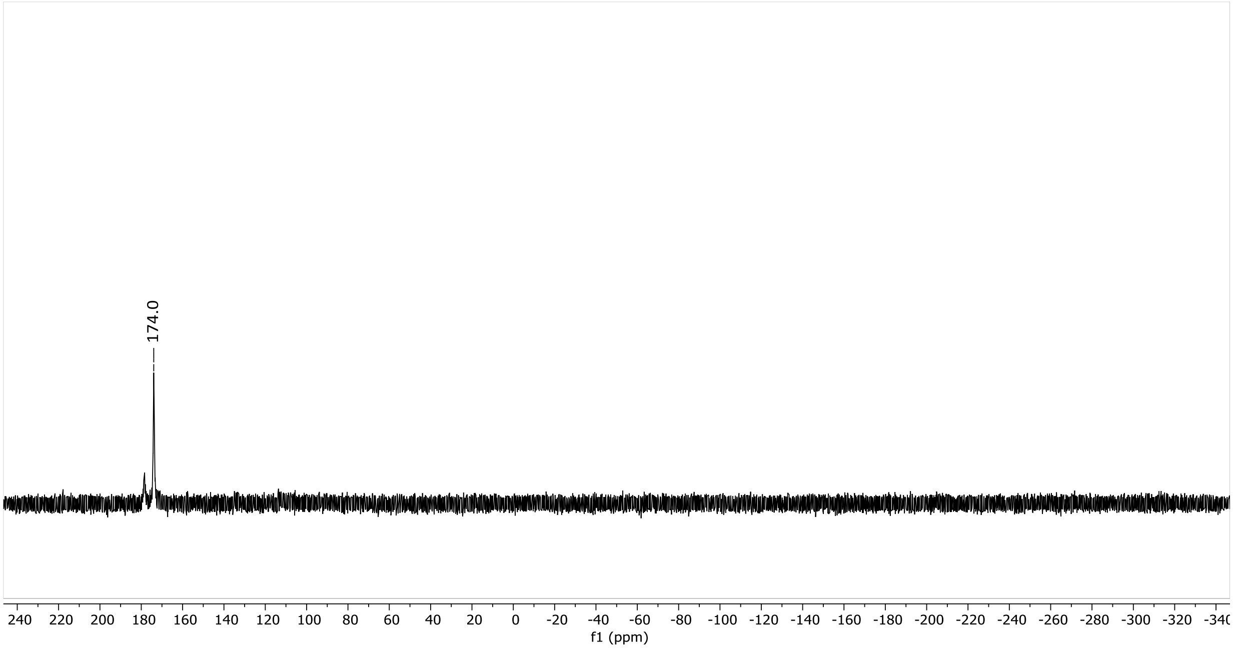

Figure S8: ^{31}P{^{1}H} NMR spectrum (162 MHz, C_6D_6, 298 K) of **10**.

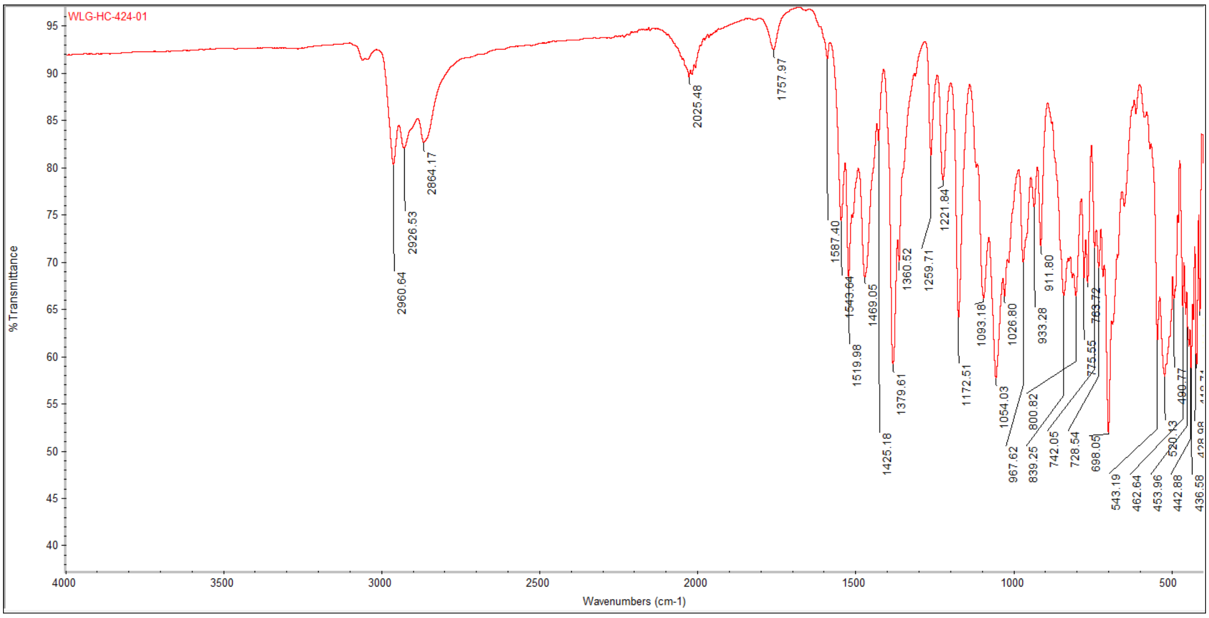

Figure S9: IR spectrum (Diamond ATR cell) of **10**.

2.1.3 [Co(^{NMe}PNP)OCHO] 11

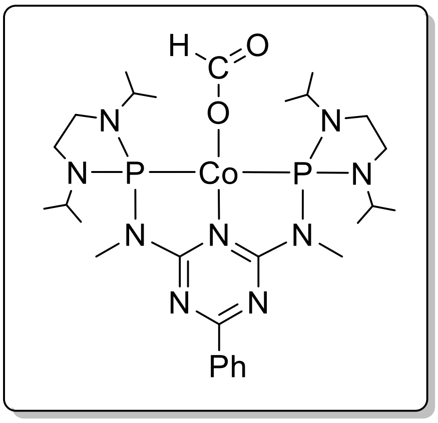

[Co(^{NMe}PNP)H] **9** (15 mg, 0.0242 mmol, 1 eq) was dissolved in 1 mL toluene-d_8 in a Schlenk tube. The reaction mixture was frozen in liquid nitrogen and the headspace evacuated. Subsequently, the reaction mixture was exposed to 1 bar CO_2 and stirred for 20 min while warming to room temperature. After subsequent filtration, the volatiles were removed in vacuo. The product mixture was obtained as a brown solid and was characterized without further purification to prevent decomposition.

^{1}H NMR (500 MHz, Toluene-d$_8$, 298 K): δ 1.24 (d, $^3J_{H,H}$ = 6.5 Hz, 12 H, ^{i}Pr–C***H***$_3$), 1.29 (d, $^3J_{H,H}$ = 6.5 Hz, 12 H, ^{i}Pr–C***H***$_3$), 2.92–2.94 (m, 6 H, NC***H***$_3$), 2.95–3.03 (m, 8 H, C***H***$_2$), 4.08 (sept, $^3J_{H,H}$ = 6.5 Hz, 4 H, ^{i}Pr–C***H***), 7.17 (t, $^3J_{H,H}$ = 7.8 Hz, 2 H, Ph-*m*–***H***), 7.39 (t, $^3J_{H,H}$ = 7.3 Hz, 1 H, Ph-*p*–***H***), 7.83 (s, 1 H, OC***H***O), 8.68 (d, $^3J_{H,H}$ = 7.5 Hz, 2 H, Ph-*o*–***H***).

^{13}C{^{1}H} NMR (126 MHz, Toluene-d$_8$, 298 K): δ 21.8 (CH_3, ^{i}Pr–***C***H$_3$), 22.3 (CH_3, ^{i}Pr–***C***H$_3$), 28.7 (CH_3, N***C***H$_3$), 43.0 (***C***H$_2$), 47.3 (m, CH, ^{i}Pr–***C***H), 127.6 (CH, Ph-*o*–***C***H), 129.0 (CH, Ph-*m*–***C***H), 130.3 (CH, Ph-*p*–***C***H), 139.1 (Ph-*i*–***C***), 157.8 (Triazine-*p*–***C***), 166.4 (m, Triazine-*o*–***C***), 173.9 (m, CH, O***C***HO).

^{31}P{^{1}H} NMR (162 MHz, Toluene-d$_8$, 298 K): δ 111.9.

IR (Diamond ATR cell, cm^{-1}): 2961, 2928, 2855, 1910, 1593, 1548, 1458, 1380, 1259, 1215, 1173, 1121, 1069, 1049, 1026, 1008, 956, 910, 853, 801, 777, 756, 694, 584, 561.

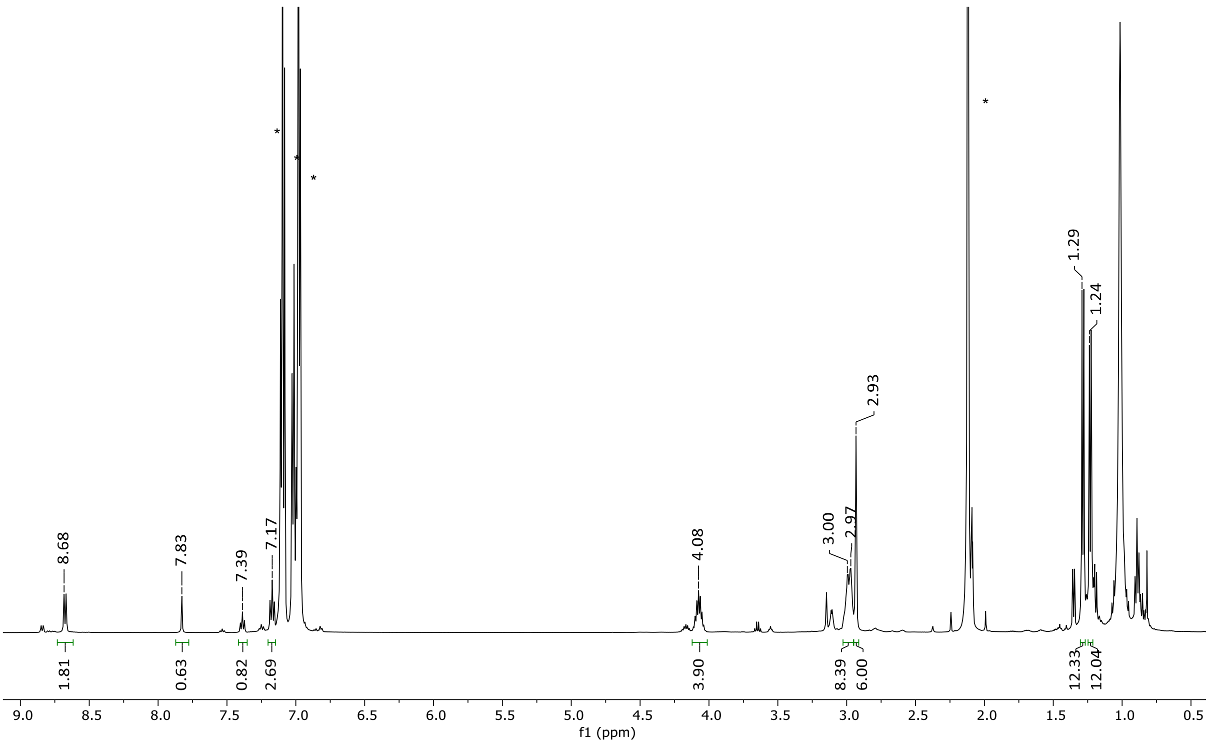

Figure S10: ^{1}H NMR spectrum (126 MHz, toluene-d8, 298 K) of **11** prepared with $^{12}CO_2$ (* = Toluene).

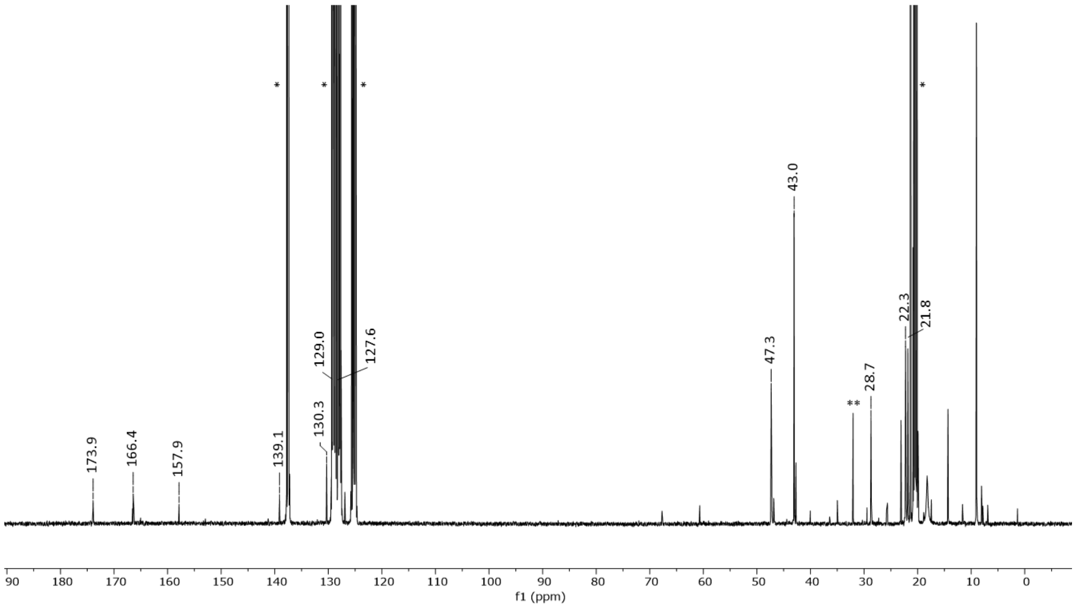

Figure S11: ^{13}C{^{1}H} NMR spectrum (500 MHz, toluene-d8, 298 K) of **11** prepared with $^{12}CO_2$ (* = Toluene, ** - H grease).

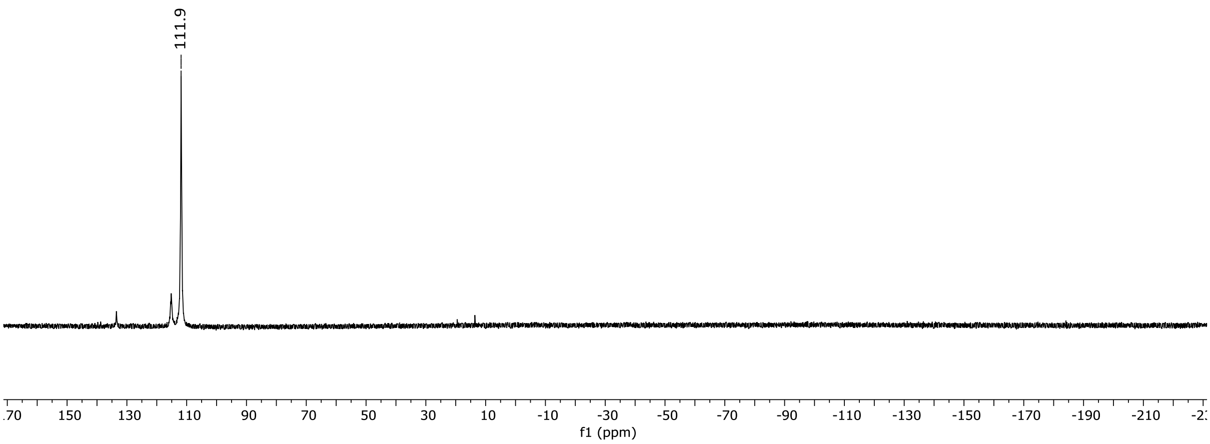

Figure S12: $^{31}P\{^{1}H\}$ NMR spectrum (202 MHz, toluene-d$_8$, 298 K) of **11** prepared with $^{12}CO_2$.

Preparation of the ^{13}C isotopologue [Co(^{NMe}PNP)O^{13}CHO]

A solution of $NaBEt_3H$ (32 µL, 0.0320 mmol, 1M in toluene) was added to a toluene-d$_8$ (1 mL) solution of [Co(^{NMe}PNP)Cl_2] **5** (10 mg, 0.0145 mmol). After stirring for 20 min at room temperature, the mixture was filtrated through a syringe filter and transferred into a J. Young NMR tube. The reaction mixture was frozen in liquid nitrogen and the headspace evacuated. Subsequently, the solution was exposed to $^{13}CO_2$ (1 bar) and warmed to room temperature. The NMR tube was carefully shaken and the mixture analyzed by NMR spectroscopy.

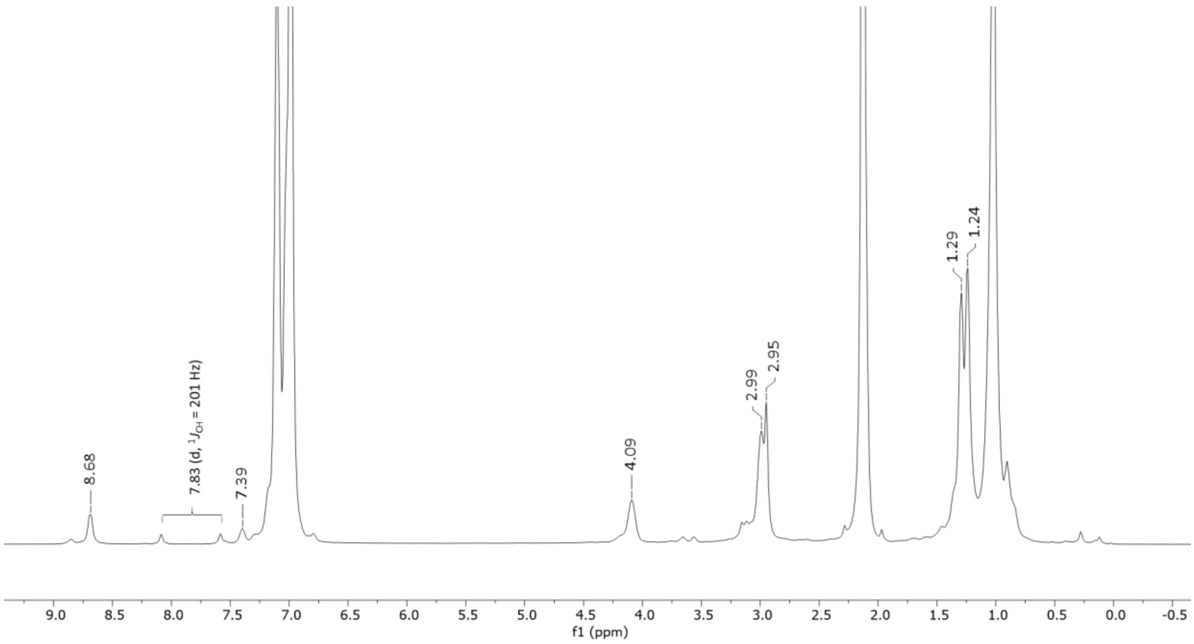

Figure S13: ^{1}H NMR spectrum (400 MHz, toluene-d$_8$, 298 K) of **11** prepared with $^{13}CO_2$.

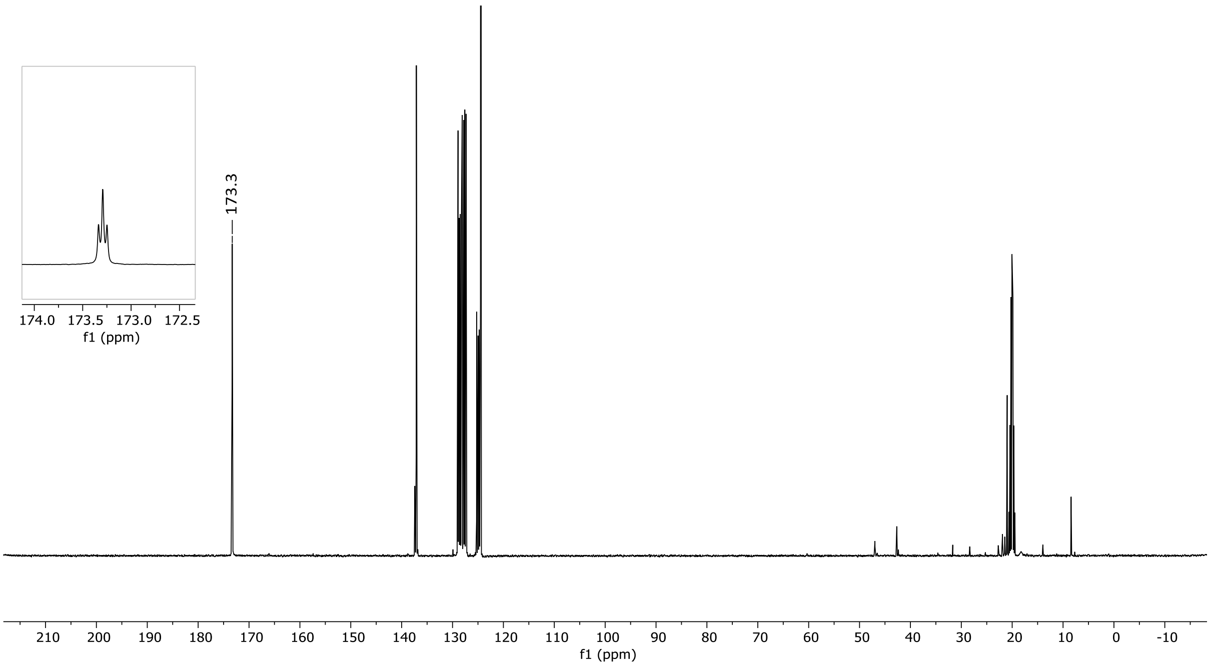

Figure S14: $^{13}C\{^1H\}$ NMR spectrum (101 MHz, toluene-d$_8$, 298 K) of **11** prepared with $^{13}CO_2$.

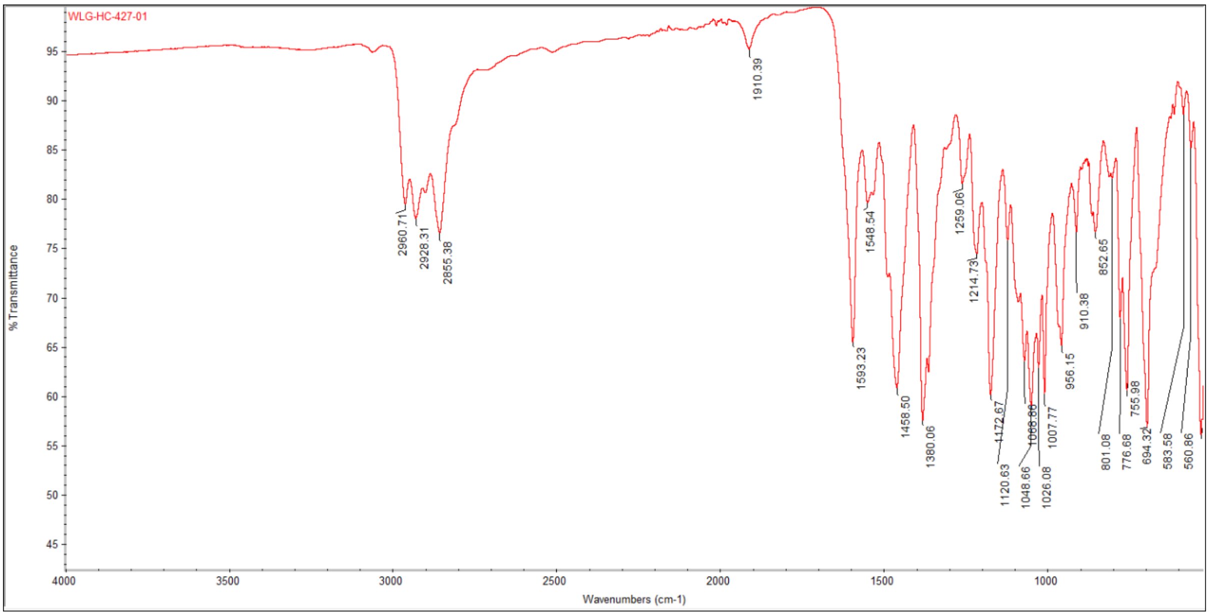

Figure S15: IR spectrum (Diamond ATR cell) of **11**.

2.1.4 [Co(^{NMe}PNP)(SiHPh(OCHO))H$_2$] 12

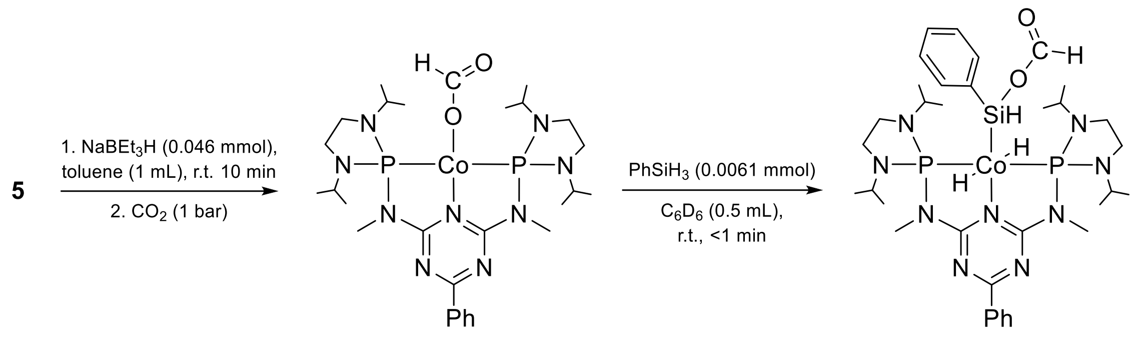

NaBEt$_3$H (46 µL, 0.046 mmol, 1M in toluene) was added to **5** (15 mg, 0.022 mmol) in toluene (1 mL). The mixture was stirred for 10 min at room temperature. Subsequently, the mixture was frozen and the headspace evacuated and exposed to 1 bar $^{13}CO_2$. The reaction mixture was stirred for 15 min while warming to room temperature, followed by removal of the volatiles in vacuum. The residue was dissolved in C_6D_6, the solution was filtered and 1,4-dioxane was added as internal standard to determine the yield of **11** (0.0061 mmol, 28%). Subsequently, phenylsilane (0.8 µL, 0.0061 mmol) was added to the NMR tube. After careful shaking, the solution was analyzed by NMR spectroscopy. Product **12** was present with an NMR yield of 0.0034 mmol (15%).

^{1}H NMR (400 MHz, C_6D_6, 298 K): δ –8.80 (t, $^2J_{P,H}$ = 47.1 Hz, 2 H, Co–***H***), 0.82–0.84 (m, 12 H), 1.04 (d, $^3J_{H,H}$ = 6.7 Hz, 6 H, ^{i}Pr–C***H***$_3$), 1.15 (d, $^3J_{H,H}$ = 6.7 Hz, 6 H, ^{i}Pr–C***H***$_3$), 2.85 (m, NC***H***$_3$, 6 H), 2.90–2.94 (m, 8 H, C***H***$_2$), 4.42–4.51 (m, 4 H, ^{i}Pr–C***H***). 5.79 (td, $^3J_{P,H}$ = 9.0 Hz, $^3J_{C,H}$ = 3.4 Hz, 1 H, Si–***H***), 7.23–7.28 (m, 6H, Ar–C***H***), 7.90 (d, $^3J_{H,H}$ = 7.8 Hz, 2 H, Ar–C***H***), 8.48 (d, $^1J_{C,H}$ = 201.7 Hz, 1 H, OC***H***O), 8.73–8.75 (m, 2 H, Ar–C***H***).

^{31}P{^{1}H} NMR (162 MHz, C_6D_6, 298 K): δ 170.8.

^{13}C{^{1}H} NMR (101 MHz, C_6D_6, 298 K): δ 20.6–21.2 (m, CH$_3$, ^{i}Pr–***C***H$_3$), 30.3 (m, CH$_3$, N***C***H$_3$), 40.3–40.5 (m, ***C***H$_2$), 44.2 (CH, ^{i}Pr–***C***H), 127.0 (Ar–***C***H), 128.4 (Ar–***C***H), 131.5 (Ar–***C***H), 134.5 (Ar–***C***H), 138.2 (Ph–***C***), 146.4 (Ph–***C***), 162.1 (O***C***HO), 165.6 (m, Triazine-*o*–***C***).

Two Ph–***C***H and the Triazine-*p*–C signal could not be properly located.

HRMS (ESI$^+$): calcd. for [^{13}C^{12}C$_{33}$H$_{55}$O$_2$N$_9$P$_2$SiCo]$^+$ = [M–H]$^+$: 771.30833; found: 771.30812.

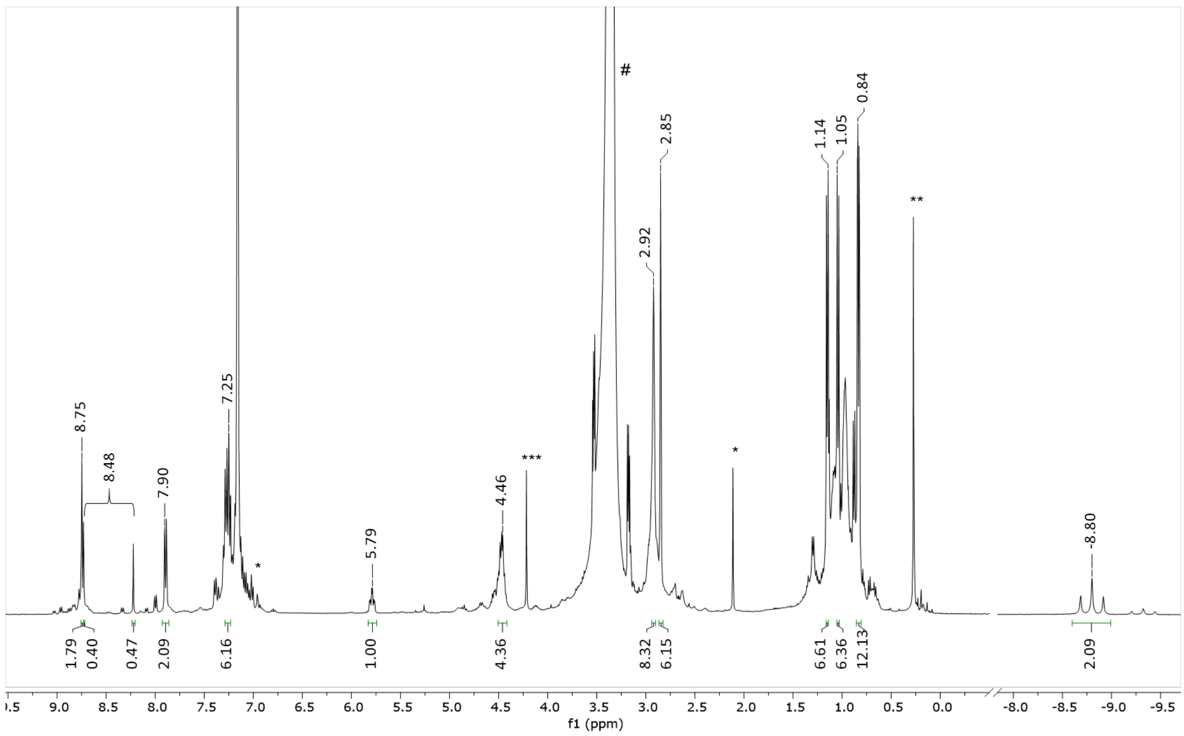

Figure S16: ^{1}H NMR spectrum (400 MHz, C_6D_6, 298 K) of **12** (* Toluene, ** silicone grease, *** phenylsilane, # 1,4-dioxane).

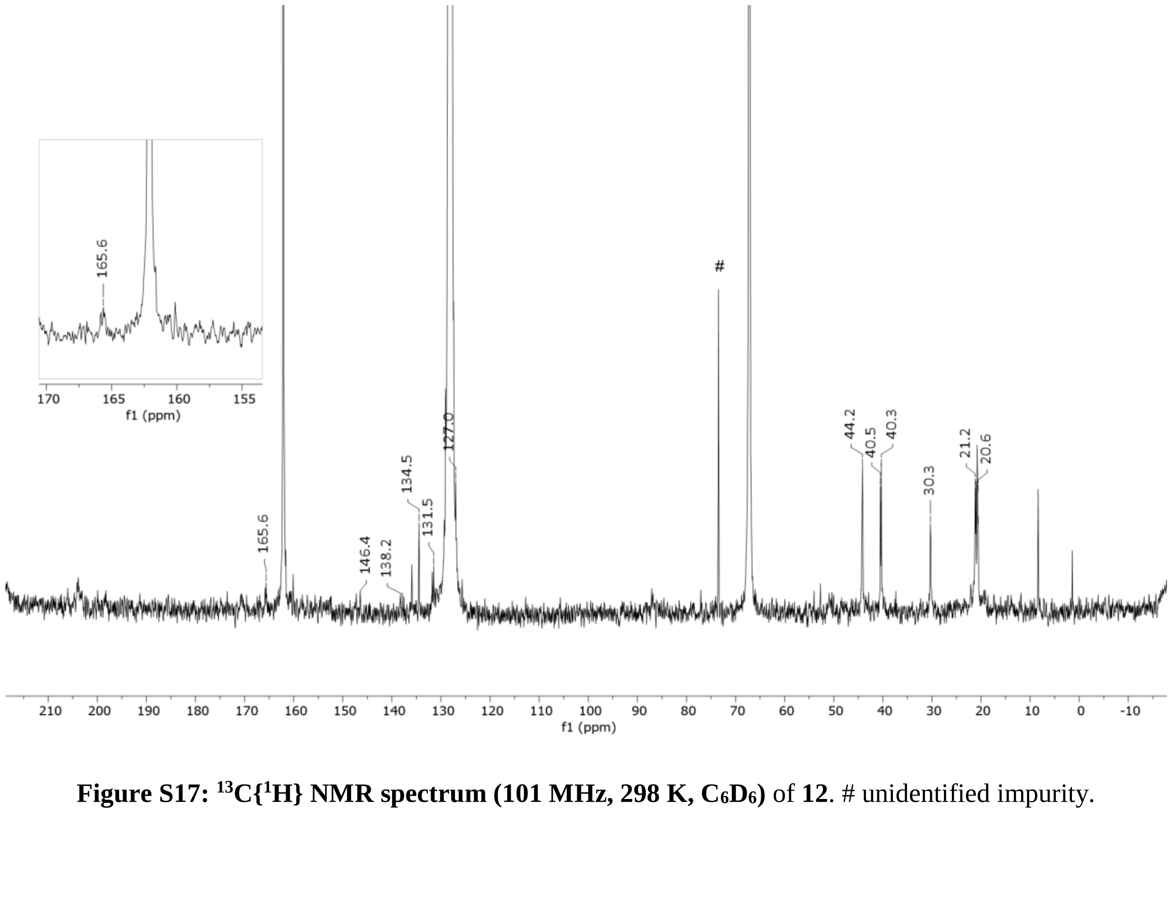

Figure S17: ^{13}C{^{1}H} NMR spectrum (101 MHz, 298 K, C_6D_6) of **12**. # unidentified impurity.

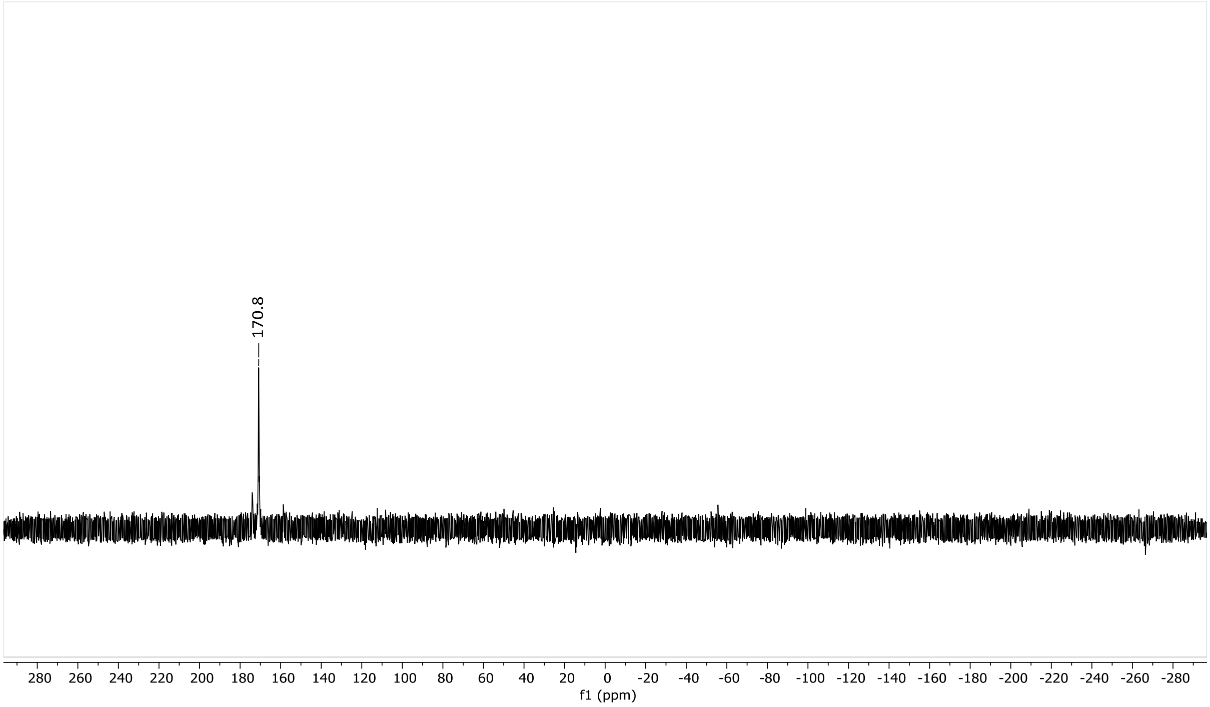

Figure S18: $^{31}P\{^{1}H\}$ NMR spectrum (162 MHz, C_6D_6, 298 K) of **12**.

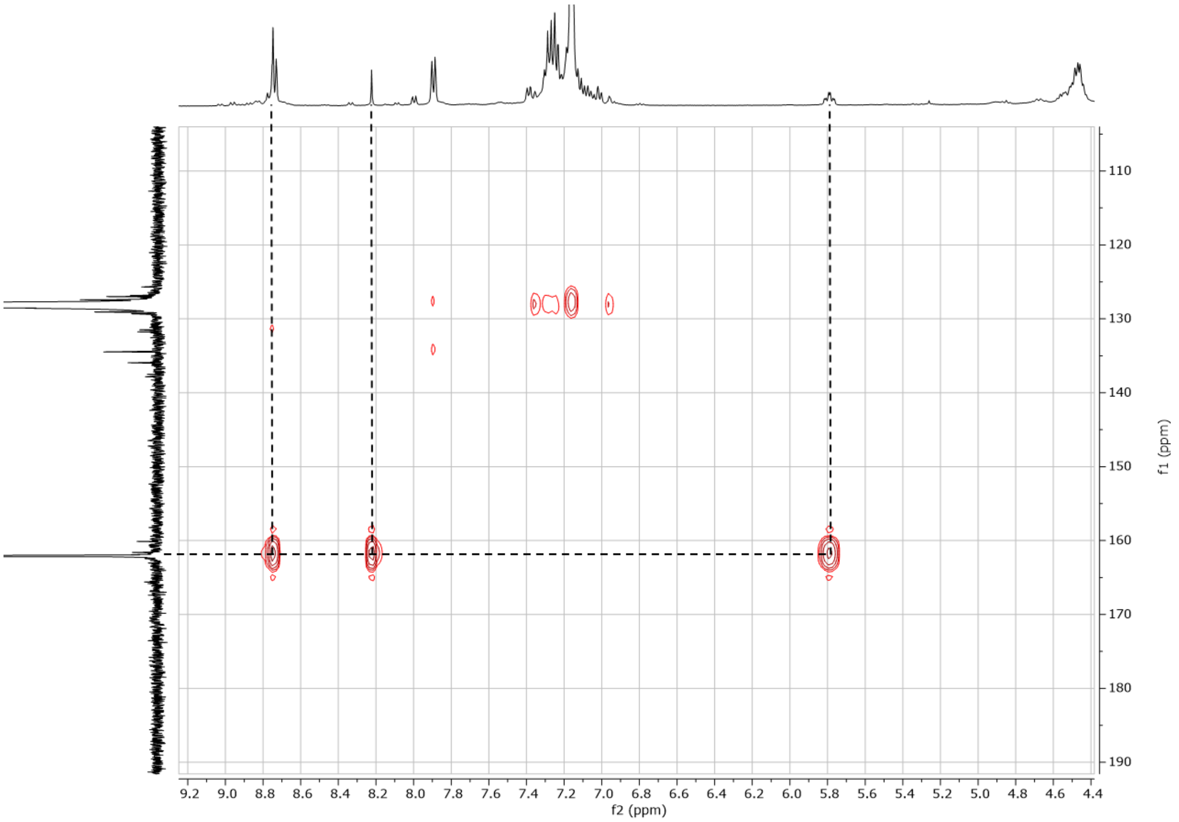

Figure S19: $^{13}C^{1}H$ HMBC NMR spectrum (298 K, C_6D_6) of **12**.

2.2 Catalytic Hydrosilylation of Carbon Dioxide

2.2.1 Catalytic activity of 9

In a flame dried Schlenk tube, solvent (0.5 mL), the respective additive, and phenylsilane (271 mg, 2500 µmol) were added to the respective catalyst (5 µmol). Followed by the addition of CO_2 by one freeze-pump-thaw cycle, the flask was left open to a continuous stream of CO_2. The reaction mixture was stirred at 80 °C for 4 h. After that, mesitylene was added as an internal standard. The mixture was filtered through a syringe filter and the amount of silylated products was analyzed by quantitative $^{13}C\{^{1}H\}$ NMR spectroscopy in accordance to the previous reports[2-3] with an estimated error of ±5%.

Table S1: Hydrosilylation of carbon dioxide with phenylsilane.

**Entry**	**Catalyst**	**Additive**	**Conversion Si–H**	**TON Formate**	**TON Acetal**	**TON Methoxide**
1	5	4 mol% KOtBu	29%	25	18	7
2	5	2.1 mol% $NaBEt_3H$	41%	13	39	12
3	9	-	35%	40	43	11

Cobalt catalyst **5** or **9** (1 mol%), CO_2 (1 bar), $PhSiH_3$ (2500 µmol), 80 °C, 4 h.

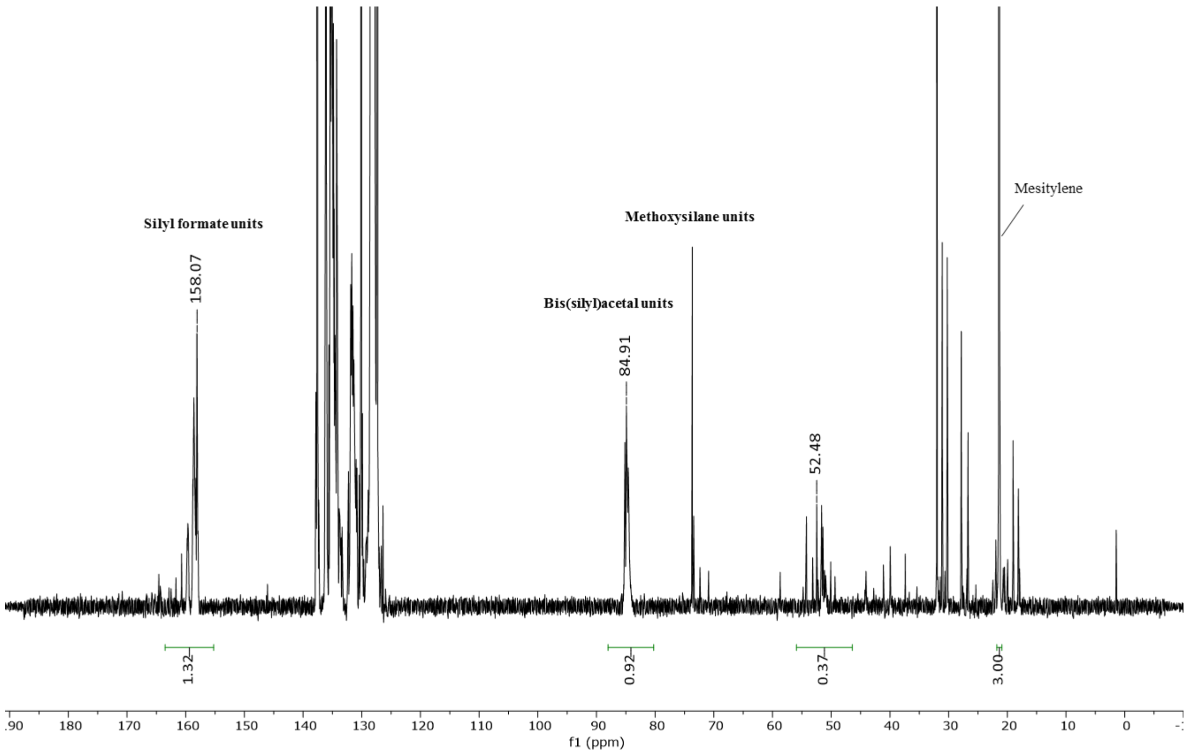

Figure S20: $^{13}C\{^{1}H\}$ NMR spectrum (126 MHz, 298 K, C_6D_6) of the reaction mixture for Table S1, Entry 1.

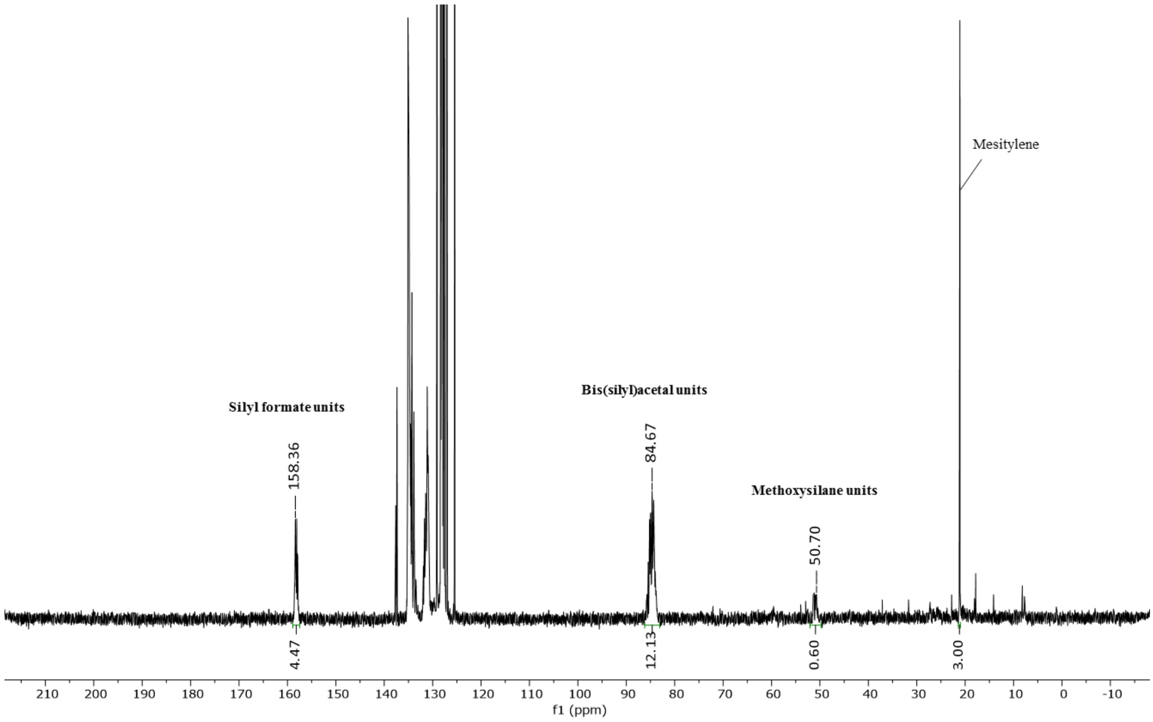

Figure S21: $^{13}C\{^{1}H\}$ NMR spectrum (126 MHz, 298 K, C_6D_6) of the reaction mixture for Table S1, Entry 2.

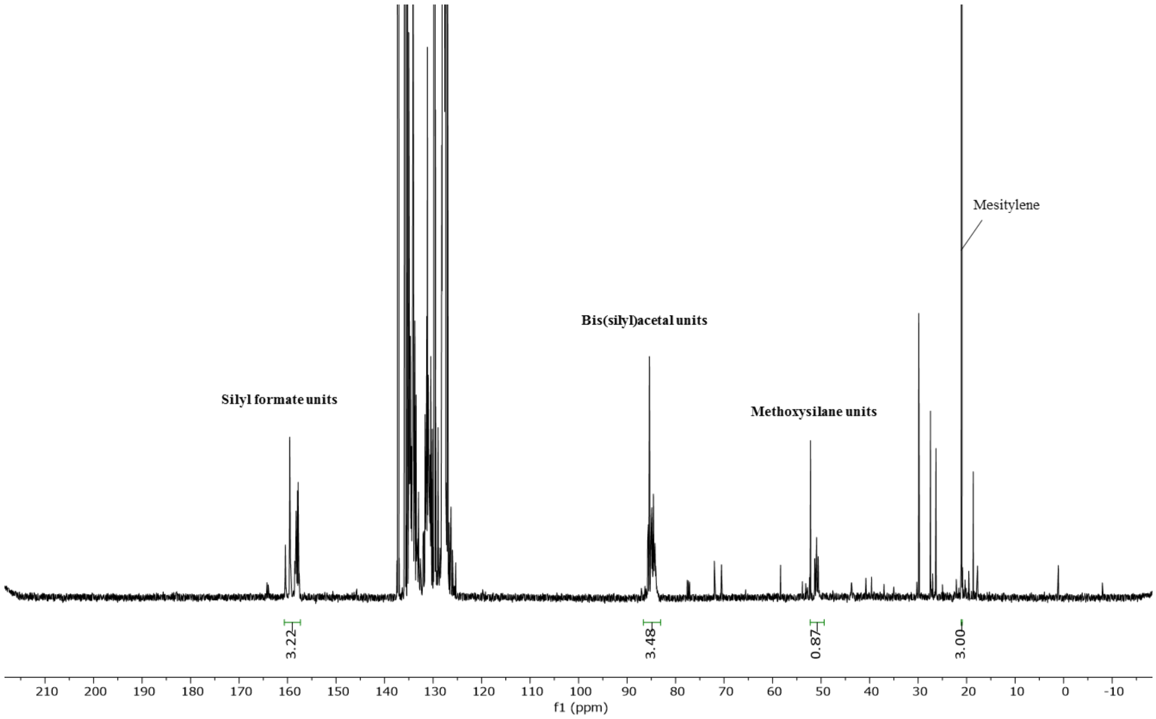

Figure S22: $^{13}C\{^{1}H\}$ NMR spectrum (126 MHz, 298 K, C_6D_6) of the reaction mixture for Table S1, Entry 3.

2.2.2 Catalytic activity of 10 and 11

In a flame-dried Schlenk tube, phenylsilane (271 mg, 2500 µmol) and benzene-d_6 (0.5 mL) were added to the respective catalyst (5 µmol). Subsequently, $^{13}CO_2$ (1 bar) was added by one freeze-pump-thaw cycle, and the reaction mixture was stirred at 80 °C for 4 h. After that, mesitylene (30 mg, 0.25 mmol) was added as an internal standard. The mixture was filtered through a syringe filter, and the silylated products were analyzed by quantitative $^{13}C\{^1H\}$ NMR spectroscopy by following the procedure reported in previous reports[2-3] with an estimated error of ±5%.

Table S2: Hydrosilylation of carbon dioxide with phenylsilane.

Entry	Catalyst	Conversion $^{13}CO_2$	Selectivity Formate	Selectivity Acetal	Selectivity Methoxide
1	10	35%	83%	10%	7%
2	11	78%	53%	34%	13%

Cobalt catalyst **10** or **11** (0.2 mol%), $^{13}CO_2$ (1 bar), C_6D_6, $PhSiH_3$ (2500 µmol), 80 °C, 4 h.

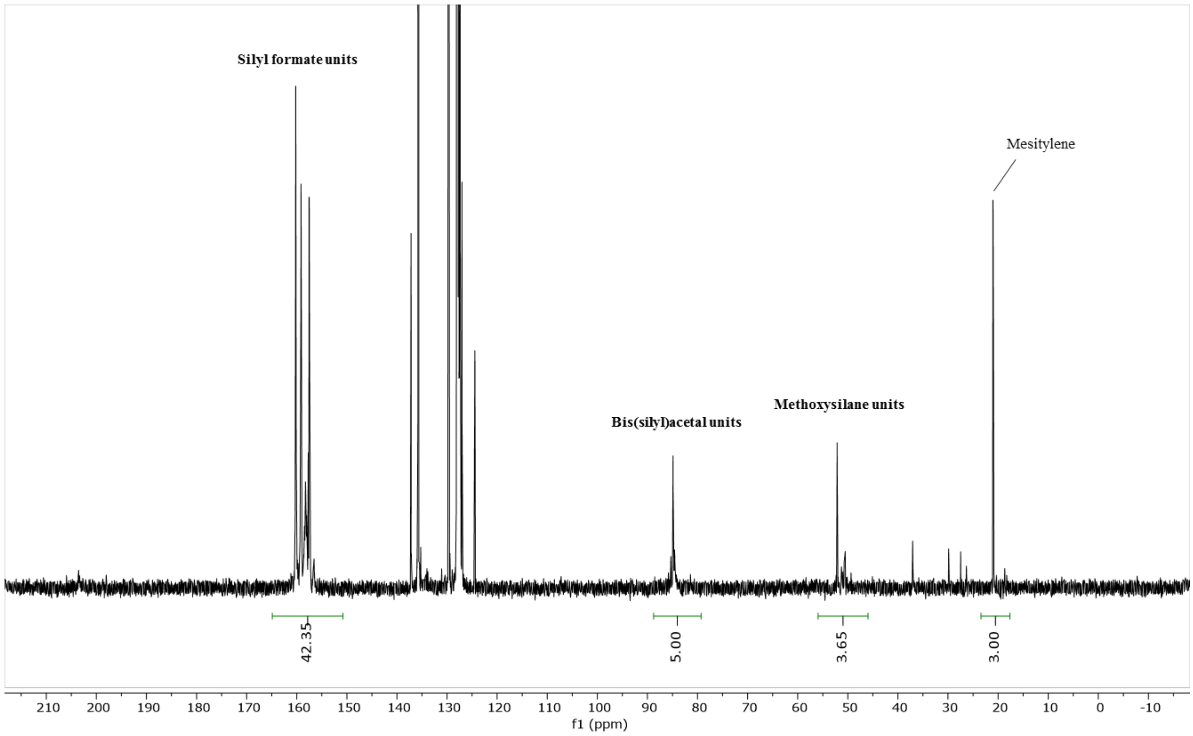

Figure S23: $^{13}C\{^{1}H\}$ NMR spectrum (126 MHz, 298 K, C_6D_6) of the reaction mixture for Table S2, Entry 1.

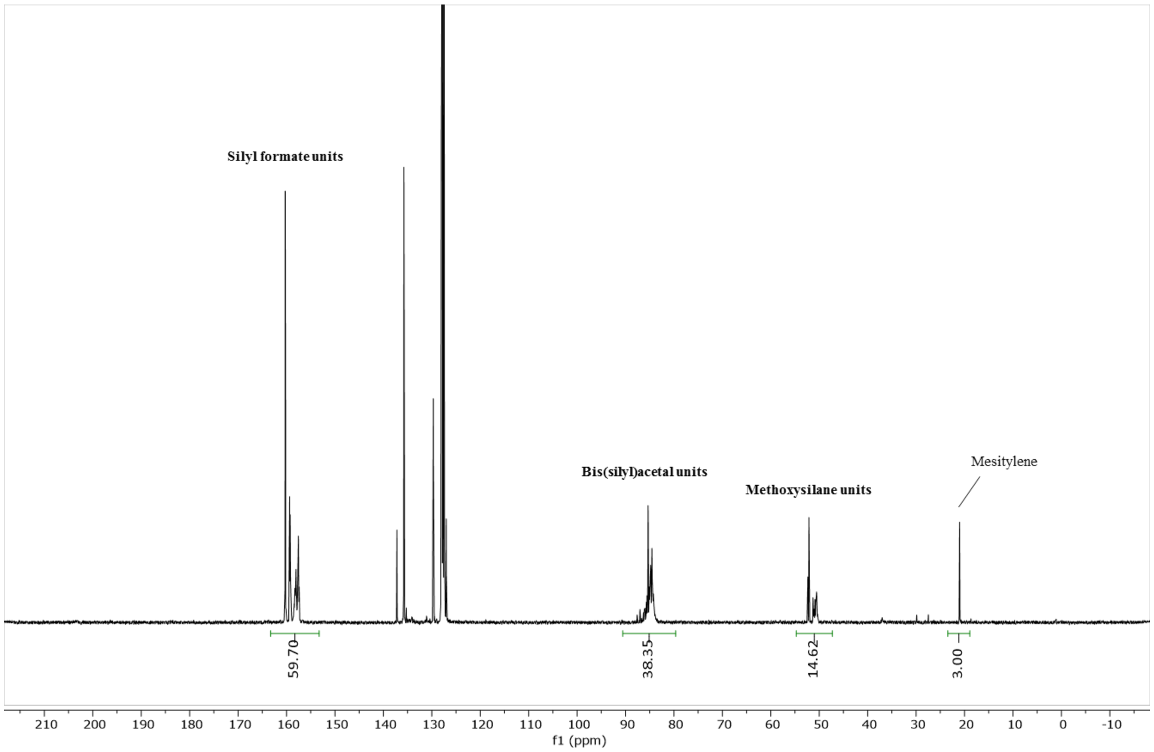

Figure S24: $^{13}C\{^{1}H\}$ NMR spectrum (126 MHz, 298 K, C_6D_6) of the reaction mixture for Table S2, Entry 2.

2.3 Control Experiments

2.3.1 Reaction of 10 with CO_2

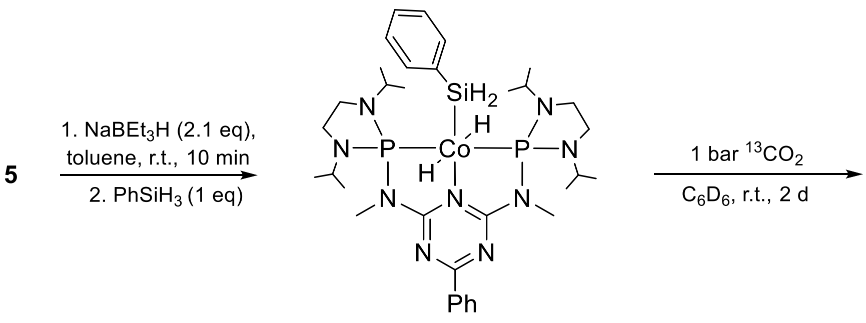

10

$NaBEt_3H$ (46 µL, 0.046 mmol, 1M in toluene) was added to **5** (15 mg, 0.022 mmol) in toluene (1 mL). The mixture was stirred for 10 min at room temperature. After that time, the volatiles were removed in vacuo, and C_6D_6 (0.6 mL) were added to the residue. The mixture was filtrated, 15 mg 1,4-dioxane was added as an internal standard and the yield of **9** determined by ^1H NMR spectroscopy (0.0068 mmol, 31%). Subsequently, the required amount of phenylsilane (0.8 µL, 0.0068 mmol) was added. The solution was frozen, the headspace evacuated and the mixture exposed to 1 bar $^{13}CO_2$. After thawing and careful shaking, the sample was analyzed by NMR spectroscopy.

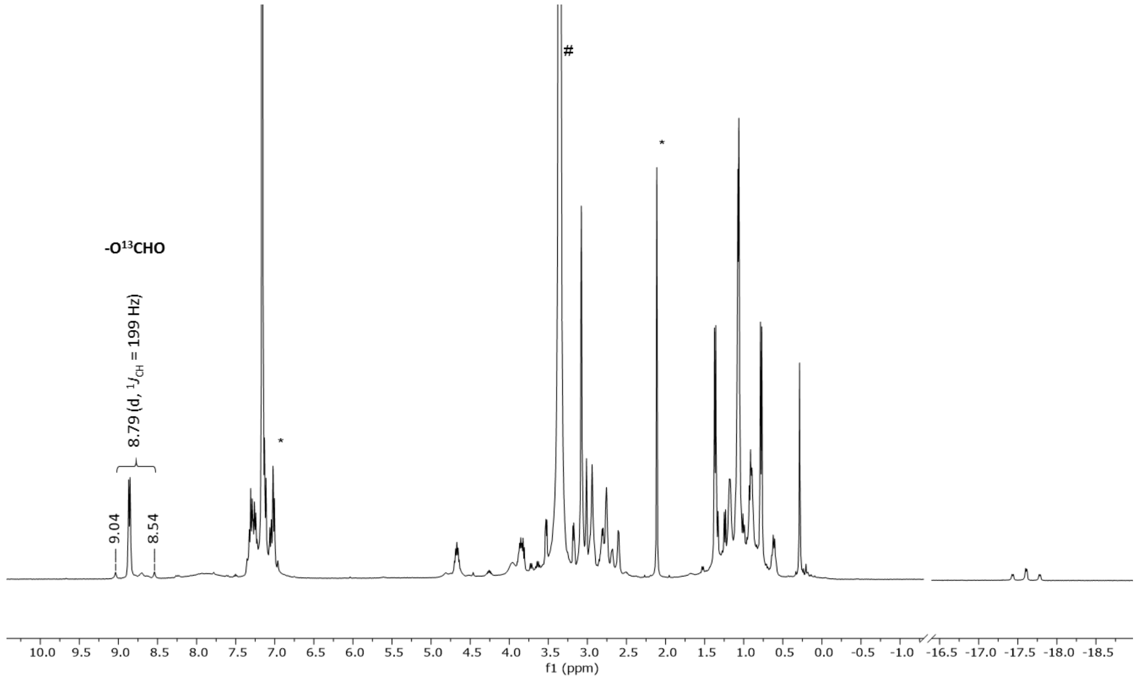

Figure S25: ^{1}H NMR (400 MHz, 298 K, C_6D_6) of the reaction of **10** with $^{13}CO_2$ after 2 d; * - Toluene, # - 1,4-dioxane.

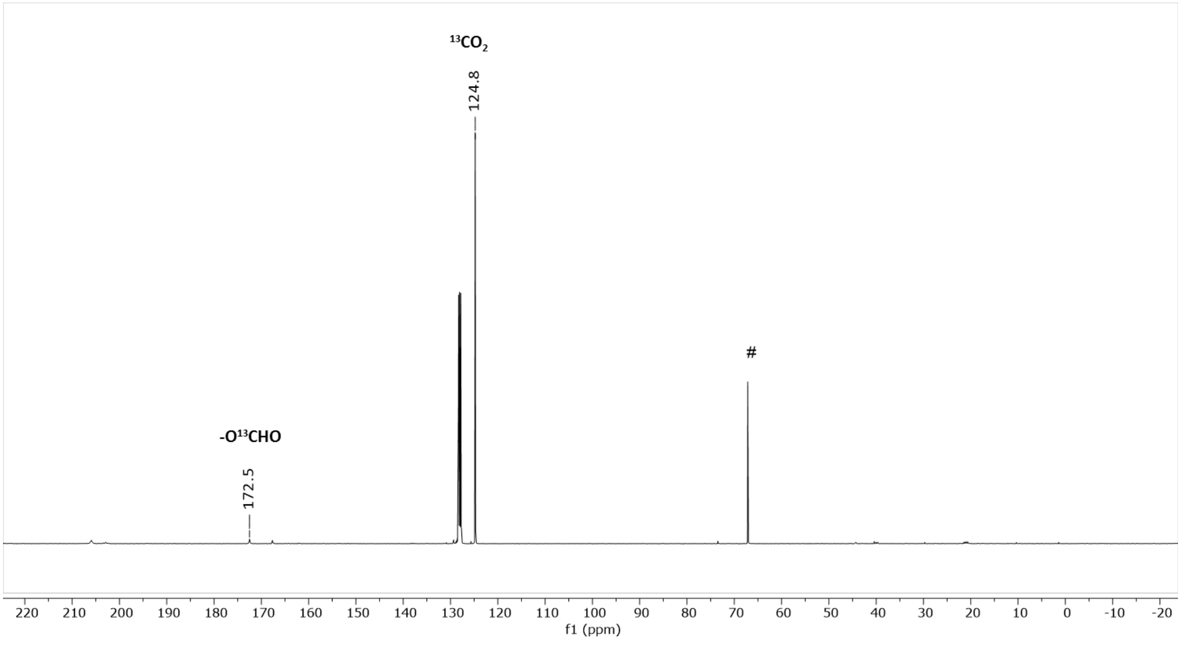

Figure S26: $^{13}C\{^1H\}$ NMR spectrum (101 MHz, C_6D_6, 298 K) of the reaction of **10** with $^{13}CO_2$ after 2 d; # - 1,4-dioxane.

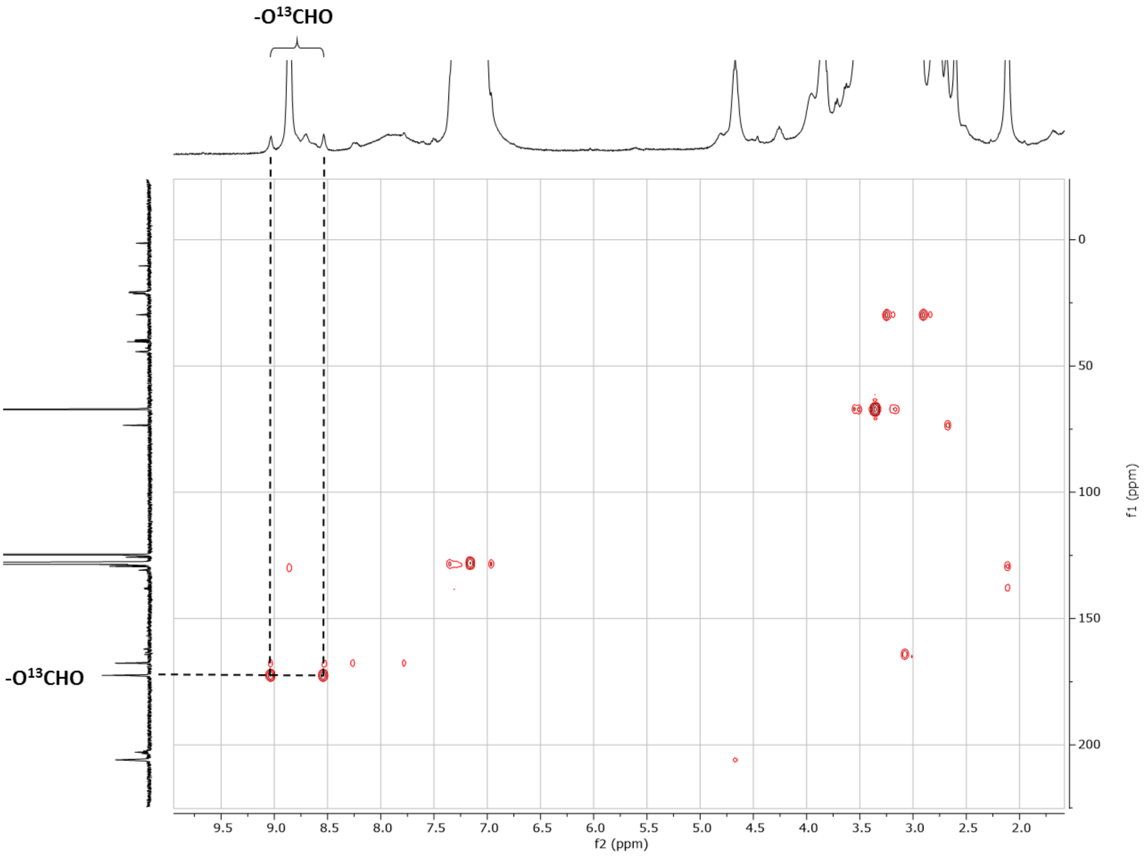

Figure S27: $^{13}C^{1}H$ HMBC NMR spectrum (298 K, C_6D_6) of the reaction of **10** with $^{13}CO_2$ after 2 d.

2.3.2 Reaction of 11 with xs. $PhSiH_3$

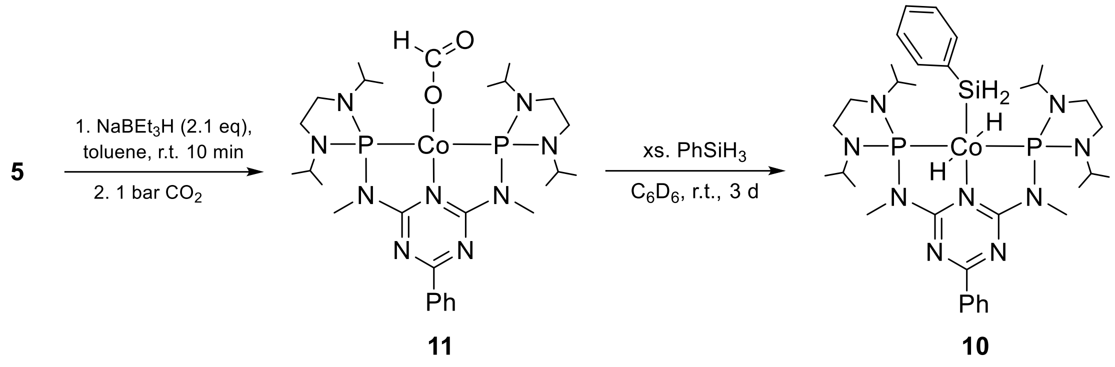

$NaBEt_3H$ (46 µL, 0.046 mmol, 1M in toluene) was added to **5** (15 mg, 0.022 mmol) in toluene (1 mL). The mixture was stirred for 10 min at room temperature. Subsequently, the mixture was frozen and the headspace evacuated and exposed to 1 bar $^{13}CO_2$. The reaction mixture was stirred for 15 min while warming to room temperature, followed by removal of the volatiles in vacuum. The residue was dissolved in C_6D_6, the solution was filtered and phenylsilane (2.8 µL, 0.022 mmol) was added. The tube was carefully shaken and the solution analyzed by NMR spectroscopy.

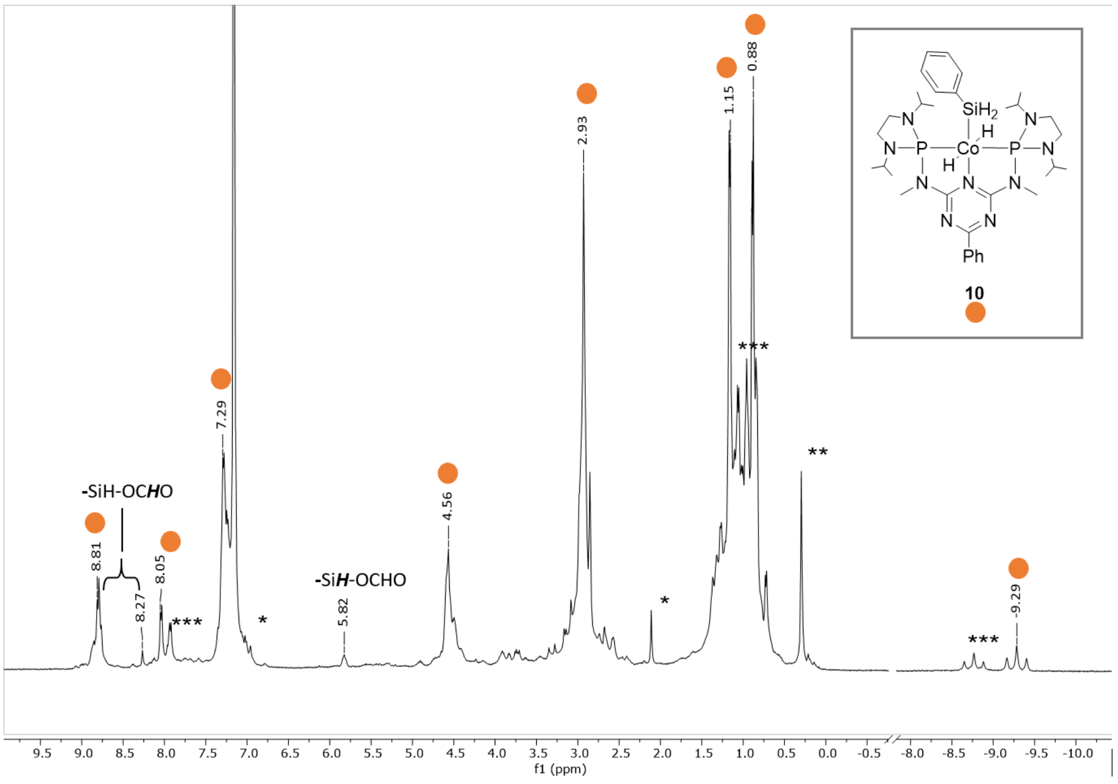

Figure S 28: ^{1}H NMR (400 MHz, 298 K, C_6D_6) of the reaction of **11** with xs. $PhSiH_3$, * - Toluene, ** - silicone grease, *** - unidentified impurity).

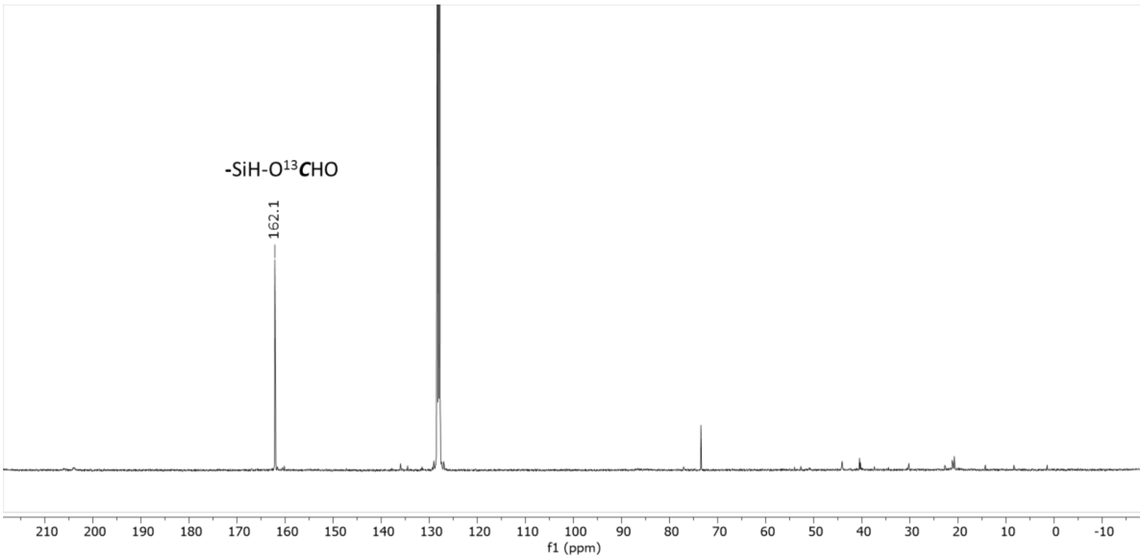

Figure S29: $^{13}C\{^{1}H\}$ NMR (101 MHz, 298 K, C_6D_6) of the reaction of **11** with xs. $PhSiH_3$.

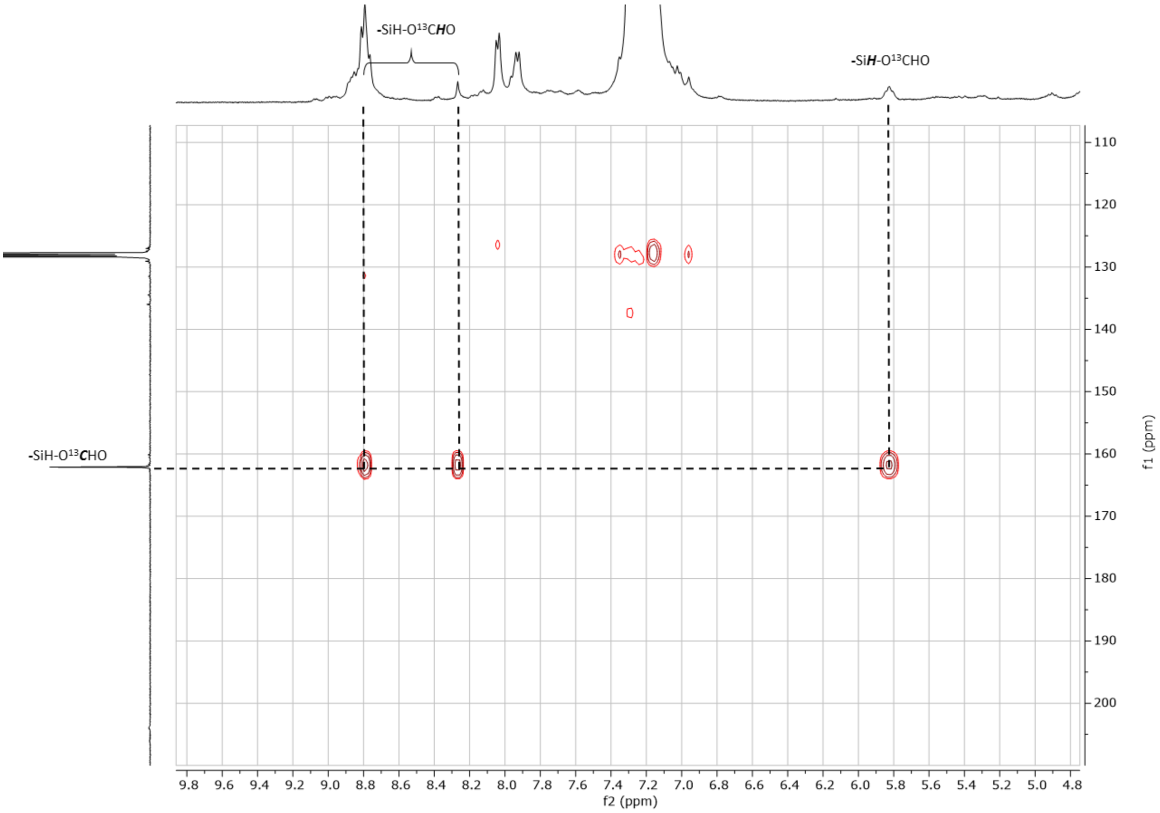

Figure S30: $^{13}C^{1}H$ HMBC NMR (298 K, C_6D_6) of the reaction of **11** with xs. $PhSiH_3$.

2.3.3 Preparation of 10 from 5, KO^{t}Bu and $PhSiH_3$

Phenylsilane (6.3 mg, 0.058 mmol) was added to a mixture of **5** (20.0 mg, 0.029 mmol) and potassium *tert*-butoxide (6.5 mg, 0.058 mmol) in toluene (3 mL). The resulting mixture was stirred for 10 min at room temperature. The volatiles were then removed under vacuum. The residue was washed with pentane (2×5 mL) and dried under reduced pressure. Finally, the product was dissolved in C_6D_6 (0.5 mL) and analyzed by ^{1}H NMR spectroscopy.

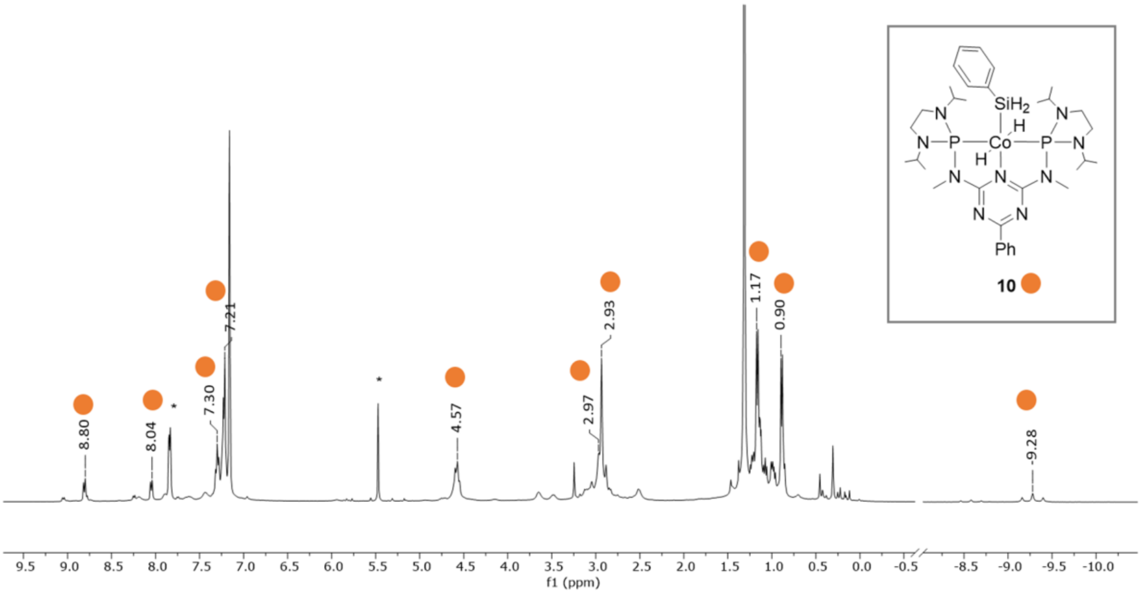

Figure S31: ^{1}H NMR (400 MHz, 298 K, C_6D_6) of the reaction of **5** with KO^{t}Bu and $PhSiH_3$ (* unidentified impurity).

2.3.4 Reaction of 5 and KO^{t}Bu

KO^{t}Bu (3.3 mg, 0.029 mmol) was added to a solution of **5** (10.00 mg, 0.015 mmol) in C_6D_6 (0.5 mL) in an NMR tube at room temperature. The mixture was carefully shaken and analyzed by ^{1}H NMR spectroscopy after 5 min. The ^{1}H NMR spectrum indicates that **5** remains present in the reaction mixture.

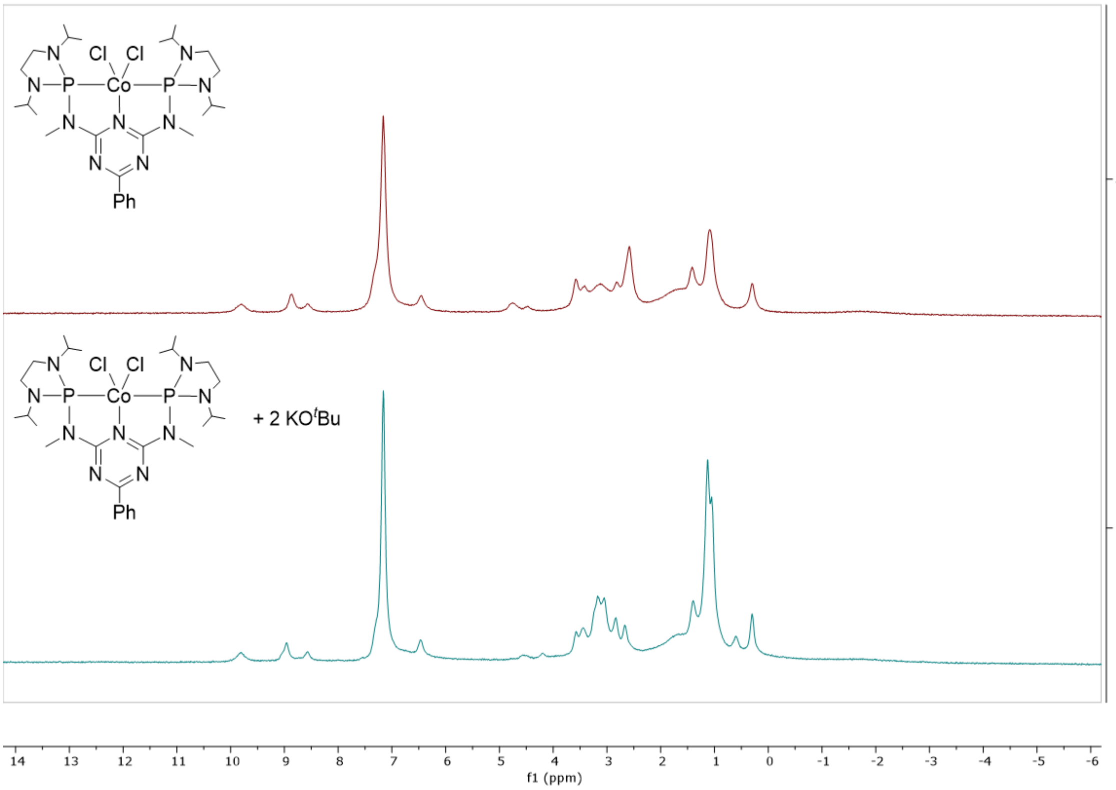

Figure S32: ^{1}H NMR (400 MHz, 298 K, C_6D_6) of **5** (top) and a mixture of **5** and KO^{t}Bu (bottom).

2.3.5 NMR analysis of the reaction mixture containing 9, $PhSiH_3$, and CO_2 under catalytic conditions

A solution of $NaBEt_3H$ in toluene (46 µL, 0.046 mmol) was added to **5** (15 mg, 0.022 mmol) in toluene-d_8 (0.6 ml), and the mixture was stirred for 5 min at room temperature. $PhSiH_3$ (27 µL, 0.22 mmol) was added, and the solution was transferred into an NMR tube. After one freeze-pump-thaw-cycle, CO_2 (1 bar) was added. The tube was shaken carefully, and the mixture was analyzed by ^1H NMR spectroscopy. The ^1H NMR spectra indicate that neither **10** nor **12** formed during the reaction.

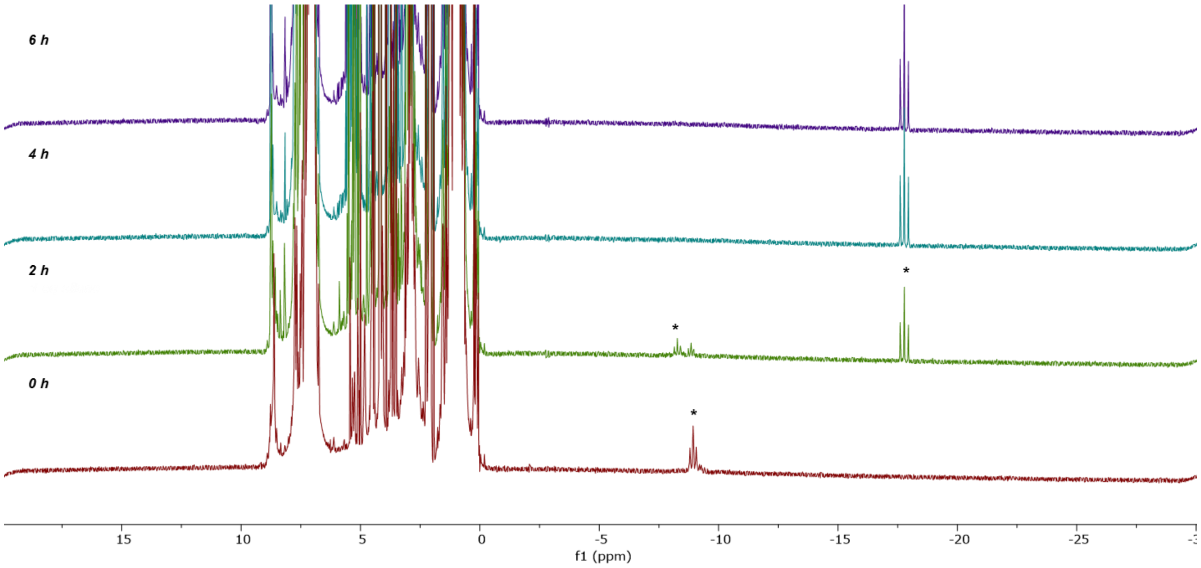

Figure S 33: ^1H NMR (400 MHz, 298 K, C_6D_6) of the reaction of **9** with 10 eq $PhSiH_3$ and 1 bar $^{13}CO_2$ (* unidentified compounds).

3. Computational Details

All calculations were performed with the ORCA program package.[4-5] All structures were calculated without any truncations of the ligand. The hybrid functional B3LYP was used for geometry optimizations and frequency calculations[6-8] in combination with the atom-pairwise dispersion correction with Becke-Johnson damping D3BJ from Grimme.[9-10] Key bond lengths and angles of the calculated structure of **5** were compared to the molecular structure determined by X-ray diffraction[1] to justify the functional choice. Several DFT functionals could successfully reproduce the geometrical features within the expected uncertainty of the calculations, as seen in Table S3. To accelerate the calculations, the density fitting (RI-J) and "chain of spheres" (COSX) approximation (RIJCOSX)[11] was used with the auxiliary basis set def2/J.[12] The geometry optimizations and frequency calculations were performed with Ahlrichs triple-ζ quality basis set Def2-TZVP for the first coordination sphere (Co, Cl, P, Si, O, N, CO_2, and H), while the triple-ζ quality basis set Def2-SVP was used for all other atoms.[13] The following thresholds were used for optimizations: energy change tolerance of 1 x 10^{-6} a.u., root mean square gradient of 3 x 10^{-5} a.u., a maximum gradient of 1 x 10^{-4} a.u., root mean square step of 6 x 10^{-4} a.u. and maximum step of 1 x 10^{-3} a.u. The DFT integration grid was set to Grid5. Tight convergence criteria (energy tolerance of 1 x 10^{-8} a.u.) were used for all calculations. The geometry optimizations were performed without constraints. The conductor-like polarizable continuum (CPCM) model was used to describe solvation effects during the geometry optimizations and frequency calculations of the intermediates. Benzene (ε = 2.28) was chosen as solvent. Calculations of harmonic vibrational frequencies were performed to ensure that the number of imaginary frequencies is zero for local minima and one for transition states. The zero-point vibrational energies, thermal corrections to the electronic energies (298 K) and the entropy contributions were taken from the frequency calculations. The Gibbs free energies of each structure were corrected to the reference state of 1M by a factor of +1.89 kcal mol^{-1}.

Table S3: Molecular structure of **5** (left) and key bond lengths and angles of the molecular structure of **5** determined by XRD[1] and the calculated geometry obtained with different DFT functionals (def2-TZVP (selected atoms), def-SVP; no solvation model). H atoms were omitted for clarity.

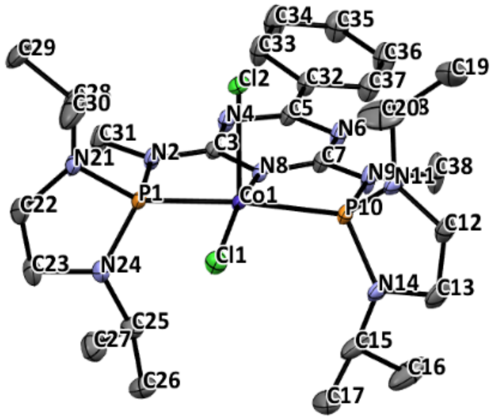

Bond/Angle [Å / °]	XRD	B3LYP	BP86	M06L
Co1–Cl1 (plan)	2.22	2.25	2.22	2.25
Co1–Cl2 (ax)	2.39	2.43	2.37	2.43
Co1–P1	2.21	2.18	2.15	2.18
Co1–P10	2.22	2.19	2.15	2.18
Co1–N8	1.92	1.95	1.91	1.94
P1–N2	1.73	1.75	1.76	1.75
P1–N21	1.66	1.66	1.68	1.66
N2–C3	1.35	1.36	1.37	1.36
C3–N8	1.36	1.35	1.37	1.36
P10–N9	1.74	1.75	1.76	1.75
P10–N14	1.65	1.66	1.67	1.66
N9–C7	1.35	1.36	1.37	1.36
C7–N8	1.35	1.35	1.37	1.36
C7–N6	1.33	1.34	1.34	1.33
N2–C31	1.48	1.45	1.46	1.45
N9–C38	1.46	1.46	1.46	1.45
Cl1–Co1–Cl2	107.8	115.14	113.14	113.13
Cl1–Co1–P1	94.6	92.41	92.48	93.91
Cl1–Co1–N8	164.9	158.57	159.60	160.82
Cl1–Co1–P10	94.1	94.09	92.54	93.65
Cl2–Co1–P1	94	95.29	95.71	95.19
Cl2–Co1–N8	87.3	86.26	87.26	86.05
Cl2–Co1–P10	94.9	95.88	95.69	94.31
N8–Co1–P10	84.8	84.25	85.10	84.16
N8–Co1–P1	83.7	84.07	85.12	84.23
P1–Co1–P10	165.1	163.26	164.59	164.44
Co1–P1–N2	99.1	99.57	99.70	99.48
Co1–P1–N21	130.1	132.60	132.41	132.48
Co1–P10–N9	97.9	99.22	99.72	99.51
Co1–P10–N14	121.1	117.38	117.89	117.72
P1–N2–C3	117.1	116.33	115.80	116.68
P10–N9–C7	118.3	116.82	115.78	116.57

Table S4: Imaginary frequencies calculated at the B3LYP level of theory.

TS	Imaginary frequency
TS1	$-155.67\ cm^{-1}$
TS2	$-114.96\ cm^{-1}$
TS3	$-188.43\ cm^{-1}$
TS4	$-508.69\ cm^{-1}$

Table S5: Selected IR frequencies calculated at the B3LYP level of theory.

**9**	Co–H	$1771.21\ cm^{-1}$
**10**	H–Co–H	$1968.63\ cm^{-1}$, $1759.84\ cm^{-1}$
**11**	C–O	$1667.89\ cm^{-1}$, $1313.92\ cm^{-1}$

Figure S34: Geometries of the key intermediates and transition states. Selected C and H atoms were omitted for clarity.

**9**<br>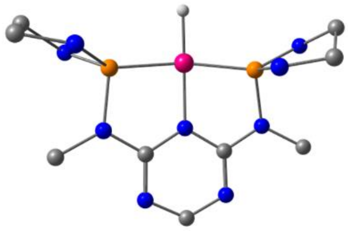<br>Co–H 153 pm<br>Co–P 211 pm<br>Co–N 192 pm	**15**<br>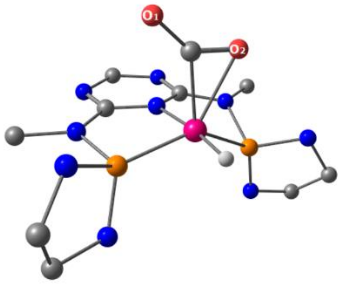<br>Co–H 151 pm<br>Co–C 197 pm, Co–O2 223 pm<br>C–O1 121 pm, C–O2 124 pm	**14**<br>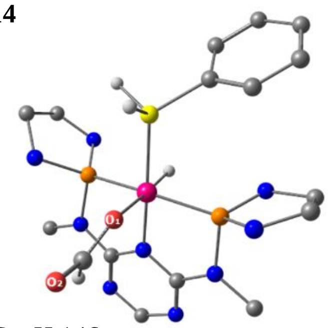<br>Co–H 146 pm<br>Co–Si 226 pm, Co–O 200 pm<br>C–O1 128 pm, C–O2 122 pm<br>C–H 111 pm
**18**<br>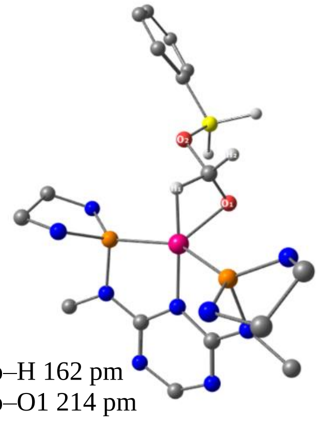<br>Co–H 162 pm<br>Co–O1 214 pm<br>C–O1 130 pm, C–O2 143 pm<br>C–H1 125 pm, C–H2 110 pm	**23**<br>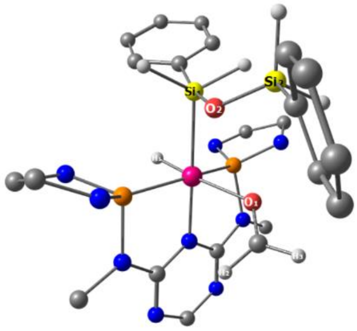<br>Co–H 144 pm<br>Co–O 208 pm<br>Co–Si 235 pm<br>Si1–O 188 pm, Si2–O 161 pm	**TS1**<br>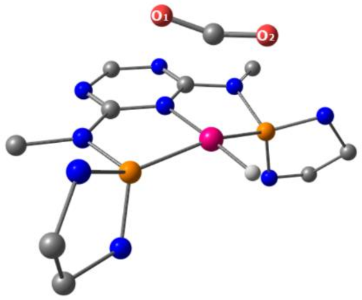<br>Co–H 152 pm<br>Co–C 255 pm<br>C–O1 118 pm, C–O2 117 pm
**TS2**<br>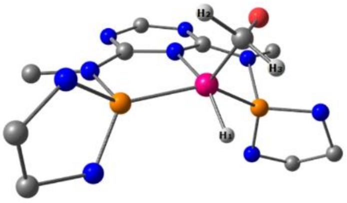<br>Co–H 148 pm<br>Co–C 205 pm<br>C–O 128 pm<br>C–H 109 pm<br>Co–O 206 pm	**TS3**<br>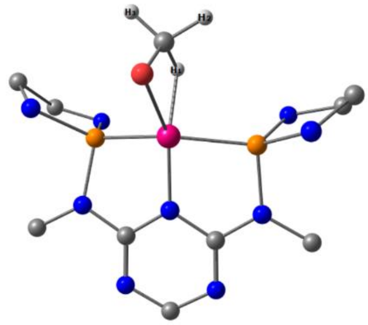<br>Co–H1 175 pm<br>Co–C 224 pm<br>C–H2, C–H3 111 pm<br>C–H1 118 pm<br>Co–O 207 pm	**TS4**<br>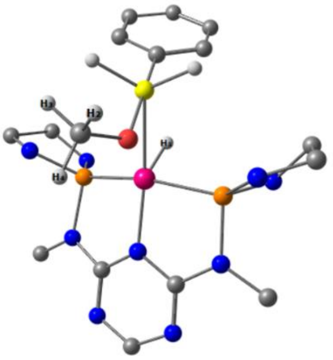<br>Co–H 145 pm<br>Co–Si 233 pm<br>C–O 140 pm<br>C–H1, H2, H3 110 pm<br>Co–O 214 pm<br>H–Co–Si 61°, Co–Si–O 60°

Table S6: Solvent-corrected electronic energies ($E_{el/sol}$) for B3LYP+D3BJ, non-thermal ZPE corrections (E_{ZPE}), thermal energy correction (E_{therm}), enthalpy correction (H_{therm}), and entropy correction (TS). All energies are given in Hartree.

Structure	$E_{el/sol}$	E_{ZPE}	E_{therm}	H_{therm}	-TS
**CO_2**	-188.59082316	0.01009254	0.00249275	0.00094421	0.02409076
**$PhSiH_3$**	-522.62306798	0.11512786	0.00614051	0.00094421	0.03737384
**$(PhSiH_2)OCHO$ (13)**	-711.24138829	0.13205729	0.00898981	0.00094421	0.04431007
**$(PhSiH_2O)_2CH_2$ (22)**	-1233.90182292	0.25505252	0.01624663	0.00094421	0.06094851
**$(PhSiH_2)_2O$**	-1119.39429800	0.22127568	0.01314765	0.00094421	0.05411528
**HCHO**	-114.49691885	0.02649370	0.00286769	0.00094421	0.02546616
**$(PhSiH_2)_2OCH_3$ (30)**	-637.19372640	0.15004776	0.00891070	0.00094421	0.04390305
**9**	-3614.22119186	0.74197559	0.04385247	0.00094421	0.11334509
**15**	-3802.82316300	0.75760781	0.04568638	0.00094421	0.11610373
**16**	-3802.82731445	0.75727365	0.04631487	0.00094421	0.11788196
**11**	-3802.85431443	0.75907714	0.04666187	0.00094421	0.11928943
**14**	-4325.51647979	0.87855285	0.05463116	0.00094421	0.13343127
**17**	-4325.48664140	0.87970985	0.05367398	0.00094421	0.13157557
**18**	-4325.47227760	0.88100400	0.05365315	0.00094421	0.13300930
**19**	-4325.51042515	0.88123012	0.05459299	0.00094421	0.13434208
**20**	-4848.15996623	1.00170727	0.06065275	0.00094421	0.14480975
**21**	-4848.16838838	1.00248332	0.06032170	0.00094421	0.14380293
**23**	-4848.11667875	0.99748734	0.06169819	0.00094421	0.14676139
**24**	-4733.65053753	0.96786039	0.05928532	0.00094421	0.14306677
**25**	-3728.75713840	0.77504443	0.04485354	0.00094421	0.11420655
**26**	-3728.75150766	0.77750765	0.04615111	0.00094421	0.11759074

**27**	-3728.77961898	0.77642975	0.04681643	0.00094421	0.11923123
**28**	-4251.43160104	0.89643538	0.05326325	0.00094421	0.13026408
**29**	-4251.43124395	0.89621151	0.05329487	0.00094421	0.13140029
**TS1**	-3802.81779069	0.75445254	0.04702311	0.00094421	0.11950221
**TS2**	-3728.74583492	0.77374444	0.04545687	0.00094421	0.11599282
**TS3**	-3728.74644868	0.77475099	0.04599720	0.00094421	0.11786869
**TS4**	-4251.41376763	0.89513166	0.05350123	0.00094421	0.13096423
**32**	-4135.69960031	0.84341703	0.04964877	0.00094421	0.12456998
**HOCHO**	-189.76410412	0.03325969	0.00324382	0.00094421	0.02826891
**33**	-4514.14165709	0.89493632	0.05807373	0.00094421	0.14081998
**34**	-4514.11187907	0.89603953	0.05700756	0.00094421	0.13875009
**10**	-4136.90444144	0.86109751	0.05114815	0.00094421	0.12670454
**10’**	-4136.89333287	0.86120018	0.04944349	0.00094421	0.12351069
**12**	-4325.53215650	0.87761517	0.0536108	0.00094421	0.13205296
**12’**	-4325.52153701	0.87759930	0.05373372	0.00094421	0.13238953
**14’**	-4325.50037247	0.87789228	0.05306100	0.00094421	0.13089307
**14’’**	-4325.48535324	0.87963195	0.05316185	0.00094421	0.13014690
**20’**	-4848.14959490	0.99985204	0.06099713	0.00094421	0.14664719
**20’’**	-4848.14009420	1.00129345	0.05944333	0.00094421	0.14184815

4. Orbital Localization Output

Occupied MOs

```
------------------------------------------------------------------------------
                    ORCA ORBITAL LOCALIZATION
------------------------------------------------------------------------------

Input orbitals are from                ... 571_C2B3a_567.gbw
Output orbitals are to                 ... 571_C2B3a_567_loc_output.gbw
Max. number of iterations              ... 128
Localizations seeded randomly          ... on
Convergence tolerance                  ... 1.000e-06
Treshold for strong local MOs          ... 9.500e-01
Treshold for bond MOs                  ... 8.500e-01
Operator                               ... 0
Orbital range for localization         ... 150 to 175
Localization criterion                 ... PIPEK-MEZEY
Warning: cannot retrieve the overlap matrix S
        ... Overlap was succesfully recalculated
Entering Jacobi type localization:
Using Cholesky decomposition as initial guess.
Initial value of the localization sum :     7.716486
ITERATION   0 : L=    8.7363572136 DL=  1.02e+00 (MAX-T)=  44.507
ITERATION   1 : L=    8.7588276417 DL=  2.25e-02 (MAX-T)=   4.327
ITERATION   2 : L=    8.7589654237 DL=  1.38e-04 (MAX-T)=   1.064
ITERATION   3 : L=    8.7589705261 DL=  5.10e-06 (MAX-T)=   0.118
ITERATION   4 : L=    8.7589705548 DL=  2.87e-08 (MAX-T)=   0.006
LOCALIZATION SUM CONVERGED


------------------------------------------------------------------------------
               LOCALIZED MOLECULAR ORBITAL COMPOSITIONS
------------------------------------------------------------------------------

The Mulliken populations for each LMO on each atom are computed
The LMO`s will be ordered according to atom index and type
  (A) Strongly localized MO`s have populations of >=0.950 on one atom
  (B) Two center bond orbitals have populations of >=0.850 on two atoms
  (C) Other MO`s are considered to be `delocalized`

FOUND  -   0 strongly local MO`s
       -   6 two center bond MO`s
       -  20 significantly delocalized MO`s

Bond-like localized orbitals:
MO 155:  89O  -   0.477872  and  88O  -   0.467100
MO 154:  89O  -   0.572257  and  88O  -   0.336421
MO 153:   2P  -   0.064217  and   0Co -   0.846502
MO 152:   1H  -   0.518583  and   0Co -   0.384474
MO 151:   1H  -   0.001081  and   0Co -   0.941438
MO 150:   1H  -   0.001145  and   0Co -   0.932398
More delocalized orbitals:
MO 175:  0Co- 0.084 2P - 0.107 11P - 0.435
MO 174:  5N - 0.118 6C - 0.109 9N - 0.341
MO 173:  58C - 0.166 60C - 0.220 61H - 0.122 64C - 0.248 67H - 0.163
MO 172:  20C - 0.151 21H - 0.143 22C - 0.078 26C - 0.093
MO 171:  30C - 0.162 32C - 0.219 35H - 0.117 36C - 0.255 37H - 0.169
```

MO 170: 3N - 0.441 5N - 0.207
MO 169: 48C - 0.179 49H - 0.166 50C - 0.121 53H - 0.079
MO 168: 72C - 0.118 73C - 0.103 74H - 0.084 75C - 0.093 76H - 0.087 77C - 0.112 79C - 0.093 80H - 0.082 81C - 0.101 82H - 0.088
MO 167: 58C - 0.223 59H - 0.201 60C - 0.090 63H - 0.091 64C - 0.099 65H - 0.100
MO 166: 30C - 0.218 31H - 0.208 32C - 0.096 33H - 0.100 36C - 0.100 39H - 0.100
MO 165: 73C - 0.247 75C - 0.245 79C - 0.251 81C - 0.245
MO 164: 72C - 0.224 75C - 0.131 77C - 0.382 79C - 0.126
MO 163: 5N - 0.627 9N - 0.095
MO 162: 12N - 0.597 14H - 0.078
MO 161: 7N - 0.198 10N - 0.446
MO 160: 19N - 0.586
MO 159: 7N - 0.641 9N - 0.094
MO 158: 0Co- 0.622 87C - 0.107 88O - 0.173
MO 157: 40N - 0.594
MO 156: 2P - 0.081 47N - 0.584
Localized MO's were stored in: 571_C2B3a_567_loc_output.gbw

Unoccupied MOs

```
------------------------------------------------------------------------------
                    ORCA ORBITAL LOCALIZATION
------------------------------------------------------------------------------

Input orbitals are from                  ... 571_C2B3a_567.gbw
Output orbitals are to                   ... 571_C2B3a_567_loc_output.gbw
Max. number of iterations                ... 128
Localizations seeded randomly            ... on
Convergence tolerance                    ... 1.000e-06
Treshold for strong local MOs            ... 9.500e-01
Treshold for bond MOs                    ... 8.500e-01
Operator                                 ... 0
Orbital range for localization           ... 176 to 195
Localization criterion                   ... PIPEK-MEZEY
Warning: cannot retrieve the overlap matrix S
       ... Overlap was succesfully recalculated
Entering Jacobi type localization:
Using Cholesky decomposition as initial guess.
Initial value of the localization sum :     4.273645
ITERATION   0 : L=    6.2460209101 DL=  1.97e+00 (MAX-T)=  34.041
ITERATION   1 : L=    6.2585078673 DL=  1.25e-02 (MAX-T)=   3.460
ITERATION   2 : L=    6.2585738627 DL=  6.60e-05 (MAX-T)=   0.236
ITERATION   3 : L=    6.2585759518 DL=  2.09e-06 (MAX-T)=   0.048
ITERATION   4 : L=    6.2585760379 DL=  8.61e-08 (MAX-T)=   0.018
LOCALIZATION SUM CONVERGED



------------------------------------------------------------------------------
               LOCALIZED MOLECULAR ORBITAL COMPOSITIONS
------------------------------------------------------------------------------

The Mulliken populations for each LMO on each atom are computed
The LMO`s will be ordered according to atom index and type
  (A) Strongly localized MO`s have populations of >=0.950 on one atom
  (B) Two center bond orbitals have populations of >=0.850 on two atoms
  (C) Other MO`s are considered to be `delocalized`

FOUND  -   0 strongly local MO`s
       -   1 two center bond MO`s
       -  19 significantly delocalized MO`s
```

Bond-like localized orbitals:
MO 176:   2P -   0.013424 and   0Co -   0.920925
More delocalized orbitals:
MO 195: 72C - 0.130 73C - 0.302 77C - 0.178 79C - 0.302
MO 194: 5N - 0.153 6C - 0.137 8C - 0.364 9N - 0.080 10N - 0.083
MO 193: 87C - 0.585 88O - 0.134 89O - 0.172
MO 192: 72C - 0.130 75C - 0.302 77C - 0.178 81C - 0.302
MO 191: 3N - 0.082 4C - 0.316 6C - 0.211 7N - 0.151 9N - 0.089
MO 190: 0Co- 0.143 2P - 0.518
MO 189: 0Co- 0.094 11P - 0.575
MO 188: 0Co- 0.316 87C - 0.478
MO 187: 11P - 0.690
MO 186: 0Co- 0.310 1H - 0.335
MO 185: 2P - 0.448 43H - 0.076 46H - 0.095
MO 184: 74H - 0.082 76H - 0.264 78H - 0.346 80H - 0.290 82H - 0.110
MO 183: 21H - 0.101 24H - 0.192 26C --0.099 28H - 0.347 54C --0.118 55H - 0.077 56H - 0.278
MO 182: 17H - 0.271 22C --0.103 25H - 0.207 83C --0.141 84H - 0.232 85H - 0.099 86H - 0.117
MO 181: 41C --0.142 42H - 0.289 43H - 0.150 60C --0.157 61H - 0.144 62H - 0.141 63H - 0.188 65H - 0.100 66H - 0.088 70H - 0.095
MO 180: 44C --0.132 45H - 0.354 46H - 0.105 50C --0.160 51H - 0.118 52H - 0.119 53H - 0.279
MO 179: 14H - 0.205 16C --0.119 18H - 0.418 26C --0.089 27H - 0.188 29H - 0.092
MO 178: 46H - 0.120 57H - 0.110 68C --0.208 69H - 0.218 70H - 0.101 71H - 0.342 74H - 0.076
MO 177: 13C --0.077 15H - 0.190 32C --0.221 33H - 0.218 34H - 0.206 35H - 0.238 38H - 0.093 85H - 0.108
Localized MO's were stored in: 571_C2B3a_567_loc_output.gbw

5. Off-cycle intermediates and alternative isomers

The relative energies of complexes **10** and **12** and the alternative isomers **10’**, **12’**, **14’**, **14’’**, **20’**, and **20’’** were calculated. The Gibbs free energies [kcal mol^{-1}] are displayed in Figure S35 relative to complex **9** (0.0 kcal mol^{-1}).

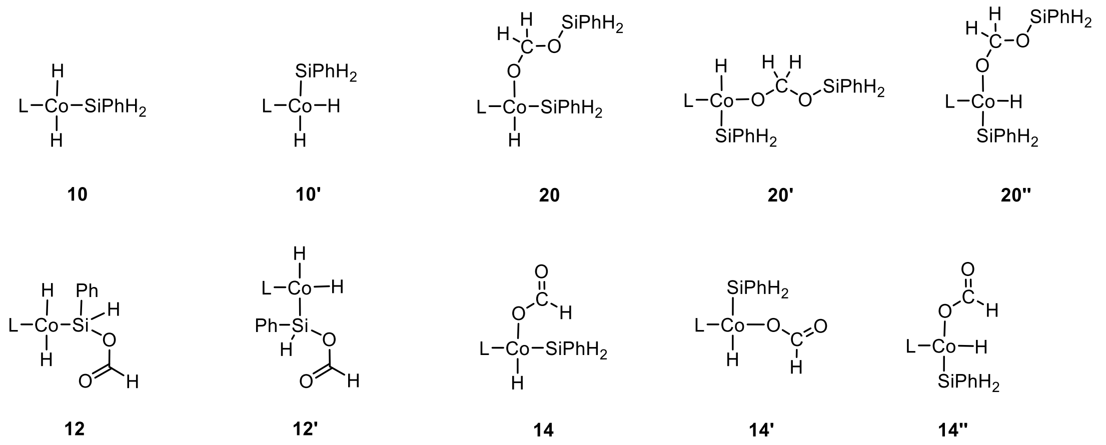

Scheme S1: Isomers of **10**, **12**, **14**, and **20**.

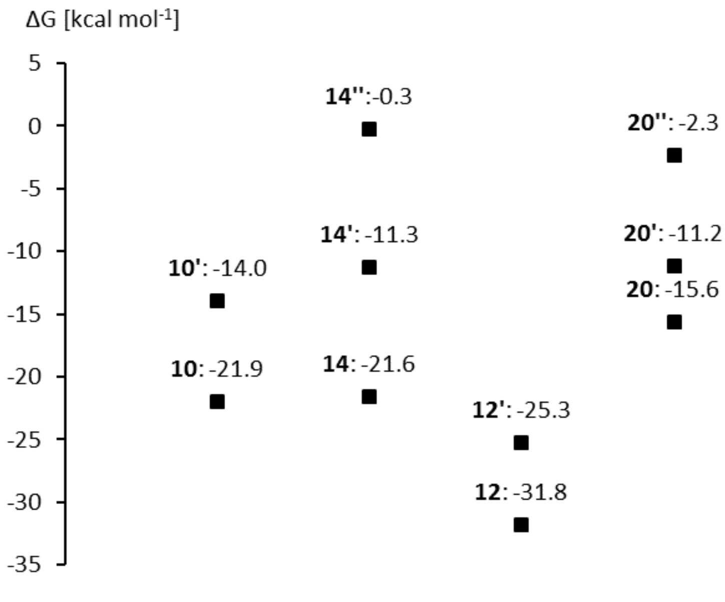

Figure S35: Gibbs free energies [kcal mol^{-1}] relative to **9** (0.0 kcal mol^{-1}) (B3LYP-D3BJ/def2-TZVP (selected atoms), def-SVP) for different isomers of the complexes **10**, **12**, **14**, and **20**.

6. Multiple Si–H activations

The computational mechanistic studies were performed based on the generation of **13** as the product of a single activation of a phenylsilane Si–H bond. This, therefore, constitutes a simplification of the reaction network since subsequent activation of the remaining Si–H bonds of the silylated products is, in theory, possible and since a complex product mixture is observed. Complex **33** forming after oxidative addition of phenylsilyl formate **13** to **11** has a relative Gibbs free energy of –30.6 kcal mol^{-1}. The product from the reductive elimination (**34**) has a relative energy of –10.6 kcal mol^{-1}. This indicates that this reaction pathway is competing with the oxidative addition of phenylsilane to **11**.

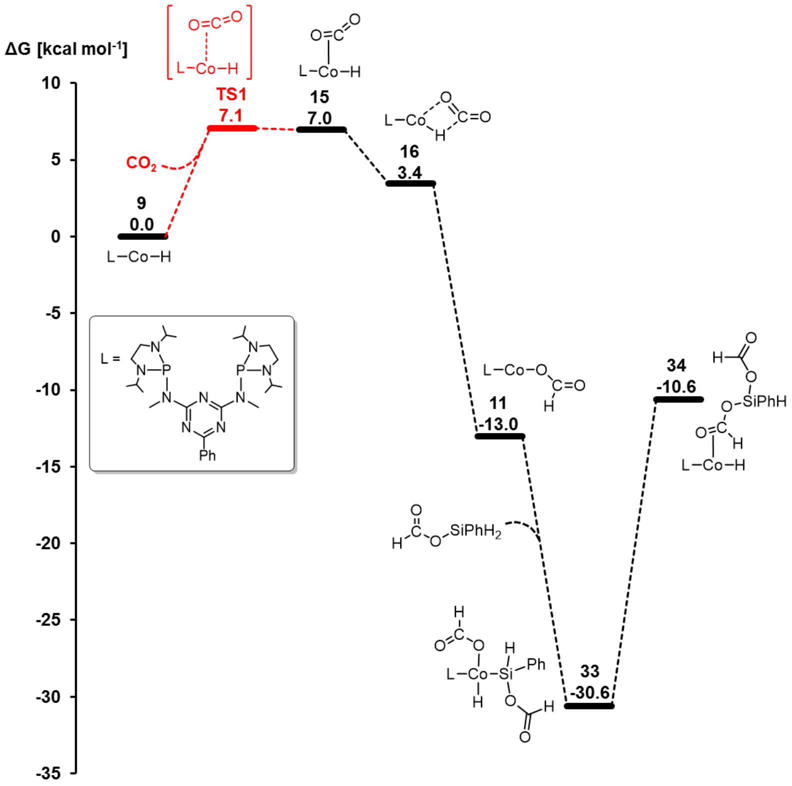

Figure S36: Relative Gibbs free energies [kcal mol^{-1}] (B3LYP-D3BJ/def2-TZVP (selected atoms), def-SVP) for the formation of a bis(formyl)silane.

7. Reductive elimination of formic acid

As an additional reaction pathway, we investigated the reductive elimination of formic acid from an alternative isomer **14’**, which is 10.3 kcal mol^{-1} higher in energy than **14**. Cobalt(I) silyl complex **32** has a relative energy of –0.5 kcal mol^{-1}. Due to the high energy of **14’** compared to **14** and the low thermodynamic driving force of the reaction of **9**, CO_2 and $PhSiH_3$ to **32** and formic acid, the reaction is considered less favored than the generation of silyl formate **13** from **14**.

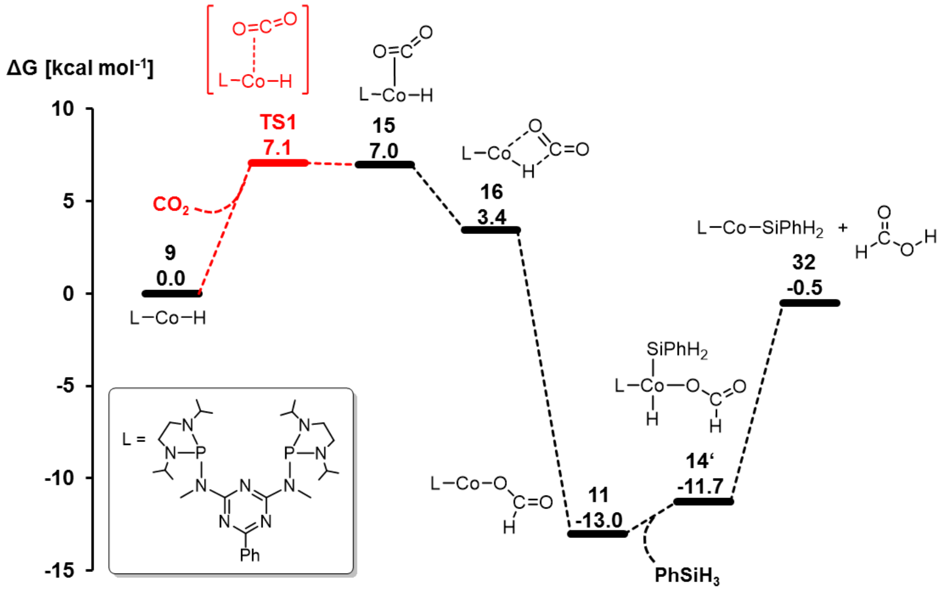

Figure S37: Relative Gibbs free energies [kcal mol^{-1}] (B3LYP-D3BJ/def2-TZVP (selected atoms), def-SVP) for the generation of **32** and formic acid from **9**, CO_2, and $PhSiH_3$.

8. Kinetic Simulations

The graphical representations of the product concentrations were obtained after microkinetic modeling.[14] Rate constants for each step were obtained with the Eyring equation using the energy difference between the connected intermediates and transition states. The free energy barriers of reactions that do not pass a transition state were neglected.

$$k = \frac{k_B T}{h} \exp\left(-\frac{\Delta G^{\#}}{RT}\right)$$

To simplify the reaction network, we only considered the insertion into the phenylsilane [Si]–H bond since under catalytic conditions, phenylsilane is in high excess compared to CO_2 (Figure 12). Furthermore, complexes **10** and **12** have a low relative energy (–21.9 kcal mol^{-1} and –31.8 kcal mol^{-1}) and may supposedly accumulate during the catalytic reaction. However, under operative conditions, **10** or **12** were not observed. Therefore, their formation was not included in the simulation.

Kinetic simulations were performed using the *Tenua* program (© 2021, Daniel Wachsstock). In the case of 1 bar pressure, the starting concentrations of phenylsilane, catalyst **9**, and CO_2 were set to 5M, 0.05M, and 1M, respectively. The CO_2 concentration was calculated from the ratio of phenylsilane and CO_2 in the reaction, and thus, the transfer from the gas phase into the liquid phase was not considered. In the case of 40 bar pressure, the CO_2 concentration was increased to 40M. The time span and the simulation accuracy ε were varied between 50 – 100 s and $\varepsilon = 1 \times 10^{-6}$ and $\varepsilon = 1 \times 10^{-5}$.

9. XYZ Coordinates of Optimized Geometries

9

27	15.048714000	11.769397000	7.404234000
1	13.537255000	11.697509000	7.186816000
15	15.364078000	13.001834000	5.721588000
7	17.128637000	13.234611000	5.832646000
6	17.751758000	12.590999000	6.855606000
7	19.080972000	12.660844000	7.003843000
6	19.600787000	11.976899000	8.032669000
7	18.895611000	11.178816000	8.848329000
6	17.578351000	11.133952000	8.631350000
7	16.946943000	11.883357000	7.689945000
7	16.783700000	10.278626000	9.329369000
15	15.197777000	10.005926000	8.561699000
7	14.322410000	9.320422000	9.821771000
6	13.969987000	7.921888000	9.586482000
1	12.964548000	7.827361000	9.133966000
1	13.975002000	7.344499000	10.524925000
6	15.025922000	7.392344000	8.613735000
1	15.914127000	7.023829000	9.164849000
1	14.627605000	6.552379000	8.023457000
7	15.332883000	8.532662000	7.761480000
6	16.193429000	8.416527000	6.584171000
1	16.272428000	9.448089000	6.204919000
6	17.615610000	7.938552000	6.905394000
1	18.108158000	8.603915000	7.628645000
1	18.226731000	7.930904000	5.989036000
1	17.620755000	6.917280000	7.322066000
6	15.528962000	7.575608000	5.491917000
1	15.414654000	6.523617000	5.801396000
1	16.136921000	7.589248000	4.573793000
1	14.531285000	7.975194000	5.255636000
6	13.468862000	10.174550000	10.660313000
1	13.999386000	11.139897000	10.727431000
6	13.353491000	9.608906000	12.077856000
1	12.762778000	8.678084000	12.092751000
1	12.844067000	10.329698000	12.735519000
1	14.346654000	9.391429000	12.501708000
6	12.093190000	10.437953000	10.034004000
1	12.208521000	10.908505000	9.045960000
1	11.498576000	11.109115000	10.673846000
1	11.523364000	9.502234000	9.913601000
7	14.877259000	14.546651000	5.245929000
6	14.298199000	14.600937000	3.908147000
1	14.587596000	15.527078000	3.384968000
1	13.192406000	14.563415000	3.941044000
6	14.843930000	13.372906000	3.171167000

1	14.130515000	13.026322000	2.408383000
1	15.791446000	13.610785000	2.649047000
7	15.020774000	12.373859000	4.211018000
6	15.232467000	10.962006000	3.912585000
1	15.260118000	10.480621000	4.902499000
6	14.041136000	10.368871000	3.159812000
1	13.114772000	10.555353000	3.725332000
1	14.163201000	9.281107000	3.041060000
1	13.929912000	10.802529000	2.152243000
6	16.578518000	10.707549000	3.224226000
1	16.639390000	11.223130000	2.251359000
1	16.732216000	9.631650000	3.043536000
1	17.404544000	11.071277000	3.855248000
6	14.504181000	15.541515000	6.256935000
1	15.148634000	15.329431000	7.127369000
6	14.819484000	16.960120000	5.776016000
1	15.861581000	17.037782000	5.427269000
1	14.663344000	17.682289000	6.592452000
1	14.158194000	17.256762000	4.945780000
6	13.045027000	15.414525000	6.716077000
1	12.346305000	15.652664000	5.897356000
1	12.838008000	16.115889000	7.539578000
1	12.839350000	14.392472000	7.070356000
6	17.916746000	14.012631000	4.890988000
1	18.632379000	14.654894000	5.424220000
1	17.234873000	14.646482000	4.311957000
1	18.486867000	13.364892000	4.203937000
6	21.056208000	12.097123000	8.285661000
6	21.852153000	12.936494000	7.485761000
1	21.378059000	13.479009000	6.667081000
6	23.215618000	13.072560000	7.748412000
1	23.826300000	13.730958000	7.125514000
6	23.802125000	12.369714000	8.808194000
1	24.867656000	12.489121000	9.022551000
6	23.019513000	11.519851000	9.599377000
1	23.476703000	10.963237000	10.421655000
6	21.655580000	11.383950000	9.340318000
1	21.029272000	10.732140000	9.950038000
6	17.368087000	9.423049000	10.347162000
1	18.009965000	8.638334000	9.911834000
1	16.550163000	8.951431000	10.906093000
1	17.984730000	10.019019000	11.035105000

15

27	-1.228206000	0.738497000	0.523469000
1	-2.717073000	0.840751000	0.262887000
15	-0.896484000	1.708857000	-1.288084000
7	0.871416000	1.947861000	-1.185824000
6	1.522837000	1.259547000	-0.213295000

7	2.859326000	1.250743000	-0.129813000
6	3.382049000	0.504484000	0.855030000
7	2.676226000	-0.312634000	1.654597000
6	1.349808000	-0.285585000	1.491356000
7	0.739887000	0.573406000	0.648553000
7	0.533023000	-1.170038000	2.128291000
15	-1.016020000	-1.483473000	1.294292000
7	-1.953012000	-2.194352000	2.483959000
6	-2.319585000	-3.574704000	2.165138000
1	-3.280474000	-3.626211000	1.619120000
1	-2.421986000	-4.177105000	3.080457000
6	-1.186752000	-4.097244000	1.286464000
1	-0.355280000	-4.480251000	1.908836000
1	-1.533496000	-4.919493000	0.643096000
7	-0.787031000	-2.941175000	0.484998000
6	0.221983000	-3.071400000	-0.573140000
1	0.331708000	-2.059221000	-0.990997000
6	1.608558000	-3.501208000	-0.073936000
1	2.033874000	-2.772320000	0.627833000
1	2.301139000	-3.580569000	-0.926271000
1	1.577652000	-4.484818000	0.422575000
6	-0.281661000	-3.987854000	-1.691273000
1	-0.351024000	-5.033084000	-1.349763000
1	0.407799000	-3.966419000	-2.548821000
1	-1.277681000	-3.671590000	-2.032921000
6	-2.802524000	-1.383179000	3.379018000
1	-2.221249000	-0.476135000	3.591769000
6	-3.040025000	-2.110513000	4.705432000
1	-3.705702000	-2.981085000	4.585471000
1	-3.521902000	-1.427676000	5.421309000
1	-2.092813000	-2.460345000	5.146807000
6	-4.116233000	-0.953388000	2.716086000
1	-3.911530000	-0.347239000	1.821493000
1	-4.715145000	-0.344008000	3.411527000
1	-4.724408000	-1.824988000	2.421531000
7	-1.427704000	3.169599000	-1.903315000
6	-1.974807000	3.072856000	-3.256350000
1	-1.686097000	3.949730000	-3.856502000
1	-3.079217000	3.022342000	-3.237520000
6	-1.392722000	1.793973000	-3.857646000
1	-2.082356000	1.354021000	-4.592932000
1	-0.438288000	1.998280000	-4.379735000
7	-1.216463000	0.909625000	-2.717279000
6	-0.964224000	-0.515660000	-2.880138000
1	-0.916825000	-0.904265000	-1.851927000
6	-2.147531000	-1.206669000	-3.558938000
1	-3.070879000	-1.002189000	-2.995118000
1	-1.992826000	-2.295169000	-3.589234000
1	-2.286819000	-0.861383000	-4.596216000
6	0.381738000	-0.788460000	-3.562217000

1	0.405085000	-0.374870000	-4.583720000
1	0.578044000	-1.868688000	-3.635654000
1	1.202663000	-0.326965000	-2.991251000
6	-1.841620000	4.287783000	-1.038404000
1	-1.216943000	4.213246000	-0.140113000
6	-1.561001000	5.636752000	-1.703183000
1	-0.514295000	5.707981000	-2.040009000
1	-1.751837000	6.447537000	-0.983020000
1	-2.215009000	5.812003000	-2.572992000
6	-3.304544000	4.174816000	-0.592002000
1	-4.002509000	4.326864000	-1.432347000
1	-3.528681000	4.939187000	0.167921000
1	-3.494772000	3.187800000	-0.145031000
6	1.638976000	2.727203000	-2.146486000
1	2.369205000	3.360908000	-1.623587000
1	0.944131000	3.367088000	-2.702799000
1	2.185631000	2.078841000	-2.850905000
6	4.848794000	0.567032000	1.063301000
6	5.645937000	1.391939000	0.249865000
1	5.164613000	1.964944000	-0.543066000
6	7.021355000	1.474784000	0.467452000
1	7.634586000	2.121148000	-0.165192000
6	7.615480000	0.734515000	1.496903000
1	8.691338000	0.811427000	1.675949000
6	6.830289000	-0.098656000	2.302902000
1	7.294882000	-0.681193000	3.102442000
6	5.454434000	-0.183013000	2.087594000
1	4.826049000	-0.821163000	2.709332000
6	1.077589000	-2.041922000	3.158342000
1	1.738497000	-2.820164000	2.743927000
1	0.237494000	-2.520001000	3.677066000
1	1.658296000	-1.448117000	3.877843000
6	-1.125832000	2.242165000	1.791427000
8	-1.428697000	1.395285000	2.646869000
8	-0.797183000	3.407430000	1.763374000

16

27	14.992855000	11.843797000	7.525957000
1	13.399156000	11.612799000	7.333146000
15	15.297806000	12.988305000	5.745773000
7	17.059570000	13.192900000	5.831465000
6	17.685146000	12.586279000	6.869105000
7	19.014334000	12.656677000	7.007065000
6	19.541617000	11.995004000	8.045800000
7	18.843320000	11.200269000	8.869939000
6	17.526646000	11.147357000	8.662577000
7	16.886362000	11.904715000	7.731353000
7	16.746495000	10.270611000	9.342141000

15	15.193246000	9.955988000	8.536514000
7	14.279139000	9.235149000	9.724675000
6	14.030266000	7.812518000	9.502960000
1	13.036161000	7.650779000	9.048265000
1	14.067740000	7.245323000	10.447254000
6	15.126622000	7.345675000	8.540461000
1	16.020975000	7.008542000	9.099981000
1	14.776250000	6.499370000	7.930231000
7	15.403628000	8.511271000	7.705858000
6	16.307454000	8.441517000	6.555042000
1	16.344650000	9.474650000	6.173793000
6	17.741713000	8.033351000	6.917719000
1	18.181129000	8.711805000	7.661810000
1	18.379533000	8.067260000	6.020598000
1	17.784110000	7.008600000	7.322949000
6	15.725448000	7.561766000	5.446464000
1	15.664445000	6.506642000	5.759213000
1	16.362467000	7.605615000	4.549854000
1	14.714501000	7.899770000	5.173543000
6	13.395026000	10.045033000	10.576126000
1	13.695504000	11.089663000	10.399016000
6	13.617967000	9.735878000	12.057258000
1	13.358704000	8.690057000	12.293569000
1	12.984394000	10.382444000	12.683339000
1	14.667816000	9.909073000	12.339895000
6	11.922311000	9.912302000	10.178871000
1	11.784483000	10.080223000	9.099817000
1	11.318371000	10.658326000	10.715818000
1	11.523603000	8.915783000	10.431009000
7	14.792388000	14.513415000	5.286570000
6	14.226776000	14.597063000	3.945138000
1	14.571394000	15.503852000	3.421070000
1	13.122739000	14.629543000	3.978083000
6	14.705106000	13.334216000	3.211291000
1	13.947579000	12.991029000	2.491418000
1	15.633654000	13.531077000	2.641778000
7	14.909181000	12.344082000	4.259572000
6	15.098327000	10.925598000	3.973052000
1	15.139516000	10.455200000	4.967668000
6	13.885970000	10.338757000	3.250390000
1	12.968445000	10.569942000	3.813777000
1	13.978979000	9.244607000	3.172290000
1	13.778117000	10.737583000	2.228332000
6	16.429745000	10.648614000	3.265739000
1	16.484862000	11.160436000	2.290837000
1	16.563549000	9.570584000	3.086110000
1	17.270904000	10.999815000	3.883874000
6	14.604564000	15.587396000	6.267062000
1	15.028917000	15.199708000	7.209059000
6	15.393431000	16.839951000	5.870134000

1	16.455462000	16.602741000	5.701544000
1	15.326392000	17.605212000	6.659062000
1	14.989668000	17.283775000	4.945015000
6	13.125304000	15.902902000	6.515489000
1	12.653860000	16.340411000	5.619963000
1	13.022476000	16.635054000	7.331208000
1	12.566390000	14.999321000	6.796661000
6	17.841841000	13.931899000	4.853041000
1	18.540500000	14.615820000	5.355747000
1	17.155197000	14.519670000	4.233166000
1	18.426330000	13.255136000	4.208865000
6	20.995435000	12.129349000	8.295800000
6	21.782384000	12.969253000	7.487887000
1	21.303664000	13.502052000	6.665553000
6	23.144766000	13.118443000	7.748143000
1	23.749626000	13.777140000	7.120146000
6	23.737317000	12.427917000	8.812625000
1	24.801967000	12.557803000	9.024978000
6	22.963383000	11.577341000	9.611458000
1	23.426399000	11.030735000	10.436996000
6	21.600490000	11.428366000	9.355287000
1	20.981041000	10.775642000	9.970965000
6	17.335552000	9.409802000	10.354038000
1	18.001042000	8.648288000	9.914755000
1	16.521719000	8.910817000	10.894140000
1	17.925620000	10.010237000	11.060346000
6	12.885209000	12.578181000	7.963860000
8	11.765488000	12.746074000	7.510471000
8	13.542757000	13.064686000	8.891264000

11

27	14.912104000	11.644836000	7.779233000
1	12.871853000	11.381065000	5.649298000
15	15.244756000	12.986099000	6.119561000
7	16.870799000	13.539349000	6.500588000
6	17.544532000	12.744112000	7.376279000
7	18.866457000	12.864732000	7.504159000
6	19.466500000	12.003836000	8.336492000
7	18.828085000	11.032464000	9.001124000
6	17.503492000	10.963708000	8.833349000
7	16.796961000	11.827502000	8.051114000
7	16.789702000	9.979537000	9.443753000
15	15.095141000	9.857850000	8.981320000
7	14.362498000	9.337014000	10.396391000
6	13.767403000	8.005749000	10.285754000
1	12.691967000	8.064787000	10.035622000
1	13.874853000	7.446370000	11.228800000
6	14.524149000	7.309774000	9.152420000

1	15.423081000	6.788681000	9.535874000
1	13.890671000	6.557281000	8.660790000
7	14.858229000	8.387112000	8.232285000
6	15.271166000	8.147235000	6.853005000
1	15.376912000	9.154540000	6.421518000
6	16.634958000	7.455354000	6.758508000
1	17.405325000	8.045842000	7.278825000
1	16.943023000	7.342606000	5.706590000
1	16.611362000	6.450390000	7.211887000
6	14.175123000	7.427930000	6.065294000
1	13.995987000	6.409917000	6.448093000
1	14.460721000	7.337315000	5.006326000
1	13.232478000	7.993239000	6.126617000
6	13.830994000	10.315347000	11.356286000
1	14.460648000	11.213366000	11.233681000
6	14.000982000	9.814869000	12.792363000
1	13.359829000	8.939443000	12.984519000
1	13.706020000	10.599336000	13.506444000
1	15.044879000	9.526655000	12.993578000
6	12.380822000	10.716908000	11.058973000
1	12.276490000	11.050161000	10.014790000
1	12.069779000	11.537970000	11.723639000
1	11.689804000	9.875808000	11.232928000
7	14.503339000	14.366410000	5.538945000
6	14.162782000	14.304394000	4.117689000
1	14.329614000	15.277054000	3.628426000
1	13.104213000	14.021177000	3.969694000
6	15.085898000	13.241326000	3.515776000
1	14.610871000	12.751331000	2.652097000
1	16.030723000	13.697822000	3.161469000
7	15.306173000	12.291344000	4.596839000
6	15.997367000	11.020642000	4.377114000
1	16.104221000	10.587878000	5.384996000
6	15.134149000	10.062611000	3.554611000
1	14.169217000	9.891149000	4.054570000
1	15.640691000	9.092212000	3.433477000
1	14.933061000	10.462288000	2.547170000
6	17.405254000	11.195576000	3.797570000
1	17.380832000	11.658024000	2.797019000
1	17.902076000	10.217635000	3.698935000
1	18.026066000	11.826281000	4.451587000
6	13.831680000	15.310060000	6.443621000
1	14.372135000	15.232293000	7.402372000
6	13.983036000	16.747417000	5.940596000
1	15.036838000	16.988610000	5.730042000
1	13.606715000	17.454493000	6.695124000
1	13.400581000	16.910336000	5.019367000
6	12.364501000	14.948460000	6.695846000
1	11.782596000	14.977936000	5.760581000
1	11.905767000	15.662864000	7.396449000

1	12.279913000	13.941994000	7.129762000
6	17.602242000	14.519149000	5.712548000
1	18.224263000	15.146151000	6.366409000
1	16.872102000	15.151783000	5.193468000
1	18.261854000	14.038507000	4.971835000
6	20.932477000	12.127311000	8.508182000
6	21.644633000	13.120093000	7.809964000
1	21.095037000	13.784756000	7.142978000
6	23.023589000	13.246392000	7.976112000
1	23.570132000	14.019914000	7.430736000
6	23.707707000	12.382195000	8.839662000
1	24.786824000	12.489669000	8.978291000
6	23.007929000	11.384779000	9.529995000
1	23.542746000	10.706027000	10.198934000
6	21.628383000	11.255943000	9.365165000
1	21.066808000	10.486409000	9.895752000
6	17.469941000	9.015294000	10.294623000
1	18.064092000	8.299172000	9.703427000
1	16.710613000	8.473451000	10.871202000
1	18.147965000	9.531168000	10.988452000
6	12.315889000	11.275150000	6.603517000
8	13.016023000	11.444075000	7.666526000
8	11.116031000	11.014797000	6.574139000

14

27	14.781418000	11.474405000	7.322816000
1	16.064988000	13.025962000	9.591926000
15	15.025309000	12.760408000	5.576839000
7	16.748829000	13.152824000	5.782025000
6	17.457188000	12.437521000	6.692408000
7	18.793543000	12.516247000	6.743913000
6	19.395162000	11.743822000	7.659274000
7	18.756832000	10.901216000	8.486415000
6	17.425185000	10.871533000	8.375452000
7	16.739953000	11.643443000	7.511324000
7	16.680104000	10.025961000	9.142733000
15	14.976727000	9.883013000	8.752443000
7	14.238247000	9.683344000	10.232145000
6	13.394180000	8.491120000	10.272868000
1	12.363371000	8.715453000	9.947300000
1	13.347551000	8.084116000	11.293637000
6	14.058437000	7.491642000	9.320549000
1	14.774311000	6.851642000	9.869356000
1	13.306888000	6.832537000	8.857192000
7	14.730563000	8.301600000	8.312849000
6	15.336822000	7.697277000	7.123036000
1	16.033816000	8.449582000	6.719993000
6	16.156716000	6.454561000	7.485684000

1	16.900186000	6.682695000	8.264360000
1	16.690865000	6.077653000	6.600104000
1	15.511040000	5.641544000	7.854835000
6	14.293095000	7.406208000	6.042184000
1	13.509837000	6.729469000	6.419972000
1	14.760010000	6.927831000	5.166580000
1	13.812036000	8.337106000	5.712349000
6	14.050130000	10.782561000	11.189841000
1	14.830224000	11.519423000	10.947207000
6	14.310133000	10.303331000	12.621256000
1	13.541423000	9.587596000	12.953338000
1	14.278867000	11.160540000	13.311052000
1	15.293887000	9.815410000	12.706644000
6	12.688558000	11.467448000	11.059576000
1	12.531868000	11.835335000	10.036591000
1	12.635702000	12.333341000	11.736272000
1	11.871667000	10.777138000	11.326398000
7	14.367796000	14.244963000	5.235962000
6	13.943396000	14.427108000	3.852138000
1	14.319966000	15.380252000	3.448596000
1	12.842956000	14.451032000	3.773207000
6	14.522798000	13.247250000	3.053107000
1	13.782027000	12.879325000	2.328767000
1	15.418450000	13.560618000	2.484828000
7	14.840221000	12.202555000	4.021963000
6	15.324511000	10.890459000	3.573276000
1	15.210359000	10.226501000	4.444419000
6	14.455225000	10.325438000	2.450084000
1	13.393483000	10.329419000	2.732782000
1	14.755565000	9.287132000	2.240817000
1	14.578250000	10.897809000	1.516443000
6	16.807925000	10.914666000	3.175632000
1	16.994719000	11.654911000	2.380661000
1	17.121159000	9.930923000	2.791375000
1	17.450729000	11.164387000	4.032345000
6	14.036460000	15.234270000	6.274493000
1	14.632608000	14.953588000	7.153865000
6	14.449871000	16.645519000	5.849112000
1	15.499478000	16.673874000	5.515426000
1	14.337780000	17.334649000	6.699793000
1	13.818018000	17.027882000	5.031030000
6	12.564133000	15.171727000	6.686763000
1	11.897083000	15.415476000	5.843263000
1	12.364457000	15.894558000	7.492326000
1	12.317362000	14.172881000	7.070305000
6	17.452924000	14.062052000	4.888614000
1	18.111950000	14.726225000	5.465097000
1	16.713640000	14.673561000	4.359932000
1	18.066741000	13.516088000	4.155522000
6	20.873876000	11.810475000	7.753002000

6	21.603547000	12.640683000	6.883305000
1	21.059366000	13.229182000	6.144429000
6	22.994317000	12.703530000	6.972612000
1	23.555920000	13.349960000	6.293789000
6	23.670696000	11.939680000	7.931547000
1	24.759997000	11.995311000	8.005991000
6	22.951764000	11.107889000	8.798404000
1	23.480406000	10.510257000	9.545064000
6	21.560998000	11.042129000	8.709869000
1	20.984741000	10.401506000	9.377623000
6	17.345171000	9.125355000	10.073441000
1	18.021332000	8.438967000	9.540918000
1	16.577974000	8.550171000	10.605096000
1	17.935653000	9.695476000	10.805192000
1	14.934709000	10.373366000	6.374613000
14	12.586430000	10.970706000	7.089421000
1	12.201598000	9.656161000	7.697474000
1	11.743098000	11.973480000	7.812754000
6	10.980269000	12.004455000	3.373823000
6	10.581281000	10.780410000	2.823754000
6	11.584145000	12.038251000	4.631623000
1	10.101349000	10.748145000	1.842163000
1	11.873447000	12.997710000	5.061101000
6	10.785612000	9.600546000	3.545343000
6	11.808750000	10.864072000	5.373911000
1	10.459488000	8.643732000	3.127343000
6	11.394421000	9.646234000	4.805387000
1	11.531588000	8.716895000	5.364501000
1	10.809085000	12.933764000	2.822947000
6	15.063954000	13.490932000	9.464504000
8	14.748500000	14.416394000	10.209685000
8	14.334831000	12.999494000	8.539531000

17

27	14.717238000	11.565917000	7.630970000
1	14.949073000	14.072219000	8.622725000
15	14.955941000	12.313860000	5.697876000
7	16.736107000	12.592716000	5.676781000
6	17.407758000	12.312096000	6.816197000
7	18.736152000	12.476051000	6.914586000
6	19.290073000	12.094436000	8.073437000
7	18.648880000	11.416702000	9.037624000
6	17.337764000	11.227752000	8.838438000
7	16.657595000	11.833460000	7.836526000
7	16.619896000	10.334644000	9.559074000
15	15.222727000	9.675643000	8.602865000
7	14.371765000	8.794511000	9.734637000
6	14.495435000	7.347508000	9.575585000

1	13.640927000	6.933891000	9.008677000
1	14.530653000	6.837661000	10.551692000
6	15.791350000	7.136444000	8.793419000
1	16.657062000	7.091380000	9.481239000
1	15.768658000	6.192848000	8.228205000
7	15.861878000	8.285127000	7.894063000
6	16.903554000	8.351282000	6.864344000
1	16.759318000	9.327446000	6.370943000
6	18.335519000	8.292659000	7.418079000
1	18.550385000	9.099716000	8.129616000
1	19.058774000	8.375965000	6.591768000
1	18.524057000	7.331908000	7.925172000
6	16.697073000	7.256187000	5.814316000
1	16.949679000	6.263271000	6.221004000
1	17.346941000	7.432743000	4.944290000
1	15.652617000	7.234461000	5.471191000
6	13.243286000	9.381917000	10.470223000
1	13.344867000	10.468496000	10.337388000
6	13.333601000	9.071021000	11.966971000
1	13.186068000	7.996794000	12.169999000
1	12.552126000	9.618195000	12.516263000
1	14.310171000	9.372983000	12.376257000
6	11.884142000	8.970516000	9.896079000
1	11.837341000	9.191144000	8.818701000
1	11.082560000	9.535820000	10.395792000
1	11.681464000	7.897494000	10.052159000
7	14.421641000	13.732308000	4.972024000
6	14.002269000	13.629873000	3.582524000
1	14.509527000	14.380843000	2.950851000
1	12.917008000	13.803758000	3.489379000
6	14.362544000	12.208731000	3.119410000
1	13.536452000	11.772420000	2.536524000
1	15.249523000	12.219516000	2.459085000
7	14.599230000	11.431599000	4.326202000
6	14.502596000	9.975211000	4.298521000
1	14.855813000	9.640196000	5.285395000
6	13.050132000	9.507558000	4.155284000
1	12.450191000	9.879699000	4.997361000
1	12.990458000	8.407315000	4.149179000
1	12.597265000	9.869857000	3.217671000
6	15.408121000	9.385652000	3.214242000
1	15.071599000	9.690736000	2.209634000
1	15.398180000	8.285993000	3.246944000
1	16.446697000	9.729846000	3.345089000
6	14.429047000	15.032479000	5.640972000
1	14.453757000	14.813771000	6.716264000
6	15.674229000	15.864917000	5.304595000
1	16.590240000	15.341264000	5.612973000
1	15.642415000	16.832090000	5.831038000
1	15.741423000	16.073490000	4.223659000

6	13.138046000	15.808884000	5.371527000
1	13.079986000	16.152868000	4.325707000
1	13.095696000	16.699915000	6.016065000
1	12.259787000	15.185807000	5.583562000
6	17.456588000	12.995498000	4.480977000
1	18.300563000	13.648847000	4.739600000
1	16.775329000	13.542519000	3.819621000
1	17.853187000	12.121476000	3.937244000
6	20.726036000	12.399288000	8.286134000
6	21.422412000	13.215959000	7.377765000
1	20.888710000	13.607166000	6.510663000
6	22.765848000	13.524428000	7.595731000
1	23.298009000	14.167036000	6.889621000
6	23.430605000	13.011907000	8.716657000
1	24.480341000	13.262360000	8.892540000
6	22.748204000	12.183067000	9.615337000
1	23.269712000	11.771870000	10.483444000
6	21.402810000	11.879707000	9.403995000
1	20.853217000	11.239203000	10.094890000
6	17.224291000	9.678214000	10.705011000
1	18.017709000	8.967818000	10.417596000
1	16.439051000	9.134446000	11.245791000
1	17.673385000	10.427905000	11.373258000
1	13.283929000	11.221881000	7.330033000
14	11.431012000	13.187047000	8.494539000
1	11.470162000	11.797916000	8.981128000
1	10.591432000	14.007818000	9.405460000
6	9.244127000	14.580763000	5.256399000
6	9.688836000	13.788230000	4.193519000
6	9.744752000	14.361620000	6.544257000
1	9.300211000	13.962872000	3.186412000
1	9.394350000	14.990380000	7.368143000
6	10.628256000	12.774785000	4.423841000
6	10.703646000	13.360350000	6.789649000
1	10.970655000	12.146108000	3.598300000
6	11.136009000	12.568131000	5.708635000
1	11.896314000	11.804689000	5.882191000
1	8.508650000	15.370074000	5.079856000
6	14.141539000	13.354564000	8.777067000
8	14.259966000	12.397452000	9.573492000
8	12.927933000	13.928847000	8.503906000

18

27	14.362341000	11.261441000	7.430862000
1	12.866409000	10.858138000	6.954827000
15	14.619419000	12.515359000	5.734540000
7	16.261236000	13.128069000	6.068882000
6	16.880124000	12.622981000	7.158041000

7	18.138530000	12.965641000	7.459040000
6	18.685950000	12.355792000	8.516071000
7	18.096073000	11.371606000	9.209853000
6	16.852131000	11.052812000	8.841589000
7	16.155946000	11.740164000	7.897490000
7	16.236906000	9.951398000	9.339763000
15	14.951308000	9.321268000	8.258392000
7	14.130588000	8.253868000	9.247509000
6	14.351676000	6.845224000	8.926807000
1	13.526814000	6.443759000	8.309562000
1	14.415306000	6.231774000	9.840380000
6	15.664364000	6.802541000	8.136423000
1	16.529727000	6.690646000	8.816833000
1	15.682224000	5.948336000	7.442671000
7	15.688859000	8.067772000	7.409068000
6	16.710789000	8.359264000	6.400554000
1	16.460493000	9.370482000	6.041155000
6	18.144604000	8.399846000	6.947891000
1	18.257288000	9.121144000	7.767959000
1	18.841300000	8.699621000	6.149174000
1	18.464488000	7.410524000	7.315190000
6	16.600347000	7.398845000	5.215158000
1	16.877688000	6.371984000	5.505256000
1	17.281053000	7.706564000	4.407186000
1	15.573167000	7.383359000	4.819757000
6	13.014808000	8.682831000	10.099745000
1	12.962472000	9.776781000	9.981196000
6	13.282426000	8.385417000	11.579095000
1	13.361177000	7.301053000	11.764982000
1	12.457848000	8.767637000	12.201057000
1	14.211761000	8.867268000	11.917941000
6	11.674607000	8.085839000	9.653018000
1	11.528629000	8.199045000	8.567778000
1	10.846652000	8.599032000	10.165907000
1	11.606169000	7.012541000	9.897444000
7	13.821689000	13.915001000	5.289035000
6	13.624743000	14.110254000	3.859429000
1	13.944160000	15.116749000	3.535429000
1	12.558482000	14.003284000	3.590939000
6	14.464250000	13.028633000	3.158088000
1	13.940156000	12.649667000	2.268288000
1	15.431660000	13.439026000	2.811488000
7	14.636076000	11.970148000	4.145255000
6	15.200409000	10.671984000	3.780544000
1	15.063279000	10.051967000	4.681095000
6	14.414638000	10.015978000	2.644434000
1	13.342001000	9.977796000	2.890311000
1	14.773506000	8.987623000	2.481689000
1	14.537581000	10.562099000	1.694895000
6	16.700781000	10.742440000	3.465990000

1	16.902359000	11.438706000	2.635413000
1	17.087514000	9.754675000	3.171332000
1	17.271189000	11.085282000	4.342232000
6	13.302773000	14.864430000	6.275828000
1	13.230188000	14.299661000	7.219842000
6	14.258505000	16.043885000	6.502716000
1	15.225223000	15.696101000	6.896368000
1	13.830626000	16.756103000	7.226344000
1	14.444360000	16.592225000	5.563702000
6	11.899337000	15.344531000	5.902456000
1	11.917657000	15.998347000	5.015348000
1	11.462709000	15.922372000	6.729292000
1	11.233308000	14.497163000	5.693198000
6	16.954426000	14.060494000	5.194437000
1	17.641638000	14.686568000	5.777141000
1	16.215915000	14.705322000	4.704738000
1	17.536846000	13.531271000	4.422532000
6	20.052647000	12.761087000	8.920941000
6	20.729812000	13.776887000	8.223250000
1	20.232754000	14.250682000	7.375989000
6	22.009522000	14.168404000	8.617117000
1	22.527717000	14.960826000	8.071088000
6	22.630983000	13.545272000	9.706337000
1	23.628832000	13.861812000	10.022157000
6	21.968918000	12.521850000	10.395507000
1	22.455758000	12.025824000	11.239087000
6	20.688415000	12.129703000	10.004841000
1	20.158263000	11.334540000	10.530217000
6	16.922930000	9.146693000	10.337978000
1	17.801372000	8.628032000	9.919638000
1	16.216764000	8.403367000	10.726835000
1	17.269228000	9.784937000	11.162676000
6	6.873988000	15.036552000	6.032296000
6	7.442180000	13.843330000	5.572992000
6	7.398335000	15.665454000	7.166829000
1	7.033926000	13.350505000	4.686300000
1	6.957882000	16.597814000	7.530730000
6	8.527778000	13.277559000	6.249903000
6	8.489009000	15.100188000	7.833873000
1	8.972409000	12.349761000	5.879864000
1	8.904749000	15.611853000	8.708083000
6	9.066905000	13.896702000	7.391740000
1	6.027447000	15.480731000	5.501818000
14	10.497275000	13.174339000	8.346697000
1	11.300023000	14.303737000	8.871911000
1	9.996044000	12.379042000	9.500179000
8	11.307339000	12.169660000	7.301866000
6	12.235736000	11.285533000	7.945721000
1	11.689583000	10.362104000	8.188091000
8	12.925083000	11.824098000	8.911533000

19

27	15.001483000	11.249306000	6.620701000
1	13.355023000	10.880081000	4.058554000
15	15.423443000	12.900131000	5.319429000
7	17.051930000	13.297482000	5.829213000
6	17.604100000	12.463122000	6.753613000
7	18.861647000	12.647544000	7.154039000
6	19.319266000	11.810494000	8.094548000
7	18.596296000	10.818549000	8.631192000
6	17.344218000	10.690311000	8.191140000
7	16.791009000	11.479966000	7.228147000
7	16.526657000	9.741669000	8.727728000
15	14.887702000	9.726213000	8.139986000
7	13.974445000	9.780307000	9.546092000
6	12.959480000	8.733506000	9.577116000
1	12.033064000	9.050810000	9.068019000
1	12.698669000	8.460826000	10.612315000
6	13.595209000	7.545655000	8.840394000
1	14.150065000	6.899230000	9.548293000
1	12.819449000	6.929462000	8.358481000
7	14.481527000	8.142003000	7.850193000
6	15.105399000	7.385440000	6.764377000
1	15.939360000	8.015754000	6.409344000
6	15.705434000	6.070834000	7.274756000
1	16.413446000	6.254320000	8.097580000
1	16.243317000	5.551834000	6.466192000
1	14.921583000	5.388846000	7.643985000
6	14.154186000	7.181847000	5.583427000
1	13.277487000	6.582886000	5.877798000
1	14.664172000	6.655807000	4.760231000
1	13.795536000	8.155862000	5.219867000
6	13.806340000	11.055066000	10.250379000
1	14.705077000	11.642844000	9.994045000
6	13.819083000	10.833891000	11.765290000
1	12.920994000	10.288505000	12.098209000
1	13.833749000	11.798649000	12.296241000
1	14.702797000	10.251498000	12.068868000
6	12.584459000	11.849507000	9.782645000
1	12.612025000	11.994998000	8.693785000
1	12.559085000	12.837900000	10.268674000
1	11.645023000	11.331989000	10.027919000
7	14.666455000	14.395241000	5.349389000
6	14.187645000	14.840325000	4.047231000
1	14.287133000	15.932449000	3.937102000
1	13.124263000	14.574172000	3.891049000
6	15.068641000	14.116311000	3.021554000
1	14.520174000	13.967545000	2.079776000

1	15.972890000	14.712720000	2.789798000
7	15.390191000	12.839269000	3.648141000
6	16.017245000	11.727081000	2.927405000
1	15.959348000	10.872824000	3.625735000
6	15.234789000	11.354688000	1.665469000
1	14.180454000	11.139938000	1.898411000
1	15.676362000	10.459049000	1.202194000
1	15.267817000	12.161570000	0.915272000
6	17.499045000	11.981928000	2.619840000
1	17.626522000	12.867923000	1.975585000
1	17.941220000	11.121179000	2.094159000
1	18.068612000	12.144839000	3.546432000
6	14.109193000	14.908710000	6.605692000
1	14.717148000	14.444367000	7.401074000
6	14.290976000	16.423896000	6.706361000
1	15.346749000	16.704245000	6.566343000
1	13.960073000	16.784913000	7.692753000
1	13.691875000	16.953187000	5.947737000
6	12.658296000	14.475682000	6.833846000
1	11.989439000	14.880352000	6.056542000
1	12.291488000	14.837090000	7.807418000
1	12.586382000	13.379177000	6.826905000
6	17.795189000	14.455641000	5.358132000
1	18.305317000	14.948399000	6.197855000
1	17.083851000	15.160421000	4.910367000
1	18.556646000	14.181038000	4.610838000
6	20.700140000	12.013569000	8.584793000
6	21.482934000	13.063629000	8.071517000
1	21.057149000	13.698933000	7.294092000
6	22.771122000	13.284038000	8.558425000
1	23.371905000	14.103711000	8.156471000
6	23.293797000	12.457595000	9.560779000
1	24.299186000	12.639478000	9.950009000
6	22.526259000	11.400754000	10.065994000
1	22.935509000	10.749838000	10.842946000
6	21.235905000	11.178957000	9.582602000
1	20.620376000	10.367078000	9.971894000
6	17.015339000	8.868929000	9.781519000
1	17.780065000	8.175409000	9.397507000
1	16.166889000	8.295613000	10.175202000
1	17.461082000	9.454153000	10.599638000
6	8.832095000	12.357031000	9.250659000
6	9.380047000	12.926570000	8.096407000
6	8.897927000	10.971405000	9.437359000
1	9.323773000	14.007292000	7.938320000
1	8.466351000	10.516443000	10.333419000
6	9.996857000	12.115639000	7.138245000
6	9.518511000	10.168363000	8.475089000
1	10.414267000	12.575081000	6.239803000
1	9.559136000	9.086426000	8.639858000

6	10.085028000	10.724292000	7.311787000
1	8.352163000	12.990525000	10.001401000
14	10.861634000	9.615864000	6.014251000
1	11.735536000	8.625875000	6.678607000
1	9.753317000	8.833055000	5.392112000
8	11.478997000	10.418646000	4.708687000
6	12.694770000	11.172141000	4.884539000
1	12.425608000	12.236392000	4.749323000
8	13.213006000	10.904674000	6.113293000

20

27	18.151837000	12.375843000	8.462618000
1	20.543396000	11.931684000	10.321877000
15	17.939257000	10.374903000	9.269161000
7	19.352660000	9.621157000	8.538050000
6	20.180242000	10.411892000	7.791430000
7	21.274533000	9.892951000	7.227454000
6	21.999015000	10.737908000	6.480199000
7	21.677389000	12.015276000	6.242732000
6	20.555332000	12.461675000	6.823746000
7	19.816410000	11.697754000	7.655339000
7	20.074171000	13.706438000	6.566599000
15	18.501614000	14.125380000	7.249793000
7	17.651013000	14.528278000	5.860821000
6	17.074121000	15.863953000	5.911177000
1	16.061381000	15.861602000	6.357512000
1	16.997877000	16.301125000	4.902922000
6	18.041144000	16.657677000	6.789175000
1	18.861518000	17.087340000	6.181946000
1	17.528721000	17.488783000	7.292906000
7	18.532931000	15.698647000	7.774585000
6	19.041747000	16.149009000	9.079910000
1	19.232964000	15.228922000	9.644440000
6	20.353066000	16.921584000	8.921332000
1	21.113418000	16.290678000	8.439217000
1	20.745031000	17.229558000	9.903684000
1	20.217888000	17.834361000	8.315759000
6	17.987119000	16.943730000	9.852866000
1	17.734624000	17.900836000	9.365890000
1	18.367012000	17.183502000	10.858386000
1	17.066419000	16.353357000	9.963109000
6	17.226722000	13.535911000	4.875374000
1	17.780186000	12.617478000	5.128728000
6	17.634310000	13.954192000	3.457056000
1	17.069974000	14.839539000	3.120995000
1	17.423601000	13.144075000	2.741373000
1	18.707610000	14.193086000	3.406748000
6	15.731259000	13.217430000	4.963213000

1	15.457133000	12.905717000	5.980643000
1	15.467461000	12.406945000	4.265985000
1	15.121824000	14.095494000	4.693073000
7	16.592946000	9.444226000	8.932781000
6	16.111673000	8.639227000	10.048040000
1	16.372338000	7.570964000	9.919526000
1	15.016012000	8.714479000	10.131533000
6	16.781688000	9.214089000	11.300777000
1	16.138392000	9.963764000	11.782043000
1	16.974128000	8.417355000	12.034325000
7	18.037185000	9.791282000	10.836165000
6	19.206553000	9.896783000	11.711214000
1	20.034850000	10.215711000	11.061474000
6	19.038236000	10.940242000	12.817150000
1	18.813287000	11.922569000	12.383801000
1	19.957003000	11.013600000	13.420715000
1	18.211837000	10.663717000	13.491201000
6	19.596182000	8.532999000	12.300597000
1	18.865845000	8.187652000	13.049448000
1	20.568859000	8.611410000	12.810417000
1	19.681311000	7.764008000	11.520075000
6	16.079621000	9.225405000	7.578687000
1	16.693254000	9.863202000	6.923606000
6	16.268261000	7.768561000	7.135653000
1	17.315188000	7.450417000	7.248261000
1	15.979531000	7.648114000	6.079729000
1	15.638167000	7.083250000	7.724641000
6	14.624222000	9.674464000	7.426863000
1	13.961696000	9.132230000	8.120394000
1	14.266307000	9.473363000	6.404552000
1	14.528263000	10.750443000	7.624413000
6	19.696976000	8.217501000	8.709522000
1	19.842588000	7.731036000	7.734149000
1	18.879950000	7.723439000	9.247566000
1	20.630128000	8.104153000	9.282664000
6	23.236039000	10.206620000	5.856848000
6	23.691600000	8.918391000	6.183521000
1	23.122467000	8.334835000	6.907371000
6	24.853620000	8.413805000	5.600660000
1	25.210205000	7.416914000	5.873387000
6	25.568427000	9.184468000	4.676285000
1	26.473178000	8.783786000	4.211454000
6	25.120061000	10.467871000	4.346512000
1	25.677073000	11.073964000	3.627652000
6	23.965295000	10.980676000	4.938741000
1	23.605962000	11.980815000	4.694683000
6	20.820968000	14.638930000	5.738494000
1	21.434737000	15.313703000	6.356068000
1	20.113611000	15.239317000	5.150693000
1	21.481249000	14.083741000	5.060680000

6	24.279121000	9.320751000	10.551929000
6	24.616397000	9.918488000	9.335218000
6	23.482846000	10.014817000	11.470909000
1	25.231049000	9.381477000	8.609781000
1	23.215037000	9.551390000	12.424452000
6	24.156437000	11.206118000	9.036615000
6	23.041984000	11.305717000	11.174064000
1	24.419606000	11.652919000	8.074444000
1	22.438520000	11.841612000	11.912783000
6	23.366602000	11.925329000	9.949516000
1	24.643075000	8.317640000	10.791023000
14	22.744395000	13.651166000	9.575239000
1	23.216090000	14.554884000	10.663460000
1	23.302709000	14.113802000	8.281539000
8	21.113728000	13.701716000	9.457171000
6	20.223542000	12.982578000	10.389169000
1	20.473576000	13.358013000	11.398533000
8	18.940507000	13.184864000	10.108041000
6	16.076251000	12.736412000	11.907256000
6	15.415150000	12.452814000	10.696551000
6	15.584245000	12.265355000	13.127327000
1	16.112738000	12.505527000	14.054310000
6	14.247136000	11.670545000	10.754629000
6	14.421245000	11.486419000	13.163980000
1	13.711521000	11.434084000	9.831835000
1	14.036264000	11.107656000	14.115132000
6	13.753471000	11.189010000	11.971626000
1	12.845435000	10.579346000	11.991519000
1	16.998265000	13.321602000	11.877864000
14	16.099635000	13.111936000	9.067227000
1	17.403601000	11.830203000	7.315010000
1	14.997982000	12.818362000	8.095829000
1	16.038378000	14.601124000	9.206231000

21

27	18.608693000	12.408275000	8.540062000
1	20.267932000	12.686840000	11.931612000
15	18.281897000	10.380363000	9.119340000
7	19.710874000	9.621620000	8.402726000
6	20.462802000	10.418010000	7.597656000
7	21.503045000	9.913451000	6.927339000
6	22.124739000	10.752302000	6.085250000
7	21.681963000	11.979951000	5.788133000
6	20.621859000	12.414945000	6.478141000
7	20.074012000	11.718014000	7.514044000
7	20.006736000	13.582565000	6.161652000
15	18.596242000	14.003274000	7.151695000
7	17.385712000	14.293630000	6.021649000

6	17.005231000	15.697213000	5.897048000
1	16.091542000	15.911976000	6.478709000
1	16.805427000	15.973813000	4.847520000
6	18.177075000	16.503646000	6.469519000
1	18.900331000	16.775333000	5.675878000
1	17.816722000	17.441971000	6.918201000
7	18.762001000	15.636723000	7.480585000
6	19.724137000	16.126982000	8.466306000
1	19.960748000	15.253533000	9.086270000
6	21.031721000	16.624803000	7.837237000
1	21.525463000	15.819662000	7.274445000
1	21.728149000	16.966020000	8.619409000
1	20.859599000	17.472589000	7.153109000
6	19.099794000	17.168374000	9.395983000
1	18.845470000	18.099890000	8.863524000
1	19.805474000	17.425420000	10.200842000
1	18.183861000	16.767301000	9.854162000
6	16.440942000	13.230315000	5.668616000
1	16.840497000	12.325828000	6.157694000
6	16.409460000	12.985431000	4.157465000
1	16.059177000	13.879261000	3.613998000
1	15.722217000	12.160529000	3.909521000
1	17.411913000	12.724972000	3.783191000
6	15.040549000	13.477435000	6.240948000
1	15.087352000	13.643239000	7.328156000
1	14.394143000	12.607728000	6.054019000
1	14.556325000	14.350668000	5.775528000
7	16.934678000	9.487020000	8.664988000
6	16.467287000	8.521928000	9.649262000
1	16.815252000	7.497518000	9.416111000
1	15.366466000	8.503214000	9.682646000
6	17.036742000	8.995894000	10.987174000
1	16.319291000	9.654580000	11.504201000
1	17.241554000	8.137633000	11.647189000
7	18.265839000	9.700228000	10.655047000
6	19.267399000	9.979802000	11.681233000
1	20.018764000	10.629070000	11.203249000
6	18.676101000	10.718663000	12.885901000
1	18.154110000	11.628335000	12.568073000
1	19.467475000	10.993696000	13.599715000
1	17.948498000	10.086919000	13.420269000
6	19.979879000	8.695235000	12.127059000
1	19.275222000	7.999146000	12.610737000
1	20.771125000	8.929314000	12.853821000
1	20.444702000	8.182677000	11.275105000
6	16.510700000	9.381847000	7.270391000
1	17.129081000	10.111018000	6.719654000
6	16.785427000	7.985561000	6.696238000
1	17.838536000	7.700278000	6.834063000
1	16.551764000	7.956395000	5.620252000

1	16.159335000	7.223656000	7.187455000
6	15.042883000	9.775288000	7.082461000
1	14.378063000	9.123522000	7.670961000
1	14.750839000	9.678141000	6.024735000
1	14.871855000	10.813006000	7.396309000
6	20.047217000	8.213062000	8.520623000
1	20.097473000	7.730125000	7.533252000
1	19.279705000	7.723135000	9.130579000
1	21.025072000	8.083810000	9.009004000
6	23.375691000	10.287222000	5.443343000
6	23.954177000	9.063438000	5.824889000
1	23.458507000	8.469531000	6.593819000
6	25.136432000	8.624967000	5.228213000
1	25.583921000	7.678298000	5.542210000
6	25.750982000	9.396052000	4.234112000
1	26.670941000	9.045657000	3.758560000
6	25.182974000	10.616962000	3.851272000
1	25.662650000	11.225751000	3.080453000
6	24.008093000	11.064807000	4.456555000
1	23.560734000	12.017745000	4.171206000
6	20.405326000	14.338826000	4.985105000
1	20.978161000	13.696366000	4.305270000
1	21.026909000	15.207781000	5.251395000
1	19.501597000	14.694990000	4.470797000
6	23.420048000	9.445311000	11.477255000
6	23.225192000	9.747429000	10.127377000
6	23.270099000	10.446829000	12.444682000
1	23.344762000	8.977019000	9.362659000
1	23.431900000	10.212048000	13.500221000
6	22.875209000	11.046776000	9.749226000
6	22.915915000	11.742749000	12.060724000
1	22.738365000	11.272960000	8.692405000
1	22.804208000	12.516036000	12.826872000
6	22.707331000	12.063044000	10.704141000
1	23.692702000	8.431162000	11.780878000
14	22.233551000	13.773284000	10.139542000
1	23.319538000	14.755034000	10.378356000
1	21.946808000	13.713001000	8.689447000
8	20.961943000	14.379725000	11.011905000
6	19.892556000	13.635632000	11.545778000
1	19.459580000	14.223838000	12.356422000
8	18.923714000	13.371355000	10.577087000
6	15.544692000	12.348084000	12.461994000
6	15.885416000	12.556400000	11.112864000
6	14.508607000	11.478886000	12.826231000
1	14.273700000	11.313635000	13.881713000
6	15.125361000	11.883555000	10.140688000
6	13.765601000	10.823160000	11.838911000
1	15.387950000	12.017170000	9.090920000
1	12.955017000	10.145489000	12.120954000

6	14.069329000	11.038730000	10.490049000
1	13.495224000	10.532653000	9.709983000
1	16.105135000	12.870935000	13.242387000
14	17.264024000	13.767471000	10.633108000
1	17.243938000	12.872206000	9.088867000
1	16.771241000	14.887399000	9.783904000
1	17.371523000	14.485823000	11.973385000

23

27	18.388444000	12.863832000	8.449435000
1	21.608121000	14.082808000	10.489213000
15	18.192909000	10.955032000	9.464570000
7	19.650347000	10.130721000	8.779975000
6	20.323259000	10.752211000	7.777797000
7	21.342757000	10.155720000	7.139712000
6	21.932374000	10.878604000	6.175954000
7	21.556438000	12.107829000	5.794216000
6	20.524548000	12.639882000	6.466520000
7	19.906199000	11.995684000	7.476232000
7	20.025212000	13.860254000	6.151287000
15	18.425308000	14.298753000	6.857170000
7	17.394253000	14.271729000	5.560552000
6	17.307123000	15.553800000	4.865481000
1	16.268718000	15.755548000	4.569415000
1	17.921290000	15.563199000	3.946267000
6	17.781352000	16.635549000	5.856858000
1	18.443574000	17.359901000	5.351062000
1	16.917106000	17.193823000	6.249941000
7	18.471424000	15.946776000	6.941859000
6	19.090962000	16.668136000	8.064059000
1	19.105823000	15.960434000	8.904041000
6	20.538374000	17.063260000	7.745350000
1	21.143286000	16.174044000	7.511958000
1	20.998891000	17.574980000	8.605336000
1	20.586708000	17.748090000	6.881714000
6	18.256628000	17.867855000	8.511331000
1	18.202626000	18.650019000	7.736803000
1	18.717226000	18.314683000	9.405214000
1	17.237167000	17.554925000	8.780092000
6	16.804659000	13.039420000	5.010564000
1	16.383747000	12.496012000	5.870828000
6	17.843023000	12.134825000	4.334191000
1	18.392523000	12.679317000	3.549348000
1	17.348811000	11.268833000	3.865893000
1	18.569555000	11.744060000	5.059733000
6	15.645120000	13.350059000	4.068402000
1	14.900967000	13.989677000	4.557946000
1	15.144735000	12.409950000	3.790113000

1	15.991072000	13.834032000	3.140373000
7	16.937494000	9.898969000	9.153141000
6	16.820666000	8.860300000	10.175222000
1	17.412110000	7.962140000	9.920182000
1	15.774298000	8.541607000	10.283945000
6	17.322712000	9.501951000	11.469785000
1	16.481279000	9.949057000	12.025952000
1	17.799064000	8.748653000	12.119442000
7	18.276488000	10.530902000	11.066996000
6	19.064856000	11.266317000	12.065738000
1	19.243397000	12.262305000	11.636885000
6	18.270788000	11.505277000	13.348462000
1	17.348840000	12.057606000	13.122976000
1	18.868221000	12.114317000	14.039896000
1	18.017224000	10.563194000	13.860647000
6	20.407575000	10.579665000	12.343167000
1	20.262474000	9.582676000	12.792084000
1	21.006024000	11.180926000	13.046111000
1	20.989896000	10.449798000	11.417548000
6	16.327662000	9.700599000	7.834517000
1	16.409703000	10.672868000	7.326426000
6	17.059490000	8.657245000	6.979418000
1	18.085888000	8.977211000	6.744205000
1	16.527663000	8.499631000	6.027656000
1	17.108307000	7.684720000	7.495863000
6	14.837922000	9.369705000	7.964426000
1	14.674857000	8.331038000	8.294775000
1	14.341260000	9.491755000	6.990090000
1	14.350173000	10.041710000	8.684752000
6	20.006073000	8.766725000	9.148077000
1	19.486596000	8.029600000	8.514889000
1	19.726748000	8.598376000	10.194554000
1	21.087269000	8.616137000	9.034938000
6	23.089329000	10.269540000	5.473483000
6	23.641890000	9.060823000	5.930198000
1	23.198558000	8.577177000	6.801061000
6	24.741123000	8.498863000	5.280390000
1	25.173435000	7.566297000	5.651955000
6	25.293474000	9.130898000	4.160234000
1	26.149872000	8.685807000	3.647029000
6	24.744235000	10.331424000	3.695754000
1	25.174453000	10.828307000	2.822529000
6	23.652035000	10.901323000	4.350762000
1	23.214740000	11.837091000	4.001272000
6	20.558285000	14.567940000	4.997316000
1	21.653599000	14.498166000	4.982692000
1	20.266977000	15.622841000	5.065116000
1	20.173255000	14.145548000	4.054299000
6	20.254024000	14.308914000	15.849022000
6	18.874961000	14.535839000	15.908966000

6	20.915366000	14.384557000	14.617914000
1	18.352660000	14.487268000	16.868620000
1	21.995134000	14.218478000	14.566640000
6	18.163295000	14.824352000	14.739731000
6	20.194631000	14.680159000	13.457292000
1	17.084800000	14.999424000	14.800392000
1	20.731364000	14.756191000	12.507081000
6	18.804694000	14.897274000	13.489989000
1	20.815198000	14.080118000	16.758924000
14	17.828134000	15.231567000	11.917573000
1	16.577169000	15.916375000	12.365678000
1	18.627894000	16.240261000	11.157218000
8	17.573316000	13.861147000	11.109052000
6	20.888916000	13.414550000	10.000146000
1	21.133588000	12.345588000	9.961256000
8	19.867968000	13.860016000	9.527872000
6	14.416604000	13.035394000	7.614414000
6	15.213046000	14.099552000	8.076899000
6	13.360432000	13.221510000	6.715949000
1	12.758369000	12.368231000	6.386461000
6	14.899842000	15.372582000	7.568077000
6	13.064899000	14.505302000	6.240506000
1	15.483197000	16.233597000	7.909349000
1	12.236092000	14.663459000	5.544625000
6	13.849048000	15.583455000	6.665160000
1	13.629185000	16.591760000	6.298084000
1	14.612956000	12.030214000	7.995338000
14	16.555860000	13.865359000	9.529797000
1	17.331042000	12.277594000	7.670972000
1	16.548835000	15.378544000	9.679280000
1	15.618332000	12.919456000	10.240060000

24

27	17.756810000	12.686750000	8.712389000
15	17.634483000	10.823390000	9.675262000
7	19.343426000	10.291354000	9.510971000
6	20.004071000	10.882502000	8.488379000
7	21.151760000	10.381538000	8.018088000
6	21.667424000	11.000580000	6.948138000
7	21.033364000	11.948368000	6.245422000
6	19.876363000	12.390257000	6.755826000
7	19.400514000	11.983362000	7.960786000
7	19.105206000	13.283799000	6.085606000
15	17.614948000	13.833516000	6.918458000
7	16.365143000	13.631297000	5.821190000
6	16.241408000	14.696885000	4.839037000
1	15.193747000	14.823914000	4.525595000
1	16.829650000	14.492030000	3.924711000

6	16.753862000	15.954061000	5.548964000
1	17.259677000	16.622232000	4.830792000
1	15.913351000	16.519145000	5.992506000
7	17.665964000	15.492213000	6.586365000
6	18.572070000	16.437786000	7.239700000
1	18.783657000	16.015300000	8.232818000
6	19.907557000	16.591422000	6.496928000
1	20.419639000	15.626155000	6.391674000
1	20.577197000	17.270967000	7.048304000
1	19.752704000	17.014957000	5.490302000
6	17.917926000	17.806790000	7.453473000
1	17.769622000	18.333787000	6.497435000
1	18.569932000	18.435337000	8.078467000
1	16.947606000	17.720139000	7.952825000
6	15.700336000	12.346035000	5.639834000
1	16.005732000	11.755004000	6.516453000
6	16.170951000	11.616767000	4.375357000
1	15.944385000	12.202620000	3.468873000
1	15.669040000	10.641640000	4.275021000
1	17.257679000	11.440512000	4.405154000
6	14.178429000	12.494527000	5.689222000
1	13.874173000	13.000726000	6.618115000
1	13.695261000	11.505825000	5.652670000
1	13.795213000	13.079124000	4.836976000
7	16.761280000	9.521756000	9.041396000
6	16.586961000	8.439401000	9.997927000
1	17.439152000	7.733779000	9.991352000
1	15.679532000	7.855009000	9.778517000
6	16.471298000	9.135409000	11.355008000
1	15.413082000	9.366002000	11.572920000
1	16.844068000	8.482474000	12.162636000
7	17.255996000	10.361156000	11.248420000
6	17.214050000	11.331680000	12.342786000
1	17.779249000	12.202087000	11.976748000
6	15.787519000	11.803949000	12.653314000
1	15.304085000	12.185696000	11.741779000
1	15.801854000	12.614090000	13.398313000
1	15.170801000	10.990215000	13.066879000
6	17.916287000	10.800265000	13.596669000
1	17.369819000	9.948893000	14.034452000
1	17.973467000	11.585322000	14.367136000
1	18.937247000	10.463261000	13.362353000
6	16.755731000	9.244648000	7.606689000
1	17.059970000	10.194172000	7.136983000
6	17.771562000	8.175601000	7.188237000
1	18.792993000	8.463189000	7.479716000
1	17.754801000	8.041761000	6.095206000
1	17.542423000	7.198834000	7.645231000
6	15.338082000	8.909557000	7.132323000
1	14.970821000	7.978749000	7.595050000

1	15.307173000	8.771889000	6.039948000
1	14.645301000	9.720818000	7.401625000
6	19.914257000	9.129589000	10.167596000
1	19.819077000	8.216559000	9.555638000
1	19.389224000	8.981092000	11.119436000
1	20.978722000	9.300863000	10.370754000
6	23.025349000	10.604236000	6.504634000
6	23.728750000	9.595773000	7.187123000
1	23.248806000	9.116189000	8.040722000
6	25.006569000	9.220954000	6.769805000
1	25.544167000	8.433446000	7.304277000
6	25.602224000	9.853557000	5.671576000
1	26.598635000	9.550639000	5.338071000
6	24.914250000	10.869226000	4.996376000
1	25.379748000	11.373674000	4.145497000
6	23.634818000	11.242759000	5.409306000
1	23.086549000	12.031117000	4.892399000
6	19.444638000	13.662879000	4.723508000
1	20.529184000	13.592047000	4.571210000
1	19.121469000	14.693998000	4.538647000
1	18.948137000	13.003577000	3.991898000
6	22.778472000	10.920570000	11.672030000
6	21.611112000	11.341009000	12.320819000
6	23.191969000	11.556388000	10.498437000
1	21.277524000	10.833505000	13.229619000
1	24.084342000	11.209904000	9.971253000
6	20.867198000	12.402400000	11.801232000
6	22.442670000	12.617581000	9.982071000
1	19.956585000	12.719614000	12.314227000
1	22.765621000	13.094373000	9.051695000
6	21.273328000	13.059467000	10.626263000
1	23.359179000	10.087315000	12.076168000
14	20.360245000	14.522044000	9.936675000
1	20.807783000	15.773020000	10.609245000
1	20.678280000	14.635467000	8.500783000
8	18.731933000	14.405796000	10.178092000
6	15.465252000	15.742892000	9.116407000
6	16.318669000	16.221733000	10.131010000
6	14.486219000	16.562394000	8.552652000
1	13.842114000	16.165973000	7.763009000
6	16.146888000	17.551972000	10.564369000
6	14.327319000	17.879856000	8.998627000
1	16.795062000	17.956764000	11.346925000
1	13.560747000	18.522314000	8.557555000
6	15.157345000	18.371989000	10.010403000
1	15.034894000	19.397766000	10.368625000
1	15.577151000	14.713553000	8.775283000
14	17.557822000	15.175839000	11.055323000
1	16.385321000	13.063516000	9.238622000
1	18.268934000	16.119614000	11.962917000

1	16.871742000	14.165866000	11.876608000

25

27	14.890846000	12.097114000	7.475396000
1	13.558371000	14.218353000	8.242630000
15	15.387621000	12.967135000	5.623221000
7	17.134372000	13.287483000	5.898138000
6	17.684987000	12.734388000	7.007176000
7	19.006428000	12.766983000	7.235829000
6	19.425729000	12.109356000	8.327954000
7	18.651482000	11.330485000	9.098775000
6	17.350691000	11.310023000	8.778176000
7	16.820665000	12.128355000	7.844677000
7	16.494395000	10.400087000	9.301098000
15	15.115598000	10.029438000	8.204281000
7	14.029986000	9.241852000	9.194463000
6	13.921894000	7.807961000	8.933000000
1	13.064995000	7.588809000	8.270296000
1	13.777399000	7.239312000	9.865978000
6	15.226797000	7.416583000	8.240493000
1	16.002378000	7.153960000	8.984037000
1	15.082825000	6.539105000	7.592701000
7	15.590847000	8.593555000	7.454873000
6	16.736937000	8.543001000	6.542096000
1	16.803142000	9.552214000	6.107381000
6	18.085794000	8.250866000	7.216311000
1	18.327779000	8.983751000	7.996260000
1	18.889358000	8.295313000	6.464349000
1	18.105656000	7.244820000	7.666610000
6	16.462988000	7.554052000	5.406734000
1	16.415740000	6.520402000	5.785230000
1	17.264601000	7.592663000	4.654227000
1	15.505952000	7.782218000	4.915301000
6	12.947416000	9.965380000	9.879142000
1	13.192957000	11.030189000	9.764703000
6	12.924684000	9.641908000	11.375227000
1	12.685086000	8.580917000	11.561448000
1	12.156204000	10.246032000	11.881901000
1	13.895768000	9.870677000	11.840711000
6	11.581872000	9.727664000	9.229807000
1	11.617391000	9.958730000	8.153584000
1	10.829634000	10.383926000	9.693883000
1	11.236953000	8.687899000	9.359404000
7	14.932729000	14.439934000	4.953948000
6	14.547559000	14.388589000	3.548602000
1	14.931140000	15.264543000	2.999345000
1	13.448151000	14.377820000	3.429128000
6	15.158507000	13.096007000	2.999718000

1	14.530152000	12.667917000	2.204274000
1	16.157953000	13.282315000	2.562205000
7	15.219585000	12.203979000	4.146789000
6	15.438553000	10.773544000	3.989748000
1	15.470941000	10.387514000	5.018553000
6	14.247840000	10.099111000	3.305659000
1	13.327151000	10.309498000	3.871634000
1	14.389422000	9.007688000	3.269719000
1	14.110572000	10.452634000	2.270404000
6	16.783799000	10.478495000	3.317473000
1	16.822881000	10.890288000	2.295516000
1	16.963715000	9.396266000	3.247937000
1	17.605880000	10.929724000	3.895854000
6	14.585732000	15.600610000	5.770150000
1	14.884365000	15.339074000	6.792829000
6	15.393255000	16.840669000	5.374564000
1	16.473001000	16.650015000	5.473540000
1	15.134847000	17.689111000	6.027044000
1	15.187483000	17.146754000	4.335351000
6	13.077040000	15.870645000	5.770519000
1	12.727110000	16.227845000	4.787980000
1	12.824247000	16.643924000	6.512046000
1	12.521374000	14.955079000	6.023591000
6	17.992173000	13.954091000	4.932227000
1	18.701525000	14.619538000	5.444574000
1	17.362291000	14.552271000	4.263716000
1	18.572693000	13.232432000	4.333491000
6	20.862875000	12.209769000	8.682224000
6	21.715122000	13.056472000	7.952403000
1	21.298897000	13.623084000	7.118650000
6	23.062010000	13.165528000	8.299721000
1	23.718065000	13.830708000	7.732898000
6	23.573664000	12.423729000	9.371272000
1	24.627137000	12.517445000	9.648751000
6	22.734284000	11.565597000	10.091952000
1	23.134845000	10.978263000	10.922208000
6	21.385340000	11.460171000	9.751144000
1	20.714224000	10.800827000	10.302588000
6	16.918117000	9.551414000	10.401005000
1	17.636910000	8.775953000	10.088003000
1	16.029023000	9.064700000	10.821861000
1	17.397367000	10.163460000	11.178380000
6	14.492507000	13.729676000	8.537388000
1	15.359257000	14.398945000	8.622948000
8	14.439826000	12.692102000	9.315670000
1	13.467438000	11.956295000	7.049072000

26

27	15.004482000	11.795212000	7.200512000
1	12.193761000	11.760985000	7.998615000
15	15.495645000	12.840976000	5.402776000
7	17.196486000	13.232268000	5.763634000
6	17.722857000	12.658032000	6.870213000
7	19.019955000	12.792308000	7.167844000
6	19.457275000	12.125909000	8.243356000
7	18.715478000	11.260459000	8.947370000
6	17.432837000	11.158446000	8.590705000
7	16.859756000	11.932817000	7.630387000
7	16.627581000	10.225680000	9.150655000
15	15.094608000	9.957560000	8.290058000
7	14.085500000	9.417508000	9.503603000
6	13.780853000	7.991939000	9.468907000
1	12.762886000	7.813861000	9.074880000
1	13.830481000	7.540738000	10.474516000
6	14.829879000	7.358059000	8.539934000
1	15.692295000	6.982244000	9.124350000
1	14.402090000	6.500029000	7.998087000
7	15.209253000	8.421805000	7.617923000
6	16.100095000	8.194552000	6.480873000
1	16.222920000	9.194855000	6.030333000
6	17.492950000	7.698889000	6.890820000
1	17.980300000	8.412612000	7.570851000
1	18.135853000	7.588038000	6.003520000
1	17.448439000	6.719658000	7.396682000
6	15.443206000	7.298387000	5.428460000
1	15.239197000	6.288127000	5.820617000
1	16.100523000	7.187591000	4.551538000
1	14.488715000	7.735066000	5.095271000
6	13.386344000	10.352446000	10.391726000
1	13.591975000	11.355744000	9.982445000
6	13.936697000	10.301768000	11.820935000
1	13.825148000	9.295975000	12.260011000
1	13.394198000	11.009035000	12.467847000
1	15.002452000	10.574033000	11.840697000
6	11.871162000	10.135814000	10.371789000
1	11.485522000	10.122514000	9.341031000
1	11.365895000	10.947932000	10.915758000
1	11.588456000	9.186562000	10.856956000
7	14.894156000	14.331094000	4.949243000
6	14.748693000	14.558515000	3.520068000
1	15.223557000	15.503839000	3.203514000
1	13.682399000	14.622307000	3.234751000
6	15.426314000	13.360532000	2.824360000
1	14.852513000	13.058184000	1.936927000
1	16.441831000	13.627755000	2.474596000
7	15.446495000	12.289163000	3.815890000

6	15.812330000	10.913114000	3.466106000
1	15.379443000	10.289671000	4.266618000
6	15.181949000	10.482003000	2.140222000
1	14.100351000	10.688559000	2.124085000
1	15.328873000	9.400604000	1.993960000
1	15.646097000	10.997857000	1.283937000
6	17.328107000	10.678526000	3.460890000
1	17.830465000	11.361852000	2.757514000
1	17.564855000	9.646572000	3.154509000
1	17.754440000	10.842896000	4.461308000
6	14.518694000	15.333320000	5.948268000
1	14.544245000	14.803812000	6.914931000
6	15.523728000	16.490449000	6.001988000
1	16.527722000	16.119281000	6.257232000
1	15.227947000	17.229268000	6.763309000
1	15.584309000	17.016176000	5.034276000
6	13.086803000	15.834441000	5.739291000
1	12.987872000	16.419198000	4.809433000
1	12.787983000	16.486769000	6.573751000
1	12.379984000	14.992111000	5.693041000
6	18.039978000	14.031880000	4.889816000
1	18.755916000	14.615957000	5.482476000
1	17.405991000	14.722845000	4.323081000
1	18.608532000	13.403296000	4.184751000
6	20.857736000	12.344412000	8.672988000
6	21.661687000	13.289632000	8.012188000
1	21.238084000	13.834652000	7.167898000
6	22.967740000	13.525005000	8.441523000
1	23.583927000	14.267177000	7.927927000
6	23.489349000	12.812414000	9.528153000
1	24.508693000	13.007903000	9.872015000
6	22.700859000	11.855417000	10.178830000
1	23.109022000	11.290703000	11.021117000
6	21.391842000	11.622786000	9.755729000
1	20.759780000	10.887947000	10.255705000
6	17.144835000	9.358789000	10.195066000
1	17.844436000	8.604980000	9.798117000
1	16.300066000	8.850361000	10.674174000
1	17.677687000	9.954094000	10.949475000
6	12.939115000	12.422053000	7.528047000
1	12.453018000	13.041454000	6.757510000
8	13.767123000	13.053395000	8.356841000
1	13.443204000	11.563577000	6.769844000

27

27	17.081866000	11.359328000	7.663650000
1	15.270656000	10.352279000	5.258885000
15	17.352203000	12.881101000	6.173507000

7	18.949226000	13.458755000	6.607287000
6	19.624525000	12.701549000	7.517829000
7	20.922890000	12.907850000	7.733624000
6	21.516881000	12.105293000	8.627874000
7	20.877016000	11.146592000	9.314226000
6	19.576898000	10.996144000	9.057224000
7	18.890246000	11.741985000	8.145485000
7	18.852438000	10.041478000	9.711884000
15	17.210859000	9.822719000	9.162862000
7	16.347018000	9.623797000	10.598758000
6	15.518132000	8.422126000	10.570014000
1	14.542636000	8.609111000	10.081482000
1	15.322521000	8.048017000	11.587265000
6	16.326832000	7.414572000	9.749869000
1	17.034596000	6.858439000	10.395995000
1	15.665853000	6.676852000	9.270036000
7	17.012896000	8.222111000	8.753181000
6	17.451467000	7.676940000	7.471505000
1	17.987186000	8.501918000	6.970492000
6	18.431305000	6.518195000	7.674361000
1	19.279706000	6.833484000	8.301914000
1	18.825297000	6.158018000	6.710716000
1	17.936163000	5.666925000	8.171205000
6	16.262381000	7.297781000	6.584806000
1	15.654247000	6.504483000	7.049320000
1	16.608409000	6.920179000	5.609060000
1	15.626358000	8.180601000	6.426276000
6	15.937573000	10.801575000	11.370299000
1	16.721345000	11.556454000	11.184446000
6	15.933392000	10.486856000	12.869292000
1	15.159637000	9.742595000	13.116847000
1	15.709369000	11.393471000	13.453493000
1	16.906770000	10.085082000	13.192575000
6	14.601631000	11.394551000	10.906079000
1	14.631449000	11.615744000	9.827339000
1	14.385098000	12.325731000	11.454024000
1	13.770220000	10.696726000	11.099000000
7	16.487883000	14.311556000	6.002557000
6	16.027073000	14.570687000	4.644661000
1	16.089394000	15.643451000	4.399712000
1	14.976600000	14.251186000	4.503321000
6	16.954101000	13.759641000	3.729389000
1	16.419725000	13.453161000	2.817573000
1	17.825043000	14.367002000	3.413054000
7	17.342309000	12.599935000	4.520481000
6	18.025928000	11.441267000	3.941672000
1	18.007420000	10.677843000	4.740308000
6	17.267769000	10.873098000	2.738039000
1	16.219972000	10.655565000	2.996633000
1	17.743779000	9.938379000	2.403438000

1	17.280990000	11.569987000	1.884182000
6	19.493075000	11.731435000	3.591011000
1	19.572002000	12.551345000	2.857672000
1	19.972287000	10.843046000	3.149422000
1	20.065034000	12.017106000	4.486043000
6	15.807442000	14.890890000	7.168164000
1	16.460943000	14.663750000	8.027871000
6	15.714428000	16.412619000	7.043465000
1	16.700867000	16.853824000	6.830057000
1	15.328338000	16.850716000	7.976772000
1	15.024157000	16.707928000	6.236636000
6	14.442515000	14.247168000	7.446062000
1	13.761305000	14.364314000	6.587566000
1	13.965263000	14.722960000	8.317993000
1	14.561940000	13.173355000	7.656074000
6	19.612043000	14.579753000	5.960459000
1	20.198687000	15.149486000	6.694800000
1	18.840877000	15.233246000	5.533143000
1	20.294974000	14.251301000	5.160595000
6	22.969991000	12.280428000	8.849769000
6	23.684927000	13.255889000	8.128855000
1	23.143266000	13.875516000	7.413577000
6	25.054956000	13.421283000	8.330745000
1	25.601083000	14.180565000	7.764879000
6	25.732125000	12.612944000	9.252130000
1	26.804166000	12.750576000	9.417546000
6	25.030971000	11.633228000	9.966046000
1	25.559047000	10.995642000	10.679719000
6	23.660180000	11.465636000	9.766129000
1	23.100353000	10.706943000	10.314096000
6	19.504773000	9.147182000	10.653408000
1	20.164999000	8.433106000	10.133363000
1	18.728027000	8.597048000	11.200056000
1	20.113221000	9.716873000	11.369926000
6	14.762112000	10.945942000	6.035480000
1	14.756472000	11.986232000	5.672673000
8	15.342096000	10.800447000	7.290956000
1	13.712868000	10.609828000	6.059013000

28

27	17.051849000	11.141364000	7.687718000
1	19.700392000	9.324521000	6.951831000
15	17.122813000	12.960567000	6.552912000
7	18.794275000	13.452867000	6.936448000
6	19.511271000	12.655340000	7.773066000
7	20.801625000	12.897202000	8.031023000
6	21.408443000	12.036162000	8.858642000
7	20.820351000	10.973247000	9.429216000

6	19.530596000	10.791837000	9.124555000
7	18.843948000	11.622099000	8.318713000
7	18.830591000	9.737239000	9.628423000
15	17.143658000	9.569200000	9.130136000
7	16.352448000	9.516165000	10.600485000
6	15.594151000	8.291420000	10.811423000
1	14.539845000	8.406673000	10.498205000
1	15.609433000	7.997641000	11.873125000
6	16.291573000	7.237748000	9.946861000
1	17.077246000	6.708625000	10.520885000
1	15.575653000	6.479490000	9.600088000
7	16.840408000	7.972502000	8.814811000
6	17.121075000	7.313030000	7.531332000
1	17.397143000	8.131407000	6.846261000
6	18.297346000	6.342179000	7.660424000
1	19.187994000	6.860530000	8.048271000
1	18.555965000	5.910335000	6.680711000
1	18.061404000	5.505658000	8.340967000
6	15.869453000	6.639882000	6.963567000
1	15.533274000	5.786078000	7.576042000
1	16.079729000	6.251221000	5.954907000
1	15.043982000	7.361974000	6.886933000
6	16.134569000	10.711373000	11.414463000
1	16.850025000	11.458034000	11.033877000
6	16.474312000	10.443441000	12.884402000
1	15.762389000	9.737787000	13.342096000
1	16.425622000	11.376598000	13.466911000
1	17.485521000	10.017980000	12.981699000
6	14.726921000	11.294626000	11.249845000
1	14.537472000	11.561471000	10.201047000
1	14.611262000	12.203399000	11.861005000
1	13.956842000	10.576496000	11.574847000
7	16.267458000	14.363403000	6.808320000
6	15.870528000	15.074236000	5.598346000
1	16.174595000	16.134919000	5.641652000
1	14.775258000	15.045599000	5.480378000
6	16.562596000	14.367605000	4.415903000
1	15.865626000	14.268513000	3.569077000
1	17.430401000	14.950481000	4.052536000
7	16.954255000	13.051905000	4.904280000
6	17.422068000	11.984616000	4.005379000
1	17.523113000	11.100076000	4.656321000
6	16.381966000	11.650862000	2.937348000
1	15.423151000	11.375583000	3.402680000
1	16.726471000	10.789262000	2.344984000
1	16.210092000	12.482455000	2.232959000
6	18.794377000	12.296312000	3.397316000
1	18.780114000	13.207990000	2.775069000
1	19.122245000	11.463132000	2.756512000
1	19.550899000	12.423696000	4.185828000

6	15.946464000	14.890739000	8.136675000
1	16.128019000	14.057343000	8.834793000
6	16.869858000	16.050422000	8.533370000
1	17.920691000	15.727006000	8.564255000
1	16.599691000	16.435499000	9.529513000
1	16.784480000	16.890223000	7.823548000
6	14.471657000	15.281670000	8.245623000
1	14.228954000	16.127618000	7.582299000
1	14.238493000	15.598961000	9.274175000
1	13.820690000	14.439640000	7.983293000
6	19.403461000	14.637375000	6.351611000
1	20.183574000	15.024289000	7.019268000
1	18.631157000	15.405817000	6.220051000
1	19.859727000	14.421289000	5.371796000
6	22.842021000	12.275251000	9.152989000
6	23.519713000	13.342109000	8.537251000
1	22.968518000	13.974253000	7.840885000
6	24.864508000	13.577381000	8.819194000
1	25.385998000	14.406919000	8.334918000
6	25.546965000	12.749081000	9.717384000
1	26.597793000	12.943288000	9.949136000
6	24.882131000	11.676691000	10.323449000
1	25.417493000	11.023453000	11.017013000
6	23.537273000	11.437274000	10.041055000
1	23.000476000	10.606923000	10.500886000
6	19.475734000	8.798152000	10.530912000
1	20.117566000	8.090255000	9.981370000
1	18.699833000	8.238753000	11.068102000
1	20.099987000	9.332601000	11.260290000
6	19.205711000	9.810412000	6.091191000
1	19.765605000	10.743426000	5.903969000
8	17.840646000	9.986852000	6.247074000
1	19.398700000	9.160808000	5.223382000
6	12.380904000	13.522993000	5.666879000
6	13.436425000	12.615617000	5.796359000
6	11.448021000	13.667634000	6.699935000
1	14.156259000	12.509811000	4.981031000
1	10.620816000	14.375142000	6.598218000
6	13.584290000	11.820632000	6.948950000
6	11.578939000	12.894899000	7.858319000
1	10.853911000	13.000586000	8.670608000
6	12.632276000	11.981214000	7.973878000
1	12.707482000	11.369939000	8.877269000
1	12.276956000	14.116066000	4.753599000
14	14.968139000	10.535420000	7.082510000
1	14.358482000	9.541226000	8.028265000
1	14.974237000	9.850558000	5.752896000
1	16.341315000	11.932126000	8.716122000

29

27	17.081963000	11.371214000	7.956557000
1	16.362330000	9.732222000	3.893997000
15	17.229847000	13.292235000	7.038534000
7	18.957852000	13.615841000	7.290156000
6	19.641970000	12.678324000	7.997686000
7	20.960778000	12.776757000	8.196171000
6	21.509806000	11.858206000	9.002233000
7	20.805505000	10.971497000	9.721095000
6	19.498195000	10.908283000	9.451879000
7	18.896388000	11.652882000	8.487908000
7	18.683630000	10.063519000	10.140344000
15	17.096826000	9.812520000	9.422397000
7	16.088021000	9.607729000	10.769660000
6	15.451065000	8.287378000	10.759513000
1	14.488963000	8.317778000	10.212331000
1	15.245305000	7.937119000	11.781862000
6	16.429616000	7.353743000	10.050097000
1	17.173416000	6.946458000	10.764373000
1	15.894524000	6.502399000	9.602419000
7	17.037581000	8.186492000	9.025944000
6	17.882706000	7.640981000	7.961737000
1	18.063129000	8.490636000	7.285321000
6	19.238799000	7.128307000	8.469888000
1	19.837690000	7.943704000	8.900196000
1	19.816932000	6.685899000	7.642955000
1	19.112000000	6.349099000	9.239859000
6	17.139128000	6.572788000	7.159751000
1	16.974043000	5.658684000	7.752791000
1	17.728421000	6.289535000	6.274204000
1	16.162811000	6.950202000	6.820884000
6	15.294154000	10.737769000	11.287499000
1	14.471882000	10.958347000	10.574326000
6	16.151266000	11.997148000	11.414743000
1	16.978512000	11.839166000	12.125130000
1	15.532365000	12.833095000	11.774092000
1	16.575952000	12.279675000	10.438290000
6	14.671634000	10.383949000	12.642528000
1	13.928417000	9.577048000	12.570574000
1	14.160265000	11.265608000	13.058762000
1	15.455979000	10.071925000	13.352311000
7	16.522318000	14.743910000	7.506633000
6	15.937857000	15.515763000	6.417135000
1	16.148484000	16.591327000	6.532235000
1	14.841153000	15.387470000	6.379846000
6	16.578653000	14.985400000	5.130025000
1	15.861180000	15.034505000	4.297572000
1	17.457275000	15.596361000	4.843793000
7	16.931967000	13.606970000	5.426484000

6	17.358019000	12.671428000	4.386625000
1	17.361773000	11.693188000	4.886237000
6	16.337581000	12.606908000	3.249670000
1	15.329287000	12.428345000	3.652794000
1	16.590675000	11.787277000	2.559468000
1	16.325170000	13.537155000	2.658805000
6	18.778030000	12.949545000	3.874930000
1	18.861993000	13.959514000	3.440049000
1	19.050698000	12.227427000	3.089325000
1	19.513892000	12.859558000	4.687864000
6	16.146460000	15.013173000	8.895094000
1	16.823532000	14.398817000	9.510389000
6	16.394713000	16.480571000	9.254137000
1	17.428479000	16.773863000	9.011764000
1	16.222278000	16.648316000	10.328610000
1	15.712857000	17.150318000	8.706166000
6	14.709962000	14.581481000	9.218526000
1	13.985077000	15.082947000	8.558197000
1	14.449199000	14.840297000	10.256973000
1	14.598638000	13.494459000	9.096019000
6	19.606869000	14.850334000	6.880959000
1	20.451726000	15.069240000	7.546429000
1	18.874413000	15.666246000	6.949996000
1	19.984057000	14.795701000	5.846904000
6	22.988879000	11.786164000	9.067480000
6	23.778590000	12.651056000	8.287979000
1	23.279502000	13.404672000	7.678201000
6	25.168271000	12.526152000	8.285770000
1	25.774222000	13.194918000	7.669343000
6	25.786677000	11.538883000	9.062962000
1	26.874605000	11.431322000	9.048482000
6	25.008988000	10.682985000	9.852332000
1	25.490876000	9.912741000	10.459893000
6	23.619061000	10.804685000	9.854493000
1	22.996218000	10.135549000	10.448506000
6	19.185922000	9.354985000	11.305878000
1	19.837394000	8.510402000	11.032154000
1	18.322854000	8.982157000	11.871939000
1	19.764881000	10.038466000	11.943633000
6	16.880072000	9.245306000	4.725159000
1	16.631624000	8.181565000	4.704221000
8	16.538563000	9.843103000	5.966765000
1	17.955885000	9.362420000	4.580135000
6	12.014625000	11.716576000	4.166229000
6	12.925817000	10.819397000	4.734016000
6	11.930611000	13.024568000	4.653127000
1	12.979993000	9.798087000	4.347354000
1	11.218921000	13.731408000	4.218234000
6	13.775816000	11.198022000	5.792406000
6	12.767175000	13.423565000	5.701859000

1	12.700519000	14.443278000	6.092216000
6	13.681696000	12.522290000	6.255004000
1	14.336779000	12.841720000	7.064213000
1	11.367731000	11.392235000	3.346180000
14	14.938230000	9.885329000	6.507661000
1	14.513528000	9.304462000	7.813202000
1	14.471765000	8.727178000	5.626641000
1	15.569905000	11.212074000	7.614306000

TS1

27	-1.196558000	0.342052000	0.251068000
1	-2.685893000	0.211112000	-0.037737000
15	-0.876263000	1.612200000	-1.416387000
7	0.884746000	1.844267000	-1.280198000
6	1.506814000	1.173667000	-0.274514000
7	2.837377000	1.233434000	-0.130061000
6	3.357136000	0.534545000	0.887644000
7	2.647900000	-0.251315000	1.712555000
6	1.330619000	-0.290040000	1.496481000
7	0.707881000	0.443205000	0.541597000
7	0.529576000	-1.131592000	2.210243000
15	-1.030734000	-1.454245000	1.416841000
7	-1.912007000	-2.185285000	2.642584000
6	-2.261540000	-3.572529000	2.338787000
1	-3.243984000	-3.644707000	1.834584000
1	-2.307845000	-4.179235000	3.256457000
6	-1.160526000	-4.069469000	1.403646000
1	-0.291427000	-4.437319000	1.984416000
1	-1.522348000	-4.899006000	0.777065000
7	-0.832398000	-2.904650000	0.590306000
6	0.085924000	-2.988921000	-0.547337000
1	0.155021000	-1.956357000	-0.923782000
6	1.506527000	-3.427283000	-0.166786000
1	1.952560000	-2.753361000	0.577389000
1	2.153786000	-3.411347000	-1.057813000
1	1.523037000	-4.450302000	0.244757000
6	-0.501250000	-3.850941000	-1.666909000
1	-0.583915000	-4.906859000	-1.361902000
1	0.142721000	-3.812980000	-2.558779000
1	-1.503721000	-3.492390000	-1.943518000
6	-2.771567000	-1.379372000	3.525461000
1	-2.219425000	-0.443629000	3.698445000
6	-2.957377000	-2.065868000	4.881362000
1	-3.586094000	-2.967574000	4.798497000
1	-3.458528000	-1.381627000	5.582697000
1	-1.989370000	-2.361544000	5.316884000
6	-4.112171000	-1.018301000	2.875322000
1	-3.944171000	-0.484936000	1.928446000

1	-4.699153000	-0.366415000	3.541428000
1	-4.713593000	-1.919385000	2.670099000
7	-1.356528000	3.134794000	-1.960772000
6	-1.909178000	3.127144000	-3.312923000
1	-1.613245000	4.033352000	-3.865130000
1	-3.015003000	3.083886000	-3.299706000
6	-1.339754000	1.877123000	-3.987260000
1	-2.027920000	1.499038000	-4.757866000
1	-0.376087000	2.098608000	-4.486413000
7	-1.195726000	0.920535000	-2.903484000
6	-0.956084000	-0.498139000	-3.140932000
1	-0.965582000	-0.943231000	-2.133400000
6	-2.109026000	-1.131607000	-3.918104000
1	-3.057647000	-0.948601000	-3.389715000
1	-1.962255000	-2.218956000	-4.005128000
1	-2.192108000	-0.724978000	-4.939371000
6	0.419611000	-0.756455000	-3.767215000
1	0.506854000	-0.279723000	-4.757698000
1	0.599465000	-1.834836000	-3.897972000
1	1.216005000	-0.351323000	-3.123795000
6	-1.750075000	4.187978000	-1.015640000
1	-1.071301000	4.082628000	-0.157555000
6	-1.523706000	5.580154000	-1.609256000
1	-0.494468000	5.689814000	-1.986559000
1	-1.700379000	6.346685000	-0.838838000
1	-2.218059000	5.785474000	-2.439684000
6	-3.188359000	4.022367000	-0.504742000
1	-3.922409000	4.196291000	-1.308731000
1	-3.398945000	4.745950000	0.298357000
1	-3.344334000	3.008627000	-0.104335000
6	1.680280000	2.640701000	-2.202333000
1	2.385607000	3.278161000	-1.650386000
1	1.001100000	3.278645000	-2.779577000
1	2.261262000	2.004586000	-2.890851000
6	4.820767000	0.616108000	1.111934000
6	5.622800000	1.428144000	0.290223000
1	5.146090000	1.984869000	-0.517185000
6	6.996928000	1.516819000	0.515357000
1	7.613281000	2.152691000	-0.125027000
6	7.586740000	0.794754000	1.560234000
1	8.661668000	0.874769000	1.743731000
6	6.797004000	-0.025568000	2.374957000
1	7.257306000	-0.595852000	3.185954000
6	5.422437000	-0.114196000	2.153217000
1	4.791225000	-0.743778000	2.780795000
6	1.116201000	-1.971749000	3.241249000
1	1.783940000	-2.741090000	2.818438000
1	0.299375000	-2.461612000	3.785293000
1	1.705815000	-1.359265000	3.938023000
6	-1.305809000	2.174075000	2.025221000

8	-2.214618000	1.674114000	2.575590000
8	-0.442917000	2.926365000	1.759672000

TS2

27	14.849773000	11.978774000	7.374035000
1	12.366639000	12.707528000	7.947316000
15	15.360579000	12.945963000	5.586215000
7	17.106613000	13.231069000	5.850218000
6	17.635874000	12.744285000	6.996148000
7	18.956276000	12.791279000	7.224791000
6	19.382916000	12.188522000	8.341648000
7	18.600452000	11.475204000	9.165061000
6	17.299945000	11.440042000	8.852630000
7	16.754274000	12.181064000	7.857076000
7	16.456973000	10.559428000	9.450658000
15	15.124574000	10.063145000	8.353919000
7	14.098147000	9.226726000	9.370728000
6	14.050174000	7.790430000	9.103312000
1	13.204498000	7.535980000	8.438038000
1	13.930075000	7.213704000	10.034625000
6	15.373440000	7.459946000	8.408369000
1	16.162588000	7.237507000	9.151378000
1	15.271418000	6.574593000	7.762888000
7	15.671662000	8.651521000	7.620208000
6	16.775329000	8.671443000	6.657780000
1	16.769766000	9.694032000	6.246046000
6	18.157184000	8.423518000	7.279713000
1	18.387511000	9.142695000	8.076736000
1	18.937252000	8.524288000	6.508549000
1	18.231953000	7.406837000	7.699975000
6	16.507790000	7.700199000	5.506863000
1	16.532400000	6.654655000	5.854575000
1	17.277062000	7.807067000	4.727794000
1	15.523017000	7.891903000	5.055225000
6	12.991034000	9.912511000	10.051517000
1	13.221463000	10.984926000	9.962700000
6	12.944380000	9.558215000	11.539945000
1	12.708708000	8.492222000	11.699497000
1	12.162897000	10.146910000	12.044969000
1	13.905691000	9.782198000	12.027580000
6	11.640916000	9.668053000	9.370895000
1	11.699986000	9.898165000	8.295324000
1	10.872131000	10.316548000	9.818749000
1	11.303829000	8.624505000	9.489305000
7	14.920076000	14.441713000	4.958434000
6	14.564569000	14.458424000	3.546917000
1	15.000708000	15.331580000	3.031778000
1	13.468759000	14.507104000	3.404002000

6	15.124343000	13.153080000	2.963006000
1	14.468023000	12.764761000	2.169276000
1	16.120294000	13.316811000	2.508794000
7	15.179181000	12.229574000	4.085932000
6	15.396146000	10.801581000	3.891400000
1	15.419292000	10.388105000	4.910390000
6	14.211886000	10.150445000	3.174502000
1	13.283113000	10.357572000	3.728320000
1	14.345576000	9.058438000	3.119028000
1	14.094745000	10.525326000	2.144075000
6	16.745987000	10.512649000	3.224652000
1	16.810342000	10.969229000	2.223071000
1	16.903453000	9.430491000	3.108725000
1	17.569273000	10.915591000	3.835431000
6	14.755441000	15.620078000	5.803545000
1	14.895432000	15.262656000	6.836829000
6	15.835860000	16.677598000	5.548447000
1	16.834462000	16.268118000	5.760652000
1	15.678644000	17.553479000	6.197187000
1	15.820487000	17.030031000	4.503548000
6	13.343267000	16.201273000	5.686868000
1	13.174241000	16.643663000	4.691334000
1	13.185162000	16.993919000	6.433819000
1	12.586426000	15.417657000	5.845692000
6	17.981307000	13.819995000	4.849708000
1	18.734128000	14.461742000	5.327467000
1	17.375760000	14.428055000	4.168323000
1	18.510687000	13.047571000	4.267528000
6	20.832885000	12.247699000	8.649681000
6	21.698185000	13.017744000	7.852518000
1	21.279182000	13.573268000	7.012630000
6	23.063896000	13.059420000	8.135854000
1	23.730056000	13.662633000	7.514001000
6	23.582115000	12.327084000	9.210996000
1	24.651788000	12.363241000	9.434841000
6	22.728678000	11.548219000	10.001480000
1	23.133606000	10.967608000	10.834369000
6	21.361265000	11.511629000	9.725456000
1	20.680987000	10.910338000	10.329357000
6	16.941713000	9.745219000	10.552666000
1	17.687077000	8.999893000	10.228841000
1	16.084138000	9.224011000	10.996235000
1	17.413158000	10.385966000	11.311185000
6	13.348707000	13.166256000	8.102440000
1	13.451131000	14.181594000	7.713179000
8	14.068667000	12.797066000	9.095708000
1	13.693292000	11.380219000	6.670645000

TS3

27	15.128347000	11.783543000	7.179870000
1	12.218223000	10.966951000	7.080698000
15	15.630561000	13.032717000	5.498594000
7	17.319316000	13.325017000	5.838752000
6	17.837494000	12.663579000	6.912800000
7	19.124106000	12.787838000	7.227195000
6	19.560872000	12.089430000	8.285856000
7	18.791180000	11.239513000	8.982309000
6	17.519602000	11.145392000	8.611497000
7	16.956870000	11.882639000	7.606011000
7	16.688983000	10.247968000	9.212296000
15	15.173955000	10.006820000	8.377254000
7	14.128163000	9.460278000	9.551289000
6	13.699951000	8.075351000	9.371621000
1	12.716475000	8.023254000	8.869461000
1	13.608741000	7.551982000	10.337814000
6	14.774239000	7.423188000	8.490472000
1	15.584367000	6.995624000	9.113375000
1	14.346339000	6.603075000	7.892986000
7	15.249538000	8.504610000	7.635946000
6	16.199679000	8.286076000	6.544701000
1	16.435203000	9.300092000	6.179363000
6	17.518450000	7.651466000	7.002805000
1	18.012884000	8.276228000	7.760654000
1	18.208536000	7.553226000	6.150299000
1	17.368024000	6.646495000	7.431917000
6	15.540370000	7.530294000	5.388742000
1	15.230737000	6.514793000	5.687253000
1	16.236026000	7.433223000	4.540267000
1	14.645450000	8.071699000	5.046325000
6	13.399333000	10.401685000	10.411156000
1	13.738845000	11.401839000	10.098517000
6	13.758908000	10.192107000	11.884919000
1	13.488942000	9.176337000	12.220790000
1	13.215751000	10.906977000	12.522240000
1	14.837242000	10.336006000	12.051357000
6	11.889347000	10.349690000	10.177072000
1	11.655493000	10.501737000	9.113677000
1	11.393299000	11.146837000	10.750244000
1	11.458695000	9.388380000	10.504000000
7	15.119439000	14.545483000	4.978166000
6	14.641240000	14.573031000	3.602239000
1	14.921801000	15.514878000	3.103850000
1	13.539926000	14.475039000	3.548136000
6	15.313949000	13.381104000	2.914466000
1	14.695064000	13.003828000	2.087142000
1	16.293615000	13.672418000	2.486958000
7	15.444790000	12.381248000	3.964291000

6	15.817679000	10.998821000	3.667265000
1	15.716509000	10.474183000	4.630743000
6	14.835731000	10.353671000	2.690548000
1	13.803025000	10.468568000	3.055263000
1	15.052725000	9.278760000	2.591340000
1	14.903503000	10.799650000	1.685046000
6	17.272250000	10.863340000	3.198711000
1	17.453673000	11.442920000	2.278710000
1	17.515100000	9.810523000	2.982059000
1	17.968889000	11.222670000	3.971100000
6	14.722015000	15.576949000	5.945080000
1	15.312789000	15.365485000	6.852617000
6	15.111404000	16.971936000	5.449346000
1	16.169583000	17.007947000	5.144725000
1	14.949582000	17.713386000	6.246854000
1	14.495608000	17.278459000	4.588112000
6	13.240276000	15.497700000	6.329396000
1	12.592025000	15.692350000	5.458754000
1	13.004212000	16.257678000	7.091206000
1	13.017230000	14.507625000	6.759544000
6	18.192817000	14.145460000	5.012260000
1	18.805649000	14.807416000	5.639987000
1	17.566611000	14.758158000	4.353096000
1	18.871837000	13.525841000	4.403908000
6	20.966784000	12.258120000	8.706556000
6	21.794388000	13.196044000	8.061996000
1	21.379871000	13.775758000	7.236409000
6	23.110872000	13.381798000	8.483644000
1	23.743437000	14.118711000	7.982346000
6	23.622943000	12.628236000	9.547231000
1	24.650997000	12.784660000	9.885113000
6	22.811694000	11.679248000	10.181971000
1	23.210780000	11.082116000	11.006296000
6	21.492376000	11.495708000	9.767385000
1	20.845181000	10.767316000	10.257369000
6	17.197619000	9.396928000	10.274911000
1	17.936360000	8.670050000	9.900372000
1	16.351973000	8.859148000	10.720188000
1	17.684182000	10.007902000	11.048427000
6	12.922931000	11.768302000	6.792591000
1	12.510161000	12.303913000	5.917792000
8	13.318440000	12.568985000	7.816135000
1	13.774707000	11.121596000	6.283727000

TS4

27	17.135371000	11.057731000	7.427789000
1	18.271037000	9.199224000	4.660756000
15	17.315164000	12.940353000	6.426231000

7	19.005177000	13.326416000	6.785007000
6	19.671456000	12.469964000	7.597479000
7	20.914929000	12.749334000	7.997466000
6	21.444826000	11.923602000	8.908226000
7	20.780676000	10.909092000	9.480850000
6	19.545042000	10.683298000	9.025717000
7	18.983549000	11.369722000	7.998862000
7	18.752109000	9.752623000	9.625472000
15	17.094593000	9.658974000	9.067457000
7	16.157067000	9.788681000	10.474109000
6	15.230390000	8.657058000	10.570464000
1	14.299186000	8.853839000	10.003661000
1	14.951448000	8.458562000	11.614565000
6	15.968958000	7.465741000	9.973271000
1	16.585693000	6.961775000	10.744062000
1	15.257398000	6.723606000	9.579134000
7	16.778160000	8.030239000	8.908007000
6	17.471295000	7.169887000	7.946027000
1	18.026726000	7.853493000	7.288480000
6	18.469709000	6.229079000	8.631131000
1	19.207533000	6.788740000	9.223615000
1	19.014154000	5.635559000	7.879443000
1	17.953985000	5.521555000	9.301955000
6	16.481095000	6.392685000	7.074804000
1	15.919989000	5.650238000	7.666409000
1	17.019998000	5.844889000	6.285746000
1	15.765808000	7.081904000	6.607739000
6	15.683132000	11.104087000	10.945876000
1	14.945051000	11.508059000	10.224838000
6	16.840822000	12.097403000	11.033605000
1	17.609551000	11.742857000	11.738293000
1	16.463182000	13.070699000	11.383408000
1	17.302275000	12.251123000	10.048391000
6	14.997069000	10.970651000	12.308369000
1	14.072194000	10.377646000	12.260987000
1	14.727903000	11.968221000	12.688387000
1	15.677416000	10.495196000	13.034445000
7	16.529731000	14.363254000	6.812708000
6	15.923404000	15.056861000	5.685961000
1	16.052249000	16.147117000	5.778553000
1	14.841217000	14.844696000	5.624806000
6	16.648771000	14.544476000	4.434287000
1	15.955108000	14.496910000	3.580365000
1	17.472286000	15.228173000	4.147773000
7	17.134483000	13.217218000	4.785834000
6	17.778042000	12.347025000	3.803580000
1	17.932968000	11.396330000	4.332186000
6	16.859746000	12.067178000	2.613207000
1	15.879907000	11.708995000	2.962220000
1	17.304815000	11.295266000	1.966442000

1	16.707883000	12.967029000	1.995157000
6	19.156151000	12.852636000	3.355651000
1	19.091948000	13.844816000	2.879281000
1	19.593605000	12.159756000	2.620033000
1	19.848350000	12.921908000	4.206024000
6	16.244814000	14.756575000	8.193153000
1	16.882289000	14.110900000	8.817801000
6	16.657434000	16.210424000	8.445993000
1	17.711254000	16.378314000	8.173263000
1	16.520282000	16.466100000	9.508283000
1	16.037743000	16.909138000	7.861397000
6	14.792082000	14.501952000	8.597736000
1	14.095239000	15.095236000	7.987027000
1	14.631466000	14.778496000	9.652462000
1	14.532041000	13.444573000	8.468164000
6	19.635810000	14.585723000	6.414280000
1	19.950011000	15.145688000	7.307961000
1	18.913318000	15.189626000	5.855948000
1	20.526071000	14.418614000	5.789959000
6	22.838645000	12.182707000	9.336170000
6	23.560731000	13.248542000	8.768828000
1	23.077047000	13.856193000	8.003279000
6	24.862329000	13.519864000	9.189372000
1	25.415838000	14.350806000	8.744717000
6	25.458630000	12.729215000	10.179351000
1	26.473502000	12.952804000	10.519475000
6	24.751356000	11.657385000	10.738077000
1	25.218494000	11.034534000	11.505183000
6	23.448404000	11.383336000	10.319176000
1	22.880097000	10.557831000	10.749640000
6	19.237761000	9.054690000	10.807391000
1	20.156994000	8.495328000	10.580482000
1	18.462283000	8.359014000	11.146377000
1	19.461567000	9.764212000	11.619306000
6	17.188450000	9.075067000	4.770199000
1	16.689789000	9.647602000	3.981924000
8	16.766956000	9.466598000	6.049563000
1	16.943327000	8.017345000	4.610771000
6	12.215580000	13.375247000	6.385540000
6	13.270754000	12.526970000	6.024370000
6	11.551216000	13.186382000	7.602368000
1	13.779081000	12.678993000	5.065639000
1	10.728681000	13.845836000	7.892571000
6	13.694817000	11.481192000	6.858973000
6	11.946519000	12.140093000	8.446433000
1	11.426984000	11.975789000	9.395341000
6	13.002674000	11.304700000	8.071946000
1	13.303451000	10.493354000	8.743795000
1	11.907989000	14.184282000	5.715907000
14	15.182315000	10.324195000	6.393239000

1	14.587133000	9.095492000	7.006749000
1	14.986183000	10.515542000	4.910880000
1	15.852907000	11.515745000	7.937553000

Phenylsilane

6	0.745117000	0.001748000	-1.941465000
6	-0.465778000	-0.020968000	-1.243514000
6	1.955731000	0.022615000	-1.239218000
1	-1.412502000	-0.039288000	-1.789454000
1	2.903517000	0.040298000	-1.783429000
6	-0.465688000	-0.023185000	0.155082000
6	1.951656000	0.020351000	0.157543000
1	-1.418981000	-0.044084000	0.690339000
1	2.905928000	0.035692000	0.693043000
6	0.741308000	-0.002360000	0.877894000
1	0.746928000	0.002077000	-3.034594000
14	0.741650000	0.001419000	2.744891000
1	1.583114000	-1.101818000	3.271493000
1	1.293709000	1.274455000	3.279893000
1	-0.647310000	-0.166954000	3.240270000

CO_2

6	0.000000000	0.000000000	0.000000000
8	0.000000000	0.000000000	1.159825000
8	0.000000000	0.000000000	-1.159825000

Phenylsilyl formate 13

6	1.063484000	-0.345407000	-2.007479000
6	-0.254626000	-0.210432000	-1.560866000
6	2.118561000	-0.310485000	-1.089096000
1	-1.080797000	-0.239398000	-2.275860000
1	3.149180000	-0.419011000	-1.437101000
6	-0.519674000	-0.037323000	-0.199258000
6	1.851781000	-0.136528000	0.271093000
1	-1.552916000	0.071294000	0.138375000
1	2.688276000	-0.106163000	0.976793000
6	0.529971000	0.004335000	0.736784000
1	1.270852000	-0.480738000	-3.072345000
14	0.226475000	0.246992000	2.551414000
1	0.586348000	-0.945852000	3.348851000
1	0.937154000	1.432378000	3.078006000
8	-1.454852000	0.501439000	2.666076000
6	-2.046769000	0.759076000	3.832748000

1	-3.129553000	0.886166000	3.710478000
8	-1.471928000	0.848468000	4.886594000

Bis(phenylsilyl)acetal 22

1	17.885475000	6.169324000	2.622519000
6	14.953271000	1.982717000	1.834903000
6	14.970357000	2.870761000	0.753589000
6	14.988295000	2.474074000	3.142551000
1	14.941049000	2.486133000	-0.269453000
1	14.976455000	1.781477000	3.987897000
6	15.021979000	4.246260000	0.984955000
6	15.041555000	3.852988000	3.369378000
1	15.036367000	4.931944000	0.132246000
1	15.071877000	4.226704000	4.397237000
6	15.057751000	4.761427000	2.296171000
1	14.912237000	0.905171000	1.654420000
14	15.114719000	6.602799000	2.573533000
1	13.915120000	7.265466000	2.016610000
1	15.238918000	6.862693000	4.029629000
8	16.372431000	7.266181000	1.715966000
6	17.736108000	7.055310000	1.997780000
1	18.241446000	6.915457000	1.037600000
8	18.283830000	8.146625000	2.678758000
6	15.135444000	10.152299000	2.457901000
6	16.404654000	10.422174000	3.005773000
6	13.974686000	10.665364000	3.040963000
1	12.999906000	10.430720000	2.604274000
6	16.471698000	11.246538000	4.144007000
6	14.062274000	11.476394000	4.178272000
1	17.443968000	11.486479000	4.584739000
1	13.156260000	11.882336000	4.636669000
6	15.313801000	11.773559000	4.725138000
1	15.388566000	12.412433000	5.609041000
1	15.055857000	9.530751000	1.563675000
14	17.965832000	9.725092000	2.259211000
1	17.916408000	9.838385000	0.777485000
1	19.108022000	10.489869000	2.810266000

Formaldehyde

8	-1.593318000	-0.000007000	-5.681296000
6	-1.593315000	-0.000001000	-4.481956000
1	-1.593314000	0.938745000	-3.893824000
1	-1.593314000	-0.938737000	-3.893812000

Phenylsilyl methoxide 30

6	-1.743671000	-0.224870000	-7.884386000
6	-2.912248000	-0.018189000	-7.142291000
6	-0.510433000	-0.315390000	-7.232103000
1	-3.877416000	0.049676000	-7.651172000
1	0.402611000	-0.485293000	-7.808368000
6	-2.842184000	0.102767000	-5.752129000
6	-0.446118000	-0.201434000	-5.839464000
1	-3.764235000	0.271262000	-5.186099000
1	0.520975000	-0.291603000	-5.338221000
6	-1.609196000	0.011455000	-5.078166000
1	-1.796835000	-0.322898000	-8.971839000
14	-1.537202000	0.147754000	-3.218715000
1	-2.038622000	1.484186000	-2.788053000
1	-2.373138000	-0.903541000	-2.592877000
8	-0.003842000	-0.070633000	-2.651554000
6	0.973002000	0.962759000	-2.610099000
1	0.556812000	1.883848000	-2.190501000
1	1.793351000	0.626874000	-1.976581000
1	1.362428000	1.177927000	-3.608893000

1,3-Diphenyldisiloxane

6	-1.799468000	0.164653000	-7.743935000
6	-2.948743000	0.122824000	-6.946136000
6	-0.535097000	0.113556000	-7.149209000
1	-3.937945000	0.166800000	-7.409690000
1	0.363178000	0.149318000	-7.770860000
6	-2.828057000	0.026204000	-5.557818000
6	-0.418342000	0.017490000	-5.758542000
1	-3.736130000	-0.006117000	-4.946814000
1	0.573247000	-0.023064000	-5.300773000
6	-1.562292000	-0.029988000	-4.942708000
1	-1.892350000	0.240152000	-8.830684000
14	-1.442618000	-0.155730000	-3.089017000
1	-2.095051000	1.017004000	-2.450975000
1	-2.142315000	-1.374776000	-2.604321000
8	0.139265000	-0.227421000	-2.648743000
6	-0.367372000	2.141735000	0.162437000
6	0.615685000	1.958752000	-0.829494000
6	-0.780898000	3.422994000	0.538400000
1	-1.546955000	3.549885000	1.307939000
6	1.177665000	3.099969000	-1.433200000
6	-0.210767000	4.545573000	-0.072743000
1	1.942959000	2.982821000	-2.206441000
1	-0.534873000	5.547002000	0.224431000
6	0.770036000	4.383955000	-1.058601000
1	1.218728000	5.258646000	-1.536937000

14	1.086800000	0.248128000	-1.387825000
1	0.892903000	-0.698619000	-0.260332000
1	2.486820000	0.206938000	-1.871900000
1	-0.819590000	1.271623000	0.647655000

32

27	14.687845000	11.289480000	6.938389000
15	14.916409000	12.760708000	5.414597000
7	16.691334000	12.881761000	5.390220000
6	17.351341000	12.172923000	6.343409000
7	18.688437000	12.187342000	6.408084000
6	19.238495000	11.492222000	7.414783000
7	18.543744000	10.842105000	8.358547000
6	17.212989000	10.866885000	8.237233000
7	16.570337000	11.477177000	7.209080000
7	16.414852000	10.267479000	9.159758000
15	14.706651000	10.112266000	8.696282000
7	13.920446000	10.381271000	10.148436000
6	13.264116000	9.198986000	10.696871000
1	12.186308000	9.192403000	10.451782000
1	13.366468000	9.161122000	11.794292000
6	13.953609000	7.994811000	10.044660000
1	14.808569000	7.646137000	10.656166000
1	13.251418000	7.152559000	9.953271000
7	14.364894000	8.475144000	8.732495000
6	14.863641000	7.583391000	7.684750000
1	14.930755000	8.220124000	6.790923000
6	16.267124000	7.033687000	7.974291000
1	17.004107000	7.847949000	8.039247000
1	16.584849000	6.352234000	7.169827000
1	16.293111000	6.467111000	8.919969000
6	13.860569000	6.476258000	7.367497000
1	13.769249000	5.754685000	8.196085000
1	14.186899000	5.920965000	6.475323000
1	12.871297000	6.908300000	7.154741000
6	13.518573000	11.727322000	10.562932000
1	14.067140000	12.414122000	9.895542000
6	13.955855000	12.011034000	12.003501000
1	13.453947000	11.330580000	12.710693000
1	13.692119000	13.040211000	12.295035000
1	15.043145000	11.880306000	12.117264000
6	12.022263000	11.985470000	10.362256000
1	11.724349000	11.752500000	9.329626000
1	11.783085000	13.040058000	10.572162000
1	11.413307000	11.368857000	11.042714000
7	14.420804000	14.357566000	5.605995000
6	13.729469000	14.911645000	4.446877000
1	14.009918000	15.965843000	4.284142000

1	12.632693000	14.867300000	4.576321000
6	14.148012000	14.049108000	3.252869000
1	13.332600000	13.980443000	2.516923000
1	15.018355000	14.490092000	2.729705000
7	14.447561000	12.739375000	3.814223000
6	14.527493000	11.548128000	2.970118000
1	14.799693000	10.729189000	3.651119000
6	13.165724000	11.207680000	2.358998000
1	12.400470000	11.146884000	3.145025000
1	13.206874000	10.232783000	1.849853000
1	12.857989000	11.962964000	1.617298000
6	15.623569000	11.663442000	1.906595000
1	15.402913000	12.472610000	1.190525000
1	15.704224000	10.727179000	1.332613000
1	16.599682000	11.873375000	2.370224000
6	14.127448000	14.874977000	6.946976000
1	14.743740000	14.269215000	7.632973000
6	14.562530000	16.335942000	7.079266000
1	15.629533000	16.453458000	6.832530000
1	14.394651000	16.694800000	8.106956000
1	13.983134000	16.988851000	6.406376000
6	12.660998000	14.676418000	7.348886000
1	11.986444000	15.289910000	6.731114000
1	12.505607000	14.970609000	8.399187000
1	12.372869000	13.621346000	7.233589000
6	17.450346000	13.745365000	4.500528000
1	18.099572000	14.425362000	5.072898000
1	16.754978000	14.344228000	3.902788000
1	18.089782000	13.156037000	3.824045000
6	20.718047000	11.424942000	7.491217000
6	21.514617000	12.106315000	6.553662000
1	21.020439000	12.687907000	5.774801000
6	22.906325000	12.030233000	6.625646000
1	23.519710000	12.562743000	5.894608000
6	23.518274000	11.272305000	7.631912000
1	24.608510000	11.212288000	7.687302000
6	22.732438000	10.587766000	8.566858000
1	23.209233000	9.992689000	9.350057000
6	21.341250000	10.664849000	8.498881000
1	20.712917000	10.137433000	9.216856000
6	16.984802000	9.722788000	10.380250000
1	17.390416000	8.707431000	10.236313000
1	16.194073000	9.689117000	11.141032000
1	17.799213000	10.367644000	10.738093000
14	12.567279000	10.707986000	6.408191000
1	11.622836000	10.261218000	7.501415000
1	11.680350000	11.721822000	5.717364000
6	11.161705000	7.622522000	3.898056000
6	12.329096000	7.065073000	3.362420000
6	11.243872000	8.683012000	4.806807000

1	12.267062000	6.235400000	2.652711000
1	10.321573000	9.107321000	5.217363000
6	13.572136000	7.578222000	3.744686000
6	12.483086000	9.223143000	5.202750000
1	14.490647000	7.148981000	3.333298000
6	13.638757000	8.642505000	4.650435000
1	14.616745000	9.039229000	4.937040000
1	10.186196000	7.226007000	3.599724000

Formic acid

6	14.675169000	14.102712000	5.627989000
8	14.667001000	14.091344000	4.431250000
8	14.890123000	12.990689000	6.349372000
1	14.506352000	15.004135000	6.236860000
1	14.853014000	13.191622000	7.295893000

33

27	14.777295000	11.426499000	7.269842000
1	16.133426000	12.793341000	9.608766000
15	15.034511000	12.725096000	5.528838000
7	16.760092000	13.084604000	5.717871000
6	17.464632000	12.371655000	6.633018000
7	18.796199000	12.484461000	6.716507000
6	19.392848000	11.737733000	7.656776000
7	18.755373000	10.880861000	8.468176000
6	17.428953000	10.813426000	8.320895000
7	16.746590000	11.555943000	7.428654000
7	16.686086000	9.971759000	9.087006000
15	14.981635000	9.801178000	8.683641000
7	14.269819000	9.552226000	10.163719000
6	13.603153000	8.260072000	10.280946000
1	12.517182000	8.354601000	10.115843000
1	13.756494000	7.838586000	11.285681000
6	14.237135000	7.348011000	9.223244000
1	15.026013000	6.719900000	9.675900000
1	13.483889000	6.670190000	8.790914000
7	14.782658000	8.228912000	8.201865000
6	15.358261000	7.684774000	6.970157000
1	15.886650000	8.521418000	6.486813000
6	16.389462000	6.591075000	7.263582000
1	17.157441000	6.951742000	7.964565000
1	16.890843000	6.277301000	6.335136000
1	15.914477000	5.698612000	7.702533000
6	14.261161000	7.213296000	6.013222000
1	13.625396000	6.448255000	6.487196000
1	14.695774000	6.772969000	5.102253000

1	13.622086000	8.057817000	5.721457000
6	13.953172000	10.646374000	11.095166000
1	14.696549000	11.432105000	10.892965000
6	14.147517000	10.196711000	12.545683000
1	13.386808000	9.458867000	12.845443000
1	14.044872000	11.062617000	13.217126000
1	15.142157000	9.748139000	12.697124000
6	12.559995000	11.241141000	10.872011000
1	12.468127000	11.664359000	9.863909000
1	12.377755000	12.054941000	11.589669000
1	11.772431000	10.484064000	11.015225000
7	14.388752000	14.216503000	5.208774000
6	13.975405000	14.425326000	3.824733000
1	14.391342000	15.365326000	3.429092000
1	12.877386000	14.497383000	3.745612000
6	14.506129000	13.228049000	3.015203000
1	13.740302000	12.878509000	2.308414000
1	15.396942000	13.516655000	2.427111000
7	14.818860000	12.174614000	3.977131000
6	15.280561000	10.856847000	3.518829000
1	15.149436000	10.187196000	4.383562000
6	14.406193000	10.318865000	2.386875000
1	13.343552000	10.337632000	2.664884000
1	14.689739000	9.277807000	2.168269000
1	14.542675000	10.898472000	1.459722000
6	16.765815000	10.856064000	3.128115000
1	16.969771000	11.598285000	2.339396000
1	17.061736000	9.869156000	2.738607000
1	17.409270000	11.087365000	3.989332000
6	14.128362000	15.226640000	6.249088000
1	14.694740000	14.898667000	7.131701000
6	14.644651000	16.606522000	5.831939000
1	15.694258000	16.563621000	5.500176000
1	14.581796000	17.295640000	6.687528000
1	14.042805000	17.039726000	5.016457000
6	12.652203000	15.270496000	6.650155000
1	12.010279000	15.563255000	5.802746000
1	12.497854000	16.004431000	7.455667000
1	12.326945000	14.291808000	7.027353000
6	17.468871000	13.995830000	4.829582000
1	18.103986000	14.677612000	5.412348000
1	16.733413000	14.588441000	4.275644000
1	18.106947000	13.449127000	4.118285000
6	20.860658000	11.867957000	7.816810000
6	21.593831000	12.727529000	6.979637000
1	21.062737000	13.281187000	6.204953000
6	22.970927000	12.869055000	7.151463000
1	23.535319000	13.540228000	6.499538000
6	23.629368000	12.155158000	8.160198000
1	24.705950000	12.279310000	8.304645000

6	22.907684000	11.289463000	8.990871000
1	23.422413000	10.728625000	9.774941000
6	21.531088000	11.144316000	8.819660000
1	20.951908000	10.479990000	9.461335000
6	17.350513000	9.111126000	10.055174000
1	18.028357000	8.402087000	9.555098000
1	16.584465000	8.560516000	10.613161000
1	17.940216000	9.713509000	10.761011000
1	14.838039000	10.340455000	6.297270000
14	12.572863000	11.124424000	7.039866000
1	11.810784000	12.212519000	7.709223000
6	10.950574000	12.031601000	3.319190000
6	10.564974000	10.790895000	2.798483000
6	11.549420000	12.101891000	4.578359000
1	10.085991000	10.730235000	1.817890000
1	11.821319000	13.075206000	4.989126000
6	10.778957000	9.629956000	3.547713000
6	11.786772000	10.945915000	5.342165000
1	10.460758000	8.660897000	3.152877000
6	11.388052000	9.710570000	4.804969000
1	11.535163000	8.795319000	5.383482000
1	10.769540000	12.945743000	2.747156000
6	15.240075000	13.417916000	9.401771000
8	15.086094000	14.450771000	10.049700000
8	14.451370000	12.982426000	8.494042000
8	12.042803000	9.663103000	7.831038000
6	10.793358000	9.407968000	8.180080000
8	9.846646000	10.143132000	8.048951000
1	10.706082000	8.406760000	8.628771000

34

27	14.745082000	11.614760000	7.602021000
1	14.902827000	14.151740000	8.604748000
15	14.984266000	12.356254000	5.673987000
7	16.764391000	12.612510000	5.646726000
6	17.436043000	12.313974000	6.783304000
7	18.764859000	12.464589000	6.882943000
6	19.312603000	12.079923000	8.044576000
7	18.663687000	11.415213000	9.012573000
6	17.350591000	11.238208000	8.814452000
7	16.682450000	11.838023000	7.800824000
7	16.616730000	10.373286000	9.552031000
15	15.198514000	9.727554000	8.618243000
7	14.317348000	8.896309000	9.760057000
6	14.436047000	7.443095000	9.655466000
1	13.576215000	7.015804000	9.108294000
1	14.470880000	6.970068000	10.649999000
6	15.726219000	7.192443000	8.875654000

1	16.594473000	7.160819000	9.560802000
1	15.689843000	6.230874000	8.342299000
7	15.806377000	8.310229000	7.938336000
6	16.851856000	8.334017000	6.909869000
1	16.735902000	9.306721000	6.402690000
6	18.280734000	8.244007000	7.466865000
1	18.513865000	9.052544000	8.171174000
1	19.006747000	8.301262000	6.640815000
1	18.444413000	7.283914000	7.983568000
6	16.614683000	7.231840000	5.874131000
1	16.825257000	6.236614000	6.298792000
1	17.279146000	7.369403000	5.008082000
1	15.573237000	7.244664000	5.521273000
6	13.160494000	9.503965000	10.438112000
1	13.236401000	10.582763000	10.244518000
6	13.227811000	9.292335000	11.951831000
1	13.113325000	8.228545000	12.220984000
1	12.417386000	9.849238000	12.445496000
1	14.184212000	9.654943000	12.359112000
6	11.825650000	9.018601000	9.865237000
1	11.805532000	9.135723000	8.771092000
1	11.005791000	9.617406000	10.289116000
1	11.631008000	7.961064000	10.110620000
7	14.452204000	13.771875000	4.945960000
6	14.009928000	13.661053000	3.563906000
1	14.505357000	14.408911000	2.919614000
1	12.923070000	13.832354000	3.488088000
6	14.364992000	12.237069000	3.104538000
1	13.535460000	11.800165000	2.526819000
1	15.249816000	12.243589000	2.441256000
7	14.603357000	11.466290000	4.314840000
6	14.543350000	10.007850000	4.294268000
1	14.884512000	9.689978000	5.291068000
6	13.106361000	9.502554000	4.126846000
1	12.477212000	9.873867000	4.947545000
1	13.073993000	8.401345000	4.142151000
1	12.665687000	9.835410000	3.172682000
6	15.486798000	9.431980000	3.235643000
1	15.165415000	9.719765000	2.220961000
1	15.503414000	8.332856000	3.279062000
1	16.513006000	9.803713000	3.387177000
6	14.439733000	15.068061000	5.622065000
1	14.497336000	14.842471000	6.695396000
6	15.656979000	15.930243000	5.262201000
1	16.590718000	15.423366000	5.545507000
1	15.616821000	16.893821000	5.794487000
1	15.693888000	16.145507000	4.181136000
6	13.123449000	15.813186000	5.386066000
1	13.033937000	16.160813000	4.343746000
1	13.072615000	16.699612000	6.036293000

1	12.265051000	15.167702000	5.612954000
6	17.484714000	13.029366000	4.455665000
1	18.320418000	13.691217000	4.720141000
1	16.798448000	13.572893000	3.796192000
1	17.892876000	12.163233000	3.908049000
6	20.750675000	12.372868000	8.257391000
6	21.453682000	13.180952000	7.346499000
1	20.923467000	13.572183000	6.477290000
6	22.798661000	13.481375000	7.565210000
1	23.335931000	14.117676000	6.857290000
6	23.458129000	12.969088000	8.689366000
1	24.509140000	13.213410000	8.865940000
6	22.768851000	12.148759000	9.590629000
1	23.286063000	11.738271000	10.461591000
6	21.421751000	11.853753000	9.378758000
1	20.866137000	11.221314000	10.072261000
6	17.191207000	9.751777000	10.732238000
1	17.972893000	9.012950000	10.487432000
1	16.386084000	9.248867000	11.283532000
1	17.646344000	10.519296000	11.375777000
1	13.298670000	11.316419000	7.286697000
14	11.510639000	12.936265000	8.398323000
1	11.578258000	11.534599000	8.785226000
6	9.229329000	14.573159000	5.333057000
6	9.651476000	13.853117000	4.210476000
6	9.758096000	14.276877000	6.593043000
1	9.236410000	14.088458000	3.226578000
1	9.417575000	14.843854000	7.462334000
6	10.600958000	12.834469000	4.353619000
6	10.728577000	13.268569000	6.750344000
1	10.927777000	12.260121000	3.483335000
6	11.141473000	12.552674000	5.610956000
1	11.911240000	11.786183000	5.712990000
1	8.484876000	15.366428000	5.225217000
6	14.162357000	13.370121000	8.777163000
8	14.358698000	12.436626000	9.580352000
8	12.893809000	13.839462000	8.518543000
8	10.431505000	13.690403000	9.491962000
6	10.471180000	13.451569000	10.801428000
8	11.245528000	12.710857000	11.350692000
1	9.697026000	14.021959000	11.331367000

10

27	14.798950000	11.399057000	7.329155000
1	14.399426000	12.458634000	8.344755000
15	14.943447000	12.782376000	5.728304000
7	16.655320000	13.256969000	5.912418000
6	17.394646000	12.565299000	6.812843000

7	18.719548000	12.745854000	6.914892000
6	19.348568000	11.981799000	7.818010000
7	18.752459000	11.060201000	8.588087000
6	17.428022000	10.942328000	8.437896000
7	16.712438000	11.690217000	7.576796000
7	16.718605000	10.032753000	9.156530000
15	15.013810000	9.868093000	8.755508000
7	14.284783000	9.715739000	10.249801000
6	13.498758000	8.496230000	10.385025000
1	12.446271000	8.657730000	10.090080000
1	13.510003000	8.135419000	11.425346000
6	14.165856000	7.476653000	9.454095000
1	14.912991000	6.876262000	10.008031000
1	13.420526000	6.776862000	9.042201000
7	14.784413000	8.258853000	8.395273000
6	15.341725000	7.615371000	7.203925000
1	16.004575000	8.361847000	6.739143000
6	16.193243000	6.395699000	7.572254000
1	16.967698000	6.660252000	8.308300000
1	16.693015000	5.994014000	6.677135000
1	15.577141000	5.586807000	7.997895000
6	14.251835000	7.275115000	6.184785000
1	13.485343000	6.617822000	6.627108000
1	14.679261000	6.756641000	5.311353000
1	13.762446000	8.195579000	5.838001000
6	14.087818000	10.861900000	11.140533000
1	14.777256000	11.638866000	10.771989000
6	14.495430000	10.508472000	12.575561000
1	13.812643000	9.763067000	13.014783000
1	14.457578000	11.403770000	13.215652000
1	15.515628000	10.094387000	12.605490000
6	12.668255000	11.433945000	11.081630000
1	12.404883000	11.730674000	10.056641000
1	12.587341000	12.321527000	11.728433000
1	11.928265000	10.697951000	11.436090000
7	14.193650000	14.254204000	5.522740000
6	13.766899000	14.561325000	4.165004000
1	14.130425000	15.552209000	3.846132000
1	12.665393000	14.581526000	4.089234000
6	14.354824000	13.463082000	3.256567000
1	13.597638000	13.125544000	2.533140000
1	15.214090000	13.852952000	2.678264000
7	14.747991000	12.362499000	4.126011000
6	15.268543000	11.112239000	3.560774000
1	15.212676000	10.385880000	4.386581000
6	14.387466000	10.583768000	2.427991000
1	13.338972000	10.504566000	2.748624000
1	14.736918000	9.582548000	2.130942000
1	14.439901000	11.226019000	1.533528000
6	16.736582000	11.232061000	3.124104000

1	16.868192000	12.021818000	2.365874000
1	17.087257000	10.286178000	2.681043000
1	17.387263000	11.467121000	3.979181000
6	13.911585000	15.149933000	6.646969000
1	14.486651000	14.742265000	7.493609000
6	14.412508000	16.572556000	6.373880000
1	15.465367000	16.568929000	6.050669000
1	14.331574000	17.183548000	7.286138000
1	13.815896000	17.069803000	5.591913000
6	12.432752000	15.132763000	7.047194000
1	11.790385000	15.486947000	6.224087000
1	12.259291000	15.796066000	7.909275000
1	12.120272000	14.116916000	7.327409000
6	17.300349000	14.240632000	5.059473000
1	17.917192000	14.924009000	5.660732000
1	16.526611000	14.824540000	4.549057000
1	17.948429000	13.764694000	4.306782000
6	20.812777000	12.165251000	7.979601000
6	21.498860000	13.126346000	7.216555000
1	20.934182000	13.722399000	6.499081000
6	22.870979000	13.313186000	7.387537000
1	23.397171000	14.064366000	6.793168000
6	23.574114000	12.538917000	8.318311000
1	24.646674000	12.695535000	8.461770000
6	22.900225000	11.570843000	9.072578000
1	23.448859000	10.960711000	9.794553000
6	21.528106000	11.383375000	8.903983000
1	20.985674000	10.638113000	9.486401000
6	17.402825000	9.136565000	10.073526000
1	18.095682000	8.471266000	9.534023000
1	16.650132000	8.533907000	10.595719000
1	17.980792000	9.705989000	10.816201000
1	15.000115000	10.303068000	6.312885000
14	12.603781000	10.964631000	7.186714000
1	12.202645000	9.662845000	7.823026000
1	11.708949000	11.947595000	7.888632000
6	10.865362000	11.965055000	3.512307000
6	10.498336000	10.732510000	2.958799000
6	11.493191000	12.009628000	4.758454000
1	9.999449000	10.690639000	1.986924000
1	11.757767000	12.976376000	5.188941000
6	10.762309000	9.555270000	3.665529000
6	11.777598000	10.839441000	5.486636000
1	10.462401000	8.590317000	3.245815000
6	11.397180000	9.613119000	4.912444000
1	11.582700000	8.683997000	5.457897000
1	10.652170000	12.892455000	2.972581000

10‘

27	14.744080000	11.979670000	7.522110000
1	13.272697000	12.077733000	7.492880000
15	15.022911000	13.096154000	5.732382000
7	16.794147000	13.282726000	5.803308000
6	17.453893000	12.630762000	6.795523000
7	18.790501000	12.662593000	6.885869000
6	19.326883000	11.994079000	7.917840000
7	18.629254000	11.301241000	8.828689000
6	17.302007000	11.295275000	8.672749000
7	16.679975000	11.956542000	7.670095000
7	16.489351000	10.601142000	9.504365000
15	14.828311000	10.371492000	8.899806000
7	13.917606000	10.009954000	10.241348000
6	13.596648000	8.588300000	10.378844000
1	12.552515000	8.397351000	10.075286000
1	13.716547000	8.251072000	11.421546000
6	14.557310000	7.838846000	9.453101000
1	15.471018000	7.536677000	9.999428000
1	14.090467000	6.920192000	9.064528000
7	14.843570000	8.774612000	8.374180000
6	15.667657000	8.357623000	7.234748000
1	15.903771000	9.285845000	6.693155000
6	16.996148000	7.709935000	7.645508000
1	17.590185000	8.385225000	8.278325000
1	17.593176000	7.477227000	6.749990000
1	16.843188000	6.766051000	8.194077000
6	14.855575000	7.476797000	6.282596000
1	14.510778000	6.555317000	6.780431000
1	15.462253000	7.180623000	5.411967000
1	13.971030000	8.024336000	5.922484000
6	13.122631000	11.013244000	10.963174000
1	13.366904000	11.974983000	10.499304000
6	13.529224000	11.096871000	12.435420000
1	13.352183000	10.141892000	12.958439000
1	12.943194000	11.872625000	12.951680000
1	14.595186000	11.355046000	12.527675000
6	11.617898000	10.804131000	10.787137000
1	11.359360000	10.716855000	9.720514000
1	11.074717000	11.667712000	11.197056000
1	11.259055000	9.903495000	11.311163000
7	14.516358000	14.612702000	5.230631000
6	13.948410000	14.649047000	3.888599000
1	14.251402000	15.564762000	3.355672000
1	12.843523000	14.629730000	3.921270000
6	14.485774000	13.407361000	3.180882000
1	13.771067000	13.044976000	2.427406000
1	15.431841000	13.632100000	2.652862000
7	14.671823000	12.419309000	4.235285000

6	14.983020000	11.035653000	3.874537000
1	15.092399000	10.506992000	4.832865000
6	13.806310000	10.388362000	3.140114000
1	12.891047000	10.476850000	3.745799000
1	14.004556000	9.319858000	2.960234000
1	13.620477000	10.858604000	2.160471000
6	16.308856000	10.905093000	3.115994000
1	16.291014000	11.449400000	2.157553000
1	16.522074000	9.847735000	2.892702000
1	17.140424000	11.300113000	3.719345000
6	14.205156000	15.694202000	6.170378000
1	14.701624000	15.419888000	7.110354000
6	14.806588000	17.026315000	5.709808000
1	15.880978000	16.924007000	5.490524000
1	14.684934000	17.785500000	6.498050000
1	14.305018000	17.408928000	4.805553000
6	12.703003000	15.813004000	6.447132000
1	12.157556000	16.207981000	5.574659000
1	12.520063000	16.493209000	7.292535000
1	12.282241000	14.833817000	6.713426000
6	17.554444000	14.073390000	4.846545000
1	18.261468000	14.730868000	5.372342000
1	16.854140000	14.693376000	4.275909000
1	18.126941000	13.434991000	4.154691000
6	20.803866000	12.000138000	8.061047000
6	21.604514000	12.742451000	7.176128000
1	21.116835000	13.309032000	6.382275000
6	22.992644000	12.748080000	7.321331000
1	23.609606000	13.330487000	6.632756000
6	23.595118000	12.010855000	8.348259000
1	24.682002000	12.020643000	8.465644000
6	22.804602000	11.261297000	9.227624000
1	23.274371000	10.680202000	10.025353000
6	21.416837000	11.258213000	9.087286000
1	20.783964000	10.684344000	9.764352000
6	17.033363000	9.952091000	10.685721000
1	17.588035000	9.031689000	10.439481000
1	16.201370000	9.701174000	11.354979000
1	17.718342000	10.634805000	11.207567000
1	14.592802000	10.863299000	6.545889000
14	14.773380000	13.637969000	9.109234000
1	15.432224000	13.198723000	10.395058000
1	15.665090000	14.810236000	8.781801000
6	12.040736000	16.080449000	11.159570000
6	10.790995000	15.594686000	10.755057000
6	13.210083000	15.510872000	10.645695000
1	9.875611000	16.035846000	11.159972000
1	14.178689000	15.897970000	10.978292000
6	10.725590000	14.543474000	9.834825000
6	13.168270000	14.447830000	9.723227000

1	9.756381000	14.155094000	9.506944000
6	11.902946000	13.982061000	9.328253000
1	11.845691000	13.157103000	8.613338000
1	12.103198000	16.901346000	11.879776000

12

27	14.719109000	11.333607000	7.271899000
1	14.238947000	12.407564000	8.219224000
15	14.883231000	12.782801000	5.734492000
7	16.602405000	13.202828000	5.907063000
6	17.315366000	12.538548000	6.847979000
7	18.630456000	12.745575000	6.999064000
6	19.224283000	12.053827000	7.981376000
7	18.595005000	11.204357000	8.804984000
6	17.283992000	11.041483000	8.589557000
7	16.614255000	11.674345000	7.607307000
7	16.541706000	10.212020000	9.366124000
15	14.856309000	9.965541000	8.880849000
7	14.027157000	10.018833000	10.316234000
6	13.506776000	8.731277000	10.764426000
1	12.417956000	8.668195000	10.590383000
1	13.685717000	8.581205000	11.842423000
6	14.241243000	7.659243000	9.943025000
1	15.108581000	7.254190000	10.498915000
1	13.568352000	6.815515000	9.730408000
7	14.644264000	8.321196000	8.707757000
6	15.184995000	7.578471000	7.564798000
1	15.197431000	8.309721000	6.741544000
6	16.625729000	7.099553000	7.793987000
1	17.299235000	7.950730000	7.972278000
1	16.995406000	6.556415000	6.909842000
1	16.695534000	6.416387000	8.656872000
6	14.265405000	6.432898000	7.139325000
1	14.224159000	5.630120000	7.893805000
1	14.644015000	5.984971000	6.207364000
1	13.243497000	6.799831000	6.958230000
6	13.740807000	11.273803000	11.023463000
1	13.919881000	12.069728000	10.282548000
6	14.702581000	11.490050000	12.198812000
1	14.644828000	10.656915000	12.919647000
1	14.451614000	12.415975000	12.740185000
1	15.741360000	11.572521000	11.846213000
6	12.278099000	11.363683000	11.458562000
1	11.602175000	11.252383000	10.600611000
1	12.086494000	12.346354000	11.916409000
1	12.030589000	10.599285000	12.213451000
7	14.150556000	14.271515000	5.664738000
6	13.720985000	14.700009000	4.341614000

1	14.115988000	15.699926000	4.097438000
1	12.620801000	14.760878000	4.282957000
6	14.261173000	13.658134000	3.341326000
1	13.479697000	13.400503000	2.612741000
1	15.120474000	14.064525000	2.774639000
7	14.637526000	12.482076000	4.117898000
6	15.044554000	11.231503000	3.460123000
1	14.802799000	10.431926000	4.177348000
6	14.241034000	10.975907000	2.185493000
1	13.162155000	11.041246000	2.372356000
1	14.460926000	9.963373000	1.813862000
1	14.505450000	11.684913000	1.384162000
6	16.552999000	11.179002000	3.183695000
1	16.869283000	12.027096000	2.554109000
1	16.817960000	10.251251000	2.652315000
1	17.128605000	11.210341000	4.120059000
6	13.907417000	15.080516000	6.862439000
1	14.515287000	14.617462000	7.655416000
6	14.397107000	16.519974000	6.675818000
1	15.441306000	16.540040000	6.325034000
1	14.341062000	17.065250000	7.630469000
1	13.779656000	17.069975000	5.947293000
6	12.445447000	15.022450000	7.317835000
1	11.767369000	15.402892000	6.536198000
1	12.297107000	15.645004000	8.214122000
1	12.156113000	13.991837000	7.569233000
6	17.267255000	14.180289000	5.060956000
1	17.882089000	14.859060000	5.668903000
1	16.508840000	14.771642000	4.536698000
1	17.921178000	13.694024000	4.319840000
6	20.684573000	12.238082000	8.171983000
6	21.404446000	13.120068000	7.346805000
1	20.867871000	13.659003000	6.565384000
6	22.774998000	13.300258000	7.536082000
1	23.327926000	13.988875000	6.892298000
6	23.442224000	12.599929000	8.548479000
1	24.513673000	12.751802000	8.704759000
6	22.734492000	11.710844000	9.366265000
1	23.255877000	11.156855000	10.150980000
6	21.364027000	11.529436000	9.178990000
1	20.796330000	10.842994000	9.807663000
6	17.158180000	9.525665000	10.491584000
1	17.767594000	8.668542000	10.164778000
1	16.366390000	9.170377000	11.160587000
1	17.806433000	10.216011000	11.048572000
1	14.962793000	10.235820000	6.251154000
14	12.645016000	10.717375000	6.900151000
1	12.362057000	9.260847000	7.110772000
6	10.687220000	12.535951000	3.660883000
6	10.436212000	11.465254000	2.794850000

6	11.340980000	12.313537000	4.875041000
1	9.915007000	11.635971000	1.849214000
1	11.512347000	13.153106000	5.548855000
6	10.845489000	10.177275000	3.154174000
6	11.776561000	11.029453000	5.249131000
1	10.639802000	9.335093000	2.487892000
6	11.515632000	9.967554000	4.366151000
1	11.833366000	8.955249000	4.635732000
1	10.361146000	13.544349000	3.389422000
8	11.527644000	11.442514000	8.043217000
6	10.252121000	11.118041000	8.118713000
8	9.508088000	11.530556000	8.977626000
1	9.904857000	10.428500000	7.331667000

12‘

27	14.622576000	11.778544000	7.456272000
1	14.527001000	10.671246000	6.454997000
15	14.834619000	12.949300000	5.697739000
7	16.598912000	13.207722000	5.763454000
6	17.287940000	12.590945000	6.755105000
7	18.609098000	12.751741000	6.897496000
6	19.165604000	12.160587000	7.963933000
7	18.507183000	11.393593000	8.848879000
6	17.197571000	11.244404000	8.635040000
7	16.554954000	11.838022000	7.602904000
7	16.412709000	10.501738000	9.454596000
15	14.773699000	10.181516000	8.829680000
7	13.832461000	9.838377000	10.156823000
6	13.595793000	8.405252000	10.351628000
1	12.569709000	8.142799000	10.043336000
1	13.717339000	8.118810000	11.408878000
6	14.610369000	7.670886000	9.469281000
1	15.528519000	7.439148000	10.041190000
1	14.197275000	6.714957000	9.111961000
7	14.868355000	8.575407000	8.355127000
6	15.737084000	8.161383000	7.247049000
1	15.915425000	9.078133000	6.664222000
6	17.098050000	7.621035000	7.705946000
1	17.644320000	8.364808000	8.303984000
1	17.718021000	7.371139000	6.831047000
1	16.993699000	6.702682000	8.306531000
6	15.003156000	7.182140000	6.328853000
1	14.721869000	6.260712000	6.864795000
1	15.642474000	6.892370000	5.480042000
1	14.085889000	7.645264000	5.933411000
6	12.846206000	10.804903000	10.667877000
1	13.153178000	11.777557000	10.267646000
6	12.892250000	10.906112000	12.191252000

1	12.588497000	9.959892000	12.669182000
1	12.199088000	11.687834000	12.537894000
1	13.904698000	11.166655000	12.536477000
6	11.430381000	10.540763000	10.144382000
1	11.437287000	10.429216000	9.049305000
1	10.778573000	11.390621000	10.395780000
1	10.983643000	9.637893000	10.589971000
7	14.216182000	14.441484000	5.292794000
6	13.735047000	14.562010000	3.921004000
1	14.063381000	15.511175000	3.465722000
1	12.631444000	14.542672000	3.890042000
6	14.314467000	13.365073000	3.165608000
1	13.620170000	13.021388000	2.384695000
1	15.261703000	13.635536000	2.663108000
7	14.511001000	12.329716000	4.173606000
6	14.869281000	10.975123000	3.749949000
1	15.028614000	10.412979000	4.682442000
6	13.698967000	10.312371000	3.018031000
1	12.802706000	10.314592000	3.657407000
1	13.943397000	9.269634000	2.760050000
1	13.454103000	10.834710000	2.078648000
6	16.174435000	10.933124000	2.947813000
1	16.092475000	11.493494000	2.002025000
1	16.437737000	9.893954000	2.694659000
1	17.002645000	11.362861000	3.531619000
6	13.880295000	15.478408000	6.272018000
1	14.065927000	15.033427000	7.252518000
6	14.803796000	16.689654000	6.145307000
1	15.849821000	16.389573000	6.304094000
1	14.551758000	17.445962000	6.904457000
1	14.714274000	17.163054000	5.153295000
6	12.397911000	15.853931000	6.234014000
1	12.136396000	16.430839000	5.332081000
1	12.150884000	16.470530000	7.110963000
1	11.768307000	14.952450000	6.271896000
6	17.308259000	14.103797000	4.860715000
1	17.971707000	14.766267000	5.433856000
1	16.573277000	14.717088000	4.328699000
1	17.916191000	13.545846000	4.130718000
6	20.610270000	12.383542000	8.204083000
6	21.339166000	13.260740000	7.382494000
1	20.823282000	13.754215000	6.558402000
6	22.688245000	13.504152000	7.639918000
1	23.246919000	14.195021000	7.003773000
6	23.324387000	12.869798000	8.714126000
1	24.377200000	13.075414000	8.925630000
6	22.608144000	11.982251000	9.526320000
1	23.105233000	11.480149000	10.360201000
6	21.258303000	11.739907000	9.273572000
1	20.681186000	11.060591000	9.901415000

6	16.946462000	9.945359000	10.686896000
1	17.485115000	8.997770000	10.520060000
1	16.109851000	9.764876000	11.374255000
1	17.643108000	10.659691000	11.146174000
1	13.146009000	11.831442000	7.435771000
14	14.653229000	13.384152000	9.056850000
1	15.223373000	12.947014000	10.381043000
6	12.097015000	16.201787000	10.803932000
6	10.826832000	15.782383000	10.388311000
6	13.234572000	15.506619000	10.383470000
1	9.937187000	16.320694000	10.726882000
1	14.219502000	15.842466000	10.721513000
6	10.705249000	14.677245000	9.539439000
6	13.131082000	14.384531000	9.540031000
1	9.719262000	14.345639000	9.200655000
6	11.850144000	13.994207000	9.115746000
1	11.755747000	13.138238000	8.442986000
1	12.199882000	17.068976000	11.462180000
8	15.815532000	14.645555000	8.699109000
6	17.037436000	14.772394000	9.172111000
1	17.309800000	14.019487000	9.931225000
8	17.809495000	15.630353000	8.815780000

14‘

27	14.800503000	11.586912000	7.195650000
1	12.579348000	9.834557000	6.627250000
15	15.078687000	12.771976000	5.397041000
7	16.833011000	12.932961000	5.476661000
6	17.479478000	12.285504000	6.478272000
7	18.799299000	12.404348000	6.634848000
6	19.336266000	11.763828000	7.682632000
7	18.657514000	10.951386000	8.505975000
6	17.345686000	10.854526000	8.290329000
7	16.708882000	11.546305000	7.312820000
7	16.563053000	10.059645000	9.065024000
15	14.870491000	9.960200000	8.607194000
7	14.006008000	9.726834000	10.008132000
6	13.275296000	8.457737000	10.027288000
1	12.251856000	8.585788000	9.632481000
1	13.198566000	8.065917000	11.052817000
6	14.076827000	7.521311000	9.128924000
1	14.878013000	7.016192000	9.702205000
1	13.435168000	6.741863000	8.691891000
7	14.613783000	8.391410000	8.090587000
6	15.311521000	7.827834000	6.931901000
1	15.708751000	8.693274000	6.378530000
6	16.497087000	6.946292000	7.338109000
1	17.205873000	7.508303000	7.963914000

1	17.036523000	6.594307000	6.444997000
1	16.168424000	6.057146000	7.900450000
6	14.328753000	7.106891000	6.007721000
1	13.843299000	6.259548000	6.518704000
1	14.848688000	6.710137000	5.121840000
1	13.542937000	7.799082000	5.670723000
6	13.603673000	10.809156000	10.917433000
1	14.142861000	11.703268000	10.578137000
6	14.053665000	10.506942000	12.350745000
1	13.510404000	9.643967000	12.769133000
1	13.851179000	11.371005000	13.002294000
1	15.131394000	10.286576000	12.390367000
6	12.106802000	11.109238000	10.837542000
1	11.794636000	11.275238000	9.797006000
1	11.877336000	12.020428000	11.408396000
1	11.507597000	10.286768000	11.260015000
7	14.540133000	14.291350000	4.963361000
6	13.892655000	14.342886000	3.654722000
1	14.143454000	15.279502000	3.132221000
1	12.794267000	14.292649000	3.754345000
6	14.419116000	13.135888000	2.884279000
1	13.667867000	12.762394000	2.172310000
1	15.320355000	13.404109000	2.302115000
7	14.713943000	12.132635000	3.899640000
6	15.052938000	10.768523000	3.486871000
1	15.438111000	10.268283000	4.388263000
6	13.797072000	10.014804000	3.042489000
1	13.067500000	9.975412000	3.865272000
1	14.042518000	8.984266000	2.739541000
1	13.318000000	10.509209000	2.181702000
6	16.162141000	10.751922000	2.430717000
1	15.820526000	11.182181000	1.475198000
1	16.487632000	9.719050000	2.232133000
1	17.036798000	11.326495000	2.773679000
6	14.262550000	15.359468000	5.932856000
1	14.769018000	15.059259000	6.859348000
6	14.881948000	16.686247000	5.480342000
1	15.952831000	16.569183000	5.251149000
1	14.777589000	17.441610000	6.274287000
1	14.379816000	17.082763000	4.582546000
6	12.767900000	15.494751000	6.234946000
1	12.215386000	15.906681000	5.375021000
1	12.612144000	16.172264000	7.087385000
1	12.338727000	14.517645000	6.497871000
6	17.599455000	13.753424000	4.546894000
1	18.222344000	14.474351000	5.095805000
1	16.897714000	14.305181000	3.911564000
1	18.257819000	13.133847000	3.918149000
6	20.769008000	11.999635000	7.973862000
6	21.509005000	12.907493000	7.195344000

1	21.017252000	13.399445000	6.355532000
6	22.838442000	13.184204000	7.513354000
1	23.404389000	13.898640000	6.910388000
6	23.445465000	12.552269000	8.605627000
1	24.481503000	12.785413000	8.866042000
6	22.720004000	11.633478000	9.373893000
1	23.194543000	11.134744000	10.222692000
6	21.389183000	11.357064000	9.060463000
1	20.804756000	10.655905000	9.657190000
6	17.150412000	9.349652000	10.192162000
1	17.893808000	8.609772000	9.860342000
1	16.345716000	8.842405000	10.736719000
1	17.647457000	10.059676000	10.870014000
1	14.778767000	10.479616000	6.184818000
14	15.218542000	13.287982000	8.770988000
1	16.033880000	12.754779000	9.919170000
1	16.197088000	14.290402000	8.218091000
6	13.249170000	16.089721000	11.179649000
6	11.893780000	15.852953000	10.914899000
6	14.227198000	15.332975000	10.528374000
1	11.126370000	16.441075000	11.425477000
1	15.281928000	15.527023000	10.750920000
6	11.529870000	14.863663000	9.996138000
6	13.878952000	14.328900000	9.601184000
1	10.474932000	14.668034000	9.782591000
6	12.514255000	14.112532000	9.344186000
1	12.226787000	13.343147000	8.625958000
1	13.542803000	16.863134000	11.895308000
6	12.075969000	10.782101000	6.899904000
8	10.850511000	10.837077000	6.930059000
8	12.865995000	11.755318000	7.178175000

14‘‘

27	15.007846000	11.129603000	6.932865000
1	17.370886000	9.694163000	5.422608000
15	15.159970000	12.622296000	5.315254000
7	16.913498000	12.485440000	5.097263000
6	17.620736000	12.122779000	6.192199000
7	18.928405000	12.379477000	6.298963000
6	19.521978000	11.967116000	7.424784000
7	18.924405000	11.222790000	8.368596000
6	17.623658000	10.982510000	8.187607000
7	16.914058000	11.499775000	7.166569000
7	16.945414000	10.177663000	9.058244000
15	15.285829000	9.762621000	8.648026000
7	14.484669000	9.648995000	10.117912000
6	13.794577000	8.368519000	10.262364000
1	12.782139000	8.403707000	9.819846000

1	13.689682000	8.105856000	11.324599000
6	14.668193000	7.357610000	9.532299000
1	15.442427000	6.954974000	10.212806000
1	14.075322000	6.508313000	9.158661000
7	15.257210000	8.104537000	8.428087000
6	16.078003000	7.402217000	7.430694000
1	16.662508000	8.174966000	6.916812000
6	17.083720000	6.448901000	8.087258000
1	17.710234000	6.971211000	8.825212000
1	17.746388000	6.025927000	7.317046000
1	16.583616000	5.607345000	8.593050000
6	15.206740000	6.693357000	6.396548000
1	14.572694000	5.930426000	6.877825000
1	15.833388000	6.196652000	5.640627000
1	14.571404000	7.426319000	5.882122000
6	14.372695000	10.657165000	11.183606000
1	14.831694000	11.573149000	10.791080000
6	15.151977000	10.239321000	12.440424000
1	14.729657000	9.321551000	12.881811000
1	15.085028000	11.030046000	13.203746000
1	16.213284000	10.057766000	12.229233000
6	12.918509000	10.964967000	11.560365000
1	12.328284000	11.244010000	10.679670000
1	12.885382000	11.802529000	12.274143000
1	12.442269000	10.098073000	12.046755000
7	14.993765000	14.280835000	5.355905000
6	14.372757000	14.862478000	4.171675000
1	14.945040000	15.735173000	3.815913000
1	13.344325000	15.205091000	4.388165000
6	14.373664000	13.753956000	3.109164000
1	13.459239000	13.791462000	2.501733000
1	15.230816000	13.870742000	2.422021000
7	14.441946000	12.484913000	3.838051000
6	13.910614000	11.247861000	3.237479000
1	14.091189000	10.459150000	3.979234000
6	12.402560000	11.363015000	3.000423000
1	11.886442000	11.661439000	3.926098000
1	11.997224000	10.394984000	2.666183000
1	12.158256000	12.100371000	2.218293000
6	14.669231000	10.874775000	1.961037000
1	14.565560000	11.651136000	1.183773000
1	14.270748000	9.934643000	1.549611000
1	15.735388000	10.710980000	2.173794000
6	15.516997000	15.117042000	6.439661000
1	15.613416000	14.455048000	7.311044000
6	16.909924000	15.692073000	6.143394000
1	17.668046000	14.901048000	6.059202000
1	17.219724000	16.365301000	6.957672000
1	16.911349000	16.277341000	5.208862000
6	14.528974000	16.220302000	6.818725000

1	14.448967000	16.979994000	6.023946000
1	14.868240000	16.723892000	7.735740000
1	13.530638000	15.801137000	7.007500000
6	17.567112000	12.988132000	3.899431000
1	18.635612000	12.745839000	3.943160000
1	17.451750000	14.079195000	3.801447000
1	17.120167000	12.501162000	3.021859000
6	20.921482000	12.389440000	7.660797000
6	21.544982000	13.293503000	6.782158000
1	20.992853000	13.643752000	5.909229000
6	22.838939000	13.745682000	7.043211000
1	23.314220000	14.456777000	6.362856000
6	23.524341000	13.293024000	8.177233000
1	24.533046000	13.658387000	8.388287000
6	22.916086000	12.377848000	9.044869000
1	23.455090000	12.015788000	9.923783000
6	21.619634000	11.929154000	8.790927000
1	21.124837000	11.226010000	9.461935000
6	17.708818000	9.499285000	10.100097000
1	18.554626000	8.947844000	9.665248000
1	17.045899000	8.799564000	10.619315000
1	18.110704000	10.224675000	10.823128000
1	13.548892000	10.894010000	6.796612000
14	13.736242000	12.522586000	8.118930000
1	12.954979000	13.456328000	7.250200000
1	12.665298000	11.854499000	8.905482000
6	14.397844000	15.691272000	10.803437000
6	15.723492000	15.519133000	11.218548000
6	13.826471000	14.798155000	9.890876000
1	16.173160000	16.216577000	11.930205000
1	12.793052000	14.958255000	9.567264000
6	16.467713000	14.449601000	10.711654000
6	14.554856000	13.712884000	9.369007000
1	17.503208000	14.297765000	11.029611000
6	15.886872000	13.567084000	9.796562000
1	16.485314000	12.746163000	9.407098000
1	13.807958000	16.525715000	11.192590000
6	16.436382000	9.393508000	4.901525000
8	16.532862000	8.765311000	3.854621000
8	15.344089000	9.724006000	5.485150000

20’

27	17.982611000	12.323965000	7.936392000
1	15.962339000	12.192523000	10.183674000
15	18.012717000	10.605549000	9.243972000
7	19.215525000	9.642636000	8.382292000
6	19.992713000	10.314086000	7.497129000
7	21.112462000	9.771226000	7.011197000

6	21.833188000	10.541429000	6.185553000
7	21.499440000	11.785581000	5.816404000
6	20.373178000	12.279204000	6.340215000
7	19.581693000	11.564098000	7.177534000
7	19.977420000	13.552840000	6.084911000
15	18.661078000	14.169282000	7.089291000
7	17.846460000	15.269091000	6.136608000
6	17.961088000	16.644004000	6.626185000
1	17.110817000	16.902156000	7.282163000
1	17.977018000	17.361591000	5.790341000
6	19.260387000	16.684108000	7.429200000
1	20.124881000	16.900787000	6.772287000
1	19.226240000	17.469692000	8.198190000
7	19.346246000	15.363486000	8.041469000
6	20.296392000	15.084914000	9.120792000
1	20.042525000	14.070583000	9.462271000
6	21.758706000	15.074359000	8.656949000
1	21.923495000	14.325334000	7.868762000
1	22.423066000	14.821385000	9.498385000
1	22.069873000	16.058742000	8.268771000
6	20.078392000	16.017212000	10.312205000
1	20.384231000	17.052910000	10.089193000
1	20.679135000	15.672128000	11.167800000
1	19.020239000	16.012399000	10.613003000
6	16.692840000	14.880335000	5.311272000
1	16.720135000	13.784459000	5.278636000
6	16.841143000	15.386698000	3.875084000
1	16.792638000	16.487800000	3.827476000
1	16.024876000	14.994315000	3.248892000
1	17.796534000	15.056277000	3.438653000
6	15.351970000	15.290738000	5.925762000
1	15.309121000	14.973881000	6.978127000
1	14.532570000	14.796279000	5.382256000
1	15.189006000	16.379505000	5.861225000
7	16.760588000	9.632807000	9.733036000
6	16.922072000	9.079979000	11.079193000
1	17.380594000	8.073200000	11.058013000
1	15.947064000	8.996510000	11.577439000
6	17.821571000	10.074020000	11.814563000
1	17.213833000	10.848137000	12.309119000
1	18.419664000	9.569416000	12.589886000
7	18.676311000	10.640397000	10.781186000
6	19.856032000	11.432710000	11.122425000
1	20.228897000	11.841350000	10.169633000
6	19.505922000	12.621928000	12.019934000
1	18.763716000	13.258611000	11.520693000
1	20.402650000	13.223710000	12.235374000
1	19.088029000	12.286536000	12.982510000
6	20.967922000	10.560037000	11.711453000
1	20.652674000	10.085843000	12.655727000

1	21.859794000	11.169363000	11.926493000
1	21.256325000	9.764268000	11.008845000
6	15.645702000	9.241444000	8.866215000
1	15.710068000	9.903095000	7.994960000
6	15.775721000	7.798811000	8.371791000
1	16.733096000	7.649169000	7.849836000
1	14.965921000	7.566738000	7.663703000
1	15.712819000	7.080523000	9.206924000
6	14.287994000	9.504139000	9.520143000
1	14.114339000	8.851933000	10.391662000
1	13.486697000	9.314130000	8.791476000
1	14.211577000	10.550845000	9.848784000
6	19.589172000	8.296392000	8.792479000
1	19.860609000	7.699141000	7.912114000
1	18.730872000	7.829902000	9.288237000
1	20.445691000	8.299786000	9.484396000
6	23.112580000	9.992356000	5.677166000
6	23.624711000	8.792589000	6.200636000
1	23.055900000	8.274155000	6.973418000
6	24.840401000	8.286551000	5.740474000
1	25.238618000	7.360537000	6.163118000
6	25.553411000	8.967563000	4.746430000
1	26.500055000	8.564148000	4.377235000
6	25.049391000	10.163335000	4.221808000
1	25.606251000	10.700203000	3.449723000
6	23.841869000	10.680038000	4.691723000
1	23.443373000	11.617001000	4.301132000
6	20.751226000	14.401645000	5.188972000
1	21.598787000	14.875927000	5.709623000
1	20.088095000	15.184410000	4.799207000
1	21.146155000	13.806621000	4.356069000
6	14.931829000	10.529031000	14.454743000
6	15.992024000	11.307566000	14.930477000
6	14.223180000	10.937035000	13.318223000
1	16.547139000	10.990196000	15.817422000
1	13.394821000	10.333355000	12.938347000
6	16.347950000	12.486758000	14.265076000
6	14.578633000	12.119050000	12.664913000
1	17.186825000	13.080117000	14.640462000
1	14.009772000	12.426060000	11.783374000
6	15.653686000	12.910250000	13.117344000
1	14.652846000	9.607192000	14.971932000
14	16.175128000	14.424386000	12.147946000
1	15.002141000	15.339892000	12.043420000
1	17.273274000	15.107635000	12.874142000
8	16.728081000	14.045726000	10.654489000
6	15.939439000	13.199111000	9.739774000
1	14.901362000	13.579906000	9.788575000
8	16.382932000	13.252511000	8.487136000
6	14.527676000	9.952172000	5.342472000

6	15.093906000	11.059791000	6.006123000
6	13.153083000	9.697608000	5.389106000
1	12.738106000	8.827511000	4.872185000
6	14.224142000	11.914564000	6.711414000
6	12.309276000	10.550635000	6.110300000
1	14.654137000	12.753047000	7.259904000
1	11.237347000	10.340186000	6.167937000
6	12.848704000	11.663187000	6.764337000
1	12.196699000	12.331481000	7.334567000
1	15.177799000	9.262266000	4.796348000
14	16.978281000	11.283343000	6.081712000
1	18.751178000	12.871444000	9.090395000
1	17.483023000	9.878011000	5.881019000
1	17.378002000	11.888698000	4.760510000

20’’

27	17.974438000	11.954849000	8.293080000
1	20.785555000	11.906657000	9.544121000
15	17.841438000	9.983283000	9.195373000
7	19.009234000	9.170916000	8.132430000
6	19.910998000	9.978616000	7.504169000
7	21.045426000	9.502314000	6.980367000
6	21.874893000	10.411748000	6.449100000
7	21.626877000	11.727097000	6.362751000
6	20.453351000	12.133373000	6.863109000
7	19.596455000	11.288218000	7.469012000
7	20.050551000	13.426887000	6.765163000
15	18.354819000	13.780569000	7.121325000
7	17.863537000	14.320596000	5.601237000
6	17.231634000	15.634651000	5.630376000
1	16.132742000	15.555743000	5.724232000
1	17.455743000	16.185553000	4.705746000
6	17.835091000	16.336863000	6.840683000
1	18.752769000	16.889084000	6.566962000
1	17.131847000	17.060404000	7.274863000
7	18.113038000	15.270225000	7.802822000
6	17.975429000	15.550746000	9.240800000
1	18.305551000	14.637368000	9.751870000
6	18.903659000	16.695954000	9.643796000
1	19.941465000	16.453332000	9.370994000
1	18.860363000	16.855831000	10.732666000
1	18.620595000	17.644740000	9.157129000
6	16.514015000	15.819086000	9.617310000
1	16.098861000	16.694115000	9.089964000
1	16.426490000	16.017837000	10.697600000
1	15.890048000	14.944474000	9.379722000
6	18.165254000	13.693962000	4.307661000
1	18.677238000	12.751240000	4.542013000

6	19.116408000	14.537338000	3.446384000
1	18.618179000	15.440549000	3.058619000
1	19.444980000	13.949095000	2.575020000
1	20.007521000	14.849794000	4.003341000
6	16.894910000	13.371311000	3.516259000
1	16.201777000	12.760642000	4.103279000
1	17.150713000	12.822681000	2.595779000
1	16.371127000	14.295945000	3.221795000
7	16.427894000	9.093571000	9.279480000
6	16.510936000	8.050329000	10.311513000
1	16.950894000	7.130325000	9.893517000
1	15.509667000	7.797738000	10.682148000
6	17.409838000	8.614862000	11.408838000
1	16.829064000	9.216745000	12.133213000
1	17.911127000	7.808889000	11.966843000
7	18.385088000	9.423572000	10.685389000
6	19.665712000	9.764295000	11.306293000
1	20.199978000	10.386919000	10.579752000
6	19.459943000	10.586953000	12.582257000
1	18.853008000	11.477515000	12.363657000
1	20.426767000	10.912937000	12.996684000
1	18.949857000	9.990667000	13.356827000
6	20.542820000	8.533705000	11.565529000
1	20.075530000	7.840175000	12.282577000
1	21.508302000	8.850251000	11.991288000
1	20.747553000	7.982523000	10.636157000
6	15.114485000	9.731093000	9.056073000
1	15.293149000	10.564768000	8.371200000
6	14.152866000	8.766273000	8.370560000
1	14.620559000	8.340643000	7.470206000
1	13.236004000	9.296535000	8.069007000
1	13.863041000	7.937408000	9.037658000
6	14.529604000	10.355031000	10.327902000
1	14.255560000	9.597493000	11.079481000
1	13.618071000	10.921190000	10.080405000
1	15.249667000	11.054481000	10.779658000
6	19.150585000	7.724080000	8.101255000
1	18.192760000	7.272817000	7.802598000
1	19.437835000	7.326254000	9.086133000
1	19.922668000	7.453066000	7.371930000
6	23.172811000	9.920644000	5.928780000
6	23.635514000	8.642878000	6.286374000
1	23.018962000	8.027864000	6.942486000
6	24.870080000	8.186221000	5.825659000
1	25.233262000	7.200597000	6.128339000
6	25.646888000	8.991968000	4.984135000
1	26.606714000	8.626824000	4.608970000
6	25.189254000	10.263838000	4.620198000
1	25.793420000	10.894901000	3.963580000
6	23.965131000	10.732364000	5.099931000

1	23.602593000	11.725713000	4.833559000
6	21.001071000	14.446721000	6.355991000
1	21.724673000	14.635268000	7.161397000
1	20.456792000	15.376013000	6.155513000
1	21.546921000	14.138734000	5.454108000
6	25.122053000	10.340977000	9.891422000
6	24.818653000	11.016329000	8.703838000
6	24.630190000	10.819138000	11.110138000
1	25.197259000	10.640089000	7.751776000
1	24.869438000	10.292962000	12.038480000
6	24.028095000	12.168920000	8.738107000
6	23.844388000	11.976400000	11.139166000
1	23.782429000	12.671751000	7.799047000
1	23.469748000	12.346762000	12.098372000
6	23.531181000	12.673162000	9.956050000
1	25.741248000	9.440315000	9.865460000
14	22.475711000	14.217052000	10.025410000
1	22.556215000	14.724915000	11.425251000
1	23.012691000	15.246695000	9.099875000
8	20.921129000	13.949902000	9.571141000
6	20.257043000	12.719539000	10.066658000
1	20.516125000	12.659556000	11.138592000
8	18.938848000	12.736765000	9.893442000
6	14.234685000	11.353040000	5.263975000
6	15.111870000	11.934987000	6.198620000
6	13.027691000	11.968204000	4.919204000
1	12.365721000	11.502662000	4.183683000
6	14.722920000	13.144898000	6.798549000
6	12.666570000	13.181664000	5.516607000
1	15.377413000	13.605317000	7.541118000
1	11.726784000	13.668732000	5.242464000
6	13.513878000	13.764607000	6.464594000
1	13.232890000	14.705475000	6.946547000
1	14.508678000	10.407968000	4.784583000
14	16.780831000	11.118949000	6.550980000
1	16.691114000	12.427909000	8.848298000
1	16.465088000	9.656929000	6.563990000
1	17.558974000	11.191926000	5.276632000

5 (BP86)

27	15.174265000	11.560706000	7.198131000
17	13.227980000	10.918291000	6.349215000
17	14.952753000	13.175245000	8.915848000
15	15.504267000	12.980019000	5.611245000
7	17.170367000	13.418861000	5.948635000
6	17.816111000	12.640808000	6.869927000
7	19.115350000	12.836253000	7.130764000
6	19.659554000	12.036698000	8.072107000

7	18.983743000	11.090144000	8.757031000
6	17.688655000	10.948597000	8.445688000
7	17.062281000	11.668312000	7.466390000
7	16.915418000	10.031289000	9.102883000
15	15.270248000	9.859007000	8.515700000
7	14.403912000	9.441169000	9.890347000
6	13.675310000	8.180150000	9.711618000
1	12.663846000	8.351381000	9.277870000
1	13.551523000	7.662138000	10.685237000
6	14.529557000	7.350651000	8.742173000
1	15.309050000	6.769185000	9.292247000
1	13.902102000	6.625771000	8.184299000
7	15.107866000	8.340353000	7.842762000
6	15.694228000	8.021671000	6.540211000
1	16.003102000	9.010410000	6.129082000
6	16.947449000	7.144074000	6.673011000
1	17.696113000	7.622711000	7.335723000
1	17.419994000	6.975052000	5.684288000
1	16.697832000	6.148650000	7.097090000
6	14.647992000	7.438864000	5.583680000
1	14.282242000	6.450059000	5.931799000
1	15.082655000	7.291740000	4.574660000
1	13.788238000	8.132133000	5.504226000
6	13.858136000	10.503155000	10.763367000
1	14.535600000	11.372155000	10.619915000
6	13.916552000	10.068454000	12.231575000
1	13.240734000	9.211174000	12.436867000
1	13.598502000	10.901456000	12.889697000
1	14.945890000	9.775004000	12.520287000
6	12.458830000	10.954600000	10.326224000
1	12.466676000	11.241810000	9.255945000
1	12.146462000	11.842037000	10.911356000
1	11.697657000	10.160966000	10.481459000
7	14.780271000	14.446096000	5.235712000
6	14.146662000	14.419031000	3.912863000
1	14.145217000	15.433122000	3.461752000
1	13.092422000	14.064786000	3.972954000
6	14.984512000	13.438936000	3.078729000
1	14.372946000	12.985553000	2.271985000
1	15.847508000	13.958864000	2.595922000
7	15.412501000	12.429538000	4.038213000
6	15.916540000	11.106831000	3.665329000
1	16.124913000	10.607091000	4.639538000
6	14.841843000	10.280101000	2.950017000
1	13.934956000	10.219204000	3.582583000
1	15.208905000	9.253104000	2.751789000
1	14.568523000	10.726385000	1.970757000
6	17.232713000	11.194288000	2.878751000
1	17.084102000	11.703228000	1.903117000
1	17.636486000	10.183325000	2.668282000

1	17.997471000	11.759133000	3.448685000
6	14.220058000	15.284729000	6.317593000
1	14.811904000	15.023670000	7.221032000
6	14.428828000	16.770075000	6.003590000
1	15.498330000	16.991323000	5.813326000
1	14.095615000	17.391870000	6.858342000
1	13.844505000	17.092211000	5.115492000
6	12.759260000	14.935993000	6.627593000
1	12.080021000	15.208815000	5.792402000
1	12.422801000	15.483816000	7.529918000
1	12.659779000	13.851727000	6.833972000
6	17.904916000	14.442699000	5.217999000
1	18.486348000	15.069430000	5.921120000
1	17.166400000	15.072591000	4.689119000
1	18.612002000	13.997819000	4.487025000
6	21.099801000	12.207462000	8.372138000
6	21.861721000	13.178172000	7.683571000
1	21.355932000	13.797372000	6.929522000
6	23.224712000	13.335316000	7.966078000
1	23.812293000	14.093965000	7.426475000
6	23.842681000	12.527261000	8.937671000
1	24.913945000	12.652490000	9.158954000
6	23.089341000	11.559524000	9.626755000
1	23.570807000	10.926904000	10.388310000
6	21.726133000	11.398973000	9.347283000
1	21.116161000	10.650551000	9.872268000
6	17.508662000	9.176267000	10.122088000
1	18.199620000	8.428820000	9.679436000
1	16.684572000	8.660642000	10.648170000
1	18.081305000	9.787128000	10.846163000

5 (M06L)

27	15.091806000	11.515431000	7.140730000
17	13.106391000	10.881903000	6.300831000
17	14.895742000	13.165130000	8.911005000
15	15.466819000	12.970683000	5.555405000
7	17.127989000	13.371021000	5.919439000
6	17.751485000	12.601660000	6.852271000
7	19.035037000	12.815570000	7.131397000
6	19.568880000	12.028922000	8.073366000
7	18.898381000	11.088506000	8.749661000
6	17.617244000	10.935057000	8.419554000
7	17.003850000	11.629350000	7.429459000
7	16.854572000	10.020590000	9.076752000
15	15.211249000	9.819208000	8.509670000
7	14.381494000	9.409917000	9.891879000
6	13.657356000	8.153969000	9.743256000
1	12.640015000	8.314032000	9.336932000

1	13.551820000	7.652629000	10.718958000
6	14.489987000	7.321921000	8.777085000
1	15.268063000	6.742191000	9.316438000
1	13.864266000	6.595111000	8.235488000
7	15.062802000	8.294170000	7.865682000
6	15.671496000	7.939340000	6.584177000
1	15.959179000	8.910737000	6.134335000
6	16.935833000	7.109514000	6.762292000
1	17.673265000	7.625155000	7.394184000
1	17.413290000	6.901946000	5.794240000
1	16.716055000	6.136483000	7.228461000
6	14.668473000	7.286229000	5.649678000
1	14.340285000	6.305738000	6.027088000
1	15.117362000	7.111748000	4.661843000
1	13.784091000	7.925219000	5.522138000
6	13.921821000	10.454874000	10.825040000
1	14.643452000	11.281812000	10.704403000
6	13.996491000	9.953679000	12.256180000
1	13.272814000	9.147134000	12.452184000
1	13.765330000	10.766538000	12.957543000
1	14.999166000	9.575115000	12.501247000
6	12.551796000	10.990460000	10.450775000
1	12.544991000	11.347878000	9.411088000
1	12.285133000	11.841378000	11.092119000
1	11.767929000	10.225570000	10.567986000
7	14.778977000	14.441543000	5.195326000
6	14.133818000	14.437774000	3.888548000
1	14.132170000	15.451900000	3.456938000
1	13.081488000	14.100388000	3.953125000
6	14.950827000	13.472469000	3.039817000
1	14.338005000	13.026773000	2.239604000
1	15.798054000	13.993620000	2.546868000
7	15.403795000	12.460558000	3.974856000
6	15.986945000	11.186559000	3.556723000
1	16.314716000	10.704265000	4.499494000
6	14.950987000	10.279667000	2.915059000
1	14.099881000	10.125019000	3.592650000
1	15.388840000	9.301573000	2.668963000
1	14.571243000	10.706197000	1.974034000
6	17.216273000	11.388362000	2.682382000
1	16.954987000	11.868809000	1.726745000
1	17.691106000	10.427619000	2.439633000
1	17.968174000	12.018033000	3.178946000
6	14.311300000	15.326510000	6.278290000
1	14.947363000	15.075359000	7.145220000
6	14.548684000	16.780220000	5.910652000
1	15.598476000	16.962290000	5.639212000
1	14.304143000	17.433494000	6.758972000
1	13.919866000	17.103592000	5.066399000
6	12.875086000	15.034672000	6.672605000

1	12.169655000	15.285568000	5.864646000
1	12.593304000	15.623687000	7.555821000
1	12.752436000	13.973410000	6.933329000
6	17.888285000	14.395281000	5.229751000
1	18.278221000	15.139142000	5.939232000
1	17.225003000	14.902957000	4.519812000
1	18.747904000	13.968163000	4.692739000
6	21.000170000	12.213462000	8.386108000
6	21.755090000	13.188329000	7.713770000
1	21.260746000	13.807042000	6.962690000
6	23.104918000	13.358793000	8.004822000
1	23.683463000	14.120860000	7.476884000
6	23.720949000	12.560258000	8.970006000
1	24.781367000	12.695668000	9.197885000
6	22.978429000	11.589381000	9.644305000
1	23.458155000	10.964160000	10.401310000
6	21.628021000	11.416144000	9.356645000
1	21.035551000	10.662074000	9.877950000
6	17.462812000	9.212871000	10.115948000
1	18.281313000	8.591675000	9.722489000
1	16.689990000	8.569569000	10.552264000
1	17.881505000	9.848160000	10.909241000

5 (B3LYP)

27	15.149844000	11.597354000	7.221749000
17	13.186346000	10.974270000	6.322994000
17	14.995587000	13.255590000	8.989588000
15	15.528013000	13.039113000	5.625749000
7	17.188213000	13.448877000	5.991382000
6	17.823676000	12.648414000	6.889991000
7	19.123283000	12.819305000	7.135435000
6	19.663972000	12.015992000	8.060122000
7	18.988833000	11.079859000	8.740160000
6	17.693572000	10.952005000	8.440685000
7	17.080417000	11.685088000	7.482853000
7	16.925376000	10.039179000	9.097507000
15	15.270504000	9.860873000	8.552832000
7	14.426367000	9.426840000	9.921834000
6	13.774696000	8.122812000	9.775006000
1	12.749720000	8.230033000	9.375586000
1	13.716505000	7.606388000	10.745601000
6	14.638093000	7.341918000	8.782610000
1	15.464299000	6.815284000	9.300560000
1	14.042862000	6.584978000	8.250923000
7	15.122554000	8.358677000	7.863004000
6	15.598688000	8.054723000	6.516154000
1	15.853996000	9.032070000	6.073607000
6	16.872446000	7.204667000	6.543568000

1	17.662053000	7.706846000	7.124368000
1	17.253055000	7.028760000	5.524757000
1	16.683537000	6.220124000	7.003765000
6	14.483990000	7.450767000	5.659071000
1	14.156689000	6.472411000	6.048423000
1	14.834635000	7.292291000	4.627042000
1	13.621857000	8.132721000	5.639669000
6	13.848316000	10.448617000	10.817501000
1	14.498340000	11.331079000	10.713969000
6	13.891947000	9.967253000	12.268350000
1	13.210740000	9.116205000	12.433874000
1	13.571067000	10.775193000	12.943473000
1	14.907869000	9.653787000	12.557313000
6	12.440294000	10.872421000	10.391144000
1	12.437025000	11.199191000	9.340556000
1	12.101014000	11.718792000	11.007157000
1	11.711512000	10.054094000	10.517959000
7	14.816649000	14.486021000	5.225803000
6	14.330661000	14.507357000	3.844522000
1	14.455329000	15.510190000	3.406574000
1	13.261069000	14.233953000	3.796548000
6	15.168119000	13.476960000	3.080235000
1	14.593536000	13.045385000	2.246729000
1	16.077654000	13.941982000	2.650230000
7	15.485069000	12.463131000	4.070500000
6	15.925510000	11.115661000	3.718839000
1	16.096547000	10.609523000	4.683850000
6	14.820871000	10.341533000	2.996046000
1	13.917942000	10.315284000	3.622718000
1	15.143812000	9.307762000	2.793920000
1	14.570561000	10.803577000	2.026717000
6	17.253089000	11.131292000	2.955067000
1	17.150730000	11.645876000	1.985054000
1	17.602438000	10.106592000	2.751360000
1	18.030712000	11.651772000	3.535699000
6	14.164494000	15.317863000	6.259109000
1	14.738097000	15.139127000	7.181323000
6	14.267866000	16.797626000	5.890956000
1	15.308570000	17.084038000	5.670072000
1	13.906627000	17.418645000	6.724416000
1	13.648389000	17.041815000	5.012054000
6	12.720571000	14.888037000	6.531302000
1	12.074309000	15.050672000	5.652330000
1	12.305118000	15.474572000	7.364562000
1	12.680284000	13.826091000	6.812708000
6	17.948770000	14.448038000	5.256811000
1	18.546581000	15.056120000	5.949754000
1	17.237979000	15.096967000	4.730499000
1	18.633430000	13.979447000	4.530717000
6	21.106131000	12.186686000	8.351464000

6	21.854611000	13.152454000	7.655801000
1	21.351269000	13.753294000	6.897993000
6	23.205437000	13.336648000	7.945984000
1	23.781216000	14.091463000	7.404628000
6	23.823246000	12.556773000	8.930271000
1	24.878462000	12.714753000	9.169554000
6	23.087857000	11.583311000	9.616322000
1	23.572591000	10.969619000	10.379710000
6	21.736178000	11.396274000	9.327853000
1	21.142819000	10.648741000	9.855114000
6	17.536447000	9.170234000	10.092301000
1	18.193142000	8.419816000	9.622478000
1	16.733446000	8.666395000	10.643658000
1	18.139953000	9.763203000	10.792919000

10. Literature

(1) Cramer, H. H.; Chatterjee, B.; Weyhermüller, T.; Werlé, C.; Leitner, W., Controlling the Product Platform of Carbon Dioxide Reduction: Adaptive Catalytic Hydrosilylation of CO_2 Using a Molecular Cobalt(II) Triazine Complex. *Angew. Chem., Int. Ed.* **2020,** *59* (36), 15674-15681.

(2) Scheuermann, M. L.; Semproni, S. P.; Pappas, I.; Chirik, P. J., Carbon Dioxide Hydrosilylation Promoted by Cobalt Pincer Complexes. *Inorg. Chem.* **2014,** *53* (18), 9463-9465.

(3) Bertini, F.; Glatz, M.; Stoger, B.; Peruzzini, M.; Veiros, L. F.; Kirchner, K.; Gonsalvi, L., Carbon Dioxide Reduction to Methanol Catalyzed by Mn(I) PNP Pincer Complexes under Mild Reaction Conditions. *ACS Catal.* **2019,** *9* (1), 632-639.

(4) Neese, F., The ORCA program system. *Wiley Interdiscip. Rev. Comput. Mol. Sci.* **2012,** *2* (1), 73-78.

(5) Neese, F., Software update: the ORCA program system, version 4.0. *Wiley Interdiscip. Rev. Comput. Mol. Sci.* **2018,** *8* (1), e1327.

(6) Becke, A. D., Density-functional exchange-energy approximation with correct asymptotic behavior. *Phys. Rev. A* **1988,** *38* (6), 3098-3100.

(7) Lee, C.; Yang, W.; Parr, R. G., Development of the Colle-Salvetti correlation-energy formula into a functional of the electron density. *Phys. Rev. B* **1988,** *37* (2), 785-789.

(8) Becke, A. D., Density-functional thermochemistry. III. The role of exact exchange. *J. Chem. Phys.* **1993,** *98* (7), 5648-5652.

(9) Grimme, S.; Antony, J.; Ehrlich, S.; Krieg, H., A consistent and accurate ab initio parametrization of density functional dispersion correction (DFT-D) for the 94 elements H-Pu. *J. Chem. Phys.* **2010,** *132* (15), 154104.

(10) Grimme, S.; Ehrlich, S.; Goerigk, L., Effect of the damping function in dispersion corrected density functional theory. *J. Comput. Chem.* **2011,** *32* (7), 1456-1465.

(11) Neese, F.; Wennmohs, F.; Hansen, A.; Becker, U., Efficient, approximate and parallel Hartree-Fock and hybrid DFT calculations. A 'chain-of-spheres' algorithm for the Hartree-Fock exchange. *Chem. Phys.* **2009,** *356* (1-3), 98-109.

(12) Weigend, F., Accurate Coulomb-fitting basis sets for H to Rn. *Phys. Chem. Chem. Phys.* **2006,** *8* (9), 1057-1065.

(13) Weigend, F.; Ahlrichs, R., Balanced basis sets of split valence, triple zeta valence and quadruple zeta valence quality for H to Rn: Design and assessment of accuracy. *Phys. Chem. Chem. Phys.* **2005,** *7* (18), 3297-3305.

(14) Besora, M.; Maseras, F., Microkinetic modeling in homogeneous catalysis. *Wiley Interdiscip. Rev. Comput. Mol. Sci.* **2018,** *8* (6), e1372.
